# Nanoporous Gold: From Structure Evolution to Functional Properties in Catalysis and Electrochemistry

**DOI:** 10.1021/acs.chemrev.2c00751

**Published:** 2023-05-03

**Authors:** Gunther Wittstock, Marcus Bäumer, Wilke Dononelli, Thorsten Klüner, Lukas Lührs, Christoph Mahr, Lyudmila V. Moskaleva, Mehtap Oezaslan, Thomas Risse, Andreas Rosenauer, Anne Staubitz, Jörg Weissmüller, Arne Wittstock

**Affiliations:** † Carl von Ossietzky University of Oldenburg, School of Mathematics and Science, Institute of Chemistry, D-26111 Oldenburg, Germany; ‡ University of Bremen, Institute for Applied and Physical Chemistry, 28359 Bremen, Germany; § University of Bremen, MAPEX Center for Materials and Processes, 28359 Bremen, Germany; ∥ University of Bremen, Bremen Center for Computational Materials Science, Hybrid Materials Interfaces Group, Am Fallturm 1, Bremen 28359, Germany; ⊥ Hamburg University of Technology, Institute of Materials Physics and Technology, 21703 Hamburg, Germany; # University of Bremen, Institute of Solid State Physics, Otto Hahn Allee 1, 28359 Bremen, Germany; ∇ University of the Free State, Department of Chemistry, P.O. Box 339, Bloemfontein 9300, South Africa; ○ Technical University of Braunschweig Institute of Technical Chemistry, Technical Electrocatalysis Laboratory, Franz-Liszt-Strasse 35a, 38106 Braunschweig, Germany; ◆ Freie Universität Berlin, Institute of Chemistry and Biochemistry, Arnimallee 22, 14195 Berlin, Germany; ¶ University of Bremen, Institute for Organic and Analytical Chemistry, Leobener Strasse 7, D-28359 Bremen, Germany; + Helmholtz-Zentrum Hereon, Institute of Materials Mechanics, 21502 Geesthacht, Germany

## Abstract

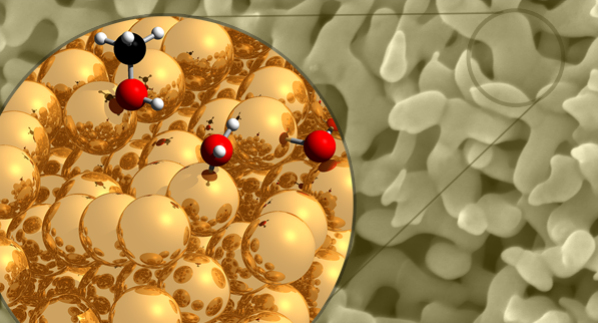

Nanoporous gold (NPG) is characterized by a bicontinuous network of nanometer-sized metallic struts and interconnected pores formed spontaneously by oxidative dissolution of the less noble element from gold alloys. The resulting material exhibits decent catalytic activity for low-temperature, aerobic total as well as partial oxidation reactions, the oxidative coupling of methanol to methyl formate being the prototypical example. This review not only provides a critical discussion of ways to tune the morphology and composition of this material and its implication for catalysis and electrocatalysis, but will also exemplarily review the current mechanistic understanding of the partial oxidation of methanol using information from quantum chemical studies, model studies on single-crystal surfaces, gas phase catalysis, aerobic liquid phase oxidation, and electrocatalysis. In this respect, a particular focus will be on mechanistic aspects not well understood, yet. Apart from the mechanistic aspects of catalysis, best practice examples with respect to material preparation and characterization will be discussed. These can improve the reproducibility of the materials property such as the catalytic activity and selectivity as well as the scope of reactions being identified as the main challenges for a broader application of NPG in target-oriented organic synthesis.

## 1 Introduction

Nanostructuring is one of the most successful strategies to prepare materials with new or improved chemical properties. Catalysis, being one of the core technologies of chemical industry and being involved in about 80–90% of all industrial processes, is closely linked to the current quest to reduce our energy and CO_2_ footprint. Heterogeneous catalysis has used nanostructuring early on as seen by the large fraction of heterogeneous catalysts which are nanostructured materials. Heterogeneous gold catalysts are a prototypical example for appearance of new chemical functionality upon nanostructuring. In particular, gold nanoparticles grafted on a suitable oxide support were found to be good catalysts for a range of low temperature oxidation reactions back in the 1980s of the last century,^1−378^ while macroscopic gold is inactive. The size (maximum activity for CO oxidation is found at a diameter of around 5 nm) as well as the support of the Au nanoparticles were identified as crucial parameters for the catalytic activity. Hence, the first reports about a high catalytic activity of nanoporous gold (NPG) for CO oxidation in 2006/2007 by some of the authors^5^ as well as by Ding and co-workers^6^ came as a surprise because NPG not only lacks a support but also exhibits characteristic length scales (ligament and pore sizes) that are about an order of magnitude larger than the size of active Au nanoparticles. The interest into NPG as a catalytic material was further enhanced by the first report on its potential as a highly selective partial oxidation catalyst as exemplified by the aerobic oxidation of methanol to methyl formate.^7^

NPG is prepared by dealloying, a process resulting in the spontaneous, self-organized creation of a nano- or microscale, bicontinuous network structure (continuous in both the material and the void space) from a previously homogeneous solid by selective removal of the less noble component.^8−11^ The pore sizes typically found in NPG (from about 10 to a few 100 nm) mostly fall in the range that is classified as mesopores comprising pore sizes of 2–50 nm according to IUPAC.^12^ Nonetheless, the term “nanoporous gold” is almost^13^ universally used for the material^6,7,9,14−17^ and sometimes extended even to other types of porous gold.^18−21^ Modern approaches to use dealloying as a material’s preparation method were pioneered by Sieradzki,^13^ and the class of nanoporous metals gained additional widespread interest by conceptual work of Erlebacher and co-workers,^9^ who not only linked the dealloying process to modern concepts of pattern formation in nonequilibrium processes but also effectively popularized the visual appeal of the resulting structures. One of the most attractive properties of nanoporous materials in general (and NPG in particular) is the outstanding definition, uniformity, and reproducibility of its nanoscale structure. Moreover, the possibility to *control* the characteristic pore and ligament sizes, the ability to modify the surface properties, and the lack of support effects render this material particularly well suited as a model system for understanding size and interface effects at the nanoscale in several fields, including mechanics,^8,14,22−27^ plasmonics,^28−31^ or (electro-)catalysis.^7,16,23,32−43^

Dealloying of AgAu alloys typically results in NPG, which predominantly consists of Au with only a small fraction (a few %) of the remaining less noble element (LNE; i.e., Ag in this case). As NPG is predominantly consisting of Au, it was suggested that its catalytic properties are largely based on the surface chemistry of gold.^7^ This notion was substantiated by comparing the oxidation chemistry on Au and Ag single-crystal surfaces studied under ultrahigh vacuum (UHV) by the Madix group^44−46^ with the aerobic catalytic oxidation on NPG.^7^ The insight that the oxidation chemistry of NPG is mainly due to Au^17,47^ was the basis for variety of experiments on Au single-crystal surfaces as well as theoretical studies. These studies have contributed significantly to the current microscopic understanding, including aspects such as the mechanism of oxidation reactions,^48,49^ the importance of the less noble element (LNE), mostly Ag, for oxidative catalysis,^50,51^ the activation of molecular oxygen,^52−54^ or the role of water.^55^ With respect to catalysis, it is particularly appealing that NPG can be used for gas phase,^56,57^ liquid phase,^58−60^ as well as electrocatalysis^61,62^ for the same reaction (e.g., partial oxidation of methanol), which allows elucidation of similarities and differences between the different scenarios.

A number of reviews are available on different aspects of NPG and its application, including structure evolution,^63^ mechanical properties^26^ and actuation,^64^ plasmonics,^31^ sensing,^65,66^ biomedical interfaces,^27,67,68^ energy conversion,^69^ or catalysis.^16,38,40,63,70,71^ We will review the recent developments with respect to oxidation catalysis, which aim at identifying decisive properties influencing catalytic performance and the ability to alter them by tuning these properties. As the catalytic properties are intimately linked to the structural and chemical properties of the materials, we set out by discussing preparation strategies for NPG, the measures of its nanostructure, and the development of these properties during catalysis. Detailed knowledge of the morphological properties is important for catalysis as mass transport of the reagents is an important aspect to be considered not only in liquid but also in gas phase catalysis. Hence, we will not only briefly introduce the conceptual framework (tortuosity) to describe diffusive transport in a nanoporous network, but will also discuss experimental approaches to quantify these aspects and show their impact on the catalytic performance. The microscopic understanding of the reaction mechanism in gas phase catalysis being a crucial ingredient to understand the catalytic properties of NPG is based on UHV experiments on single-crystal surfaces and to a large part on theory. Emphasis will be put on comparing the mechanistic pictures in gas phase catalysis evolving from the theoretical calculations with various experimental approaches on a system with systematically increasing complexity ranging from single-crystalline model surfaces to NPG. The catalysis at the gas/solid interface will be compared to results in liquid phase as well as electrocatalysis, furthermore highlighting current developments which afforded microscopic insight into the properties decisive for catalysis under these conditions.

## 2 Tuning of Structure and Composition during Dealloying

### 2.1 Nanoporous Gold by Dealloying

The notion of “dealloying” refers to a family of processes in which one chemical element is selectively removed from a solid solution or compound. The removal of this sacrificial element can exploit various mechanisms, for instance, evaporation at elevated temperature,^72,73^ dissolution in a molten metal,^11,74,75^ or corrosive attack in acid or aqueous electrolyte.^9,13,14,76^ An important commonality is that dealloying converts the uniform and massive (not porous) initial crystal into a nanoporous product crystal; the characteristic size of the pores represents a new length scale that is generated by nanoscale self-organization processes. Although experimental preparation protocols differ greatly among the dealloying variants, the atomic-scale processes that drive the nanostructure formation are quite similar.^11^ In each case, they rely on a competition between the active process of dissolution of the sacrificial element and a passivating process mediated by diffusive rearrangement of the conserved element.^77,78^

The present section focuses primarily on nanoporous gold (NPG) made by dealloying, emphasizing protocols for preparing macroscopic volumes of the material in clean and uniform quality (section 2.4). Why a focus on macroscopic volumes? First, it is a distinguishing feature of NPG that one and the same material provides—at the atomic scale—the catalytically active sites and—at the meso- or macroscale—the strong scaffold structure, which supplies and stabilizes the pore channels that bring the large area of surface and that enable access to its active sites. Second, macroscale samples of nanoporous gold have been the subject of intense studies with respect to their mechanical behavior. This behavior is not only important for the stability of the scaffold, it is also extremely sensitive to heterogeneity. Therefore, the excellent agreement of independent studies of mechanical behavior by several groups^26^ provides a signature of exceptional uniformity and reproducibility in NPG preparation. This distinguishes the underlying protocols as a unique basis for meaningful model studies of NPG in any field, and specifically including studies of catalysis.

For catalysis, the absence of an extrinsic scaffold material endows monolithic bulk samples of NPG with a unique conceptual simplicity. Yet, long transport pathways through a confined pore space can impair the catalytic conversion rates. This section also addresses four approaches to preparing samples that can mitigate this issue. First, monolithic NPG with a hierarchical pore structure (section 2.5) combines large pores for accelerated transport with small pores for high specific surface area and function. Second, cavity microelectrodes filled with μm-scale particles of powdered NPG (section 2.7) combine large open pathways (between the powder particles) with small active volume for enhanced kinetics and equilibration. Third, a similar effect is achieved with substrate-supported thin films of NPG (section 2.6), which have at least one external sample dimension in the range of 1 μm or below. Fourth and last, NPG nanoparticles (section 2.8) have an external size in the order of 100 nm or below in all three dimensions. Such particles can be employed in classic catalysis scheme, with particles attached to an extrinsic support scaffold, yet they profit from the enhanced specific surface area and activity of NPG.

### 2.2 Overview on Phenomenology and Processes during Dealloying

Preparation of the uniform, load bearing, monolithic macroscopic NPG that forms the focus of this section has been demonstrated with dealloying in aqueous media and near room temperature, in the form of either free or electrochemical corrosion. Here, bulk diffusion is exceptionally slow and can be neglected.^79^ The nanostructure formation is then simply the result of the two elementary processes, dissolution and surface diffusion. The related phenomenology has been investigated for more than a century in metallurgy, specifically in the contexts of alloy corrosion and stress corrosion cracking. The reader is referred to related review articles for details, and the following, incomplete list may serve as a starting point:^8,10,80−85^ The simplest concepts are that (i) the mole fraction *x*_LNE_ (also referd to as “atom fraction”) of the sacrificial element (here the less noble element, LNE) needs to exceed a minimum value, the parting limit, *x*_P_, and that (ii) the dealloying potential, *E*_D_, (i.e., the electrode potential applied during electrochemical dealloying) needs to exceed the critical dealloying potential, *E*_C_. The parting limit is related to the requirement for a percolating cluster of the LNE in the master alloy,^86^ and the critical potential is related to the requirement that the corrosion is driven sufficiently fast to proceed into the depth before the lateral rearrangement of the more noble element (MNE) by surface diffusion can form a closed layer of pure MNE that passivates the corrosion surface.^87,88^

**Figure 1:**
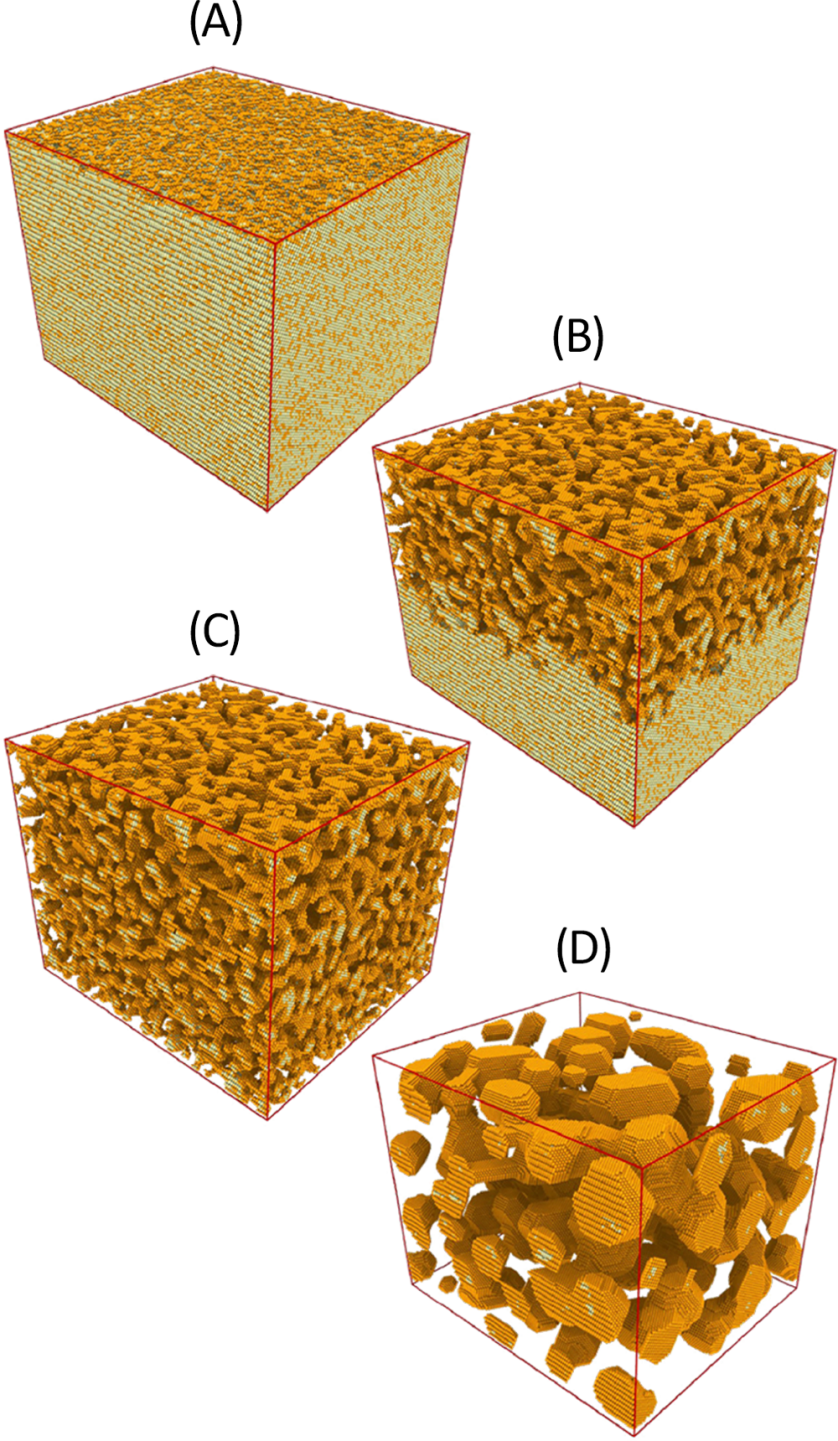
Porosity evolution during dealloying modeled by kinetic Monte Carlo simulation. Primary dealloying (A–C) starts with surface roughening and formation of passivated mounds (A). Small ligaments form by undercutting and the corrosion front proceeds inward, leaving a high concentration of less noble species in the ligaments’ interior (B,C). Secondary dealloying coarsens the structure and reduces the residual amount of less noble element (D). Reproduced with permission from ref (90). Copyright 2022 Elsevier.

The current understanding of the atomic-scale processes behind the above phenomena rests largely on kinetic Monte Carlo (KMC) simulation studies.^9,86−90^ Working with a minimal and physically motivated set of materials parameters, this approach reproduces many of the key experimental observations, thereby validating the underlying model. Early work suggested that a spinodal decomposition process in an adatom gas of the more noble element on the surface was responsible for the characteristic length scale of the nanoporosity.^9^ Yet, more detailed inspection of the KMC results identifies the nucleation of vacancy islands on crystal terraces, followed by dissolution from receding step edges, as a more appropriate scenario.^88^Figure 1A illustrates the roughened external surface in this early stage of dealloying. In principle, the just-mentioned processes link the characteristic ligament or pore size, *L*, to the magnitude of *E*_D_. Yet, this link has not been understood in all detail, and a predictive theory for *L* as the function of *E*_D_ is missing.^90^

The corrosive attack on the bulk is carried by the formation of pore channels which bifurcate on a characteristic scale that establishes the initial pore size (Figure 1B,C). That process of “primary dealloying”^91^ is followed by the coarsening of the porous structure by surface diffusion (Figure 1D). During coarsening, isolated domains (sometimes referred to as “clusters” because they represent percolating clusters of Ag atoms on the crystal lattice)^89,90^ of precursor alloy that had previously been buried in the interior of the primary dealloyed ligaments are exposed to the corrosive environment. “Secondary dealloying”^91^ then further dissolves the LNE, leading to an ongoing reduction in the residual LNE content. As an immediate consequence, the residual sacrificial element content systematically decreases with increasing, as-prepared *L*.^92^ A fraction of the buried Ag-rich domains survive in the as-prepared material and can be imaged with element-sensitive microscopy techniques.^89,93,94^ Ligament sizes, *L*, in as-prepared NPG thus reflect the initial, very small structure size during primary dealloying, convoluted with substantial coarsening during secondary dealloying.

The atomic-scale processes that act during dealloying do not, as a general rule, include the nucleation of new crystals. As will be addressed in section 3.6, dealloying can thus conserve the grain size, often several tens of μm, of the master alloy. In other words, dealloyed nanoporous metals can be single crystals when viewed at the scale of few ligament sizes. There is, however, a volume contraction that implies that the pore volume is typically smaller than the volume that was taken up, in the master alloy, by the dissolved LNE atoms. Section 3.7 addresses this issue, and it also points out that samples of NPG can exhibit a densified skin layer at their external surface, with the thickness comparable to the ligament size. The skin layer has little effect on the external dimensions or on the mean solid fraction of macroscopic samples, yet it contributes decisively to the densification of nanoporous nanoparticles.

Empirically, the structural evolution during dealloying can be, to some extent, controlled. To name a few, dissolution and interface diffusion rate are determined by temperature,^78,95,96^ electrolytic environment,^80,97^ alloy composition,^98−100^ and, in case of electrochemical dealloying, the dealloying potential.^9,101−104^ Furthermore, various postdealloying treatments have been demonstrated. They include the deposition of conformal oxide coatings along the pore surfaces by atomic layer deposition^105^ or of metal monolayers by underpotential deposition,^106,107^ and the functionalization of the pore surfaces by electroactive polymers such as polyaniline^108^ or polypyrrole^109,110^ or by organic molecules such as thiols,^111^ nucleic acids, or antibodies.^27^

An example for the control of the structural evolution during dealloying is the addition of Pt to the master alloy.^99,112−114^ Dealloying Ag–Au–Pt produces nanoporous Au–Pt with *L* as small as 4 nm, substantially less than the 20–40 nm that are typical for as-prepared NPG.^115−118^ The extremely small *L* in nanoporous Au–Pt has been attributed to the low surface diffusivity of Pt, which slows down microstructural coarsening. With attention to catalysis, it is relevant that Pt is strongly enriched at the surface of as-dealloyed nanoporous Au–Pt and that the Pt/Au atom fraction at the surface can be tuned in a wide interval by annealing.^119^

The trend of nanoporous metals to coarsen by surface diffusion underlies a simple and powerful postdealloying treatment. Enhancing the diffusivity, either by thermal or by electrochemical annealing, can be exploited for tuning *L* in an interval ranging from the as-prepared value, which can be as small as 4 nm, all the way up to several micrometers.^13,120,121^ The continuous tunability of its characteristic length scale by 3 orders of magnitude distinguishes NPG from practically all other nanomaterials.

A classic theory of microstructure evolution by diffusion predicts that *L* depends on time, *t*, as *L ∝ t*^1*/*4^ during coarsening.^122^ Atomistic numerical simulation confirms this prediction.^123^ Some experimental studies of NPG coarsening support the *t*^1*/*4^ law,^124,125^ while others report large confidence limits for the exponent.^95,126−128^ A data mining survey of the literature favors *t*^1*/*8^ (emphasizing that this has no support from theory), yet ultimately qualifies the available data as inconclusive.^129^ If the power law is accepted, then the ramifications of its low exponent merit a closer look. The exponent 1/4 implies that the time increment for a given relative increase in ligament size varies as *L*^4^. In other words, local regions in the high-*L* tail of the size distribution coarsen more slowly than the remaining microstructure, allowing the smaller ligaments to catch up. The scenario is similar to the diffusion-controlled Ostwald ripening of precipitates. That process exhibits a similarly low time exponent (1/3 as opposed to the present 1/4) and, by virtue to the Lifshitz–Slyozov–Wagner (LSW) theory, a quasi-stationary size distribution even after extended coarsening.^130,131^ Quite analogously, NPG typically maintains a highly uniform microstructure, even after extended coarsening (see also section 3.9). When NPG coarsens outside the corrosive environment, then residual LNE exposed to the surface is not dissolved. The substantial relocation of atoms will then also affect the distribution of LNE within the ligaments. Their composition field may be homogenized, or an LNE-enriched layer at the surface may be formed. See section 3.13 for details.

Control over the dealloying process parameters is a prerequisite for the successful design of preparation protocols for nanoporous metals. This will be discussed in the following section.

### 2.3 Approaches to Dealloying

In principle, NPG can be prepared in every shape and dimension, as long as the precursor alloy and a surrounding electrolyte share a common interface. Geometries range from nanoscale particles,^132^ wires,^133^ films,^134−136^ and leaves^80,102,137−139^ to μm-thick coatings,^140^ pillars,^141,142^ and sheets,^143−146^ up to mm-^100,116,118,147−150^ or cm-size^151^ bulk samples. The making of even larger parts from NPG, for instance in the form of macroscale scaffolds for catalysis, has been demonstrated based on 3D printing.^152^

Precursor alloys need to meet certain requirements to ensure successful dealloying. The corrosion potentials between the noble and less-noble species must differ by typically a few 100 mV. Also, the master alloy should be a single phase of uniform composition, either in the form of a solid solution, a glass, or an intermetallic compound. Both criteria can be readily met by various master alloys for NPG. Specifically, preparation protocols for NPG have been based on alloys containing Ag,^13,76,92^ Cu,^153−157^ Ni,^100^ Al,^158,159^ Sn,^135^ Zn,^140,160,161^ Li,^162^ and multicomponent glasses.^163−165^ However, in macroscopic dimensions, i.e., for at least mm-sized samples, preparation of NPG has so far been limited to alloys prepared by classical metallurgical methods such as Au–Cu, Au–Ni, and, as the by far most commonly used alloy system, Au–Ag.^13,118,148−150^ The parting limit for Au–Ag is 45 at. % Au.^10,86,166^ Monolithic mm-size samples of NPG have been prepared from master alloys with gold atom fractions between 5 at. %^167^ and 42 at. %.^168^

Dealloying can be prompted by free^80,92,118,139,169−172^ or by electrochemically^9,13,76,92,103,118,150,173,174^ controlled corrosion. Electrolytes include aqueous solutions across the whole pH range^13,92,96,135,146,174^ as well as ionic liquids.^140,160,175^ However, the majority of preparation protocols for NPG use acidic environments. For Au–Ag master alloys, free corrosion is generally carried out by concentrated HNO_3_,^80,92,118,139,169−172^ while electrochemical dealloying typically uses dilute HClO_4_ solutions.^9,13,76,92,118,150^ Both free and electrochemical dealloying can produce large monolithic volumes of NPG with comparable microstructure.^176^ For samples with identical *L* and master alloy composition, similar mechanical properties are observed irrespective of which corrosion method is used.^118,176^ This suggests comparable sample quality and comparable microstructure. Yet, electrochemical dealloying offers a more direct control over the corrosion process. For example, variation of *E*_D_ is a facile method to control the final *L*. Here, higher *E*_D_ result in smaller *L*.^102,103^

Dealloying does not dissolve the LNE entirely. The residual content of LNE can vary between *<*1 at. %^148^ and up to 80 at. %.^177^ The retained amount of LNE is crucial for the preparation of hierarchical nanoporous structures by sequential dealloying.^167,177^ This will be explained in section 2.5. More importantly, the LNE content may be decisive for the catalytic performance of NPG. This will be inspected in sections 4–6. Here again, the electrochemical (as opposed to free) corrosion affords tuning the preparation protocol toward a direct control of the residual LNE content. The next section will elaborate in more detail on preparation protocols.

### 2.4 Preparation Protocols for Nanoporous Gold: A Case Study

#### 2.4.1 Motivation

Among the many preparation protocols for NPG, few can produce the macroscale, uniform, crack-free, monolithic bodies with exceptionally clean surfaces that are the signature of high-quality NPG. The present section attempts a critical discussion of relevant process parameters for preparing NPG, and it also proposes a “best practice” guide for that material. The protocol is based on an approach developed in the teams of Jin and of Weissmüller more than a decade ago^178−180^ and refined subsequently in both teams.^116−118,147,148,150^ Results consistently show that macroscopic samples with a homogeneous microstructure can be obtained that are free of macro-defects such as cracks, show little dealloying-related shrinkage, and exhibit a low residual LNE content.^116−118,148,150^ The consistency of the mechanical behavior of samples prepared by the protocol^116,118,148,150^ testifies to an exceptional reproducibility. This is major asset and a motivation for describing that particular protocol here.

#### 2.4.2 Master Alloy Selection and Preparation

In this case study, the master alloy is AuAg, the most commonly used precursor system in the preparation of NPG. The main advantage of AuAg is the continuous series of solid solutions in the equilibrium alloy phase diagram, in other words, full miscibility at any composition and at all temperatures up to the solidus.^181^ By contrast, AuCu and AuNi, the other binary alloy systems used for the preparation of macroscopic NPG, exhibit multiple ordered superstructure phases (AuCu)^182^ or a miscibility gap (AuNi)^183^ at room temperature. In order to avoid phase separation, careful heat treatment is required during preparation of AuCu and AuNi precursor alloys.^100,154,156^ Even when the master alloy is prepared in the form of a uniform solid solution, AuCu-based NPG has been found to exhibit poor mechanical properties in the form of brittle deformation behavior. This is the result of microstructural heterogeneity, and specifically of crack formation along grain boundaries that were weakened during dealloying.^154^ While macroscopic bodies of NPG dealloyed from AuNi show high strength and ductility, the ligament size tends to be large, *L ≈* 120 nm.^100^ Because many application scenarios proposed for NPG require both uniform microstructure and small *L*, AuAg is often the master alloy system of choice for making of NPG.

**Figure 2:**
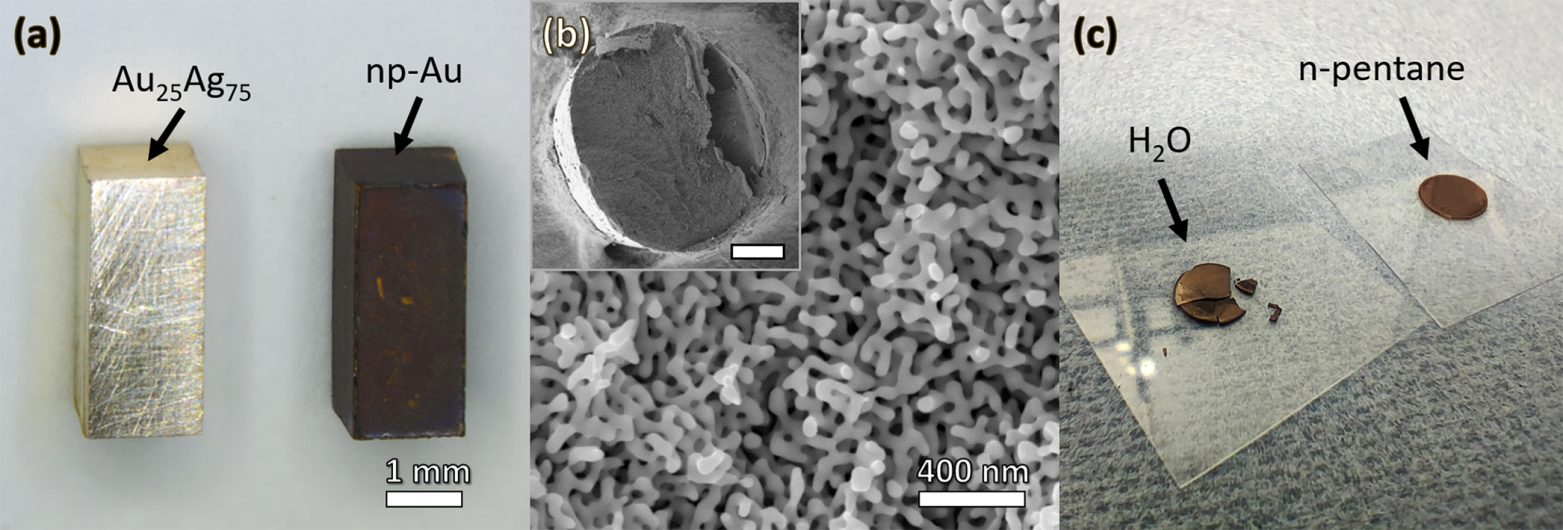
Preparation of nanoporous gold (NPG) by electrochemical dealloying. (a) Au_25_Ag_75_ precursor (left) and as-dealloyed NPG sample (right). (b) Microstructure of NPG imaged by scanning electron microscopy. Inset shows the cross section of a sample that was intentionally cleaved using a scalpel; the scale bar corresponds to 300 μm. (c) Nanoporous gold disks air-dried after preparation. The samples were wetted by H_2_O (left) and *n*-pentane (right) before drying. Note, capillary-induced stresses cause bending and breaking of samples wetted by H_2_O which can be avoided by a final transfer to *n*-pentane. (a,b) Reproduced with permission from ref (192). Copyright 2020 The Author.

The present example uses master alloys Au_25_Ag_75_, prepared from Au and Ag wires (≥99.99% purity) by arc melting. The ingot is then encapsulated in evacuated fused silica and homogenized by annealing for 5 days at 850 °C. Afterward, numerous conventional metal shaping techniques, such as rolling, drawing, and cutting can be applied to prepare the precursor samples; an example of a cuboid sample before and after dealloying is shown in Figure 2a.

It is emphasized that a homogeneous precursor microstructure is important for obtaining high-quality nanoporous samples. Therefore, a final recovery anneal may be required to annihilate lattice dislocations and relax residual stress that result from the final machining. For AuAg alloys, an annealing temperature of 300 °C has been found suitable for recovery.

#### 2.4.3 Potentiostatic or Galvanostatic Dealloying

The present protocol uses electrochemically controlled dealloying under conditions of constant applied potential, in other words, potentiostatic dealloying. In rare cases, galvanostatic control (constant net current, *I*) is used during electrochemical dealloying.^184,185^ This approach results, in principle, in a constant dissolution rate of the less noble species. That may seem an appealing concept for uniform dealloying. However, it may be argued that constant *I* does explicitly not lead to uniform dealloying conditions. This is so because the active surface area, *A*, as well as the alloy composition, vary as the dealloying procedes. In the early stages of dealloying, *A* of the smooth surfaces of the pristine master alloy sample is small. Therefore, the dissolution current density, *j* = *I*/*A*, is initially high. As soon as the rough corrosion front of primary dealloying is formed, *A* increases significantly and this decreases *j*. With progressing dealloying, dissolution by secondary dealloying becomes more important, as ions not only dissolve at the dealloying front but also in the trailing volume. It can be seen that secondary dealloying introduces a time dependence to the magnitude of *A* and thereby to *j*. Moreover, as soon as primary dealloying is complete and residual species are solely dissolved by secondary dealloying, the corrosion acts on a surface that is effectively quite dilute in LNE. In order to maintain the required high dissolution current, the dealloying potential then needs to be controlled to very positive values. That changes the oxidation state of the surface, and it may move the dissolution process into a different regime of the Pourbaix diagram. Clearly, those features of galvanostatic dealloying are not compatible with constant and uniform corrosion conditions. The effect of specific adsorbates on the dealloying process is discussed later in this section.

The by far most common electrochemical dealloying procedure is potentiostatic dealloying. Regarding the dealloying process, a constant *E*_D_ corresponds to an invariant driving force for the LNE dissolution. As the elemental composition changes during the dealloying process, a static potential causes a gradual reduction in *j*. Although dissolution conditions vary during both electrochemical preparation methods, potentiostatic dealloying can be considered the more uniform preparation technique because every volume element is subjected to the same dissolution progression irrespective of its location within the sample. In this case study, potentiostatically controlled dealloying is used. A three-electrode configuration is mandatory, not the least because the dissolved LNE, a large amount of matter, is deposited on the counter electrode.

#### 2.4.4 Choice of Electrolyte

As with most of the above-mentioned electrochemical dealloying protocols, the present case study uses dilute, namely 1 mol L^–1^, HClO_4_ as the electrolyte. In this environment, the surface diffusivity of Au atoms is rather slow. This results in a low coarsening rate during secondary dealloying and thereby reduces the final *L*.^97^ Additions of halide-ions (Cl^–^, Br^–^, and I^–^) have been reported to significantly increase the Au surface diffusion rate of HClO_4_-based electrolytes.^97^ This may shorten the overall dealloying time, yet at the expense of considerably larger *L*.

While HClO_4_ is unsuspicious of specific adsorption on gold,^186^ adsorbates formed by impurities may influence the dealloying process and the catalytic performance of the nanoporous material. For example, during the long period of dealloying Cl^–^ impurities may enter the electrolyte when reference electrodes with a KCl environment are used, such as Ag/AgCl or calomel. In this case study, the issue is avoided by usage of a pseudoreference electrode in the same solution, namely a AgCl-coated Ag wire (+0.52 V (SHE) in 1 mol L^–1^ HClO_4_). This minimizes contamination, due to the extremely low solubility of AgCl. It is good practice to calibrate pseudoreference electrodes against more stable ones, such as the standard hydrogen electrode (SHE), before each dealloying run. When calibrated, other pseudo reference electrodes may also be appropriate, such as the reversible hydrogen electrodes (RHE). In order to minimize impurities, ultrapure water (≥18.2 MΩ cm) is used to prepare all aqueous solutions.

It should be noted that dealloying in electrolytes that are, in pristine condition, devoid of ions of the less noble species exhibits poorly defined starting conditions because the Nernst potential is very low or ill-defined at the onset of corrosion. This issue settles once ions dissolve into the electrolyte. Still, as the dealloying critical potential depends on the Ag^+^ concentration in solution, the dealloying conditions in the just-mentioned media may vary with the electrolyte volume and with the ion transport kinetics.^101^ An approach that circumvents this issue is dealloying of Au–Ag in AgNO_3_ solutions.^146,174^ However, due to the formation of passivating AgO layers, the residual Ag mole fraction, *x*_Ag_^res^, in as-dealloyed NPG was found to be very high (*x*_Ag_^res^ > 30%).^146^ Addition of AgClO_4_ into HClO_4_ solution is another strategy to obtain well-defined dealloying conditions.^101^ Although this dealloying approach has been reported two decades ago,^101^ systematic addition of AgClO_4_ into electrolytes for the purpose of making of NPG under even better controlled conditions has not been implemented so far.

#### 2.4.5 Dealloying Potential and Postdealloying Conditioning

The dealloying critical potential of Au–Ag exhibits comparatively high values in pure HClO_4_-based electrolytes.^98^ The present protocol features potentiostatic dealloying of Au_25_Ag_75_ precursors at *E*_D_ = 1.27 V (SHE), i.e., 0.75 V (pseudo Ag/AgCl). Dealloying is considered as complete when the current decays to a few tens of μA. For cylindrical Au_25_Ag_75_ precursors with a diameter of 1 mm and a length of 2 mm a value of 10 μA is used. As a result of the quite positive value of *E*_D_, oxygen species adsorb on the Au surface.^187^ The adsorption impairs surface diffusion and slows down the dealloying rate. In alloy systems with a less noble sacrificial element, such as Au–Ni, oxidation of the Au surface can be mitigated by dealloying at lower *E*.^100^ Oxygen adsorption on NPG also embrittles the material, as lattice dislocation end points are pinned at adsorbate sites.^188^ While oxygen species adsorption on Au thus seems unfavorable, it can be used to hinder coarsening at ambient conditions. Thus, deliberate oxidation may enhance the shelflife of stored samples of NPG.

After dealloying by the steps described so far, samples of NPG exhibit *L ≈* 20 nm^115^ and a high *x*_Ag_^res^, around 20 at. %.^116^ The next step in the preparation protocol is designed to reduce *x*_Ag_^res^ further. To this end, the sample is first polarized at *E* = 1.37 V (SHE) until the current diminishes. Afterward, the electrolyte (1 mol L^–1^ HClO_4_) is renewed and 20 potential cycles (0.12 to 1.62 V vs SHE) are applied at a scan rate of 5 mV s^–1^. The two steps, polarization and potential cycles, are then repeated once. In each case, the potential cycling is concluded by an anodic scan to the electrode potential 0.82 V (SHE). This corresponds to an adsorbate-free Au surface state.^189^ This preparation routine results in clean and reduced metallic Au surfaces. Note also that the dealloying and conditioning do not involve organic solvents. This is one more distinctive feature of dealloying that affords exceptionally clean surfaces.

The postdealloying treatment procedure reduces the overall Ag content, as determined by energy dispersive X-ray analysis, to values below *x*_Ag_^res^ < 1%. X-ray photoelectron spectroscopy (XPS) suggests that the remaining Ag preferably accumulates at the surface.^34,190,191^ With the present protocol, the XPS (near-surface) Ag fraction was determined at *x*_Ag_^res^ = 8%.^191^ Final ligament diameters, as determined by scanning electron microscopy, are around 40 nm.^116,117,192^

#### 2.4.6 Drying

Drying is an often a neglected topic in the preparation of nanoporous metals. Throughout the dealloying and conditioning, the samples are wetted by electrolyte. Upon drying, the surface tension of the receding liquid–vapor meniscus, γ_LV_, introduces a pressure, *p*, in the pore fluid according to the Young–Laplace equation, namely
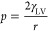
1Here *r* denotes the radius of the meniscus, approximately equal to the pore radius.^193^ Given that the pore size of NPG is only a few tens of nm, *p* takes on values of around 10 MPa for water (γ_LV_ = 72 mJ*/*m^2^)^193^ in the pore space. However, significantly higher values of *p*, exceeding the yield strength, can ensue at local constrictions. As a result, improper drying procedures can cause severe deformation or spontaneous fracture of nanoporous metals, as demonstrated in Figure 2c. Although this issue has been well-known for many decades, for example, in the fields of porous semiconductors^193−195^ or silica aerogels,^196^ it is hardly appreciated in the context of nanoporous metal preparation. The drying technique below, adopted from protocols for mesoporous silicon,^193^ has proved useful in the making of nanoporous metals.^197,198^

An efficient way to minimize capillary-induced stresses is to transfer the nanoporous metals from the preparation medium first into water, then into ethanol and finally into *n*-pentane, whereupon the samples are deposited on glass and allowed to dry. This procedure profits from the considerably lower surface tension of *n*-pentane, which is γ_LV_ = 14 mJ*/*m^2^ as opposed to the 72 mJ*/*m^2^ of water. The contrasting juxtaposition of NPG sheets displayed in Figure 2c clearly emphasizes the need for consideration of capillary-induced stresses when crack-free monolithic bodies of NPG are required. A drawback of the drying routine is its use of organic solvents that may alter the surface state of the porous metal. Therefore, drying in *n*-pentane is not established as a standard preparation step in the presented case study, and application should be made dependent on need. Other, more laborious techniques such as freeze- and supercritical drying avoid the organics but appear not to have been applied to nanoporous metal so far.

### 2.5 Hierarchical Nanoporous Gold

**Figure 3:**
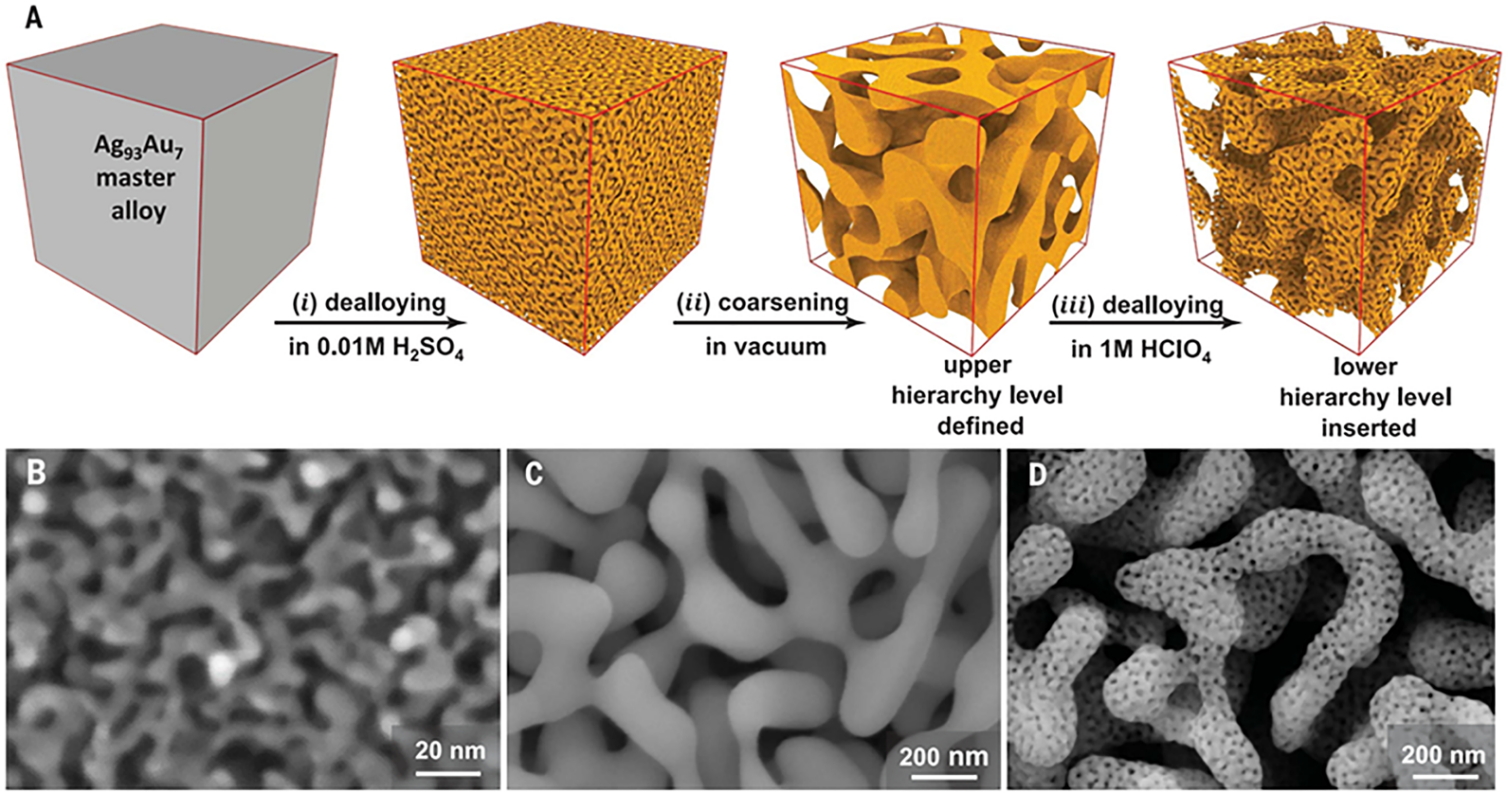
Making of hierarchical nanoporous gold by dealloying. (A) Schematic illustration of the preparation steps: (i) first dealloying of a Ag-rich precursor alloy leaves a nanoporous network with high amount of residual Ag; (ii) coarsening of the ligament structure by thermal annealing; (iii) second dealloying forms the lower level nanoporous microstructure. (B–D) Scanning electron micrographs of the microstructure after the first dealloying (B), coarsening (C), and second dealloying steps (D). Reproduced with permission from ref (177). Copyright 2021 American Association for the Advancement of Science.

So far, this section 2 addressed the formation mechanisms behind the evolution of the lowest level microstructure required to form bodies of NPG. Most of the unique properties of NPG can be directly attributed to its small ligament size. Yet, the small size may also have adverse effects, specifically in slowing down mass transport through the pore space. This is of obvious relevance for the performance in catalysis. Structuring on multiple length scales may solve the dilemma by admitting both small pores for achieving a high specific surface area and large pores for transport. Nanoporous metals with multiple microstructural length scales have been the subject of considerable interest in recent years.^199−201^ The lowest structural level of multimodal NPG is typically made by dealloying, and numerous approaches toward the higher structural levels have been proposed, such as templating,^202,203^ additive manufacturing,^152^ and selective dissolution of multiphase alloys.^158^ A particularly stringent implementation of multileveled pore structures is hierarchical porous materials in which the structural organization at each of the individual hierarchy levels is geometrically similar, with identical geometry and topology at each level but distinctly different characteristic length scales. For the specific structural motive of NPG, networks nested on two separate hierarchy levels have been demonstrated and exemplify the stringent hierarchical organization.^167,177,204−207^ The microstructure of ”nested-network nanoporous gold”^167^ is illustrated in Figure 3. The straightforward architecture of such materials predestines them as model systems for understanding the impact of hierarchical structuring on properties and for the rational design on multiple microstructure levels.^177^ Below, we briefly indicate how the preparation protocol of the previous sections can be expanded to yield nested-network nanoporous gold with two hierarchy levels. The making of porous metals with other, less stringent multiscale geometries is described in several recent reviews.^199−201^

Figure 3A schematically illustrates the preparation of nested-network NPG by dealloying, as first reported in ref (167) and recently refined for robust macroscopic samples.^177^ While minor preparation details may vary in other studies,^205,207^Figure 3 captures the essentials. The master alloy is dilute in Au, e.g., Au_7_Ag_93_, and the dealloying conditions are carefully selected so that *x*_Ag_^res^ remains very high, affording a later, second dealloying step. The resulting, nanoporous Ag–Au (with, for instance, *x*_Ag_^res^ = 76%)^177^ is then coarsened by annealing, which establishes the microstructure of the upper hierarchy level. The second dealloying then introduces porosity into the ligaments of that upper level, establishing the lower hierarchy level. The anticipated enhanced transport rate in nested-network nanoporous gold is indeed confirmed by experiment.^167,207^ Furthermore, the range of solid fractions accessible to dealloying is extended downward to as low as 0.12 while mass-specific mechanical strength and stiffness are superior to NPG with a single hierarchy level.^177^

### 2.6 Engineering the Macroscopic Shape of Nanoporous Gold

#### 2.6.1 Machining the Master Alloy Preform

**Figure 4:**
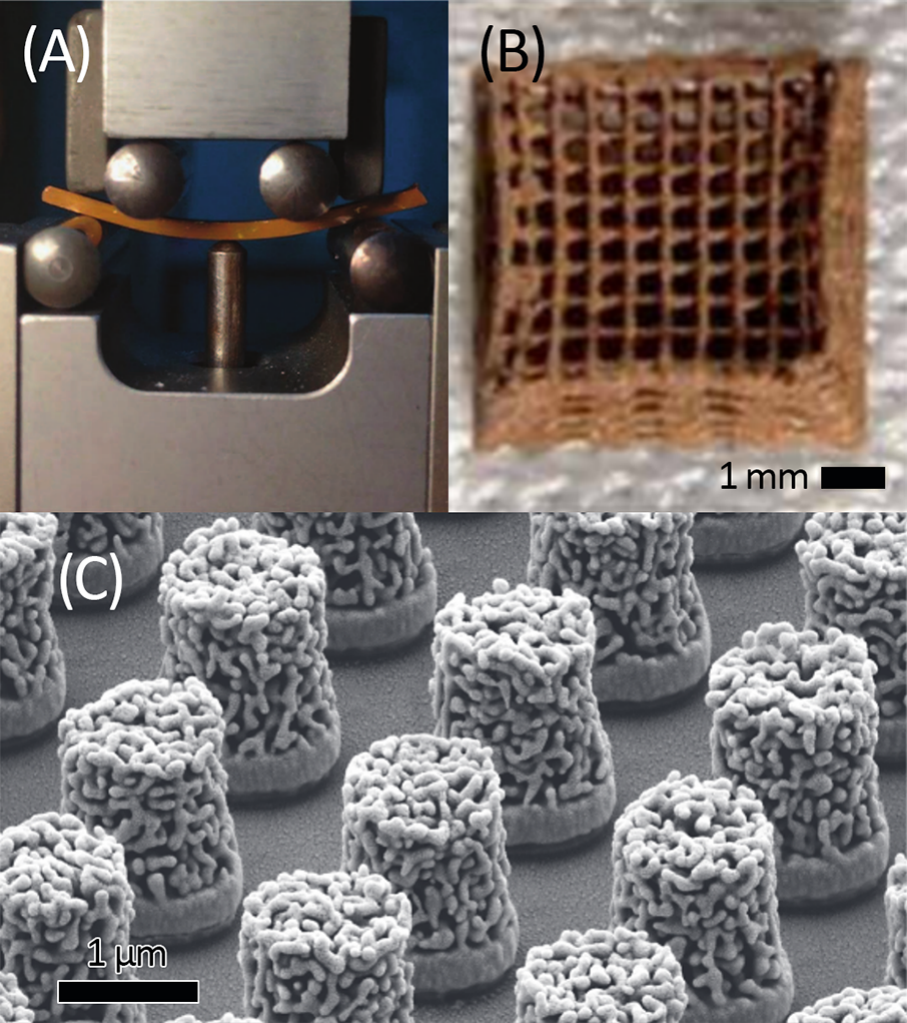
Shaping samples of NPG. (A) A 1 mm × 2 mm × 26 mm size rectangular bar of epoxy–resin infiltrated NPG (center, brownish color), here subject to a four-point bending test. (B) 3D-printed, cm-size lattice of NPG. (C) NPG bumps with diameter of 1.0 μm on planarized CMOS chip. (A) Reproduced in part from ref (151) under Creative Commons license CC BY 4.0. Copyright 2015 The Authors. (B) Reproduced in part with permission from ref (152). Copyright 2018 The Authors. (C) Reproduced with permission from ref (220). Copyright 2022 IEEE.

A wide variety of conventional metal forming approaches can be applied to master alloys for macroscopic samples of NPG. These include, for instance, cutting, milling, rolling, or wiredrawing. In principle, dealloying is a shape-conserving process. Size and shape of NPG samples may therefore be selected by appropriate machining of the master alloy. Yet, care has to be taken to choose the conditions for minimizing sample shrinkage during dealloying. Section 3.7 discusses phenomenology and mechanisms of shrinkage. Figure 4A shows a cm-size rectangular beam of (epoxy–resin infiltrated) NPG as an example for a machined macroscale geometry.

#### 2.6.2 3D Printing the Master Alloy

Recently, it has been demonstrated that NPG catalyst samples with complex microscopic geometries can be produced by 3D printing. The process acts on an ink containing master alloy particles, which are dealloyed subsequent to printing the preform (Figure 4B).^152^

#### 2.6.3 Nanoporous Thin Layers and Nanoporous Nanowires

Monolithic dealloyed bodies of NPG, of extended size in all three dimensions, are often not very well suited for the integration in reactors. That is a consequence of the inefficient mass transport in the pore space, in conflict with the need for mitigation of diffusive and resistive gradients in the reactor. Many studies have solved that problem by working with thin layers of NPG. These can be made by dealloying the free-standing thin (typically around 100 nm) master alloy sheets of Ag–Au that are commercially available as white gold leaf.^80^ Thin films based on vapor-deposited master alloys on planar substrates are also frequently explored.^27^ Alternatively, thin layers of NPG can be conformally deposited on current conductive support structures by galvanic deposition of an alloy that is subsequently dealloyed to NPG. Such processes can exploit the advantages of an all-wet-chemical processing of electrodes of almost any shape and size.^208^

Layers of NPG have been studied as cantilever actuators^209,210^ and sensors.^211^ Sensors with optical or electrical readout have been proposed for biomedical applications,^27^ substrates for surface-enhanced Raman scattering,^212^ studies of optical properties,^28,213,214^ energy-storage,^215^ electrocatalysis,^216^ and photoenhanced catalysis.^29,217^

Substrate-supported thin layers of master alloy for NPG can be structured by electron-beam lithography and subsequently dealloyed to produce thin structures of NPG with a wide range of 2D shapes. That is of interest because lithographic structuring may, in principle, be seamlessly integrated into microtechnology manufacturing schemes. Wire- and disk-shaped samples have been demonstrated,^218−220^ and an example is shown in Figure 4C.^220^

Nanoporous nanowires can also be made as freestanding objects. Several works have demonstrated that the master alloy can be electrodeposited into suitable nanoscale channels in a substrate, for instance, anodized alumina^221,222^ or ion-track-etched channels in a polymer.^223^ Removing the substrate and subsequently dealloying generates the nanowires. In a less controlled way, dealloying columnar microstructures in rapidly solidified metal ribbons may also yield rod-like nanoporous particles.^224^

**Figure 5:**
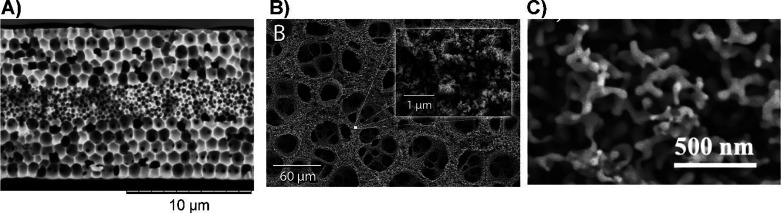
Preparation of porous gold by processes different than dealloying. (A) anodization in HCl solution; (B) H_2_ bubble templated deposition; (C) templated electrodeposition between sacrificial nanoparticles. (A) Reproduced with permission from ref (20). Copyright 2020 Elsevier. (B) Reproduced with permission from ref (252). Copyright 2014 Elsevier. (C) Reproduced with permission from ref (233). Copyright 2012 Wiley-VCH.

Lastly, thin layers of nanoporous gold can be obtained by electrochemically roughening an initially flat Au electrode. A typical procedures involved anodization, followed by a reduction of the created oxide layers.^20,225−229^ The resulting morphology is similar to that of NPG obtained by dealloying (Figure 5A). The thickness of such layers of NPG may be restricted to few tens of nanometers. Roughening of initially flat Au electrodes has also been demonstrated by electrodepositon/alloying/dealloying cycles,^140,160,230−232^ often using Zn^140,160,230^ or Ag^232^ as the LNE. These can be applied to electrodes of nearly any size and shape, even when preassembled in an electrochemical cell of specific geometries such as microelectrodes for sensors.

### 2.7 Nanoporous Powders

#### 2.7.1 Making Powder from Bulk NPG

Electrodes for electrochemical applications have to be integrated with other electrodes into an electrochemical cell optimized for the specific application, such as a parallel plate reactor. The direct integration of NPG has only rarely been demonstrated in devices such as fuel cell^234^ or metal–air batteries.^235,236^ Nanoporous gold has also been used for alloy-type negative electrodes in Li-ion batteries.^237^ However, the overall accounts of such application has remained limited to date.

**Figure 6:**
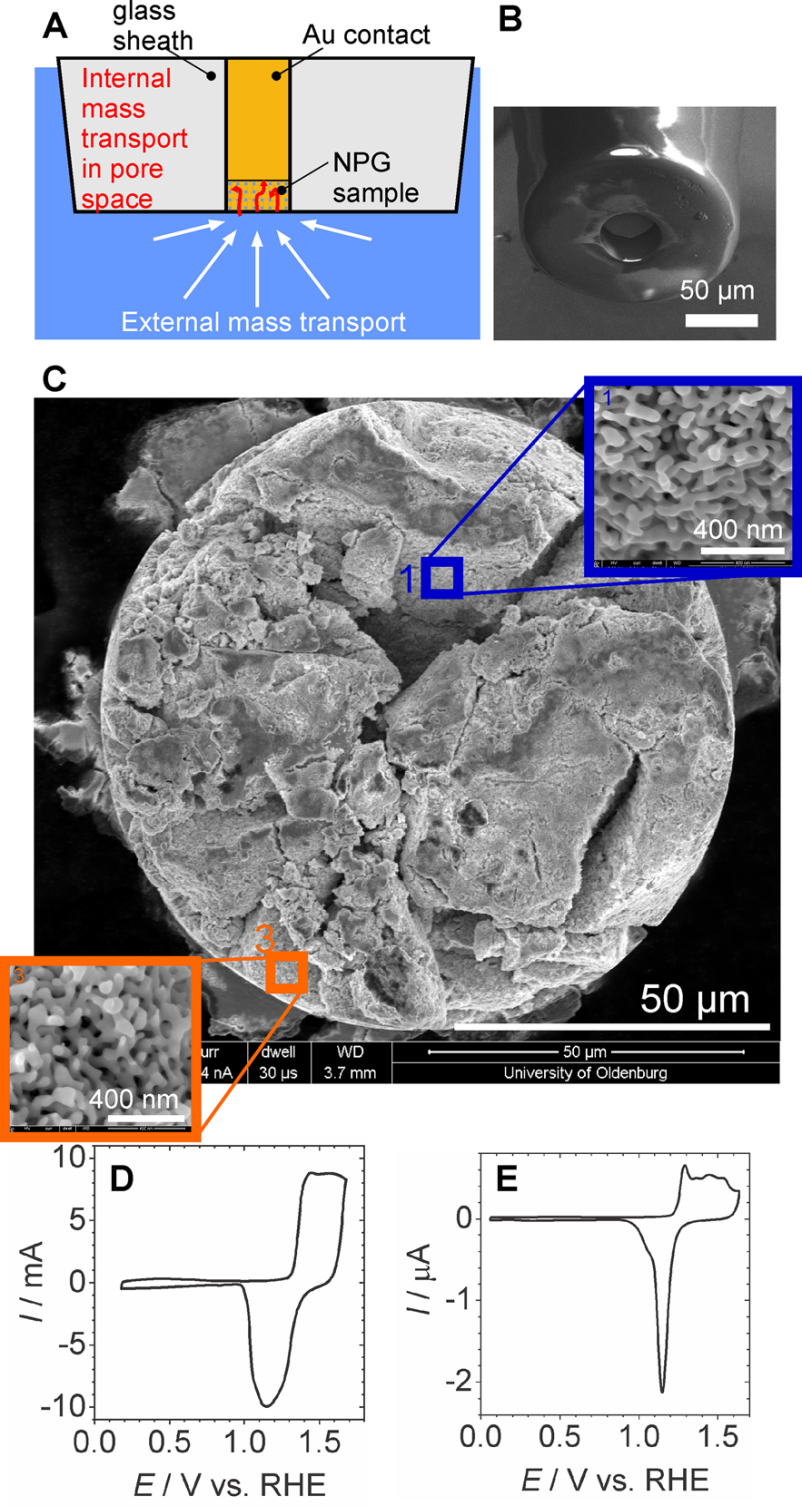
Cavity microelectrodes. (A) Schematic cross section; (B) SEM image of empty cavity microelectrode produced by etching a glass-sealed gold wire; (C) SEM image of a cavity microelectrodes filled with NPG powder, insets show zoomed areas with NPG ligaments; (D) cyclic voltammogram of a *macroscopic* NPG monolith in 1 mol L^–1^ HClO_4_, potential scan rate *v* = 10 mV s^–1^; (E) cyclic voltammogram of the very same NPG material inside a cavity microelectrode in 1 mol L^–1^ HClO_4_, *v* = 10 mV s^–1^. (B) Reproduced with permission from ref (238). Copyright 2019 Wiley-VCH. (C) Adapted from ref (191) under Creative Commons license CC 4.0. Copyright 2020 The Authors. (D,E) Reproduced with permission from ref (638). Copyright 2022 The Author.

In principle, powders obtained from NPG monoliths offer another pathway to shaping samples of NPG because they can be consolidated into appropriate shape. Cavity microelectrodes of NPG form an example, providing for particularly precise electrochemical characterization. Due to the limited material, the overall currents are small and thus the voltage drop *U*_drop_ = *IR*_e_ (“*IR* drop”) is kept small. Voltammograms recorded with cavity microelectrodes have particularly little distortion, cf., Figure 6E vs D. Furthermore, the external mass transport (outside of the pore space) is controlled by the effective radial diffusion (Figure 6A). In addition, the exchange of the solution in the pore space is efficient.

NPG monoliths can be powdered by mechanical impact, sonication, or similar processes. The powder can be filled in a cavity microelectrode (Figure 6A), while preserving the nanoscale structure, without the use of binder (Figure 6C).^191^ NPG can be released from cavity microelectrodes by intensive sonification, after which the same cavity may be washed and filled by another aliquote of the same NPG powder (Figure 6B). Using powders from identical starting material tends to decreases data scatter in electrochemical experiments with NPG by avoiding small variation between monoliths in the dealloying process.^107^

#### 2.7.2 Processing NPG Powders

Instead of upscaling the size of the original NPG electrode, pulverized monoliths can be processed with binders to obtain porous electrodes coated on a back contact. The use of binders and the associated problems in reliably establishing electrical contact between particles removes many of the original advantages associated with the bicontinuous structure of NPG. Nevertheless, such processes may be required for the integration of NPG into devices.^239−241^

### 2.8 Dealloyed Porous Nanoparticles

Use of a dealloying process is increasingly extending from macroscopic materials to nanomaterials. Dealloying of binary and ternary alloy nanoparticles has become a broadly accepted facile synthetic route toward highly active and durable catalyst materials for heterogeneous and electrochemical reactions.^42,63,79,242^ The focus of this section is on the relation of nanosizing of binary alloy nanoparticles on the (electro)chemical dealloying and their comparison to bulk materials.

Dealloying of multimetallic nanoparticles covers a broad range of particle motifs like core–shell, hollow, donut-like to porous nanoparticles to tune their physicochemical and (electro)catalytic properties. In contrast to the macroscopic dealloyed materials, the fundamental dealloying mechanisms behind the formation of size-dependent particle morphologies are still poorly understood to date.

The critical dealloying potential is largely influenced by the initial composition of the master alloys. It is well studied that smaller monometallic nanoparticles tend to dissolve at lower anodic potentials compared to the bulk materials; this tendency is more pronounced with decreasing particle size.^243^ This shift of the dissolution potential as the function of the particle size can be related to the Gibbs–Thomson effect. With decreasing size, the fraction of low-coordinated surface atoms rises, resulting in an increase of the surface energy of these nanoparticles.

Recent KMC simulations have indicated how the size-dependent electrochemical properties of monometallic nanoparticles can be related to the critical dealloying potential of alloy nanoparticles.^244^ One of the key results is that the evolution of porosity in alloy nanoparticles takes place at higher critical dealloying potentials than in the corresponding bulk alloys. The increase of the critical potential in relationship to 1/*r*_NP_, where *r*_NP_ is the radius of the alloy nanoparticle is based on kinetic effects. Compared to bulk Ag_75_Au_25_, the KMC simulations predicted an increase in critical dealloying potential by 25 mV for 17 nm and by 100 mV for 8 nm nanoparticles.^244^ As has been explained in section 2.2, the relative rates of (1) the dissolution rate of the LNE and (2) the passivation of the surface by diffusion of the remaining MNE are decisive for the nanostructure formation during dealloying. The ratio between these rates may be significantly modified in nanoparticles. According to these KMC simulations,^244^ the following time-dependent characteristics have been identified: In the initial stage of the dealloying process, Ag atoms at the top layer of the nanoparticles are dissolved very rapidly. During the intermediate time, the particle surface is entirely passivated by the very mobile Au surface atoms. The surface roughening induced by dissolution of Ag surface atoms enhances the formation of a monolayer of Au atoms as a passivation layer. Therefore, nanoparticles tend to form a passivation layer of MNE atoms very quickly. Only over longer times and at a constant potential, the fluctuations in the passivation layer might be sufficient for the dissolution of the Ag atoms from the subsurface layers. Smaller alloy nanoparticles with a high content of low-coordinated surface atoms and short-lived fluctuations in the structure of passivation layer do not tend to evolve pores. In contrast, the pore evolution is more favored if the fluctuations are long-lived and allow successive Ag dissolution, which is the case for larger particles.

Only few experimental studies have investigated the particle size effect on the morphology of dealloyed nanoparticles. One of the first systematic studies pointed out the particle size effect for electrochemical dealloying on binary alloy nanoparticles.^242^ In case of Ag–Au, the particle size effect on the morphology of 2–6 nm and 20–55 nm alloy has been studied by applying different constant potentials in acidic media and characterized by high-resolution microscopic techniques.^79^ In agreement with the reported KMC simulations,^244^ a superficial dealloying occurs at potentials above the critical potential of 2–6 nm AgAu nanoparticles. Due to the inappropriate ratio of surface atoms to bulk atoms, the pore evolution is not favored for small nanoparticles, and therefore the core–shell formation with an Au-rich particle shell takes place. For AgAu alloy nanoparticles larger than 40 nm, the pores form only above 0.75 V (SHE), and a significant shrinkage of these dealloyed particles is observed.

**Figure 7:**
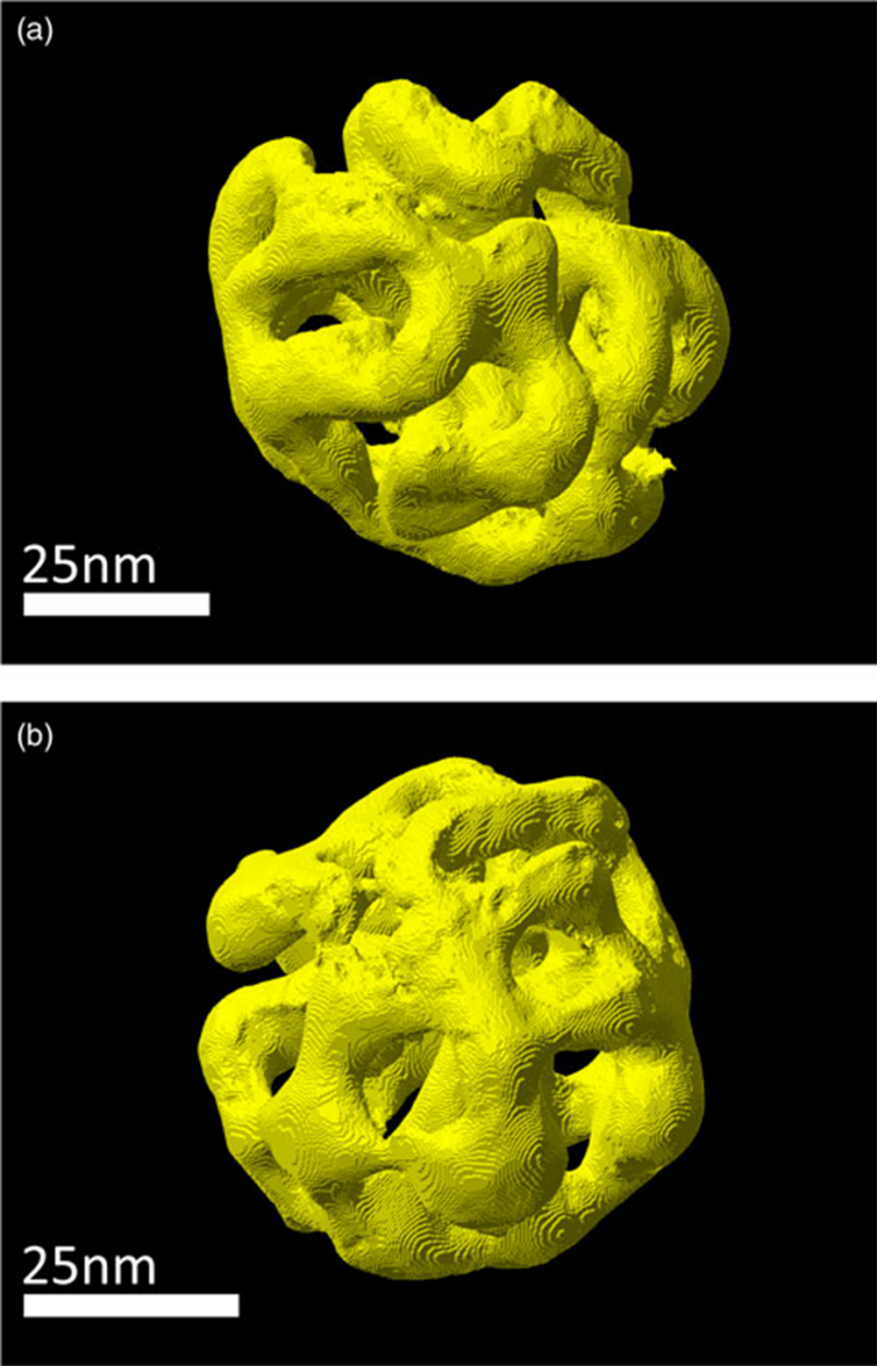
Three-dimensional STEM tomography reconstruction of porous nanoparticles prepared by dealloying. (a) particle I, (b) particle II. Reproduced with permission from ref (245) Copyright 2021 The Author(s) under License CC-BY.

A comprehensive reconstruction study of porous Au nanoparticles prepared by dealloying has been reported.^245^Figure 7 illustrates a reconstruction of a single representative porous Au-rich particle prepared by electrochemical dealloying. Structural information like pore size, porosity, specific surface area, and tortuosity were determined for porous Au-rich nanoparticles with an initial diameter of 77 ± 22 nm, which can be used for the simulations and models of diffusion and mass transport phenomena in confined environments.^246^ Two types of dealloyed porous Au nanoparticles with different ligament size and residual Ag content could be identified: Porous nanoparticles with smaller ligaments (∼14 nm) show a low residual Ag content (*x*_Ag_^res^ ∼6%). In contrast, higher Ag content (*x*_Ag_^res^ = 20%) is observed for porous nanoparticles with larger ligament sizes (∼23 nm). However, bulk nanoporous Au-rich materials prepared by dealloying show an opposite behavior. Moreover, the porosity and ligament size of the dealloyed nanoparticles were evaluated by a statistical analysis method, so-called chord length distributions.^247−249^ For these analysis a large number of arbitrary straight lines is placed within the reconstructed volume. The so-called chords can be divided whether they are within the material or pores and averaged over their length, giving statistical information on pore and ligament size. Very interestingly, the dealloyed nanoparticles published in ref (245) show less porosity compared to macroscopic materials published in ref (137). It is expected that a high porosity is associated with a large specific surface area. The bulk materials, yet possess a lower specific surface area compared to the porous nanoparticles. This discrepancy might be explained by the differences in the structure and interconnection between the ligaments and pores between bulk materials and nanoparticles.

### 2.9 Other Ways to Meso- or Macroporous Gold Electrodes

#### 2.9.1 Direct Electrodeposition of Porous Gold Electrodes

Studies of nanoporous gold in the context of catalysis invite the comparison to other, meso- or macroporous gold electrodes. Selected procedures toward such electrodes will be briefly discussed here. Porous gold can be electrodeposited using sacrificial nanoparticles as templates.^250,233,251^ The template particles can be arranged to inverse opal structures followed by electrodeposition of gold, yielding a macroporous material that can be refined by the use of different template nanoparticles (Figure 5C).^233^ Instead of particle templates, H_2_ bubbles can serve as template.^18,252−255^ The resulting material has a wide distribution of pore sizes (Figure 5B), but the process can easily be applied to substrates electrodes of almost any shape and size. The direct deposition of alloys is also possible.^18,255^ Sophisticated template structures have been used such as diatoms, i.e., marine microorganisms with an exoskeleton.^256^ Those exoskeletons exhibit regular nanostructures in a large variety of shapes that can be selected by choosing a specific kind of diatom as template.

Electrodeposition can also be guided by electrolyte additives and control of the current density to yield directly a flexible porous material with interwoven needles similar to the structure of paper.^257^ Powders of different metals can also be sintered to a porous network of alloy materials with micrometer-sized feature sizes followed by dealloying to form NPG.^206^

#### 2.9.2 Assembly of Nanoparticles to Porous Gold

Some other approaches shall be mentioned for the specific potential to alter either composition or the macroscopic shape of the resulting porous solid. Presynthesized metal nanoparticles can be combined to an interconnected aerogel using either only Au nanoparticles or mixtures of different nanoparticles.^258,259^ Those aerogels exhibit a much higher porosity than dealloyed NPG, and the size of the struts is related to the size of the used nanoparticles. The possible use of different nanoparticles provides means for exactly controlling the resulting overall composition. The local composition may greatly deviate from the mean composition and may be used for bifunctional catalyst materials. Presynthesized nanoparticles with a hydrophobic ligand shell can also be prearranged at the air–water interface of a Langmuir–Blodgett trough.^21,260,261^ After solution exchange the initially loosely arranged particles can be connected by electroless deposition of gold yielding a mesoporous membrane with a thickness similar to that of the original nanoparticles.^21,260,261^ Nanoporous gold has also been obtained by chemical reduction of aqueous 1 mmol L^–1^ H[AuCl_4_] by ascorbic acid and using 100 mmol L^–1^ of the ionic liquid tetrapropylammonium glycine to control the nucleation. The process yields a porous material of aggregated particles that was efficient for electroreduction of nitroaromatric compounds.^262^ This area has recently been reviewed.^263^

#### 2.9.3 Coating of Au on Other Porous Electrodes

Further suggested procedures use hydrothermal growth on Ti^264^ or deposition of an Au monolayer on nanoporous copper by a galvanic displacement reaction.^265^ Galvanic displacements reactions have also been used to convert a nanostructured Co electrode to nanoporous Au/Pd electrodes.^266^

## 3 Microstructure and Metrics for its Description

### 3.1 Overview

As has been pointed out in section 2, the preparation and processing conditions of dealloying afford the controlled preparation of quite different microstructures in the nanoporous material. As the materials properties can be tuned along with the microstructure, the wide options for microstructure design provide a key asset of nanoporous gold. In consequence, understanding the microstructure–property relations is an important scientific challenge. Raising to this challenge requires, in the first place, that an appropriate and complete set of microstructural parameters is identified, that the implications of those parameters for scale and geometry of the microstructure are understood, and that their values are reliably measured for the material at hand. The present section exposes the relevant state of the art, highlighting as distinct metrics as the characteristic length scales, phase fractions, residual LNE content and distribution, connectivity, and tortuosity. Emphasis is on the relevance specifically in the field of catalysis. Recent reviews with reference to the parametrization of NPG’s microstructure can be found in refs (267 and 268).

### 3.2 Geometric Model for the Nanoporous Gold Microstructure

In the form of the “leveled-wave model”, we here introduce a toy model that provides illustration for our discussion of the microstructure metrics of NPG.^269^ Other models and approaches to 3D representation of NPG will be discussed in section 3.14. The leveled-wave model goes back to the seminal work on early stage spinodal decomposition by Cahn.^270^ Its underlying notion is that, out of a random spectrum of small fluctuations, the microstructural evolution of the decomposition selects those with a single characteristic wavelength for fastest growth. The selection results from a competition between interfacial energy and transport kinetics. Those factors are equally decisive in spinodal decomposition and in dealloying, even though the driving forces and the kinetics underlying those two processes are quite different. The resulting microstructure can be constructed in two simple steps, first superimposing plane waves of identical wavelength but different wave vector orientation and with random phase shifts, and second binarizing the resulting random field by a level cut that is selected to provide the desired phase fraction (for instance, the solid volume fraction in the case of a nanoporous solid).^270,269^ A simple strategy for selecting the wave vector directions provides the model with a 3D periodic structure, enhancing its appeal as a basis for numerical simulation.^269^ Earlier studies had generated model microstructures for NPG by following the spinodal decomposition numerically.^271−274^ The resulting geometry depends on the time at which the evolution is interrupted. The leveled-wave algorithm is free of that issue and generates random structures with unique statistical characteristics. The algorithm is also extremely facile to implement and execute.

**Figure 8:**
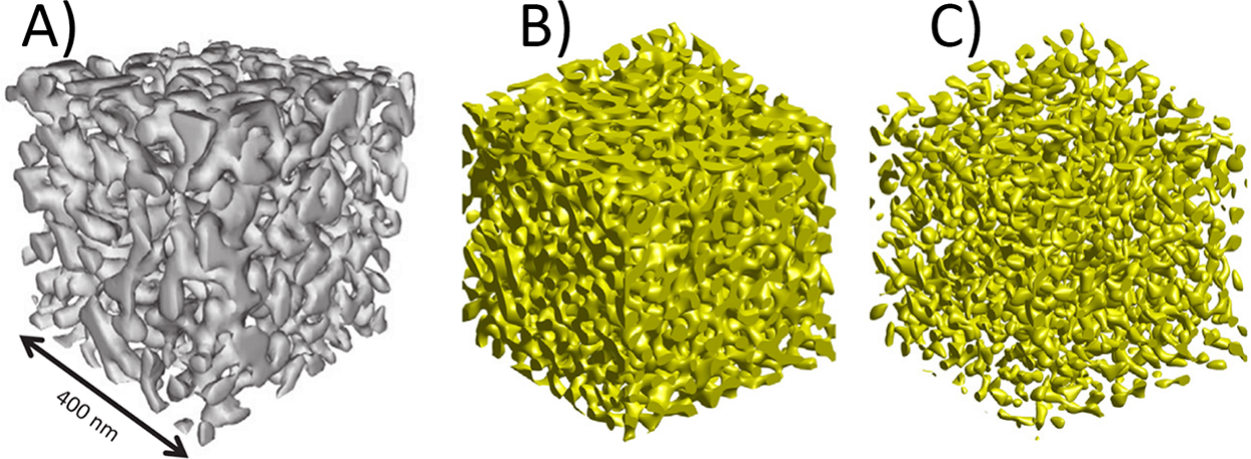
3D geometry and topology of NPG’s microstructure. (A) Rendering of an experimental tomographic reconstruction; solid fraction φ = 0.30.^124^ (B) Rendering of a leveled-wave model generated structure with φ = 0.30.^269^ (C) Rendering of the identical realization of the leveled-wave model, yet with φ = 0.10.^269^ Note the remarkable qualitative agreement between experimental and model structures. Note also that the model structure with φ = 0.10 represents an array of disconnected clusters, not a percolating and loadbearing network. (A) Reproduced with permission from ref (124). Copyright 2016 Taylor and Francis Ltd. (B,C) Reproduced with permission from ref (269). Copyright 2016 The Authors.

Figure 8B,C illustrates a typical leveled-wave structure^123,269^ and sets it next to an experimental 3D tomographic reconstruction of NPG (Figure 8A).^124^ The close resemblance of the microstructural geometries is apparent. What is more, leveled-wave structures have been found to accurately reproduce, with no free parameters, the dependence of Young’s modulus, Poisson’s number, and yield strength on the solid fraction for as-prepared NPG.^177,269^ The model also accurately reproduces a topological feature, the scaled genus (see section 3.9) of as-prepared NPG.^124,269^ As an added benefit of the leveled-wave model, many of its microstructural metrics, including characteristic size, solid fraction, volume-specific surface area, and averages of the mean and Gaussian curvatures of the surface, are interrelated by exact analytical expressions.^269^

The characteristic length-scale of the leveled-wave model is set by the underlying wavelength, which can be chosen at will. Applying the model to NPG rests on the tacit assumption that NPG microstructures of different characteristic microstructural length scale (different ligament size) are self-similar, which is discussed in section 3.9. Available studies present strong evidence for the applicability of the model to as-prepared NPG, irrespective of the specific preparation protocol or the ligament size.^118,177,269^

### 3.3 Metrics for the Characteristic Length Scale

**Figure 9:**
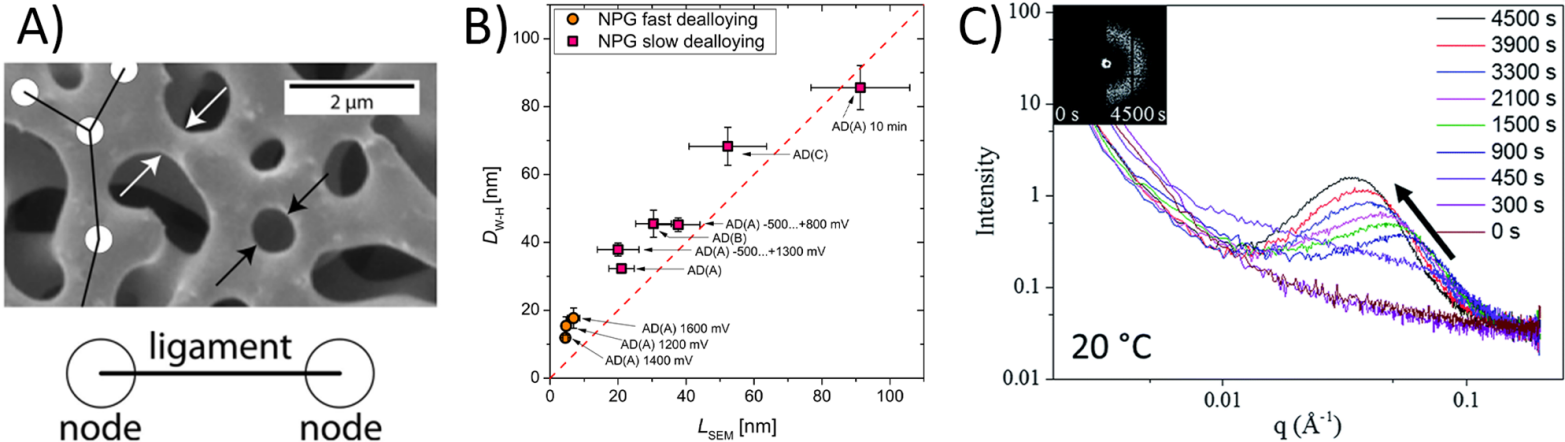
Selected examples for signatures of and experimental approaches to the ligament size. (A) SEM-based evaluation of a size, *L*_SEM_, by analysis of the characteristic diameter of ligaments at their waist (white arrows).^129^ Pore size (dark arrows) and the skeleton of the ligament network (legend) are also indicated. (B) Analysis based on Bragg reflection width (Williamson–Hall analysis) in wide-angle X-ray powder diffraction.^104^ The Williamson–Hall size, *D*_W–H_, is systematically correlated to *L*_SEM_, validating this analysis for measuring the ligament size. (C) In situ small-angle X-ray scattering showing the characteristic interference peak of NPG and its evolution to lesser wave numbers as the structure coarsens during dealloying at 20 °C.^283^ The mean spacing between neighboring ligaments can be inferred from the peak position. (A) Reproduced with permission from ref (129). Copyright 2018 under license CC-BY-4.0. (B) Reproduced with permission from ref (104). Copyright 2017 under license CC. (C) Reproduced with permission from ref (283). Copyright 2017 under license CC 3.0.

Undisputedly, one of the most important microstructural parameters of any given sample of NPG is the magnitude of its characteristic length scale. This scale determines area per volume and, thereby, the fraction of atoms exposed at the surface and available for catalysis. It also determines size effects controlling mechanical strength,^26,141,275^ optical function,^214,276,277^ and electric resistance^222,278,279^ of the nanomaterial. The importance of the characteristic length scale for so diverse phenomena emphasizes that the option of tuning this scale within wide margins (section 2) is a distinguishing asset of NPG. Inspection of the microstructural geometry, for instance in Figures 8 and 9A, immediately reveals that any single notion of “size” may not adequately cover all relevant aspects of the geometry. One may focus on different features, e.g., a characteristic diameter of the struts or “ligaments” that make up the network material or, alternatively, a characteristic spacing between such features, and one may be interested in various measures for the distribution of the feature in question. In other words, there is no such thing as a unique definition of size, and one must expect that different measures for size may be relevant for different phenomena. That situation is ubiquitous in materials science, and specifically it can be relevant for the materials performance in catalysis.

The most common approach to parametrizing the characteristic length scale for NPG is evaluating “the ligament size” by measuring diameters along the waists of ligaments in scanning electron micrographs (Figure 9A).^24,92,105,118,173,190,280−282^ This is done manually for a certain number of ligaments, and the mean diameter, *L*_SEM_, is reported. Good practice requires that the number of ligaments counted and the variance of the diameters be specified. As macroscopic samples of NPG tend to have reduced (relative to bulk) *L*_SEM_ close the external surface,^34,92,173^ good practice also requires that micrographs from the interior of the sample are examined and that their location is specified.

For nanoparticles and nanocrystalline materials, the evaluation of X-ray line broadening is a standard approach to characteristic size.^284^ The simple evaluation of the breadth of the strongest Bragg reflection in terms of the Scherrer formula must be rejected because it does not discriminate between size and microstrain broadening, thus it systematically and strongly underestimates the size. Variants of the Williamson–Hall approach avoid that error and are readily applied whenever a sufficient number of Bragg reflections are available.^284,285^ NPG exhibits substantial microstrain (section 3.12) that systematically increases with decreasing ligament size, emphasizing the need for Williamson–Hall type correction.^104^ In NPG, an apparent complication resides in the presence of a coherent crystal lattice that extends to dimensions much larger than *L*. That obliterates the simple analysis of the impact of coherency on the X-ray line broadening of the standard diffraction theory. Nonetheless, empirical data for NPG show an excellent correlation between the *L* values derived by Williamson–Hall and SEM (Figure 9B),^104^ validating X-ray line broadening analysis as a method for measuring the ligament size of NPG.

Experimentally more demanding approaches to measuring a mean ligament size involve the analysis of 3D reconstructions.^124,126,137,245,286−288^ This may provide variously defined measures for size. An example, based on “granulometry”, is the mean, *L*_G_, over all materials points in the solid phase, of the diameter of the largest in-fitting sphere containing the respective point.^137,289^ A closer inspection of such approaches reveals various issues that require attention.

First, the sampling of space in granulometry emphasizes the wider regions of the microstructure, providing a comparatively large characteristic size.^289^ A related algorithm, combining skeletonization with a euclidean distance transform,^145,290^ provides around 30% smaller size for NPG.^268,291^ This algorithm has been applied in a data mining approach to coarsening of NPG,^129^ yet its relation to other measures for *L* remains to be established.

Second, whenever there is a distribution of sizes, averaging is required for condensing the data into a single size parameter. The averaging involves a decision on the weighting function, for instance number-, area-, or volume-weighted averaging, and the resulting average values can differ strongly.^292^ Different materials phenomena may depend on different weighting functions, and this interrelation is poorly appreciated or understood in the field of NPG.

The characteristic spacing, *L̃*, between the centers of neighboring ligaments is another obvious measure for size.^269,293^ A robust signature for *L̃* of aperiodic structures is provided by the position of the first maximum in their autocorrelation function. For NPG and the leveled-wave model, *L̃* defined in this way has an immediate relation to the structure factor or interference function. As a result of the Debye formula,^294,295^ that function exhibits a distinct peak with a maximum at the wavenumber^269^
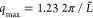
2Indeed, experimental small-angle X-ray scattering data of NPG exhibit a pronounced interference peak (Figure 9C). Various studies have measured *L̃* from the position of the peak in neutron^8^ or small X-ray angle scattering^125,205,283,296^ data, or from a maximum in the numerical Fourier transform of electron micrographs.^93,293^ In one instance,^293^ an equation similar to (eq 2) but without the prefactor was assumed ad hoc. The characteristic length evaluated in that way is smaller than the characteristic spacing *L̃*.

### 3.4 Specific Area of Surface

Another common microstructural parameter related to characteristic size is the “specific surface area”, which is again linked to the number of sites available for catalysis. The specific surface area also determines in how far capillary forces induce pressure in the bulk. As will be discussed in section 3.12, that surface-induced pressure generates strain throughout the crystal lattice, which in turn may affect the catalytic activity. The surface-induced pressure is also of relevance for actuation^100,209,297−300^ or active strain sensing^301^ with NPG and for its strength.^111,188,302^

The specific area of surface is sometimes specified as area per mass, α_m_, and then has no immediately obvious relation to size. Various scientific communities have their own distinct notions on how mass-specific surface area provides a figure of merit. Yet, the related numbers mix a microstructure property (area of surface per volume) with a materials parameter of the solid phase, namely its mass density. Obviously, the same microstructural geometry will provide vastly different α_*m*_, when it is realized as porous carbon as compared to porous gold. The parameter α*_m_* is therefore not very informative when it comes to understanding the microstructure; thus, its use in studies of NPG is discouraged.

The area, α_V_, per volume of the solid phase relates to microstructural geometry alone and should be preferred. In fact, this parameter has a particularly close relation to measures for the ligament size. That is apparent when one considers idealized cylindrical ligaments of diameter *L*, which have simply α_V_ = 4/*L*. Consequently, a specific-surface-area related size, *L*_α_, may be defined as
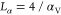
3Through its product with the surface stress, *f*, along with the generalized capillary equation for solids, ,^303^ the specific surface area α_V_ determines the surface-induced mean pressure in a nanoscale solid. Thereby, α_V_ also parametrizes the impact of the microstructure on the size-induced change in the mean lattice parameter of NPG (see section 3.12 below).

In most experimental situations, the mass density of the solid phase is known with sufficient confidence and accuracy to afford computing the volume of the solid phase based on the mass (which, typically, is readily measured). By contrast, determining the surface area of porous electrodes is a more complex issue. The topic has been thoroughly reviewed by the IUPAC commission.^304^ There are several options, and each has advantages and disadvantages. Their application to NPG has been evaluated and compared,^173,305^ leading to the recommendation that either the charge for surface oxidation and subsequent reduction should be evaluated or the charge consumed in the underpotential deposition (UPD) of Cu. UPD of Pb has also been used^106^ and provides additional information on the prevailing facets in NPG (see section 6.1.2 below).

**Figure 10:**
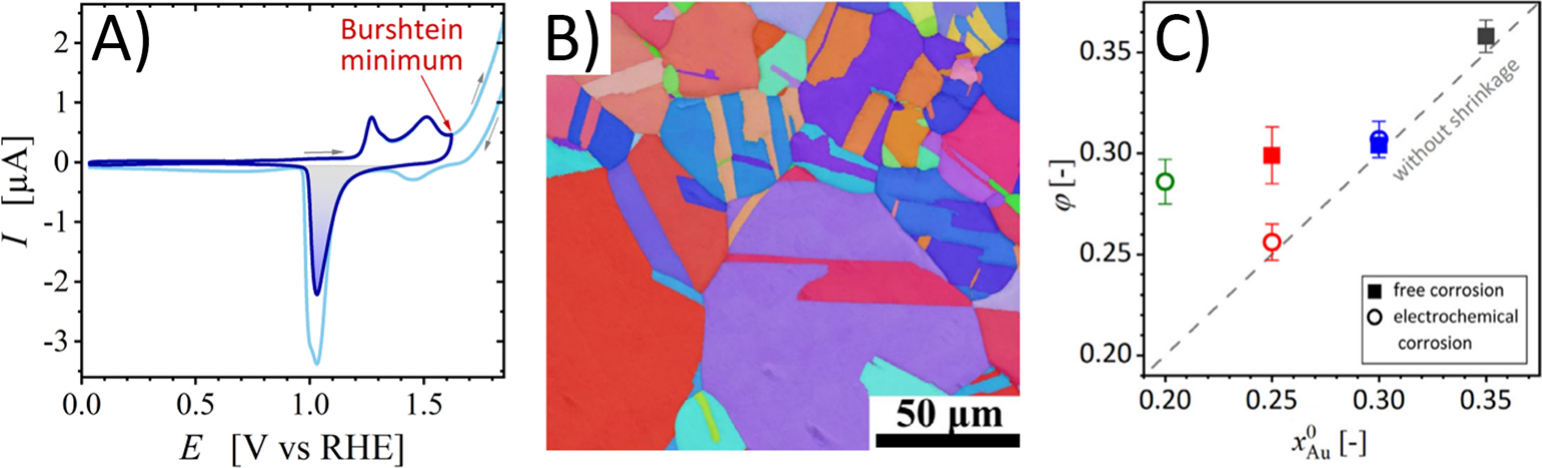
Selected examples for additional microstructural signatures. (A) Cyclic voltammogram of current *I* versus electrode potential *E* for NPG in deaerated 0.1 mol L^–1^ NaOH. Dark blue: scan to the switching potential at the Burshtein minimum (indicated by label). The shaded area under the surface oxide reduction peak scales with the surface area and can be used to measure that quantity. Light blue: scan to larger positive switching potential; note the larger reduction charge. (B) Grain size of NPG from electron backscatter diffraction in a scanning electron microscope.^115^ Color codes crystallographic orientation, identifying individual crystal grains as regions of uniform color. Note up to 100 μm grain size, more than thousandfold larger than the ligament size, *L*_SEM_ = 18 nm here. (C) Solid fraction, φ, of NPG versus initial Au fraction, *x*_Au_^0^, in the starting alloy.^118^ Strong deviation at low *x*_Au_^0^ shows that the starting alloy composition does not provide a good indicator of NPG solid fraction. Note also the dependency on the preparation protocol, free versus electrochemical corrosion. (B) Reproduced with permission from ref (115). Copyright 2021 from Elsevier. (C) Reproduced with permission from ref (118). Copyright 2021 The Authors.

Because the UPD process ultimately changes the surface state, and it might be impossible to completely remove Cu^2+^ from the surface and the pore space, the authors of the present review prefer the determination based on the formation of surface oxides (Figure 10A). The surface is oxidized up to the Burshtein minimum^306^ at around 1.65 V in Figure 10A. At this potential, an AuO layer is formed on all facets of Au. Its reduction is associated with a charge of 386 μC cm^–2^,^307^ and dividing the net charge by that value defines the “electrochemically active surface area”, *A*_ECSA_. This quantity may be identified with the geometric area of surface in a description of the NPG microstructure, permitting the computation of α_V_ and, with eq 3, of the characteristic size *L*_α_. Slightly different values for the area-specific reduction charge of an AuO layer have been determined by other authors. One advantage of the oxidation/reduction approach is that, even though the peak widths may vary, it can be applied in different aqueous electrolytes. The disadvantage is a possible change of the surface structure already during one potential excursion to the Burshtein minimum, which is especially evident in acidic electrolytes.^191^ This is related to the high mobility of Au on the rough surface that is generated, as a transient state, when the oxygen adsorbate layers are lifted.^308^ In fact, as one instance of electrochemical annealing, repeated oxidation/reduction cycles on NPG are sometimes deliberately instigated in order to induce coarsening.^121^

The commonly applied measurement of the double-layer capacity^92,167,173,178,305,309,310^ in the potential range below the onset of surface oxidation is plagued with several problems.^304^ It can only be recommended for a relative comparison of very similar samples within one laboratory after optimizing the procedure with respect to the used range of scan rates, the nature and the concentration of the electrolyte. An approach based on electrochemical impedance spectroscopy provided consistent values.^305^ However, those values were clearly distinct of the results obtained from UPD and surface oxide procedures.^305^

For catalysis with NPG, the numerical value of the material’s specific surface area is of relevance. As NPG can be prepared with a wide range of ligament sizes, α_V_ can take on vastly different values. Inasmuch as α_V_ can be approximated as 4/*L*, typical *L* values of 20–40 nm (see section 2.2) translate into volume-specific surface areas of α_V_ ≈ 0.1–0.2 nm^–1^ and mass-specific areas of α_m_ ≈ 5–10 m^2^ g^–1^. Yet, smaller ligament sizes can be stabilized, for instance, by alloying with Pt (see section 2.2). For *L* = 4 nm, one then expects α_V_ and α_m_ as large as ≈1 nm^–1^ and 50 m^2^ g^–1^, respectively.

In principle, Brunauer–Emmett–Teller (BET) adsorption isotherms provide an alternative approach to the specific surface area of NPG. Agreement with electrochemical oxide stripping data has been achieved.^311^ Yet, large quantities of the material are required for meaningful data, and this has prevented widespread use of the technique. Furthermore, NPG tends to coarsen during the heating cycle that is required for removing physisorbed monolayers of water, a prerequisite for meaningful BET data.^106^ The adsorption will then not probe the original sample state, and the area value may emerge erroneously low. Indeed, comparative studies found the BET surface area drastically less than that obtained from the capacitance ratio^312^ or Pb UPD^106^ methods. So far, BET has not been established as a routine approach to surface area measurement for NPG.

The evaluation of small-angle X-ray scattering in its high-wavenumber (“Porod”) regime provides yet another approach to α_V_.^171^ However, the required reduction of the scattering intensity to absolute units is rarely applied. Other approaches to a volume-specific surface area include the analysis of 3D representations^124,126,137,286^ and of the initial slope of the microstructure’s autocorrelation function.^90^

Besides α_V_, the area per total volume (solid plus pores) may be considered. That parameter depends not only on *L* but also on φ; it can differ considerably from α_V_. This emphasizes that it is imperative to disambiguate specific surface area data by specifying which measure is used.

### 3.5 Comparison of Different Measures for Ligament Size

**Figure 11:**
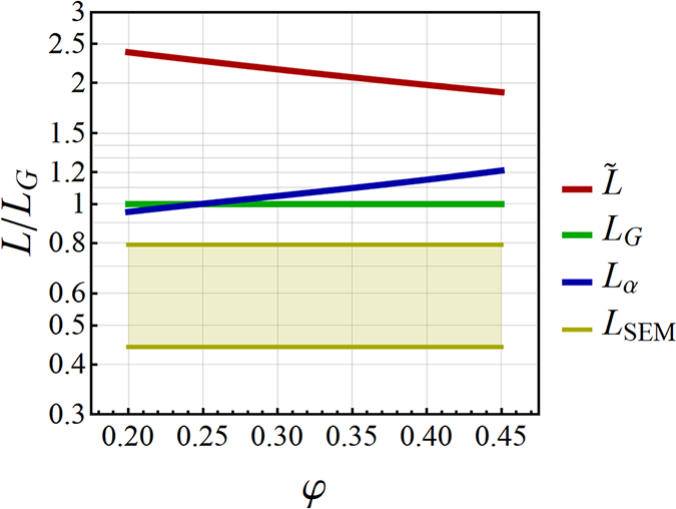
Comparing different measures for the ligament size and their dependency on the solid fraction, φ. All sizes are normalized to the granulometry mean ligament size, *L*_G_, and shown with logarithmic axis scaling. The geometry of the leveled-wave model is assumed to approximate NPG. *L*_α_: size derived as 4/α_V_ from α_V_, the area of surface per solid volume. *L̃*: mean spacing between neighboring ligaments, as derived by eq 2, from the interference peak position in the structure factor. *L*_SEM_: mean diameter of ligaments at their waist as determined from scanning electron micrographs. Shaded area: confidence limit. See SI, SI-1, for detailed discussion and references. As emphasized there, the data for *L*_SEM_ in this figure must be considered as tentative, simply illustrating that this measure for size provides particularly small values. Additional experiments are required for quantifying the relation between *L*_SEM_ and the remaining measures for size.

How are the size measures *L*_SEM_, *L*_G_, *L̃*, and *L*_*α*_ interrelated? In general, materials can have widely different microstructural morphologies that allow for individually quite different relations between those size measures. Yet, inasmuch as the microstructure of NPG can be approximated by that of the leveled-wave model, one can find approximate conversion rules between those quantities. Figure 11 presents those rules, and the Supporting Information (SI), SI-1, shows their derivation. In essence, it is based on the following insights from the literature. First, results in ref (269) imply an exact close-form relation between *L̃* and *L*_*α*_ for the leveled-wave morphology. Second, Richert et al.^289^ present *L*_G_ for that morphology based on numerical evaluation. Third, there are experimental data for *L*_SEM_ and α_V_ (which imply *L*_*α*_) for one and the same set of NPG samples.^310^

As motivated in SI, SI-1, *L*_G_ has been chosen as the reference quantity in Figure 11. The comparative display emphasizes that the numerical values of the individual measures for size can be quite different. Two important insights are that the difference depends rather weakly on the solid fraction and that *L*_SEM_ provides the smallest size values. That latter observation is not surprising in view of the fact that the measurement probes the dimension of the ligaments at their thinnest cross-section. Furthermore, the figure illustrates that *L̃* is twice or more larger than the other size parameters. This is natural because the distance between the centers of neighboring ligaments represents a combination of ligament and pore-related sizes. An obvious consequence of the compilation in Figure 11 is that different measures highlight different aspects of the microstructural geometry; in other words, there is no such thing as “the true ligament size” of NPG.

Uncertainties in the data set^310^ comparing *L*_SEM_ to *L*_G_ are responsible for the quite large confidence interval for *L*_SEM_ in Figure 11. In fact, and as is argued in SI, SI-1, the available experimental data in the literature provide at best weak support for the assumption of a linear scaling between *L*_SEM_ and *L*_G_, as it underlies the representation in Figure 11. Additional experiments are needed as a basis for a reliable conclusion on that scaling. The result, as it stands, however, confirms the expectation, based on the definitions of the various size parameters, that *L*_SEM_ provides a particularly small numerical value compared to the remaining measures, possibly about twice smaller than the granulometry ligament size.

### 3.6 Grain Size

The various measures for ligament size relate to the microstructural length scale of primary interest for NPG. The grain size constitutes an additional length scale. Based on ion channeling^102^ and electron backscatter diffraction images,^115,179,280,282^ it is well established that dealloying Ag–Au to make NPG conserves the grain structure of the master alloy. For standard preparation protocols of macroscopic NPG samples, grain sizes in the order of 50 μm are typical (Figure 10B).^179^ In other words, even though ligament sizes can vary between a few nm and few μm, the crystalline coherency length is typically very much larger. In fact, dedicated protocols allow NPG to be made in the form of mm-sized single crystals,^313,314^ and micrometer-sized single crystals of the material are readily prepared by focused ion beam cutting from standard samples.^141,142,275^

While the retention of the coarse grain structure of the master alloy during dealloying is well established for NPG, the finding is by no means obvious. The success of KMC simulations (see section 2.2) on rigid lattices, which maintain crystalline coherency by construction, verifies that the nucleation of new crystals is not inherently necessary for forming the nanoscale pore structure during dealloying. Dealloying Ag–Au is particularly close to that simulation scenario for several reasons. First, the two constituents Ag and Au form a complete series of solid solutions down to room temperature, second, they share the same crystal lattice symmetry, and last, their lattice parameters are exceptionally close, agreeing to within 0.19%.^181^ This favors a continuous transition from the Ag-rich solid solution to the Au-rich product phase, on the same crystal lattice. Yet, experiments find the crystal lattice structure retained even under much less ideal conditions. Most notably, NPG prepared by dealloying Cu–Au also retains the master alloy grain size,^154,156^ even though the lattice parameters differ by 12%, 60-fold more than for Ag–Au.^182^ In spite of the large misfit, epitaxial layers of Au on the Cu-rich host crystal have been demonstrated by in situ diffraction during dealloying.^315^ A large crystalline coherency length has also been reported in NPG made by dealloying the intermetallic compound Al_2_Au,^96,316^ suggesting that a one-to-one match of the crystal lattice structures of host and product is not mandatory for coarse-grained NPG. Apparently, a substantial amount of lattice parameter misfit can be accommodated by elastic strain. This is reminiscent of the well-known phenomenology of precipitation from supersaturated metallic solid solutions, where nanoscale precipitates tend to minimize their interfacial excess energy by remaining coherent, even at the expense of elastic distortion.^317^

Full solubility is also not required for coherency at the dealloying front. This is exemplified by Ag–Au–Pt, which can be dealloyed into single-phase nanoporous equiatomic Au–Pt with a crystalline coherency length much larger than the ligament size, even though Au and Pt are immiscible at room temperature and equilibrium.^119^

Besides the idealized structure of typical NPG samples, with nanoscale pores in an extended coherent crystal lattice, dealloying can also produce nanocrystalline nanoporous metal. The grain size can then be comparable to the ligament size. The dealloying of Au-based metallic glasses exemplifies this situation.^318,319^ Nanocrystalline nanoporous metals have also been obtained by dealloying crystalline precursors, for instance, from Al-^320^ and Mn-based^197^ master alloys. The absence of simple epitaxial relations between master alloy and the product structure may favor such nanocrystalline nanoporous products. For NPG made from Ag–Au, the grain size can be reduced by working with severely plastically deformed master alloys,^282^ and the number density of crystal lattice defects can be strongly enhanced by dealloying at very high driving forces.^102^

### 3.7 Phase Fractions

Next to *L*, the solid volume fraction, φ, is a fundamental microstructural parameter of a porous solid. This parameter adds up to unity together with the “porosity” (i.e., the pore volume fraction). It is relevant for transport cross sections in the pore space^321^ and for mechanics.^26^ As exposed in section 2, the external sample dimensions are preserved during an idealized preparation by dealloying (assuming that differences in the atomic volumes of the constituents can be neglected, which is a good assumption for Ag–Au). Inasmuch as the idealized process also removes all of the less noble element, φ would then agree with the more-noble element atom fraction in the starting alloy. Yet, φ-values estimated in this way may be considerably in error. This is due to spontaneous plastic deformation during dealloying. As a general rule, this phenomenon leads to substantial shrinkage, acting to increase φ. The volume reduction can range from 2% to 25% in standard dealloying scenarios,^91,92,102,118^ and 40% or more reduction have been reported for starting alloys dilute in Au, that is, with gold atom fraction ≤0.2.^118,167^ The retention of Ag (section 2) can further increase φ.^322,323^ These observations imply that reliable values of φ may not be inferred from the starting alloy composition; instead, φ requires a dedicated measurement. Figure 10C illustrates that φ of NPG can deviate substantially from the Au fraction in the starting alloy.

Classic Archimedes density measurements are problematic for solids with open porosity, such as NPG. Thus, the standard approach to φ is the separate measurement of sample mass and of external sample volume (typically using an optical measurement microscope).^110,118^ As an equivalent approach, the sample volume can be estimated from the starting alloy sample dimensions and the relative length change, measured by in situ dilatometry^102,143^ or from the displacement of markers on the sample surface,^150^ during dealloying. That approach presupposes isotropic dimension change.

The amount of densification by shrinkage during dealloying increases systematically with the dealloying rate.^102^ This appears natural as faster dealloying brings lesser *L* and,^90,104^ hence, more impact of surface stress and surface tension. The densification is also strongly dependent on the dealloying protocol, with substantially more shrinkage during free corrosion as compared to electrochemical corrosion.^92,118^

The microscopic mechanisms behind shrinkage during dealloying have not been conclusively identified.^91^ As sintering can be ruled out in view of the large grain size, plastic deformation appears the obvious process.^102^ It has been suggested that the shear components of the stress within the ligaments, induced by their surface stress, prompt spontaneous plastic shear by dislocation glide.^102^ Yet, it is not obvious which capillary parameter drives the shrinkage–surface stress (parametrizing a local stress state in the surface) or surface tension (parametrizing the area-specific excess energy of the surface). In fact, investigations of the impact of the surface on plasticity of NPG single out surface tension, as opposed to surface stress, as the relevant capillary parameter.^117,302^ When NPG shrinks, its ligaments become shorter and thicker, thereby reducing their surface area. Because that process also reduces the overall excess energy residing in the surface, surface tension provides a driving force for shrinkage.

### 3.8 Skin Layers on Macro- and Nanoscale Porous Bodies and Disconnection of Thin Films

The shrinkage and densification discussed so far arise from deformation processes that act throughout the bulk of NPG. On top of these processes, there is also a separate densification process that acts near the external sample surface and that results in a skin layer of locally enhanced solid fraction, with a thickness comparable to the ligament size.^104,173^ That skin layer has little effect on the external dimensions or on the mean solid fraction of macroscopic samples, yet it can contribute decisively to the densification of nanoporous nanoparticles (section 2.8). A volume shrinkage, during dealloying, of up to around 50% has been reported for nanoparticles.^79^ Their densification is enhanced at lesser particle size, and the magnitude of that effect has been found consistent with a dense skin layer underneath the surface.^324^

A recent study has confirmed that the subsurface densification originates in enhanced inward MNE diffusion fluxes near the external surface. Those fluxes act during secondary dealloying or coarsening, and they originate in the enhanced convexity of the ligament surfaces near their termination at the external surface of a porous body.^325^ The same process also leads to an opposite effect, namely loss of density and disconnection, at the interface between NPG and support layers of massive (not porous) gold. This is of relevance because it leads to the widely observed, but rarely acknowledged, observation of a low-density, poorly connected layer at the base of thin films of NPG.^325^

### 3.9 Connectivity, Percolation-to-Cluster Transition, and Self-Similarity

A defining feature of NPG as a catalyst is that one and the same, homogeneous material provides both the active surface sites at the atomic scale and the supporting macroscale scaffold. This presupposes mechanical integrity and not too low strength and stiffness. In other words, percolating loadbearing pathways within the material are essential. For electrocatalysis, the scaffold also needs percolating paths for the conduction of electrons. It is therefore of interest to characterize a degree of connectivity within the solid phase of NPG’s microstructure.

Reports of an anomalously low elastic stiffness in NPG^116,274^ first led to the suggestion that “broken” ligaments may be inherent in its microstructure^116,271,326^ and that its connectivity may deteriorate upon coarsening.^26,323^ Comparing the mechanics of the leveled-wave model to experiment confirmed that notion and revealed that the connectivity of the as-prepared material systematically depends on φ.^269^

An important parameter in the above context is the topological genus, *G*. In essence, *G* counts the number of interconnected (as opposed to broken) ligaments within a sample of the material.^327^ Normalization to the characteristic volume of an elementary structural unit in the microstructure leads to a “scaled genus”, *g*, that is independent of *L* or of the sample volume and so provides a measure for the inherent contiguity of the material’s microstructure.^124,267,271^ Experimental tomographic reconstruction suggests excellent agreement between *g* in as-prepared NPG and in the leveled-wave model.^269^ Significantly, that model features a strong correlation between *g* and φ: as the solid fraction decreases, so does the connectivity. This effect is strong and it abuts in a percolation-to-cluster transition at the solid fraction ,^269^ see exemplification by the φ = 0.1 leveled-wave structure in the right panel of Figure 8C. At even lower solid fractions, regular dealloying will not form load-bearing bodies of NPG. Furthermore, strength and stiffness of the material decrease dramatically as φ approaches φ_perc_ from above.^269^ Hierarchical structuring (section 2.5) has been shown to afford bodies of NPG at solid fractions below φ_perc_ that remain loadbearing, beating the percolation limit of structures with a single characteristic length scale.^177^

The topology-changing event in the microstructural evolution of NPG is the breaking of ligaments through a Plateau–Rayleigh-like instability.^327^ The link between solid fraction and scaled genus has been rationalized as a consequence of this instability: low φ requires long, thin ligaments, and these will disconnect more readily by the instability.^123^ That is also in keeping with the observation that NPG with φ > 0.3 (low aspect-ratio ligaments) tends to maintain *g*, whereas NPG with φ < 0.3 will gradually disconnect upon coarsening.^123,323^ Reconnection has been shown to partly compensate for the disconnection.^115^

The observations on the evolution of the connectivity during coarsening link to the question, will the NPG microstructure evolve self-similarly in that process?^267^ Experimental studies have been discussed as evidence for^124,288,328^ or against^116,126,323^ that notion. The more recent work highlights the dependence of the connectivity evolution on φ as a possible origin of the discrepancy,^123^ the samples in the respective studies may have been partly above and partly below the threshold value of φ ≈ 0.3.

To summarize, the state of the art provides good arguments for expecting NPG with φ > 0.3 to coarsen self-similarly. By contrast, NPG with lesser solid fraction must be expected to disconnect and/or densify during coarsening.

### 3.10 Tortuosity, Multiscale, and Hierarchical Nanoporous Gold

The geometry of NPG implies that mass transport in the pore space is slowed down as it follows sinuous pathways that may feature constrictions. The notion of “tortuosity” parametrizes that situation in an effective continuum description of transport. The precise nature of the link between microstructure and transport rate depends on the transport process, for instance, molecular diffusion or viscous flow.^329,330^ Consequently, there are different notions of tortuosity.

A tortuosity parameter for viscous flow in NPG has been inferred from imbibition experiments, and its value, τ = 3.2 ± 0.2, was found^331^ to be compatible with that, τ = 3.5 ± 0.3, reported^332^ for porous Vycor glass.^333,334^ The agreement is in keeping with the notion that the Vycor microstructure, resulting from spinodal decomposition, is equally well compatible with the leveled-wave model^335^ as is NPG. Branch and geometric tortuosities for NPG, with values in the range 1.2 < τ < 2.0, have been estimated based on φ,^321^ or on tomographic reconstructions of nanoparticles.^245^ Apparently, these parameters remain yet to be linked to observations on transport in NPG. Structural hierarchy (see details in section 2) may reduce the constrictions and accelerate transport; the tortuosity of hierarchically structured NPG remains to be studied.

### 3.11 Surface Curvature, Faceting, and Roughness

The local value of the mean curvature, κ (the sum of the inverse values of the principal radii), at a given point on the surface is an important parameter, because gradients in κ provide the driving forces for transport of matter by surface diffusion that lead to coarsening. The curvature is linked to an excess, Δμ, in chemical potential by the Gibbs–Thomson equation, Δμ = κγΩ, with γ the surface tension (i.e., specific surface excess free energy) and Ω the atomic volume (see, e.g., refs (101 and 336)).

The specific surface excess free energy (“surface tension”) of the continuum thermodynamics description is reflected, at the atomic scale, by broken bonds at low-coordinated surface atoms. The atomic coordination number is reduced at any type of crystallographic surface facet. It is reduced even further at step-edge and corner lines or on rough regions of the surface. The effective Δμ of a nanostructure is controlled by its size, and it is otherwise sensibly independent of whether a surface is rough or faceted.^336^ This underlines that low-coordinated atom positions, of central importance for catalysis, are an inherent and geometrically necessary property of nanostructures such as NPG, irrespective of whether their surfaces are rough or faceted. The presence of low-coordinated sites in NPG and their relevance for catalysis are well acknowledged.^5,16,23,93,337,338^

High-resolution transmission electron microscopy reveals microfacets (at the scale of few atomic diameters) at the surface of NPG.^93,337^ Transitions in the crystallography of the microfacets can be induced by changes of the environment^337^ or by electrochemical cycles,^107,191,339^ see section 4.4. In view of the established relevance of surface crystallography for catalysis, it can be expected that these details of the microstructure are important for the catalytic activity of NPG.

Equilibration of the NPG surface structure at the scale of the ligament size requires a prevailing presence of the equilibrium facets of the Wulff shape at that scale (see, for instance, ref (340)). The Wulff shape of gold exhibits facets at any temperature, up to the melting point.^341,342^ Indeed, NPG with faceted ligaments at the scale of *L* may be obtained by a custom annealing protocol.^120^ Yet, the mainstream of scanning electron micrographs of the NPG microstructure at that scale presents smoothly rounded and, hence, rough surfaces.^120^ Thus, the observations would seem to suggest that the kinetics of ordering by surface diffusion near room temperature permit microfaceting at a very small scale, but is too slow for the surface crystallography of NPG to reflect the Wulff shape.

Experimental 3D reconstructions find the average value, ⟨κ⟩, of κ in NPG either near zero^286,343^ or positive,^126,137,267^ with the near-zero results reported for symmetric microstructures with roughly equal volume fractions of pore and solid, .^126^ That is also borne out by the leveled-wave model, which has ⟨κ⟩ = 0 (at any *L*) when , along with a gradual increase in κ upon decreasing φ.^269^ The mainstream of studies of NPG work with solid fractions near 0.3, where convex surfaces (*κ* > 0) may be expected on average. Interfacial shape distributions, characterizing statistic correlations between the two principal radii of curvature, have been found to evolve in a roughly self-similar manner during coarsening. This has been discussed as the signature of self-similar microstructure evolution^126,267,328^ (yet, see also section 3.9).

### 3.12 Mean Lattice Parameter and Microstrain

When dealloying converts the master alloy into nearly pure (nanoporous) gold, the composition change will entail a change in the stress-free lattice parameter, *a*_0_. A small contraction of the crystal lattice after dealloying is indeed observed in NPG made from Ag–Au,^104^ consistent with *a*_0_ of Au falling below *a*_0_ of Ag by about 0.19%.^181^ Furthermore, local strains in the crystal lattice of NPG are expected because the surface stress, a capillary force that arises from modified interatomic bond forces at low-coordinated surface atoms sites, requires compensating stresses in the bulk. As addressed in section 3.4, the mean magnitude of those stresses scales with the volume-specific surface area. Thus, the lattice of NPG will exhibit stress and strain that increase in magnitude with diminishing ligament size. A dependence of the mean lattice parameter, *a̅*, on the ligament size may then arise, similar to the well-known size dependence of nanoparticle lattice parameters (e.g., refs (344 and 345)).

The size dependence of *a̅* appears as an obvious consequence of the surface-induced stresses. Yet, that effect is small: for NPG with clean surfaces and with *L*_SEM_ = 20 nm, *a̅* was found reduced, relative to the bulk value, by merely 0.03%.^104^ Stresses at the corrosion front can locally induce larger lattice strain (relative change in the lattice parameter) during ongoing corrosion.^171,280,346^ Changes in surface stress controlled through adsorption or electrochemical charging will change *a̅*, and this can be exploited for sensing or actuation with NPG.^209,297,301,347,348^ A detailed inspection of that topic is beyond the scope of this review.

While the surface-induced change in the mean value, *a̅*, of the lattice parameter, *a*, of NPG is small, the distribution of lattice parameters around the mean can be quite wide. In other words, local regions of substantially different *a* abound. This phenomenon is quantified by the “microstrain”, that is, the standard deviation of the distribution.^284^ The local lattice distortions may be important for catalysis. Density functional theory predicts that tensile strain in the tangent plane typically (experimental counterexamples show that the trend is by no means forceful)^349^ makes the surface of transition and noble metals more binding for the reactants and intermediates of heterogeneous catalysis, and this affects their reactivity.^350^ The strain-dependent reaction rate at the surface of Au has been quantified and confirmed in situ.^351^ This suggests that local strains at the surfaces of NPG ligaments will have an impact on the catalytic activity. X-ray diffraction shows that the microstrain of NPG increases systematically with decreasing ligament size. A microstrain of 0.61% was found for ligament sizes of ≈10 nm.^104^ Along with the findings for the lattice parameter, the observation emphasizes that the variance of the lattice parameter distribution along the surface is much larger than the shift of the mean lattice parameter.

Yet another aspect of strain in NPG is a rhombohedral distortion of the crystal lattice in each ligament. Nanobeam electron diffraction in scanning transmission electron microscopy (STEM) revealed a compression of the lattice along the ligament long axis, along with an expansion in radial direction by several percent.^352^ That confirms theory for the stress and strain in the bulk that is induced by surface stresses in elongated nanoscale objects.^303,353^

Lastly, high-resolution STEM revealed inward or outward relaxation of the outermost atomic layer at the surfaces of ligaments.^337,352^ This feature exemplifies a relaxation at both clean^354^ and adsorbate-covered surfaces^355^ that has been well established in studies of single crystals.

To summarize, the following statements can be made about the distribution of lattice parameters in NPG. The mean value does not significantly deviate from bulk Au, yet the standard deviation can be quite wide. That implies that regions of increased as well as decreased lattice parameter can be found in both the bulk and the surface of NPG.

Besides the strain state of pristine NPG, there are first hints that restructuring of the samples in catalysis experiments also changes the strain state.^356^ The TEM images used for that analysis are not ideal with respect to defocus and thus delocalization problems, yet they provide compelling evidence for the generation of lattice defects in operando. In principle, the link between microstructure and stress in NPG might be exploited for deliberately tailoring the strain state at the pore surfaces, thereby tuning the catalytic activity. This “strain engineering” of NPG is a field to be explored in the future.

### 3.13 Chemical Heterogeneity

The chemical composition is another important metric for characterization of NPG. Although the majority of the LNE is removed during dealloying, a significant atom fraction of residual LNE is always retained. As outlined in section 2, the net amount of retained LNE can be controlled in a wide range by proper choice of the dealloying conditions. That is of relevance because, as will be discussed below in sections 4.1, 5.3, and 6.1, the residual LNE has important consequences for the catalytic activity. Mechanical properties may also depend on the LNE content, either indirectly through the solid fraction (which is higher if not all LNE has been removed),^322^ or directly through the effect of the composition on the local strength of the ligaments.^357^

The impact of the residual LNE on the materials behavior, for instance for catalysis and mechanics, depends on the spatial distribution of that component. For instance, catalytic reactions or the nucleation of lattice dislocations in mechanics will depend on the local concentration of LNE at the surface. It is therefore of relevance that the LNE is distributed inhomogeneously within NPG. Typically, the overall content of the LNE is determined by energy-dispersive X-ray analysis (EDX), which probes a layer of about 1 μm thickness, i.e., several times the diameter of typical ligaments. Within a ligament the concentration of the LNE changes by surface segregation^190,358^ during storage or during (electro)chemical reactions. XPS with a conventional Al K_α_ source probes a layer thickness of 4.4 nm limited by the inelastic mean free path length of the photoelectrons in NPG. A single number, namely the net LNE atom fraction, is then not sufficient to describe the impact of the extra component on the material’s behavior. More surface sensitive determination of the LNE requires excitation with soft X-rays.^190^

Several publications have pointed toward a macroscale heterogeneity in the LNE distribution. That heterogeneity takes the form of a skin layer with a thickness <100 nm at the macroscopic outer surface of NPG samples. As compared to the bulk, the surface layer exhibits reduced pore size (section 3.8) and an enhanced LNE content.^34,92,173^

**Figure 12:**
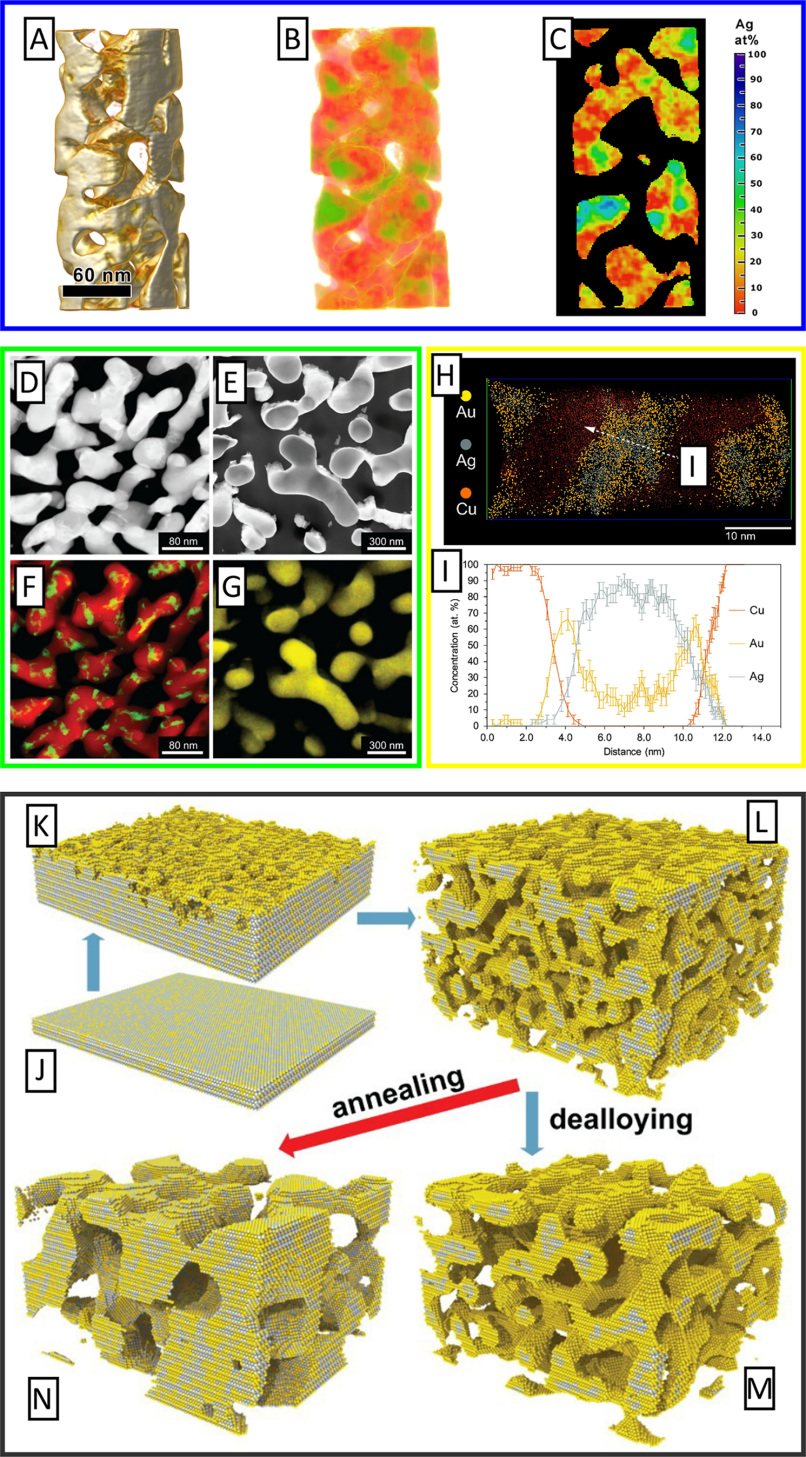
(A) STEM tomography reconstruction of NPG. (B) Quantitative 3D elemental distribution. (C) Slice cut out of the reconstruction in (B), revealing a Ag mole fraction up to 70 at. %, close to the composition of the master alloy. (D,F) STEM image and elemental map of a pristine NPG sample. (E,G) STEM image and elemental map of NPG after annealing showing a homogenized distribution of elements. (H) APT reconstruction of NPG. (I) Line profile along the line indicated in (H), showing higher Au content at the surface of a ligament. (J–N) kinetic Monte Carlo simulations of dealloying and annealing. (J) Top-view of surface of model sample. (K) Surface roughening during dealloying. (L) Initial ligament network after primary dealloying. (M) Coarsened ligament network after secondary dealloying. (N) Coarsened and homogenized network after annealing without corrosion. (A–C) Reproduced with permission from ref (93). Copyright 2017 Elsevier. (D–G, J–N) Reproduced with permission with permission from ref (89). Copyright 2017 The Authors under License CC. (H–I) Reproduced with permission from ref (94). Copyright 2017 Elsevier.

As has been discussed in section 2, the nanostructure formation during dealloying rests inherently on a competition between the trend for corrosion to passivate the alloy surface by enriching it in the MNE and a trend for the nucleation and growth of nanoscale pores that consume the pristine master alloy below the passive layer. It is then not surprising that the nanoporous product structure of dealloying exhibits heterogeneities in composition at the nanoscale, including regions that are practically pure MNE, yet also regions that still retain the LNE-rich master alloy. Indeed, element-resolved 2D micrographs show nonuniform element distribution in NPG made from Ag–Au^359^ and from Cu–Au.^155,281,360^ Nature and origin of these features could only be unraveled with the advent of composition-resolved 3D nanotomographic reconstruction. Using analytical transmission electron microscopy or the tomographic atom probe, three independent studies of NPG made from Ag–Au revealed (Figure 12A–I) that Ag in the porous material is typically localized in mostly buried Ag-rich regions that are embedded in a matrix of essentially pure Au.^89,93,94^ KMC simulations naturally reproduce that microstructure, revealing that the Ag-rich regions are relics of the master alloy that have never been exposed to the corrosive environment (Figure 12J–N).^89,90^ This is consistent with passivation, in other words, with an at least one atom thick layer of pure Au covering the Ag-rich regions. Experiment confirms that the Ag content within those latter regions is not exceeding that of the master alloy.^93^

KMC simulations reveal that the characteristic size of the Ag-rich regions scale with the initial ligament size that is generated during primary dealloying, prior to coarsening.^89,90^ As the regions are buried, and as bulk diffusion in Ag–Au is negligible near room temperature,^79,91,361^ the regions cannot grow when the microstructure coarsens during secondary dealloying. In principle, this offers the opportunity of studying their size experimentally as a basis for unraveling the initial structure size of the nanostructure formation during primary dealloying, right at the corrosion front.

The present understanding of dealloying suggests a strong trend for passivation in the form of an MNE-rich ligament surface during dealloying. This is confirmed by tomographic atom probe data^94,362^ and by KMC simulation.^88−90^ Yet, once the dealloying process is stopped, LNE that was originally buried in the bulk of ligaments may accumulate at their surface. The underlying processes may be surface migration during coarsening by surface diffusion or surface reconstruction driven by changes in the environment. A trend for surface segregation may contribute as a driving force. During the coarsening of NPG (see section 2.2), the surface migrates and sweeps the crystal lattice. As a consequence, LNE-rich regions that were originally buried underneath the surface are exposed. Their LNE content can then be redistributed by surface diffusion. In this way, the bulk composition is homogenized even though bulk diffusion is inactive. LNE-rich regions connected to the surface have indeed been identified in experiment,^93^ bulk-homogenization during coarsening has been confirmed by experiment and surface diffusion has been identified as the active process by simulation.^89^

Reconstruction is well documented for Au surfaces and is known to react to changes in the environment.^363^ Not surprisingly, the surface of NPG has been found to reorder and/or migrate during operation in catalysis^23,57,190,359,364^ or during potential cycles in electrochemistry^107,121,339^ (see also sections 3.11, 4.4, and 6.1.2).

This brief overview shows that a complete characterization of the LNE distribution requires that the average LNE content, an LNE content profile (depending on the sample geometry), and an analysis of LNE-rich regions (size and composition) are all considered. As such measurements consume time and experimental resources, reasonable trade-offs must be found for the number of samples investigated. For catalysis, immediate interest is on the composition right at the pore surfaces. Yet, the composition field throughout the material may be of importance for the stability and for the evolution of the material under operating conditions.

### 3.14 3D Representations

It is readily estimated that 1 mm^3^ of NPG with 10 nm ligament size contains around 10^14^ ligaments,^177^ and this represents an extreme structural complexity. Most of the microstructure metrics discussed in the previous sections condense individual, relevant aspects of this complex geometry into single scalar parameters. The abstraction provided by this strategy is indispensable as a basis for identifying and understanding microstructure–property relationships and for comparing and assessing results from separate studies. Yet, some situations require more complex representations of the microstructure. This applies specifically to numerical studies exploring transport behavior,^246,365,366^ wave propagation,^367^ optical properties,^214,368^ or the mechanics of porous solids and specifically NPG. Volume elements containing a representative sample of the 3D microstructure (representative volume elements, RVEs) of a stochastic material for those purposes can be generated in various ways, as recently reviewed.^369^

Some studies in the area of NPG directly adopt experimental 3D reconstructions as their RVEs,^271,289,370^ and approaches based more generally on experimentally informed RVEs have been reviewed.^268^ The major asset of this experiment-based pathway is that it warrants a realistic geometry. Yet, there are also drawbacks—conceivable artifacts from the reconstruction need to be considered, periodic boundary conditions do not apply, and it is not typically possible to vary microstructural parameters.

Overcoming the limitations of experimental reconstructions requires models—more or less realistic abstractions matching selected aspects of the real geometry—for generating 3D representations of the NPG microstructure. A prominent example is the truss structure by Gibson and Ashby,^371^ which successfully describes the mechanical behavior of a surprisingly wide variety of open-cell foams in nature and technology^372^ and which provides benchmarks for the mechanics of NPG.^26,141,168,170^ This structure takes the form of a crystalline array of nodes, connected by struts. It exemplifies periodic models for porous materials. Periodic network models based on other crystal structures, for instance simple cubic^214,370^ or diamond,^373^ have also been considered. The stochastic nature of NPG can partly be accounted for when random displacements of the nodes are incorporated,^373^ or when struts are randomly cut.^374^ Other periodic models for NPG adopt minimal surface geometries, such as the gyroid structure.^271,312,370^

Periodic models are at odds with the stochastic nature of the NPG microstructure and with its statistical isotropy. Models representing the pore structure by random arrays of spherical voids in a solid matrix^353,368,375^ remedy that problem, but their geometry conflicts with the network character of NPG. Furthermore, along with all of the aforementioned approaches, they do not naturally reflect the systematic variation of NPG’s connectivity with the solid fraction. In those respects, spinodal structures are closer to NPG. Several modeling studies of NPG have generated spinodal-like RVEs by implementing spinodal decomposition numerically in Monte Carlo^128,274^ or phase field^271−273,376^ simulation. The resulting geometries appear quite close to NPG. Yet, spinodal structures can lose connectivity when allowed to enter the “late stage” (coarsening) regime,^377,123^ and that regime is readily encountered in simulations of spinodal decomposition. Indeed, studies modeling NPG with solid fractions near 0.3 by numerically generated spinodal structures report effective Young’s moduli that vary more than 10-fold, ranging from 0.004^271^ and 0.007^274^ to 0.05^273,376^ of the bulk value. In view of the strong correlation between effective elasticity and connectivity (section 3.9), the discrepancy confirms that the topology of numerically generated spinodal structures is not unique.

The observations above emphasize the relevance of the leveled-wave model (section 3.2). That model approximates early stage spinodal decomposition, avoiding the extra degrees of freedom for disconnection that come with coarsening during the later stage. As compared to other strategies toward 3D representations of NPG, the leveled-wave model is distinguished by its uniqueness and by its ability to reproduce, with no free parameters, several decisive properties of NPG, specifically (1) the systematic variation of the connectivity with solid fraction, (2) the isotropic macroscopic materials properties, and (3) the presence of a well-defined dominant wavelength. Lastly, as outlined in section 3.2, the microstructure of the model results from energetics and kinetics that act in closely similar form in the real material. In that sense, the model appears particularly suitable as a basis for guiding the intuition toward new insights into the fundamentals of NPG.

## 4 Gas Phase Catalysis

In 2006, NPG was established as an active catalyst for low-temperature aerobic oxidation reactions as demonstrated for CO oxidation.^5,6^ While oxide-supported Au nanoparticles (NPs) had been in the focus of gold catalysis since the 1980s, starting with the work by Haruta^1^ on CO oxidation and Hutchings^378^ on the hydrochlorination of acetylene, the high activity found for NPG at room temperature and even below was quite unexpected at that time. This was due to the large structural differences between NPG being a (bi)continuous metal structure with ligament sizes in the range of several 10 nm and Au nanoparticle catalysts, which were found to be catalytically active only for a narrow range of particle sizes around 5 nm and only in the presence of particular (reducible) oxide supports.

Shortly afterward, it turned out that the material is not only a good catalyst for total but also for partial oxidation reactions. The latter ability was first exemplified for aerobic methanol oxidation, yielding methyl formate at high selectivity (at not too high oxygen partial pressures).^7^ This finding initiated a wealth of further experimental as well as theoretical studies in the following years, which widened the knowledge regarding the catalytic scope of NPG considerably. Apart from other oxidation reactions, such as preferential CO oxidation (PROX) or the oxidative coupling of alcohols and amines to the corresponding amides, it was demonstrated that even reductions are feasible, and this oftentimes in gas and liquid phase. To this end, it is important to note that some of these reactions require modifications of NPG, e.g., by introduction of oxide particles deposited onto the ligaments of NPG or the use of NPG systems with a different LNE than Ag.^58,379−381^

The interest in the material was also driven by the perception that NPG is structurally and chemically less complex than oxide-supported Au nanoparticles. This perception was corroborated already by the first reports of NPG’s partial oxidation capabilities demonstrated at the example of methanol conversion to methyl formate, which could be explained by insights from previous surface science studies on Au single crystals.^7,382^ Hence, NPG may open a way to potentially address the catalytic properties of Au more directly than it was possible for Au nanoparticle catalysts. The notion that NPG is a metallic catalyst (lacking the complexity arising from a metal–oxide interface) renders a combination of catalytic studies on NPG with surface science experiments on single-crystal surfaces and computationally based methodologies a particularly promising strategy to attain a mechanistic understanding of heterogeneously catalyzed processes at the atomic level. This strategy is clearly not restricted to the above-mentioned partial oxidation reaction but can also be applied to total oxidation reactions such as CO oxidation. In this realm, it could, e.g., provide insights to the question, which oxygen species or oxide surface phases are responsible for the efficient catalytic turnover.^45,55^ The strategy has been successfully used over the past decade to obtain insight into several catalytically interesting reactions, and several reviews have summarized these efforts already.^71,383−386^

Nevertheless, it is important to note that the current mechanistic understanding of the aerobic oxidation chemistry on NPG is still fragmentary as far as some important aspects are concerned. One question, which immediately came up when the first reports regarding NPG’s catalytic potential were published, relates to the mechanism of O_2_ activation. Notably, the binding of molecular oxygen is very weak on Au surfaces and the probability for its subsequent dissociation is negligible even at pressures of several mbar, where the catalytic experiments with NPG were carried out. Accordingly, its ability to catalyze aerobic oxidation reactions cannot be explained on the basis of the Au surface chemistry alone. In agreement with that, experimental as well as theoretical insight gained over the last years indicate that oxygen activation indeed does not occur on Au sites but that residual amounts of the less noble metal (Ag for most of the catalytic studies on NPG) are likely to be responsible for this step. At the same time, a comparison with Ag, exhibiting a distinctly different oxidation chemistry, suggests that all subsequent reaction steps take place on Au surface sites and not on Ag sites.^45,382,387,388^ As far as the chemical nature of the oxygen species, which are involved and determine NPG’s selectivity for partial or total oxidation reactions is concerned, many questions, however, remained unsolved so far. The selective formation of coupling products (esters) observed for alcohol oxidation, for instance, was primarily explained in the literature on the basis of chemisorbed O atoms and their Brønstedt basicity, enabling the necessary H-abstraction steps.^7,383^ Such a perspective, however, neither takes sufficiently into account the wealth of oxygen species or surface oxide phases, theoretically predicted and also partly observed in Au single-crystal studies, nor their Lewis basicity (nucleophilicity), i.e., the ability for oxygen transfer steps and formation of total oxidation products. Even though a number of experimental findings could be interpreted on this basis, for a more differentiated mechanistic picture, a detailed view on the potential range of oxygen species and associated activation and reaction pathways on the surface is mandatory.

Aiming at complementing other reviews about NPG in this respect, we have thus chosen a corresponding focus in this chapter.^16,38,40,71,389^ The current state of knowledge will be summarized at the examples of CO and methanol oxidation, representing the most intensively studied examples of total and partial oxidation reactions catalyzed by NPG. In section 4.1, we dwell on corresponding insight from theory, whereas section 4.2 provides an overview over the most important results from UHV single-crystal studies. Section 4.3 elucidates the connection of these fields to the catalytic behavior of NPG (in the gas phase), and finally section 4.4 addresses the structural evolution of NPG during catalytic reactions.

### 4.1 Surface Composition, Active Sites, and Reaction Mechanisms from Computational Studies

#### 4.1.1 Aerobic Oxidation Reactions and O_2_ Activation

The ability to catalyze aerobic oxidation makes nanoporous gold attractive in the context of green chemistry and sustainable development. Molecular oxygen (or ideally air) is an inexpensive, abundant, and environmentally friendly oxidant. The activation of a dioxygen molecule is known to be central to all aerobic reactions. Therefore, this important reaction step will also be one of the reoccurring themes throughout this subsection, where current understanding of the mechanistic aspects of the catalytic activity (including open questions and opposing viewpoints) will be reviewed.

The activation of the adsorbed O_2_ molecule on Au is believed to be a difficult step in aerobic oxidation reactions catalyzed by the material. Here, “activation” subsumes all chemical transformations leading to reactive oxygen species. This may either be the O–O bond cleavage, producing two adsorbed O atoms (with a formal charge state O^2–^), or nondissociative pathways. Options of the latter kind are connected with O_2_^–^ or O_2_^2–^ surface species which exhibit a weakened O–O bond, resulting from a charge transfer from the metal to the adsorbate. Moreover, addition/abstraction reactions between O_2_ and surface species formed after adsorption of the other reactant are conceivable, which then leads to adsorbed peroxide derivatives, such as OCOO^2–^ or HOO^–^. (In the following, we will omit charges because such an assignment represents only one formal way to describe the scenario, based on the idea of a complete charge transfer so that the Au surface atoms and the adsorbate carry opposite charges of the same magnitude. An alternative perspective which is widely accepted by many scholars emanates from (uncharged) radicals, keeping in mind that the unpaired electrons of the adsorbate atoms are in fact involved in the bonds with the metal atoms at the surface.)

As noted in many prior reviews,^16,38,41,383,386,388,390−394^ not only the activation barrier for O_2_ dissociation is very high on extended Au surfaces, (>1 eV according to theoretical estimates)^50,52,53,395,396^ but, and more importantly, also the adsorption of O_2_ is very weak. Calculated adsorption energies range between −0.28 and −0.14 eV on various stepped Au surfaces,^50,53,396,397^ while no adsorption occurs on Au(111).^52,395^ Adsorption energies of comparable magnitude were also found for lattice defects exposing low-coordinated surface sites such as twin boundaries which exhibit 6-fold coordinated surface sites.^398,399^

Based on these results, O_2_ activation is unlikely to take place on Au surface sites during aerobic oxidation reactions catalyzed by NPG. Instead, there has been growing evidence over the past decade that residual impurities of the LNE (typically Ag) are of key importance for this step.^50−52,54,397,400^ Other structural features of NPG which are potentially important for binding and activating O_2_ are the high abundance of low-coordinated atoms on its ligaments and the occurrence of crystallographic strain, dislocations, and preexisting atomic oxygen species.^50,53,337,359,398,399^ Once surface atomic O is formed on nanoporous gold through one of the mechanisms outlined below, it can easily react with various adsorbates, resulting in partial or total oxidation products. Notably, chemically different types of adsorbed oxygen exhibiting different reactivity appear to exist on both single-crystalline Au surfaces and NPG, as will be discussed in more detail in sections 4.2 and 4.3.

While the way how dioxygen is activated and supplied seems to be characteristic of NPG and its special features, the remaining reaction steps (determining the catalytic behavior with respect to total or partial oxidation reactions) are in general agreement with the surface chemistry observed on well-defined single-crystal Au surfaces under UHV conditions when atomic O was preadsorbed.^44,47,401−403^ Specifically, it has been frequently emphasized in the literature that the selective oxidation chemistry of NPG corresponds to gold rather than to silver,^7,16,38,392,404^ a conclusion drawn on the basis of insight gained from UHV single-crystal studies regarding the specific and fundamental differences of Au and Ag surface chemistry.^44,46,382^

In spite of extensive research efforts, the detailed molecular-level mechanism of O_2_ activation on NPG remains a matter of debate. Although experimental studies provide evidence that NPG is able to dissociate O_2_,^405,406^ these studies provide no insight into the reaction mechanism. In turn, it is not known if adsorbed O_2_ reacts with surface species or dissociates directly into adsorbed O atoms. The dominant mechanism may depend on the specific reaction conditions. Moreover, the different resulting active oxygen species may impact the catalytic properties of NPG such as activity or the selectivity of partial oxidation reactions.

**Figure 13:**
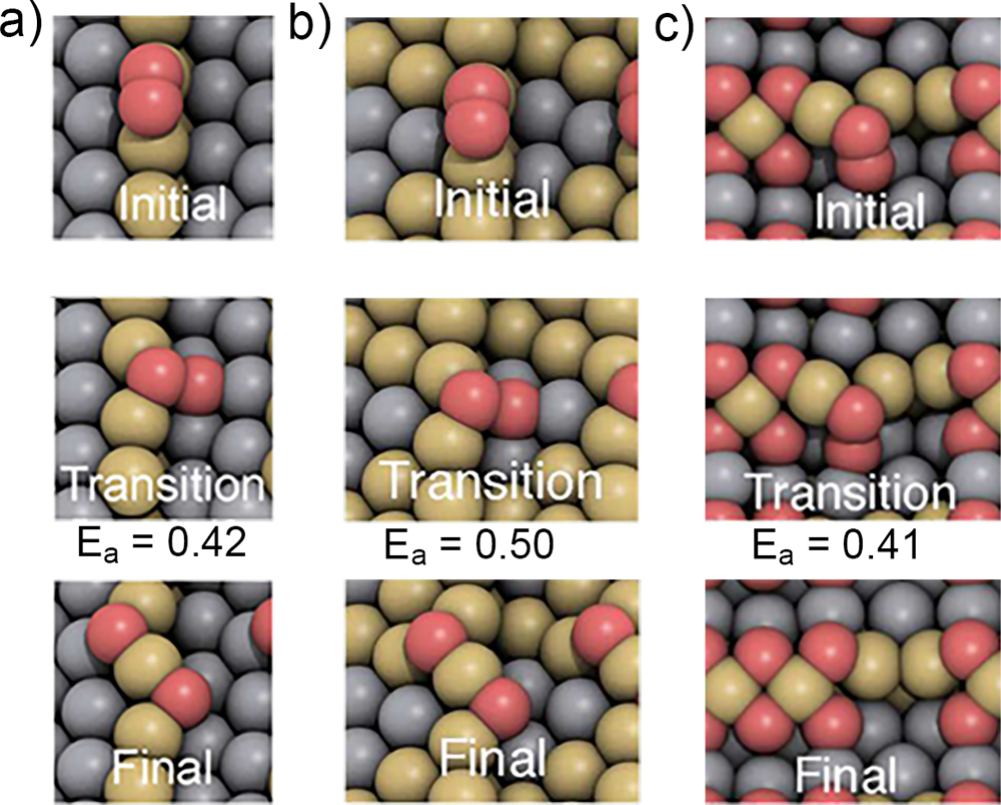
Activation energy (in eV) and transition state geometry for O_2_ dissociation on a AgAu(211) stepped surface (a) with Ag atoms in the rows next to the step Au atoms, (b) with a three-atom Ag ensemble, and (c) same as (a) but with coadsorbed O, corresponding to four O vacancies. Color coding: Au, yellow; Ag, gray; O, red. Adapted from ref (53). Copyright 2016 American Chemical Society.

**Figure 14:**
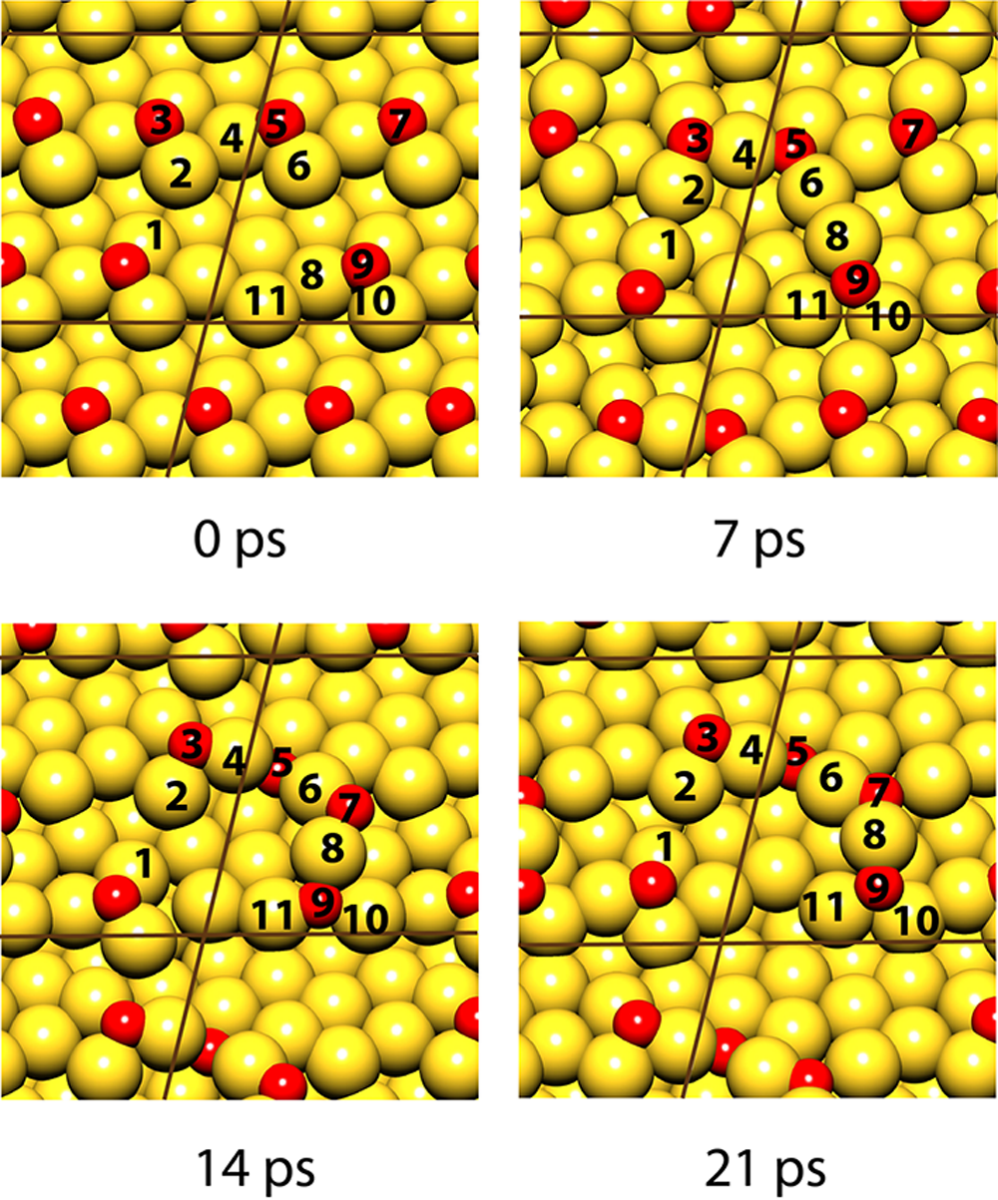
Snapshots of an AIMD simulation showing −(Au–O)– chain formation from individually adsorbed O atoms on Au(321) without Ag impurities. (3×2) unit cell and O coverage of 0.17 ML. Color coding: Au, yellow; O, red. Reproduced with permission from ref (411). Copyright 2018 American Chemical Society.

Whereas a large body of experimental literature assumed that O_2_ dissociates into O atoms at the active sites of NPG (presumably Ag or bimetallic sites),^16,38,41,383,386,388,390−394^ the relatively high activation barrier for O_2_ dissociation typically found in computational studies does not readily fit into this picture.^50,52,53,395,396^ In this respect, it is important to note that high activation barriers are not limited to low-index surfaces with coordinatively saturated Au atoms but are also found for stepped surfaces with low-coordinated sites. In case of bimetallic Au–Ag sites, expected to be present on the surface of NPG prepared from Au–Ag master alloys, activation energies in the range of 0.4–0.7 eV were calculated. Even though these barriers are lower than found on pure Au surfaces, these values are still too high to explain the high activity of NPG observed at room temperature and even below.^50,52,53,407^ The lowest activation energies for O_2_ dissociation (0.4–0.5 eV) were calculated on bimetallic Au–Ag sites on a stepped AgAu(211) surface for which Au atoms at the steps were surrounded by ensembles of three or more Ag atoms located on the surface and at subsurface positions (Figure 13a,b).^53^ In one of the considered structures, monoatomic Au chains were decorated by adsorbed O atoms so that a chain-like 1D “gold oxide” consisting of repeated squared AuO_4_ structural fragments is formed. O_2_ dissociation taking place at a vacancy site (Figure 13c) was connected with a barrier of only 0.4 eV. The authors also demonstrated from first-principles thermodynamics that such arrangements of Au–Ag were more favorable than others. Another study found a slightly higher barrier for O_2_ dissociation of 0.56 eV on a AgAu(110) surface, where Au was occupying low-coordinated “comb” sites, again forming monatomic Au chains, surrounded by Ag in subsurface (“valley”) positions.^52^ The presence of monatomic Au chains surrounded by Ag is a structural similarity between the latter structure as shown in Figure 13a. Another similarity results from the observation that the dissociating O atoms form a linear O–Au–O fragment. Such a fragment represents the shortest possible O–Au–O chain and is a structural element of a 1D Au oxide. One- and two-dimensional (branched) −(O–Au)– chains were identified in several theoretical studies as thermodynamically most stable forms of adsorbed oxygen on various Au surfaces.^50,53,408−410^ As shown in Figure 14, it was predicted theoretically that surface O atoms on a stepped Au surface tend to self-organize into chain structures with O–Au–O structural motifs.^411^ On a bimetallic Ag–Au surface, formation of such oxygen chains is predicted to be accompanied by surface and subsurface diffusion of Ag atoms which tend to segregate near the −(O–Au)– chains, further stabilizing them.^364,411^

**Scheme 1:**
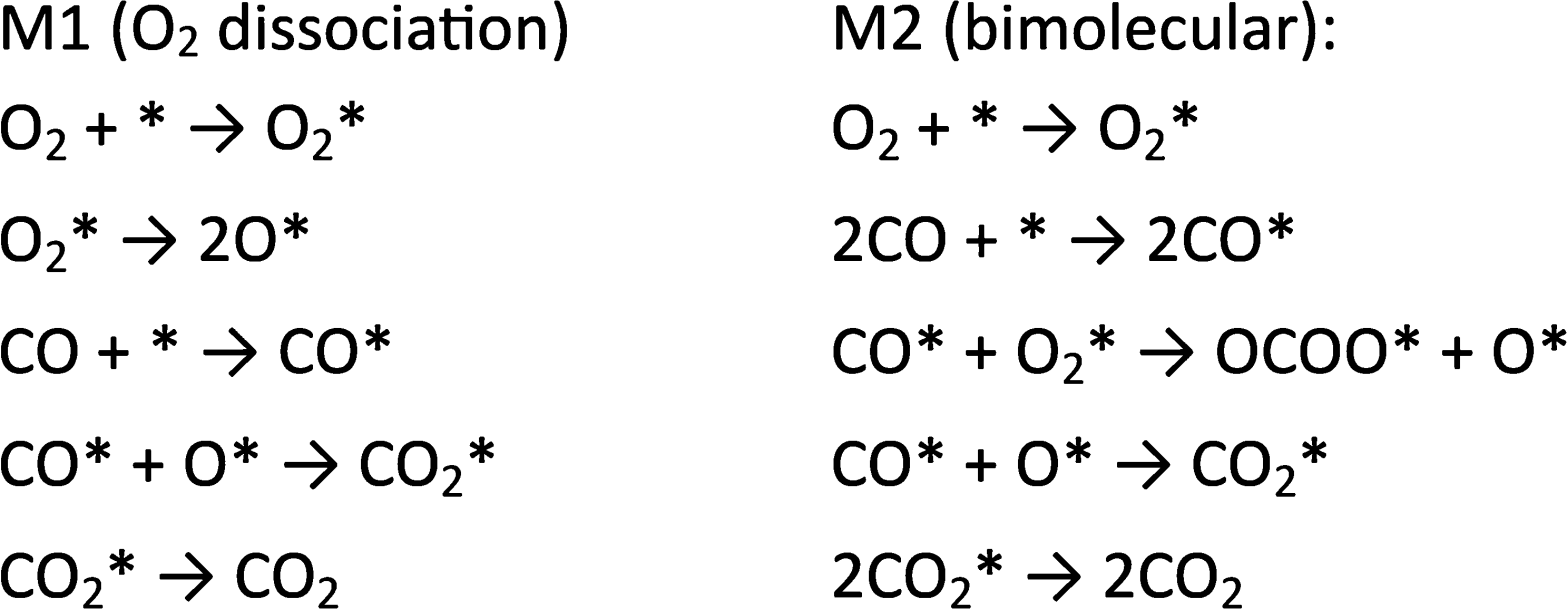
Dissociative and Associative (Bimolecular) Mechanisms Discussed for CO Oxidation

In the case of CO oxidation, computational studies have explicitly considered a bimolecular mechanism, in which adsorbed O_2_ (from here on adsorbed species are denoted by *, O_2_*) directly reacts with CO* on Au, Ag, or bimetallic surface sites and identified pathways with lower barriers than for the dissociation of O_2_.^54,397−399,412,413^ The set of elementary reactions involved in the two mechanisms (O_2_ dissociation (M1) and bimolecular reaction (M2)) is given in Scheme 1 for oxidation of CO.

**Figure 15:**
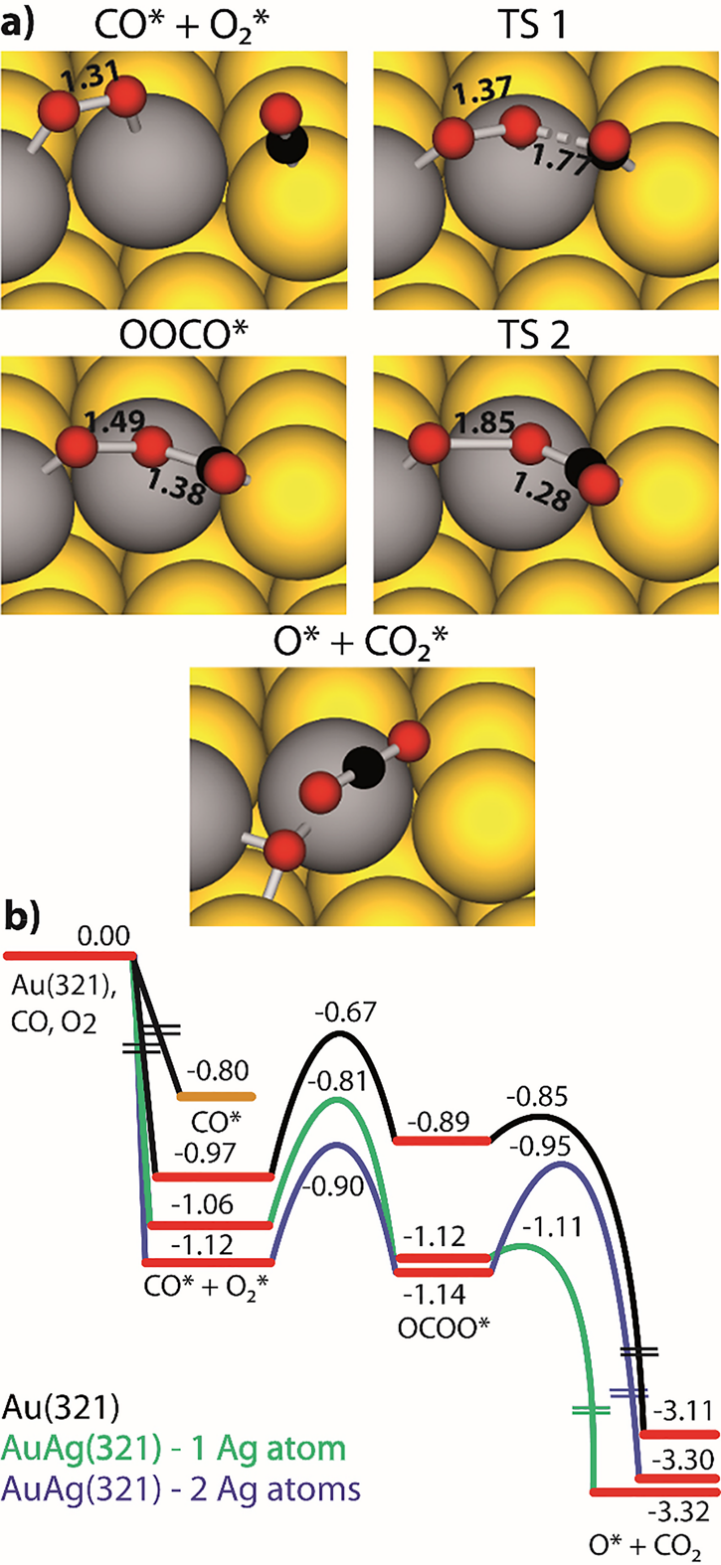
Direct reaction of CO* with molecular oxygen. (a) Top view of reactants, products, OCOO* intermediate, and transition states on Au(321) with Ag impurities. Selected bond distances are given in Å. (b) Reaction energy profiles in eV. Energies are given with respect to the CO and O_2_ molecules in the gas phase and Au(321) with or without Ag impurities. The horizontal bars denote axis breaks. The adsorption energies of CO* on pure Au(321) and bimetallic surfaces with one and two Ag atoms at the reaction site are −0.80, −0.76, and −0.79 eV, respectively. Color coding: Au, yellow; Ag, gray; O, red; C, black. Adapted with permission from ref (54). Copyright 2019 American Chemical Society.

Figure 15 exemplifies the role of Ag in CO oxidation following the bimolecular reaction mechanism for Au(321), which exhibits different low-coordinated surface sites. As seen from Figure 15b, impurities strengthen the rather weak O_2_ adsorption on 6-fold coordinated Au sites. However, this increase in O_2_ binding energy is associated with an increase of the reaction barrier of the subsequent association step, rendering also this mechanism limited by O_2_ adsorption.

Fajín et al.^414^ performed KMC simulations of the reaction rates of CO oxidation (CO + O_2_) on pure Au(110) and Ag(110) and found no reaction on Au(110), whereas the theoretically estimated TOF on Ag(110) was comparable to experimentally reported values on NPG. That allowed the authors^414^ to propose that patches of pure Ag could be active sites for O_2_ dissociation on NPG. Another theoretical study performed microkinetic modeling of CO oxidation on Au(321), Ag(321), as well as on AgAu(321) bimetallic surfaces and found similarly negligible activity on pure Au(321) but much higher activity on Ag(321), whereas intermediate activity was observed on bimetallic AgAu(321) surfaces with varied concentrations of Ag.^54^

Somewhat different models of stepped surfaces based on Au(111) and Au(100) slabs where some metal atoms were removed to create channel-like depressions were employed in another computational work.^397^ These depressions exhibited a stepped profile exposing infinite rows of low-coordinated Au atoms. In some of the considered models, a varied amount of Ag impurity atoms was added to replace Au atoms at the reaction site. The study reported an increasing binding strength of O_2_* (more negative adsorption energy) with increasing amounts of Ag impurity atoms at the adsorption site, in agreement with the findings on Au(321).^54^ Concomitantly, higher reaction rates were predicted (using a microkinetic model) on bimetallic surfaces as compared to pure gold surfaces. In an extension to the bimolecular mechanism described above, the attack of the OCOO* intermediate by a second coadsorbed CO* molecule was found to lower the barrier for its dissociation.^397^ Hence, instead of one CO_2_ molecule resulting from a reaction of a single CO* molecule with an O_2_* molecule, two CO_2_ molecules were formed with a low activation barrier. Assuming this mechanism, microkinetic modeling predicted higher rates of CO oxidation compared to a conventional bimolecular mechanism, especially on pure gold and on some of the Ag substituted models.

To summarize, theoretical studies predict the adsorption of O_2_ on Ag or bimetallic sites of NPG. Adsorption on Au sites is found to be too weak to result in a reaction. Several mechanisms of O_2_ activation have been discussed. Most mechanistic studies focus on a dissociative pathway with oxygen atoms as the reactive species. In the case of CO oxidation, the computational results suggest that a bimolecular mechanism, in which CO* reacts with O_2_*, is the more likely pathway, at least at low temperatures because of considerably lower activation barriers associated with the bimolecular mechanism as compared to O_2_ dissociation.

#### 4.1.2 Water as a Co-catalyst in CO and Alcohol Oxidation

A possible beneficial role of water in cocatalyzing CO oxidation was reported in catalytic studies on supported Au nanoparticles,^415−417^ and a similar rate enhancement by moisture was also experimentally found for CO oxidation on NPG.^390^ Even though the underlying mechanistic details are likely to be different, and related to the oxide support (formation of surface OH groups) in the former case, it is interesting to understand the effect of water on the reactivity of both types of Au catalysts. Notably, moisture could either be inadvertently present in reaction mixtures (as an impurity) or water may be a byproduct of the catalytic transformation as in the partial oxidation of organic compounds (such as methanol oxidation). It has been suggested that water could facilitate O_2_* activation by converting O_2_* to surface hydroperoxyl (OOH*).^416,417^ However, other ways by which water may be involved have also been proposed and investigated. The proposed roles of water in enhancing catalytic activity of supported Au nanoparticles were comprehensively summarized and classified in four categories:^418^ “(i) maintain cationic state of gold (Au^3+^ or Au^+^), (ii) direct involvement of H_2_O and OH-groups in CO oxidation, (iii) activation of O_2_ molecules, and (iv) transformation of catalytic intermediates and inhibitors (spectators) such as carbonate species”.

Computational studies played a crucial role in addressing possible mechanistic scenarios of how either water (an impurity or byproduct) or an alcohol molecule itself (in the context of partial alcohol oxidation) may help to activate O_2_*. The latter aspect is related to an important question: What nondissociative mechanistic pathways may exist for O_2_ activation that are based on an involvement or assistance of the reductant molecule itself? A formation of OOH* as a result of an abstraction of a hydrogen atom from water or an α-H from the OH-group of an alcohol molecule by O_2_* (note the analogy) was proposed in a computational study about methanol oxidation on Au(111):^395^
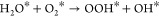
4
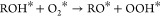
5

Several pathways involving these reactions were considered.^395^ For a simple hydrogen transfer from water or from methanol, quite large barriers of 0.84 and 0.91 eV, respectively, were calculated. However, the authors also found lower-energy transition states involving a mediator H_2_O or methanol molecule connected to the reactants via a chain of H bonds that facilitated H transfer to O_2_* significantly lowering the barriers to about 0.45 eV. In the next step, OOH* is expected to dissociate to OH* and O*, but quite a large barrier of 0.79 eV was calculated for this process. Therefore, it was suggested that the OOH* intermediates themselves might be acting as oxidizing agents rather than their dissociation products OH* and O*. To this end, a surprisingly small barrier of only 0.3 eV was calculated in that work for a transfer of an α-H from the OH-group of methanol to a surface OOH* species resulting in a methoxy (CH_3_O*) species and H_2_O_2_*. Nevertheless, this activation barrier is larger than the values of 0.13 and 0.08 eV, as calculated in the same work, for the analogous α-H abstractions by O* or OH*, respectively.

Generally, it seems that a high concentration of methoxy on the surface helps converting all formaldehyde that is formed in the subsequent dehydrogenation step to the partial oxidation product methyl formate, most of which desorbs without reacting further. This is possible thanks to low concentrations of the strongly oxidizing surface species (O* and OH*), which otherwise would drive overoxidation to the total oxidation product CO_2_. In conjunction with relatively low diffusion barriers for adsorbates on Au surfaces, these factors appear to be the basis of the high selectivity of aerobic methanol oxidation toward partial oxidation products, as discussed in the following.

To further investigate nondissociative routes of O_2_ activation discussed above, a Au–Ag(111) surface model was considered, where Ag impurities were introduced as Ag dimers substituting two adjacent Au atoms.^419^ Much lower activation barriers were found for the formation and dissociation of the OOH* species^419^ than calculated in a previous study for the same reaction steps on pure Au(111) without additional H_2_O molecules as mediators.^395^ The α-H-transfer from methanol to O_2_* proceeded with an activation energy of 0.5 eV and the dissociation to O* + OH* with a barrier of 0.45 eV. The α-H abstraction from the OH-group of methanol by OOH* revealed an activation barrier of 0.32 eV, very close to the result mentioned above.^395^ The hydrogen peroxide (H_2_O_2_*) formed in this process was found to easily dissociate yielding two OH* groups with a negligible activation barrier.

**Figure 16:**
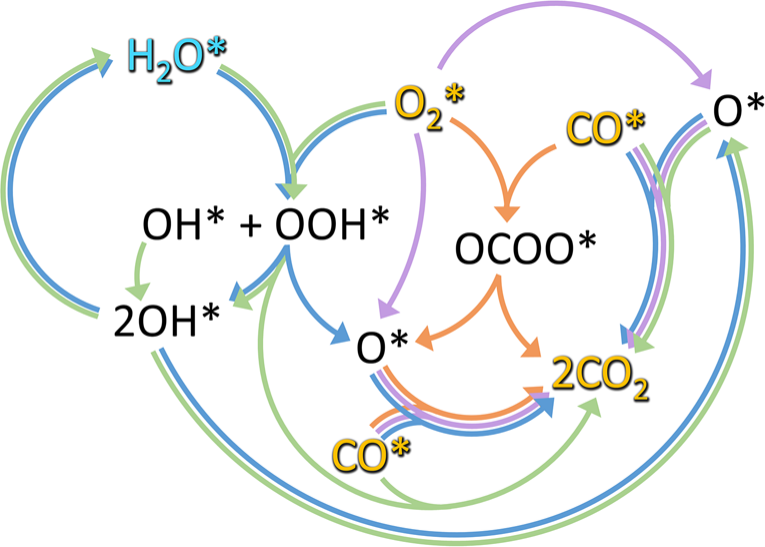
Possible pathways for CO* + O_2_* reaction over gold including those cocatalyzed with water.^54^ Reactants and products are in yellow, and water as cocatalyst is in blue. Arrow colors mark four different mechanisms: purple, dissociative mechanism; orange, associative mechanism; blue, water catalyzed mechanism via dissociation of OOH*; green, water catalyzed mechanism via direct reaction of OOH* with CO*.

Significantly smaller activation barriers than found on a Au(111) surface, all lying below 0.4 eV, were reported for the formation of OOH* from O_2_* and adsorbed water or methanol and its dissociation to O* and OH* on a stepped Au(310) surface.^396^ Likewise, comparatively low barriers were calculated for the H transfer to O_2_* from water and for OOH* dissociation on a stepped Au(321) surface, with or without Ag impurities.^54^Figure 16 shows a reaction network considered including the pathways involving water as a reactant. The first step (a H transfer from adsorbed water to O_2_*) exhibits a barrier of 0.30 eV on pure Au(321) which remains almost unchanged on Ag substituted surfaces, whereas the barriers for OOH* dissociation increase from 0.26 eV on pure Au(321) to 0.35–0.49 eV on Ag alloyed Ag–Au(321). Alternatively, OOH* can react directly with CO* to give CO_2_ + OH* with a barrier of 0.36 eV. Possibly, the lowering of activation barriers compared to the Au(111) model can be attributed to more flexible low-coordinated Au atoms at the step sites of Au(321) and Au(310) helping to stabilize the transition states. Despite rather low calculated barriers for water-assisted O_2_ activation on Au(321), microkinetic modeling predicted the bimolecular CO* + O_2_* mechanism via an OCOO* intermediate to be the fastest reaction route for CO oxidation. In this respect, theory could not explain conclusively (i.e., on the basis of the selected models and investigated mechanisms) why the CO oxidation–as found in the experiment–is accelerated by water.^54^ This inability of theory to provide evidence for the beneficial effect of water in CO oxidation catalysis on NPG may be related to the reaction network considered. To this end, an interesting idea was proposed in a recent computational work,^420^ where very fast pathway for CO oxidation on Au(100) was suggested in the context of PROX. It involves the formation of a hydrocarboxyl intermediate, OCOH*, from CO* and OH*. This species could react with O_2_* with a negligible barrier forming CO_2_ and OOH*. In combination with OOH* + CO* → CO_2_ + OH* this could provide a claimed very fast pathway for CO oxidation.^420^

#### 4.1.3 Detailed Mechanism of Aerobic Alcohol Oxidation from Computational Studies

**Figure 17:**
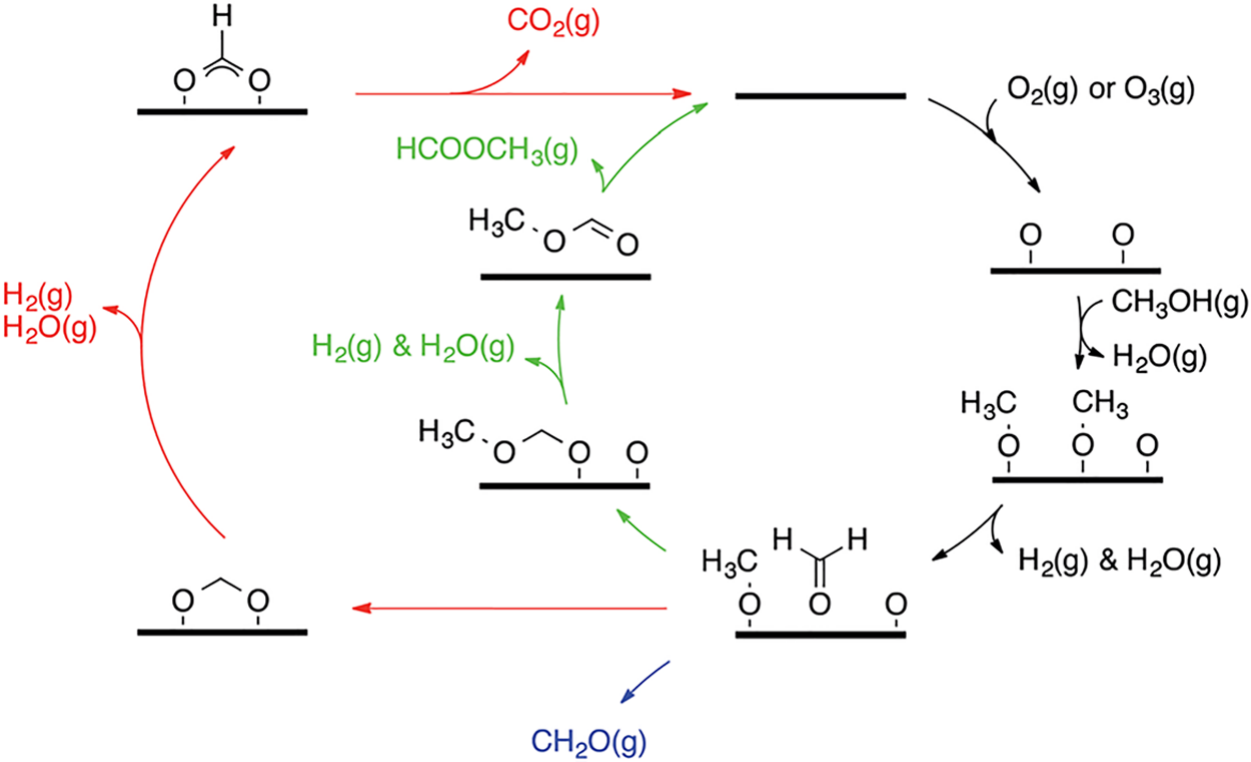
General mechanism for methanol oxidation over gold. The green arrows show the coupling route, while the red ones show the overoxidation route. Reproduced with permission from ref (421). Copyright 2021 The Authors.

While reviewing the proposed role of surface hydroperoxyl (OOH*) in O_2_ activation, we have already mentioned the potential importance of this intermediate in the context of alcohol oxidation. Despite the significantly lower activation barriers predicted for the methanol-assisted OOH* route (0.35–0.5 eV^54,396,419^) as compared to the O_2_* dissociation pathway on Au and even Ag–Au surfaces (0.45 to ∼1 eV,^50,52,53,407^), the former mechanism has not been given attention in the published experimental work on methanol oxidation so far. The standard mechanistic model used in many experimental studies emanated from the postulated O_2_* dissociation into (single) adsorbed O* atoms and their Brønsted basic properties.^44,47,382,401^ The corresponding scheme in Figure 17 is based on consecutive H-abstraction reactions. In this way, the conversion of methanol to methoxy and subsequently (by β-H abstraction) to formaldehyde is enabled. (The notations α and β are applied to the H atom of the OH and to the hydrogen atoms of the CH_3_ group, respectively.) Thus, the transformation sequence is CH_3_OH* → CH_3_O* → CH_2_O*. While surface O* atoms are mostly considered the key reactive intermediates, a mechanistic discussion restricted to this species might be an inappropriate simplification in view of the accumulated body of computational results reviewed above.

Along these lines, other oxygenated species, such as O_2_*, OH*, OOH*, or CH_3_O*, are likely to be important intermediates in the reaction network, as described in section 4.1.2 above. These species could exhibit sufficient (Brønsted) basicity as well—at least for the α-H abstraction converting methanol to methoxy—and some of them also for β-H abstraction from methoxy, yielding formaldehyde or could be otherwise involved in the corresponding elementary steps (e.g., OOH* may dissociate to strongly basic O* and OH*). Consequently, four possibilities have been considered for the formation of methoxy (CH_3_O*) in computational studies:
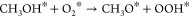
6
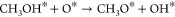
7
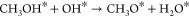
8
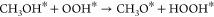
9

Reaction 6 is the most difficult one, with theoretically predicted activation barriers of 0.4–0.5 eV on extended Au surfaces (depending on the specific surface model used).^395,396,419^Reaction 9, assuming α-H atom abstraction from methanol by OOH*, is a bit more facile with a theoretical barrier <0.4 eV.^395,419^ For the remaining two reactions, reactions 7 and 8, however, DFT calculations yielded notably lower activation energies <0.27 eV.^395,419,421−423^ Most of the respective computations have been performed on a flat Au(111) model surface (or Au(111) alloyed with Ag) with the exception of two studies carried out for a stepped Au(310) surface^396^ and a stepped Au(997) surface.^424^

Further oxidation of methoxy to formaldehyde by O*, OH*, OOH*, or CH_3_O* species can then proceed according to the following reaction steps:
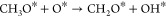
10
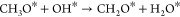
11
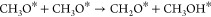
12
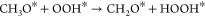
13

O_2_* has not been considered here as a potential β-H abstracting agent due to its expected low nucleophilicity and basicity. The first two reactions (CH_3_O* + O*/OH*) were predicted to proceed quite easily on Ag–Au(111), with activation barriers of only 0.27 and 0.45 eV, respectively.^419^ Even smaller values, 0.07 and 0.24 eV, were found for the same reactions on pure Au(111),^395^ whereas 0.14 eV was calculated in another study on Au(111) for CH_3_O* + O*.^422^ Notably, these kinetic barriers are lower than reported for the same reactions in three further computational studies of methanol oxidation on Au(111).^48,421,423^ These latter works computed barriers of 0.43–0.49 eV and 0.48–0.70 eV for β-H abstraction from methoxy by O* and OH*, respectively. A study on a stepped Au(310) surface, on the other hand, reported an activation energy of 0.48 eV for a reaction of CH_3_O* with O* but also found that the barrier increases to 0.65 eV if methoxy and O* are far away from each other in the initial state and are not interacting.^396^ The same tendency of increasing the barrier when reactants are placed far apart from each other was found in a study of methanol oxidation on Au(111).^421^ Here, the activation barrier for the β-H abstraction by O* increased from 0.43 to 0.56 eV for CH_3_O* + O* and from 0.70 to 0.79 eV for CH_3_O* + OH* when calculated with respect to noninteracting reactants. Two studies also considered β-H abstraction by OOH* and reported low barriers of 0.06 and 0.02 eV on Au(111)^395^ and Ag–Au(111),^419^ respectively.^44,382^ Nevertheless, one should keep in mind that under real catalytic conditions the concentrations of surface O*, OH*, as well as OOH* species are probably very low. Furthermore, as will be discussed below, OH* and O* species commonly assumed in the literature to be responsible for β-H abstraction from CH_3_O*, can as well be responsible for undesired deep oxidation routes. The observation that the latter routes are almost not followed on gold catalysts can be attributed to too low concentration of these reactive oxygenates on the surface under the corresponding reaction conditions. In contrast, CH_3_O* is likely to be the most abundant surface intermediate, which could abstract β-H from another methoxy species, acting as a base in analogy to OH* and O*. The activation barrier for this reaction was calculated to be 0.66 eV on Au(111).^48^ Hence, it is important to take this reaction channel for the CH_3_O* → CH_2_O* transformation into consideration under the conditions of ambient catalysis.

Once formaldehyde is formed, it can either desorb or undergo a barrierless reaction with either CH_3_O*, OH*, or O*, forming CH_3_OCH_2_O* (hemiacetal alkoxide), HOCH_2_O*, or OCH_2_O* (methane diolates), respectively. In all of these reactions, oxygenated species act as nucleophiles, while formaldehyde is an electrophile. As far as the latter pathway is concerned (CH_2_O* + O* → OCH_2_O*), an experimental study indicates that the formation of OCH_2_O* is reversible.^384^ The hemiacetal and diolate intermediates, resulting from the three addition reactions above, can quite easily undergo β-H elimination, yielding CH_3_OCHO* (methyl formate), HOCHO* (formic acid), or OCHO* (formate), respectively.^48,419,421,422^ Formate is a very stable species on the surface. According to computational studies on Au(111), a hydrogen abstraction from OCHO* by O* or OH* forming CO_2_ + OH*/H_2_O* is associated with a large barrier in the range of 0.95–1.16 eV.^421−423^

In catalytic studies regarding the aerobic oxidation of methanol over NPG and in surface-science experiments on Au surfaces precovered with small amounts of O*, however, almost exclusively methyl formate was formed.^7,382^ This high selectivity toward methyl formate can be explained by a relatively high activation barrier of the CH_3_O* → CH_2_O* step (“β-H elimination”)—higher than for the preceding CH_3_OH* → CH_3_O* step—and essentially barrierless subsequent coupling and deprotonation steps: CH_3_O* + CH_2_O* → CH_3_OCH_2_O* → CH_3_OCHO*.^48,419^ The same basic oxygenated species as in the case of β-H abstraction are likely to also be responsible for the “final” H abstraction step in this sequence. Formaldehyde (CH_2_O*) formed in a slow step should be a minority, whereas CH_3_O* a majority species on the surface. Formaldehyde can thus immediately react with the unreacted methoxy (CH_3_O*), yielding methyl formate (CH_3_OCHO*).^7,382^ In contrast, an analogous addition reaction between CH_2_O* and O*, OH*, or OOH* (which may react directly or first decompose to O* + OH*) would result in the formation of diolates OCH_2_O* or HOCH_2_O*, followed by their transformation to OCHO* (formate) and HOCHO* (formic acid), respectively, after subsequent β-H abstraction steps. Thus, it is the abundance of methoxy that seems to be responsible for the selectivity to partial oxidation. Total oxidation of methanol would proceed via OCHO* (formate). However, this route is limited by the concentration of O*, OH*, and/or OOH* on the surface (required to convert formaldehyde to HOCH_2_O* or OCH_2_O*).

### 4.2 Insights from Single-Crystal Surfaces

#### 4.2.1 Nature of Activated Oxygen Species

Experiments conducted on single-crystal surfaces under well-defined conditions significantly contributed to our understanding of some of the structural and chemical preconditions manifesting NPG’s catalytic potential. Although gold surfaces, due to their inertness, exhibit only weak interactions with many molecules, including molecular oxygen and hydrogen (see above), corresponding UHV experiments carried out at sufficiently low temperatures allowed studying some of the central surface species involved in the catalytic reaction network and, in this way, tremendously helped unraveling their respective surface chemistry.

As far as oxygen is concerned, one important impediment inherent to this approach had, however, to be conquered first, namely the fact that, under UHV conditions, molecular oxygen cannot be activated on Au surfaces. The effectiveness of this restriction was validated for polycrystalline as well as for various single-crystal Au surfaces, including stepped ones, such as Au(221), for pressures up to 1 bar and temperatures up to 900 K.^425−428^ This inability is not unexpected and perfectly in line with theoretical calculations predicting very low adsorption energies and high dissociation barriers for molecular oxygen on pure gold surfaces (section 4.1).

To experimentally overcome this problem, single-crystal Au samples have to be exposed to activated oxygen species to allow studies of their subsequent reaction with other molecules, such as CO or methanol. To this end, various strategies had been taken advantage of, including reactive adsorption of NO_2_, O_3_, or atomic oxygen as generated by a plasma source or thermally by a filament or a cracker.^401,428−432^ To characterize the nature and the varying properties of the oxygen phases, produced on the surface by such methods, STM and XPS studies, mainly on Au(111), were carried out which succeeded to identify a wealth of different species forming as a function of coverage and differing in structure and reactivity.^401^ Interestingly, aggregation leading to the growth of first small oxygen islands was already observed at small coverages. These features, however, structurally changed considerably at higher O doses, as also reflected by characteristic variations in the O 1s binding energy. In short, the results were interpreted in terms of chemisorbed oxygen atoms created first, followed by the formation of various surface and subsurface oxides at higher coverages.^401^

Even though such oxygen surface species are most likely relevant for NPG as an oxidation catalyst as well, the situation is expected to be even more intricate here, taking the significantly higher structural complexity of the material into account. As discussed in the previous section, low-coordinated sites, such as steps or kinks, were theoretically proposed to serve as preferred adsorption sites for oxygen atoms which then tend to aggregate into chain-like structures with increasing coverage.^411^

Catalytic results with NPG (see section 4.3) are compatible with the idea that different oxygen species with deviating chemical nature are available on its surface and that these are the decisive factor regulating the activity for total and partial oxidations. It was found, for instance, that NPG samples pretreated with ozone (O_3_) and strongly oxidized are readily active for methanol oxidation, while it turned out that they are inactive for CO oxidation (for which, in contrast, other activation protocols work: see below).^433^ These results therefore suggest that the oxygen species required for both types of reactions are of different character. Even though the picture obtained in this respect is not complete so far, we will, in the following, review the hitherto existing knowledge gathered from single-crystal studies of CO oxidation and methanol oxidation.

#### 4.2.2 CO Oxidation

**Figure 18:**
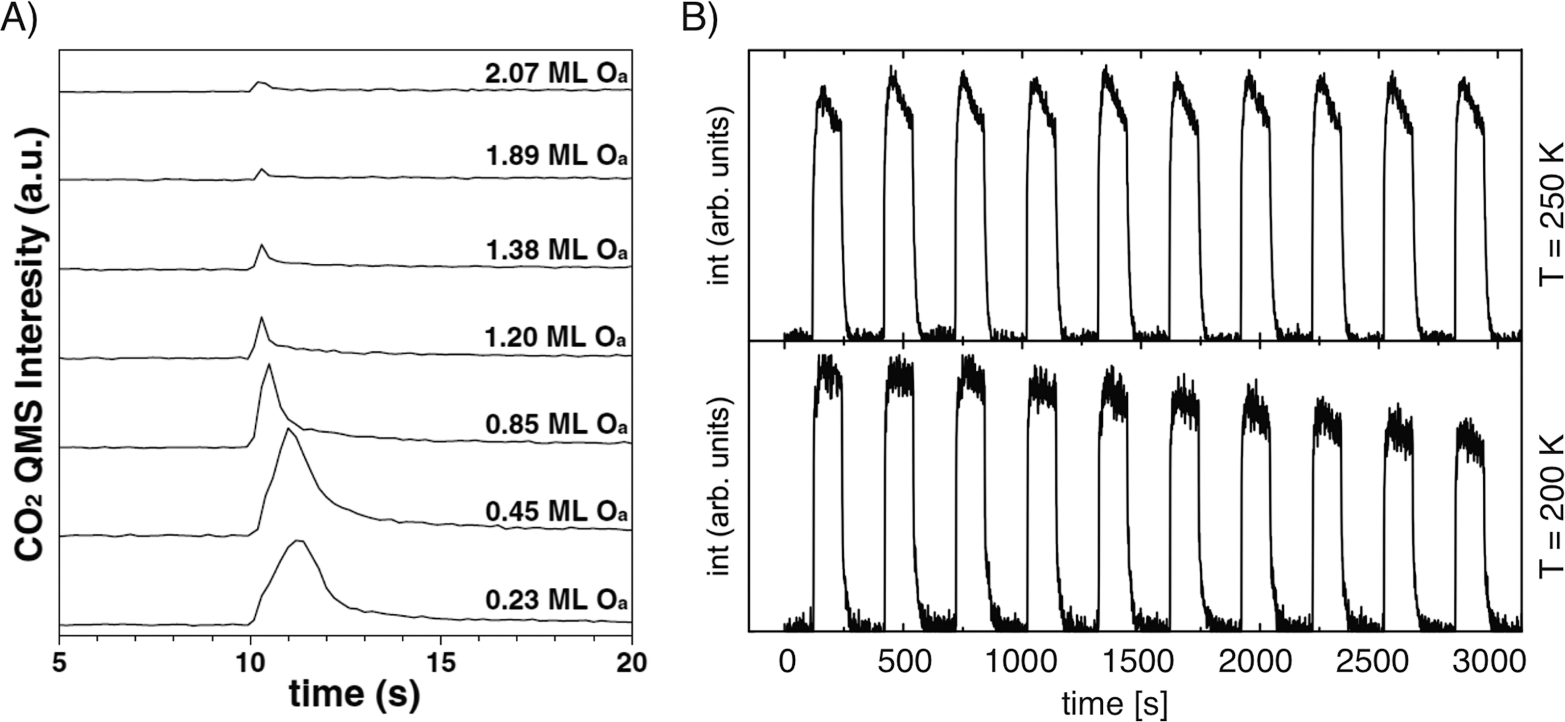
(A) CO titration experiment on Au(111) at 77 K. An RF-plasma was used to adsorb reactive oxygen in the quantities given above each curve. The surface is exposed to a beam of CO molecules (flux 9 × 10^13^ molecules/cm^2^.^434^ (B) CO_2_ intensity (*m*/*z* = 45) observed for CO oxidation on Au(332) during a pulsed molecular beam experiment. A constant flux of ^13^CO (*p* = 2.4 × 10^–6^ mbar) and pulses of oxygen atoms (duration 150 s; flux(O) = 8 × 10^12^ cm^–2^ s^–1^) were applied.^435^ (A) Reproduced with permission from ref (434). Copyright 2007 Springer Nature. (B) Prepared by us from data in ref (435).

Already in the late 1980s, isothermal (300 K) CO titration experiments on an oxygen precovered Au(110) surface suggested that the reactivity of activated oxygen species toward CO oxidation drops substantially with increasing oxygen coverage.^45^ This result was corroborated by later studies depicted in Figure 18A. The CO titration experiments shown and carried out at 77 K on Au(111) evidence a significant activity toward CO oxidation at small initial oxygen coverages but reveal very little activity at higher coverages under otherwise identical conditions.^434^ Concomitantly, the changes in the shape of the titration curves point to a rather complex evolution as far as the reactivity of the formed oxygen species is concerned, when increasing the surface concentration.

More profound insight into such questions can be gained based on molecular beam experiments, offering a unique way to follow the development of such oxygen species alongside with their reactivity under isothermal and well-defined flux conditions. Corresponding results, recently obtained using a stepped Au(332) surface and two separate effusive beam sources for CO (constant) and oxygen atoms (pulsed supply), are presented in Figure 18B.^435^ Qualitative differences in CO_2_ formation are readily identified when comparing the pulse series acquired for 200 and 250 K. While the amount of CO_2_ formed during the oxygen pulses decreases with the number of pulses in the first case, such a behavior is not detected at the higher temperature. Yet, at 250 K, a considerable deactivation *within* the pulse is found instead, as deduced from the peak shapes. (In contrast, the activity toward CO_2_ formation during each pulse is only slightly reduced at 200 K.) As the rate and total number of oxygen atoms impinging on the Au surface are constant during each pulse and a constant sticking coefficient was found for a wide range of oxygen exposures, the declining peak intensities during the pulse sequence at 200 K suggest that the removal of oxygen atoms by CO oxidation cannot compete with their supply by the molecular beam so that oxygen accumulates during the pulse and cannot be reacted off completely in the delay times. In consequence, unreactive oxygen islands (see above) are apparently formed which cumulatively deaden the oxidation activity as more oxygen is deposited.^435^

While, as proven by IR spectroscopy, CO has a sizable transient surface concentration at 200 K on Au(332), the corresponding absorption bands disappear at 250 K indicative for a significantly reduced transient concentration.^436,437^ As a consequence, more oxygen is accumulating during the pulse, resulting in a more pronounced decrease of the reactivity. However, the rate constant for CO oxidation is significantly higher at 250 K than at 200 K, as readily inferred from the fact that CO is capable to react with the accumulated oxygen atoms in the off-periods of the pulse sequence. In essence, a comparable (reactive) surface state is reached at the beginning of each pulse so that the same shape of the CO_2_ formation pulse is recorded throughout the sequence. This and the other work described above implies that NPG’s catalytic activity for low-temperature CO oxidation, i.e., the CO_2_ formation rate observed under (real) reaction conditions (section 4.3), should sensitively depend on the availability of oxygen entities providing the right reactivity for total oxidation on the surface which, in turn, is expected to be the result of a delicate balance of different species and phases changing with temperature and oxygen partial pressure.

In contrast, for CO, as the other reactant, the situation seems less complex. In this case, surface science studies on Au(111) clearly indicated that the CO binding energy on ideal terrace sites is too low to account for sizable CO surface coverages under catalytic conditions (300 K, 1 bar).^45,437−440^ Yet, the detailed structural characterization of NPG by HRTEM provided ample evidence for a high abundance of low-coordinated Au surface atoms located along atomic step edges (in areas of step bunching) or at kinks on the ligaments.^359^ Experiments on stepped as well as roughened surfaces showed that low coordinated sites exhibit significantly higher binding energies, which is also in line with the computational results (section 4.1).^437,439^

Nevertheless, not only with respect to oxygen activation, but also to CO adsorption, UHV model studies were not able to disclose all the properties of NPG. Specifically, temperature-programmed desorption spectra recorded for NPG samples under UHV conditions provided clear evidence for additional CO species bound even stronger than at “simple” Au step and kink sites.^406^ Different scenarios such as Au sites in the direct neighborhood of Ag (or AgO_*x*_ species) were discussed to account for this extra stabilization. Yet, a final clarification of this topic, including the question whether these CO species play a role in the catalytic cycle, is still outstanding.

#### 4.2.3 The Role of Water in CO Oxidation

The preceding subsection summarized what is known regarding the dependence of CO oxidation activity on the coverage and speciation of oxygen species that can form on Au. It should be stressed, however, that the catalytic behavior can also be strongly impacted by other environmental conditions, i.e., by other molecules which are present in the gas phase and can coadsorb on the surface. This topic can be especially well-illustrated at the example of water which, as a matter of fact, represents a common impurity in many (technical) feeds.

Interestingly, it was observed that water enhances the catalytic activity of NPG for CO oxidation.^390^ This is in line with findings for oxide-supported Au nanoparticles, but opposed to other catalytic reaction of NPG such as the partial oxidation of methanol. This cocatalytic effect could also be reenacted in experiments on Au(111) under UHV conditions, suggesting an increase of the reactivity of predeposited oxygen species toward CO oxidation.^431^ In conjunction with further work on this subject, such model studies enabled proposing a reasonable mechanism for this effect.

**Figure 19:**
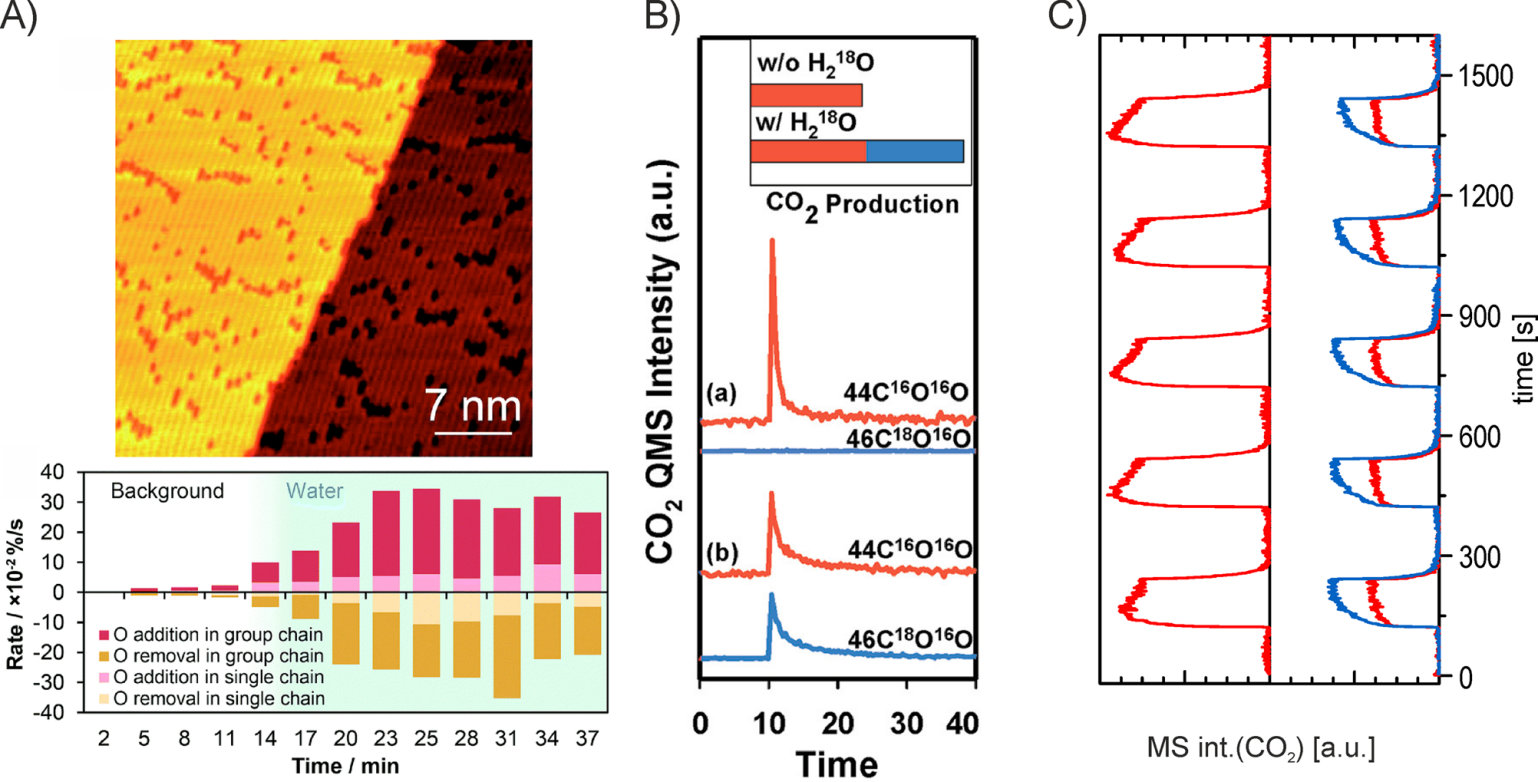
(A) (top) STM image of 0.04 ML of O/Au(110)-(1×2); (bottom) analysis of oxygen redistribution induced by 3 × 10^–10^ Torr water at 170 K.^442^ (B) CO_2_ formation upon a CO titration experiment on Au(111) at 140 K. The Au(111) surface had been precovered with 0.11 ML ^16^O. In (B) 0.14 ML H_2_^18^O were additionally adsorbed. Inset shows the total amount of CO_2_ produced in each case.^55^ (C) CO_2_ intensity (*m*/*z* = 45 (red); *m*/*z* = 47 (blue)) during CO oxidation on Au(332) during a pulsed molecular beam experiment at 250 K for “dry” (left) and “wet” conditions (right). A constant flux of ^13^CO (*p* = 2.4 × 10^–6^ mbar) and H_2_^18^O (*p* = 5.1 × 10^–6^ mbar) and pulses of oxygen atoms (duration 150 s; flux(^16^O) = 8 × 10^12^ cm^–2^s^–1^) was applied. (A) Reproduced with permission from ref (442). Copyright 2018 Royal Society of Chemistry. (B) Reproduced in part with permission from ref (55). Copyright 2008 American Chemical Society. (C) Prepared by us from data in ref (435).

First, experiments using isotopically labeled water (H_2_^18^O) yielded evidence that oxygen atoms (pre)adsorbed on Au can efficiently interact with water by transiently forming hydroxyl groups. Specifically, CO_2_ containing ^18^O was detected as an oxidation product of C^16^O and ^16^O_ad_ when H_2_^18^O was co-dosed (Figure 19B), a finding which is only explainable by a scrambling reaction according to
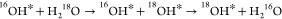
14

In experimental agreement with larger desorption temperatures of water from oxygen precovered vs clean Au surfaces, DFT calculations confirmed that such exchange processes are indeed feasible because they are associated with comparatively low activation barriers.^55,441^

Another piece of information was provided by STM investigations performed on oxygen precovered Au(110) surfaces. Upon coadsorption of water, an increased mobility of oxygen atoms and, in succession, a restructuring of previously formed oxygen islands on the surface was observed (Figure 19A).^442^ Combining all findings, the following scenario becomes likely: The presence of water and its coadsorption lays the foundation for a transient and reversible formation of surface hydroxyl groups. In addition to their impact on the reaction mechanism discussed in section 4.1, they have the capability to mobilize oxygen on the surface and may thus initiate (at least a partial) disaggregation of otherwise catalytically inactive oxygen phases/islands. On these grounds, water can bring about a regeneration of active oxygen species and, in consequence, can lead to higher catalytic turnovers.

This cocatalytic effect of water for low-temperature CO oxidation was also underpinned by recent isothermal molecular beam experiments depicted in Figure 19C. Here, CO oxidation was probed at 250 K by pulsing ^16^O atoms on a Au(332) surface while keeping a constant flux of ^13^C^16^O under “dry” (left) as well as under “wet” (right) conditions, using H_2_^18^O.^435^ In line with the titration experiments shown in Figure 19B, quite efficient isotopic scrambling within the product CO_2_ (^16^O/^18^O) was detected in spite of the fact that the reaction was carried out about 60 K *above* the water desorption temperature. While corroborating the studies discussed above, the data additionally disclosed considerable changes in the transient kinetics of the system under the “wet” conditions. While significant deactivation is observed without water (Figure 18B), the presence of water obviously enables a stable steady-state reactivity. Attaining this steady state requires almost the whole pulse length (note the increasing and finally saturating CO_2_ formation during the pulses), indicating that the corresponding processes to establish steady-state conditions are rather slow.

Overall, all results reviewed here point in the same direction, namely to an enhanced availability of those oxygen species which are responsible for the catalytic activity in case of CO, i.e., for total oxidation, in the presence of water. Even though a more detailed mechanistic description of the altered reaction network would be highly desirable, it currently lies beyond the scope of the knowledge which has been achieved based on UHV model studies carried out so far.

#### 4.2.4 Methanol Oxidation

Apart from single-crystal experiments related to CO oxidation, model studies were particularly successful in proposing the reaction mechanism and further mechanistic details for the partial oxidation of alcohols over NPG.^7^ First and foremost, methanol has been the focus of numerous studies ever since the first work was published reporting the ability of NPG to efficiently catalyze both, total and partial oxidation reactions at low temperatures.

**Figure 20:**
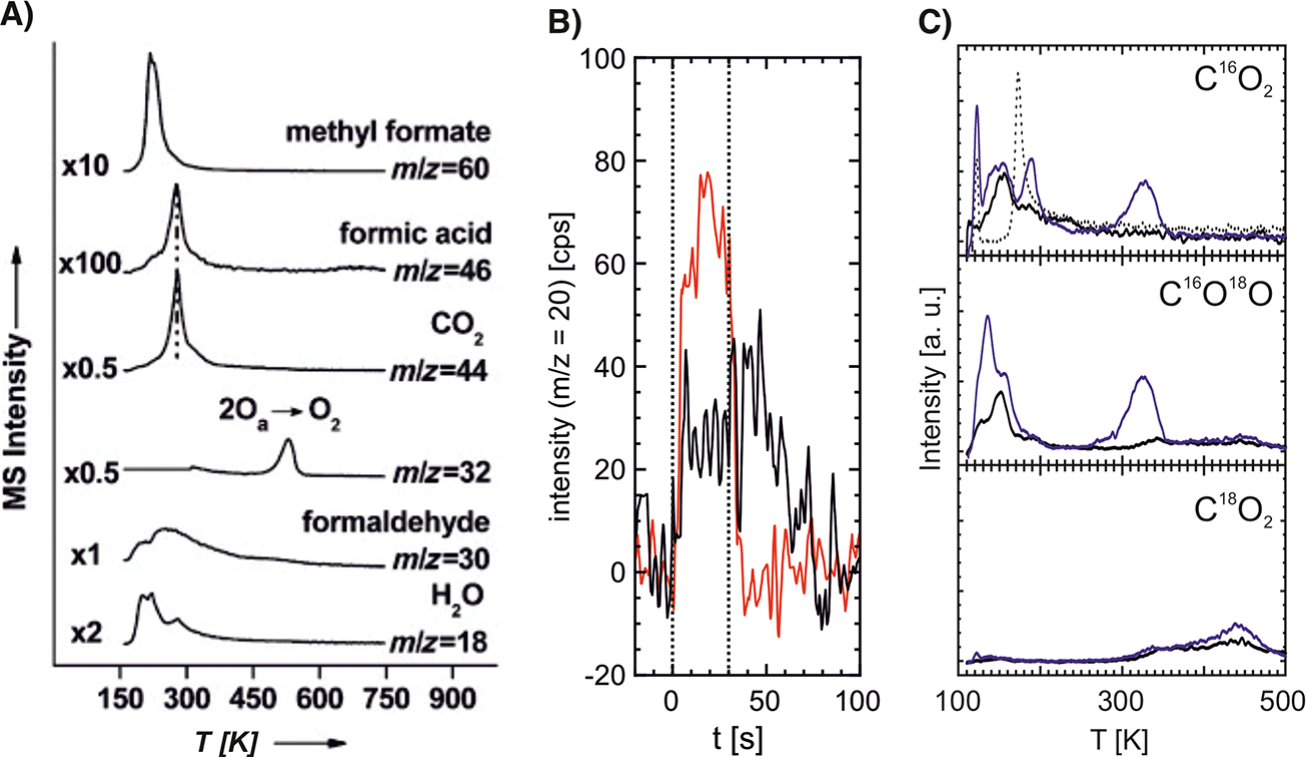
(A) TPR spectra of CH_3_OH on O/Au(111) (θ_O_ = 0.2 ML). Ozone was dosed at 200 K to deposit oxygen on the Au(111) surface.^382^ (B) Isothermal pulsed molecular beam experiments on Au(332) at 310 K pulsing atomic oxygen (6.9 × 10^11^ cm^2^ s^–1^, 30 s on, 100 s off), while continuously supplying methyl formate (1.2 × 10^15^ cm^2^ s^–1^; black trace) or methanol-^13^C (4.2 × 10^13^ cm^2^ s^–1^; red trace).^449^ (C) TPR spectra of 0.11 L methyl formate on 0.13 ML ^18^O precovered Au(332) (blue); reference spectra: 0.13 ML ^18^O reference on Au(332) (black) and a 0.11 L methyl formate reference (gray dots).^450^ (A) Reproduced with permission from ref (382). Copyright 2009 Wiley-VCH. (B) Adapted with permission from ref (449). Copyright 2022 Royal Society of Chemistry. (C) Adapted with permission from ref (450). Copyright 2021 American Chemical Society.

The underlying mechanism already proposed in the first reports and experimentally derived from a combination of previous TPR experiments (Figure 20A) and vibrational spectroscopy was already presented in Figure 17.^382^ The surface chemistry determining the partial oxidation of alcohols was accordingly interpreted on the basis of acid–base surface chemistry concepts and proved to be quite predictable on these grounds. The starting point of the mechanistic considerations is the Brønsted basicity of the oxygen species adsorbed on the Au surface.^46,443−446^ It was possible to readily explain the selectivity pattern observed for methanol oxidation over NPG as soon as the first results were published on this topic. In agreement with that, not the direct aldehydes or carboxylic acids were found as products, but solely esters, i.e., coupling products, such as methyl formate in the case of methanol oxidation. This preference for oxidative coupling reactions was not only verified for single alcohols, but the validity of the mechanistic framework was also demonstrated to hold for the cross coupling of different alcohols and alcohol aldehyde mixtures as well as for the oxidative coupling of amines and alcohols to amides.^379,447,448^

The common notion regarding the nature of the relevant oxygen species driving the selective formation of oxidative cross coupling products is that chemisorbed O atoms and their favorable basicity are responsible for the initial and rate-determining hydrogen abstraction steps. Within this picture, however, the less specific redox properties of the oxygen species and oxide phases, governing, as discussed above, the ability of NPG for CO oxidation, are not considered. A corresponding transition of reactivity is reflected, e.g., in temperature programmed reaction spectroscopy (TPRS) experiments on Au(111) showing a rapid decrease of selectivity toward overoxidation products at oxygen coverages above 0.1 ML. Only below, selectivities above 70% toward methyl formate were found.^382^ In agreement, comparatively high selectivities toward methyl formate of 65% were reported recently for Au(332) at 230 K if exposing the surface to a high flux ratio of methanol to oxygen atoms (*f*(MeOH)/*f*(O) = 660) in a pulsed molecular beam experiment. Notably, these were carried out under single collision conditions, meaning that desorbing educts or intermediates cannot undergo subsequent reactions. In contrast, for NPG studied under catalytic conditions molecules, be it reactants, intermediates, or even products, typically collide several times with the surface before leaving the catalyst bed. Consequently, also model studies based on molecular beam experiments, although coming closer to real catalytic conditions as compared to TPD or TPRS studies may yield selectivity patterns deviating from what is observed for the working catalyst. In the case of partial methanol oxidation, for example, formaldehyde desorption taking place before the molecule can undergo the subsequent coupling reaction to the ester will obviously result in a lower selectivity. In accord, dropping selectivities were indeed observed in isothermal molecular beam experiments upon raising the temperature.^451^

A further aspect that may limit the comparability of such model studies with catalytic results is related to the fact that, in the former case, the product methyl formate may desorb after its formation and thus escape further potential conversion steps. On the contrary, the chance for secondary reactions, possibly leading to total oxidation, in a catalyst bed or in a larger monolithic NPG catalyst is high due to the likelihood of multiple collisions with the catalytic surface. The propensity of a partial oxidation product, in this case methyl formate, to undergo such an overoxidation process upon readsorbing on the catalyst surface can, however, be separately checked in molecular beam experiments. Notably, such information can be of value when aiming at an optimization of real catalytic processes, e.g., by adapting catalyst bed lengths or the flow conditions to minimize secondary reactions.

In this spirit, additional pulsed molecular beam experiments were performed under isothermal conditions to assess the propensity of methyl formate for overoxidation, i.e., to allow for an explanation why methanol partial oxidation is distinctly preferred over its total oxidation under working conditions. To this end, the isothermal oxidation rate of methanol and methyl formate was compared by monitoring the water (mass spectrometrically) formed in the corresponding reactions. To mimic the scenario of an NPG catalyst working at high conversion and high selectivity the flux of methyl formate was chosen to be a factor of 35 higher than that of methanol, keeping all other parameters constant. Figure 20B shows the amount of water produced during an oxygen pulse while exposing the surface to a constant flux of either methyl formate (black) or methanol (red) during the entire period.^449^ Despite the significantly higher supply of methyl formate, its overoxidation rate is small as compared to that of partial oxidation. These results suggest that reaction pathways leading to overoxidation of methyl formate do not play any significant role under practical conditions, well in line with the high selectivity of NPG in the partial oxidation of methanol to methyl formate.

In general, two different circumstances could be responsible for the low extent of overoxidation (in spite of its thermodynamic preference): either no reaction channels with sufficiently low activation barriers exist or the availability of corresponding surface sites is (too) low to compete with the partial oxidation pathway. TPRS as shown in Figure 20C also allows to discriminate between the two scenarios.^450^ Three reaction channels resulting in CO_2_ formation are identified, all belonging to minority species of the preadsorbed oxygen. Importantly, two of these are related to low desorption temperatures (135 and 185 K) in accord with low activation energies, while only one occurs at significantly higher temperatures (320 K). Because the two lower ones fall below the temperature of methyl formate formation from methanol in TPRS experiments (220 K, Figure 20A), the associated reaction channels cannot be observed in these experiments as methyl formate has not yet formed.^382^ These results clearly show that overoxidation pathways for methyl formate exist on stepped Au surfaces, although only small amounts of formate are formed. A high selectivity toward methyl formate hence requires that the highly reactive oxygen species capable of overoxidation of methyl formate were not populated under the catalytic conditions applied. In the case of methanol oxidation, this is maintained at not too high oxygen pressures because of the rather slow formation of the oxygen species and a fast and rather unspecific reaction of methanol with different oxygen species. The difference in reactivity can be rationalized by a simple chemical picture: while the partial oxidation of methanol involves a series of hydrogen abstraction processes, i.e., requires Brønstedt basicity of the oxygen species, the overoxidation of methyl formate and other overoxidation channels as well involve an attack of the carbon atom by an oxygen species, i.e., depend on the nucleophilicity or—in the language of acid base chemistry—on the Lewis basicity of these species.

### 4.3 Gas Phase Catalysis on Nanoporous Gold

In sections 4.1 and 4.2, some of the key findings identifying NPG as an active and, more importantly, very selective catalyst for aerobic oxidation reactions in the gas phase have already been mentioned and correlated with insight from theory and advanced UHV model studies. Here, we will complete the picture with the results of catalytic experiments carried out under ambient conditions, again focusing on oxygen as the species apparently controlling the reactivity on the surface. In addition, we review recent efforts to quantify diffusive mass transport in NPG and address its impact on limiting the catalytic performance in gas phase applications.

#### 4.3.1 Impact of Oxygen Heterogeneity on Catalytic Properties

With respect to aerobic oxidation catalysis on NPG, the activation of molecular oxygen is one of the key steps. As discussed in section 4.1, theory has proposed a variety of mechanisms for oxygen activation. Experiments combining catalysis with spectroscopy provided evidence that residual Ag moieties are involved in the activation amending the original perception that NPG is a pure Au catalyst.^57^ In agreement with that, an apparent barrier of about 21 kJ mol^–1^ was determined for oxygen activation on ozone-treated NPG being active for selective methanol oxidation.^452^ This value is close to values found for different Ag surfaces,^453^ while for Au surfaces significantly higher activation barriers are expected (section 4.1). Experimentally, a barrier of about 140 kJ mol^–1^ was estimated for molecular oxygen dissociation on Au(110), for instance, on the basis of temperature programmed desorption results.^438^

Experiments with NPG in a temporal analysis of products (TAP) reactor revealed an oxygen saturation coverage of 4 × 10^–3^ ML at 423 K, being consistent with an activation of molecular oxygen on Ag sites. Yet, no indication for spillover of oxygen species to Au sites could be deduced from these results.^452^ This is a surprising observation as the selective oxidation chemistry of NPG is characteristic of Au, hence, requiring oxygen spillover from Ag to Au when oxygen activation is not possible on Au sites alone. As far as CO oxidation is concerned, previous experiments with a similar experimental setup revealed for a NPG sample containing about 3 times as much Ag on the surface (as determined by XPS), an oxygen saturation coverage being about 5 times as large, corroborating the role of Ag as that component which lends NPG the ability to bind oxygen at the surface.^405^

Although it is difficult on these grounds to draw clear conclusions with respect to those fundamental structure–property relationships governing the catalytic properties of NPG, theory as well as experiments on model systems provide indications that surface oxygen species of different reactivity play a central role, as discussed above. This is consistent with experimental observations of decreasing selectivity for the partial oxidation of methanol to methyl formate when increasing the oxygen partial pressure.^7^ Further evidence comes from differences noticed for the procedures which are necessary to catalytically activate NPG. While ozone treatments result in an active and selective catalyst for the esterification of methanol, this treatment is not suitable for achieving steady-state activity for CO oxidation.^433^ Only transiently oxygen species are created which are active in this case and also lead to total oxidation of methanol (to CO_2_); these sites, however, apparently cannot be replenished sustainably under reaction conditions. In contrast to that, it was recently shown that NPG can be reliably activated for CO oxidation by short annealing treatments to 300 °C in a reaction mixture of CO and O_2_.^454^ It was verified that no thermal coarsening of the pore structure is induced as a side effect, as previously reported for longer annealing times at such temperatures.^455^

**Figure 21:**
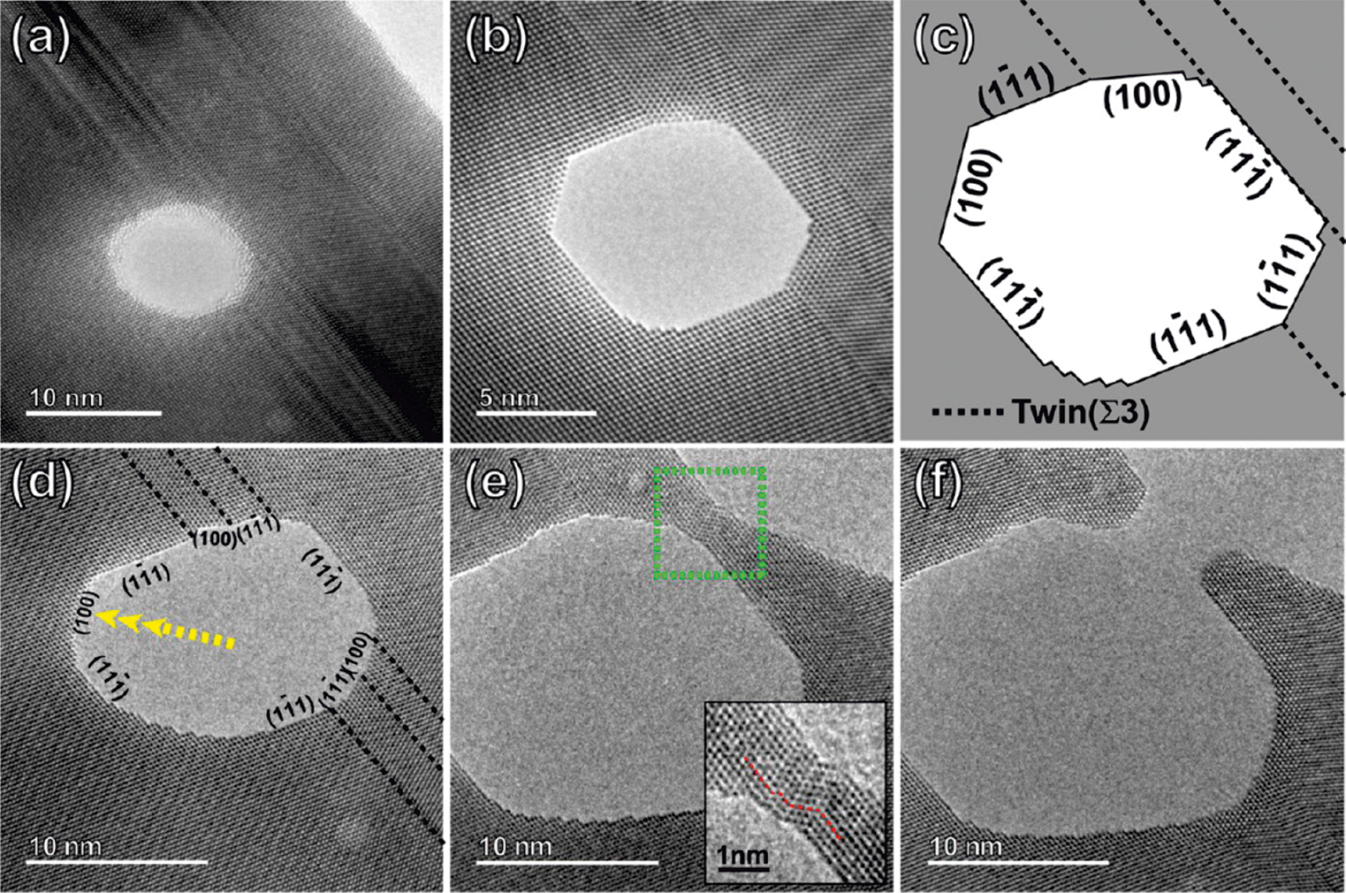
Nanopore coarsening during catalytic reaction: (a) Initial state. (b) Faceting after exposure to CO/air mixture for ∼50 s. (c) Crystallography of nanopore shown in (b). (d) Indication of the preferential growth direction normal to (100) surface plane. (e) Necking of gold ligament before rupture. Inset: magnified view shows deformation twins. (f) Coalescence of small and large nanopores after rupture. Reproduced with permission from ref (359). Copyright 2014 American Chemical Society.

The rather complex transient kinetics associated with the activation procedures point to significant changes of the surface chemistry induced in this way. In this context, dynamic surface restructuring causing a different Ag surface composition could play a role, as observed, for instance, by in situ TEM during CO oxidation (Figure 21).^359^ The extent to which Ag concentrations at the surface are altered is probably also influenced by the fact that the Ag distribution in NPG samples can be quite heterogeneous after dealloying (section 3.13).^89,93^ It furthermore has to be considered for the restructuring dynamics that residual Ag was found to stabilize steps and kinks and is thus likely to mitigate structural changes of the surface structure.^359^

Overall, the combined insight from theory, single-crystal studies and catalytic experiments suggests that oxygen atoms with quite different properties drive CO oxidation and the esterification of methanol. While the latter requires surface oxygen with sufficient Brønsted basicity to allow for a rapid formation of adsorbed methoxy species (or, in general, alkoxy species) and subsequent β-hydrogen abstraction, such species should exhibit a low reactivity with respect to a nucleophilic attack of the reaction intermediates such as formaldehyde to avoid overoxidation products (Figure 17). In contrast to that, good CO oxidation activity requires oxygen species which readily attack the carbon atom of adsorbed CO, i.e., have a sufficiently high Lewis basicity. More far-reaching conclusions, however, are hampered in view of a complex surface chemistry as revealed, for example, by the finding that various oxygen species on NPG were found to oxidize CO readily, but that only a subset of them seems to possess the potential for stable and sustainable conversion rates under catalytic condition. This has not only been observed in case of ozone-activated NPG (s. above) but also for NPG samples activated in a mixed CO/O_2_ stream.^406^

The heterogeneity of the system renders a clear assignment of the moieties responsible for the catalytic activity of NPG, in particular for CO oxidation, challenging. Future mechanistic models, however, have to comply with the observation that experimental kinetic studies revealed reaction orders for oxygen close to 0 and for CO between 0.8 and 1.^57^ These data suggest that the supply of active oxygen species for CO oxidation is rather efficient on the surface and in the first place limited by the availability of CO molecules. Because oxygen activation is ascribed to Ag sites, in line with the experimental finding that increasing amounts of residual Ag leads to increasing CO_2_ formation rates, the low reaction order of O_2_ suggests a high reactivity of such sites. Even though in the literature mostly metallic Ag or bimetallic Ag/Au sites have been discussed in this context, AgO_*x*_ species, likely to be formed on the surface in particular under oxygen excess conditions, cannot be ruled out as possible candidates. In essence, it has to be stated that the current mechanistic understanding derived from theory, model studies, and catalysis is not sufficient enough to explain all aspects of the catalytic behavior of NPG, including, for instance, selectivity changes. So far it is not clear which changes regarding the nature and/or concentration of Ag-related surface sites or also Au sites are in detail responsible for the detected transition from partial (esters) to total oxidation products (CO_2_) in the case of alcohol oxidation.^7^

#### 4.3.2 Macro- and Microkinetics: From Transport Properties to Reaction Mechanism

As already pointed out, one aspect also and potentially considerably impacting the catalytic performance of NPG in practical applications relates to mass transport limitations as resulting from the nano- or, being more precise, mesoporosity of the material. If the rate at which the reactants are supplied within the pore system or the products are transported out of it is in the same range as the reaction rate or even lower, a reduction of the achievable catalytic conversion is expected. In view of NPG’s pore diameters lying in the range of a few 10 nm, corresponding mass transport limitations are not unlikely.

Such contributions can be assessed based on the Thiele modulus formalism, being a well-established concept in chemical engineering. To this end, however, structural information is needed.^456^ On the one hand, the mean pore diameter will determine to which extent the mean free path of the diffusing gas molecules is reduced by Knudsen diffusion. On the other hand, nonstraight pores, i.e., the tortuosity of the pore system (section 3.10), will lead to elongated diffusive pathways which the molecules have to overcome within the porous material.

Recently, pulsed field gradient (PFG) NMR spectroscopy was employed to evaluate to which extent NPG’s tortuosity slows down the diffusive transport. For CO and CO_2_, as reactant and product molecules of CO oxidation, as well as for CH_4_, included in the study as an inert gas for reference purposes, consistently, a mean pathway elongation by a factor of about 2 was derived in all cases.^457^ On the basis of this result, representing the first experimental assessment of NPG’s (diffusive) tortuosity factor, and the mean pore diameter to take the contribution of Knudsen diffusion into account, it was possible to quantitatively describe the macrokinetics, i.e., the experimentally observable and obtainable catalytic CO conversion rates, as a function of the diffusive mass transport and the microkinetics of the surface reactions.

**Figure 22:**
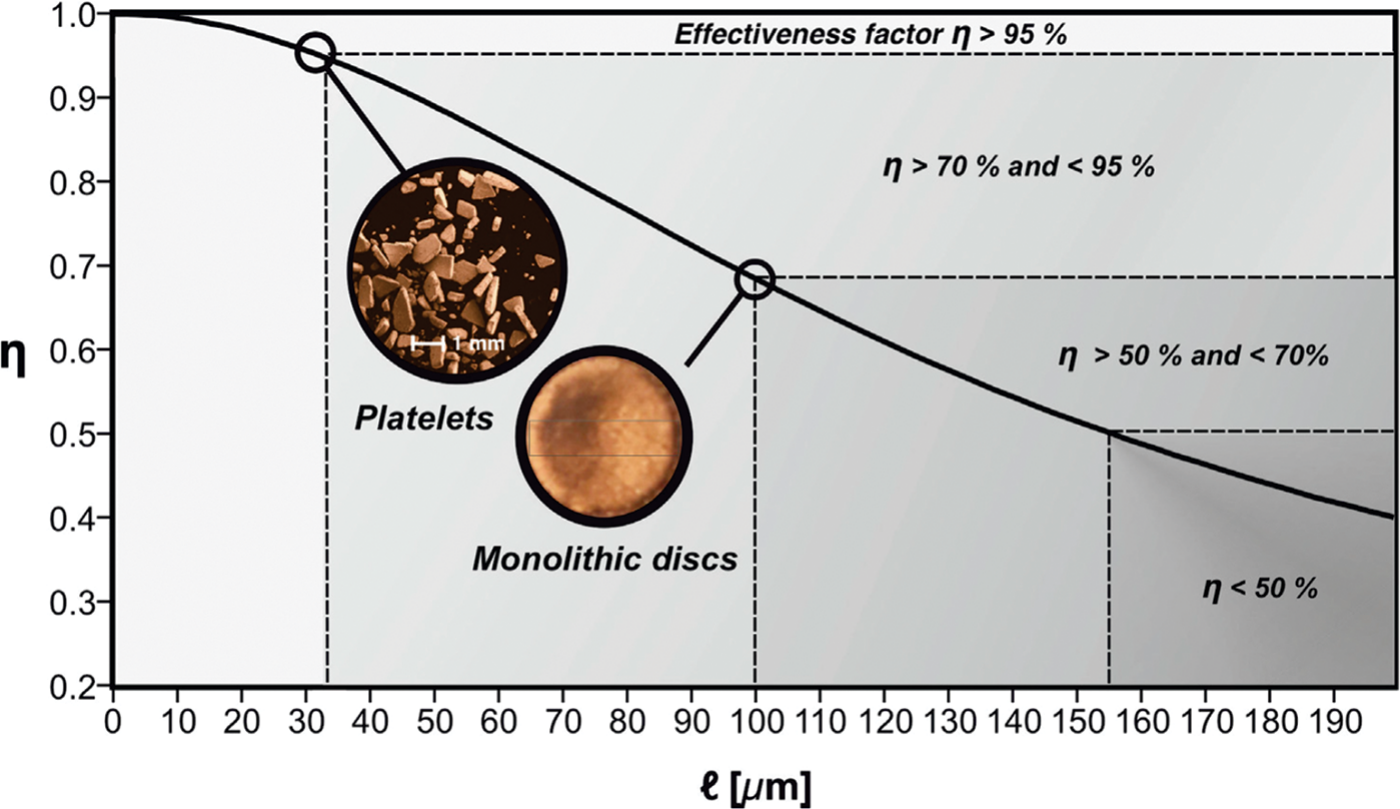
Plot of the catalytic efficiency (η) as a function of the characteristic length () associated with NPG catalysts exhibiting different macroscopic shapes. Values for NPG (ligament size 30 nm) discs (*d* = 5 mm; *h* = 200 mm) and particles (all dimensions approximately 200 mm) are marked. Reproduced with permission from ref (457). Copyright 2022 The Authors.

In this context, it turned out that larger monolithic NPG catalysts, as were often applied in the literature in the form of discs exhibiting diameters in the range of several mm and thicknesses of a few 100 μm, are indeed associated with reduced catalytic efficiencies. As shown in Figure 22, for such a monolithic NPG disc (diameter, 5 mm; thickness, 200 μm; mean ligament size, 30 nm), the catalytic efficiency is limited to ∼70%. When, however, breaking up the discs into smaller platelets (of the same thickness) so that their diameters decrease to just about a few 100 micometers, the efficiency can be increased to almost 100%—in agreement with the predictions of the Thiele modulus formalism and NPG’s structural characteristics (see solid line in Figure 22).^457^ This result demonstrates that the well-defined and well-controllable features of NPG in conjunction with options to adapt the macroscopic shape of the material to a particular set of reaction conditions provides a good basis for predicting and optimizing its performance in a given catalytic application.^456^

An insight into the kinetics determining the processes on the surface itself was gained by surface microkinetic simulations carried out for the esterification of methanol using a reaction network in line with the knowledge about the reaction mechanism established so far (Figure 17).^403^ For these simulations, the kinetic constants entering the microkinetic simulations were largely deduced from experimental data as obtained from single-crystal experiments; however, a few were taken from DFT calculations.

**Figure 23:**
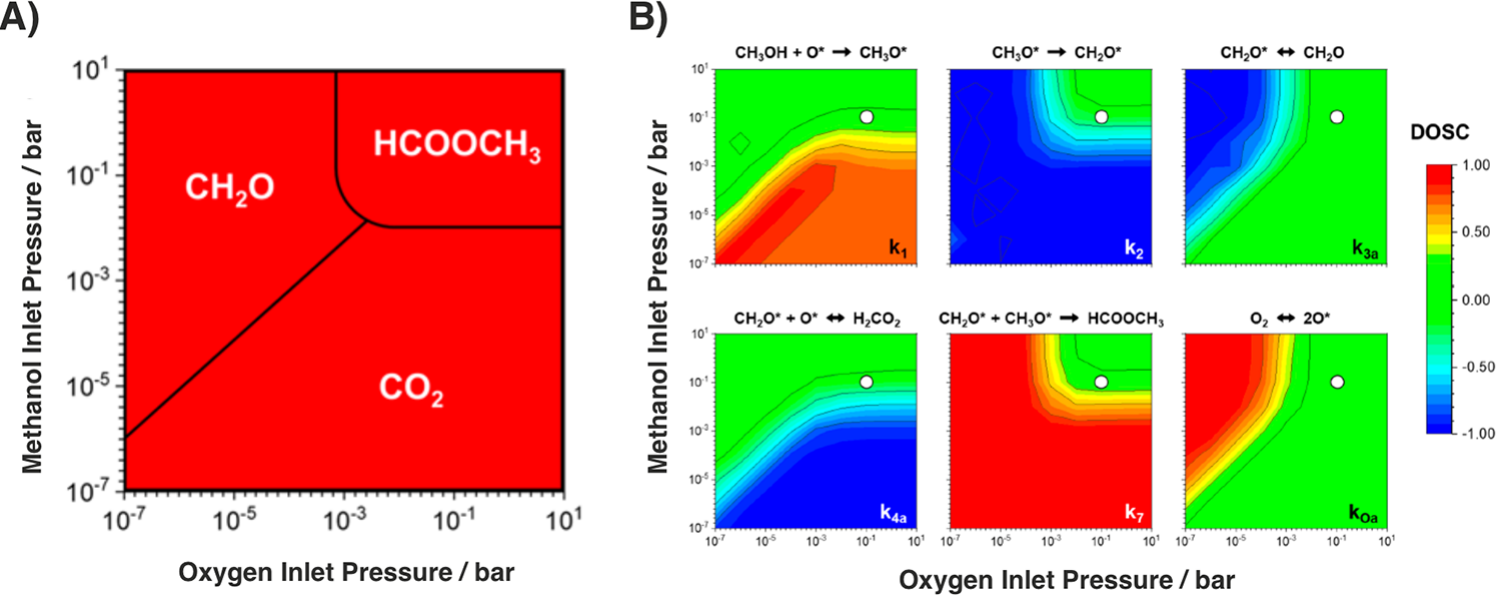
(A) Selectivity phase diagram for the oxidation of methanol at 383 K. In each region formaldehyde, methyl formate, or CO_2_ is the major product (>50% selectivity). (B) Degree of selectivity control (DOSC) for methyl formate formation. The DOSC was calculated for different elementary reactions using plug flow reactor simulations at 423 K as a function of oxygen and methanol partial pressures varying between 10^–7^ and 10^1^ bar. Reproduced from ref (403). Copyright 2019 American Chemical Society.

In a first study, the validity of the approach was confirmed by simulating the results of a TAP reactor study.^458^ As the calculations were able to predict the measured transients with reasonable precision, the methodology was extended to simulations of a plug flow reactor.^403^ Based on these simulations, it was possible to predict a variety of interesting properties important for an understanding of the catalytic performance, in particular the selectivity of the system. Figure 23 illustrates this point. The simulations predict preferential formation of formaldehyde, methyl formate, and CO_2_ in specific regions of the pressure space (Figure 23A). Additionally, Figure 23B shows so-called degree of selectivity control (DOSC) plots for several elementary reactions of the reaction network using the same parameter space as in Figure 23A, i.e., the pressure of methanol and oxygen. The DOSC is a parameter which ranges between −1 and 1 and approaches 1 (−1) for a specific reaction channel in case the selectivity toward a certain product is strongly enhanced (decreased) with increasing reaction rate. As seen for the three pairs of elementary reactions plotted on top of each other, the simulations predict complementary DOSC values, i.e., the plots in each row show DOSC with opposite sign in very similar regions of methanol/oxygen pressures, suggesting a rather strict interdependence of the associated reaction paths within the network. It should, however, be kept in mind that the analysis still needs to simplify the system. In this particular case, the simulation assumes a single active oxygen species, whose activation energy of formation is comparable to values found on Ag surfaces.^452^

### 4.4 Evolution of the Material during Catalysis

Although it was claimed that NPG is a catalyst with a high structural stability,^7,459^ there is an evolution of the material during catalysis. The most obvious change of the material is coarsening, i.e., an increase of pore and ligament sizes, which sometimes is noticeable at first glance when examining the samples, e.g., in a SEM. As has been outlined in section 2.2 above, coarsening is routinely observed in NPG; it acts during the dealloying process itself, and it can be deliberately promoted by annealing in order to tune the ligament size. Under otherwise identical annealing conditions, it has been found that the chemical environment in the pore space has a strong influence on the thermal coarsening of NPG; samples in vacuum are typically more stable than in a gas atmosphere,^460^ but strongly adsorbing species such as atomic oxygen can also impede the coarsening.^461^ Obviously, a strong connection between the coarsening in NPG and the environment to which it is imposed during catalysis may be expected. Generated heat during exothermic reactions or high reaction temperatures (>70–80 °C) might suffice to overcome kinetic barriers for transport processes at the surface which are required for reducing the surface area and, thereby, the net excess surface free energy, by coarsening.^63^

The growth of ligament sizes was observed for example during (electrochemical) methanol oxidation^34,59,61^ and aerobic CO oxidation.^359^ Yet, it was found in the case of disc shaped NPG that coarsening of the structure is smaller in the bulk than in a skin layer at the outer surface of the disc exhibiting a thickness of ≈100 nm (see section 3.8).^34^

As coarsening is mainly a surface diffusion-dominated process, the surface of NPG must be considered as a highly dynamic system under reaction conditions.^364^ A great challenge thus is the in situ investigation under catalytic conditions. In recent years, in situ TEM measurements became technically more and more available, but such experiments still suffer from significant material and pressure gaps as compared to catalytic studies carried out at atmospheric pressure or even above. In particular, a dedicated sample preparation is necessary because TEM measurements require samples with a thickness <100 nm. Furthermore, only 2D projections can be measured in situ by TEM, and the influence of the electron beam can never be excluded fully and has to be checked carefully.^15,337,359^ Besides TEM, the surface of NPG can be investigated by electrochemical cycling, XPS or STM during catalysis to unravel its structural and chemical characteristics. Despite all challenges, important insights with respect to the prerequisites for catalytic activity were obtained. A rearrangement of atoms on NPG surfaces was observed, for instance, when samples were exposed to CO/air mixtures, which does not occur under exposure to the single gases (N_2_, O_2_, or CO).^337,359^ Another study found that adsorbed oxygen can stabilize the Au surface (see above).^461^ Further studies found pronounced changes by heating in air, N_2_, or O_2_ but not under an Ar atmosphere.^127^ Studies in which electrochemical cycling of NPG was investigated revealed that surfaces restructure to form thermodynamically more stable facets under reaction conditions.^34,99,191^ For example, the fraction of the most stable Au (111)-facet increased by 10% after cycling 200 times in sulfuric acid. The degree of atom surface diffusion depends on the scan rate and is limited by curvature and sizes of ligaments, respectively. Again, this finding identifies curvature-driven surface diffusion as the dominating process for the reordering.^462^

Several processes were identified that can suppress the mobility of atoms on the NPG surface. It was found that the higher the concentration of LNEs on the surface, the more the mobility is suppressed. The presence of Ag on the surface, for instance, turned out to exert a stabilizing effect.^337,364,462^ Enhanced stability was also found when a third element with a higher melting point (e.g., Pt) was added, which is able to stabilize undercoordinated Au surface atoms.^61,99,114,241^ Also dealloying of ternary alloys results in lower surface mobility and hence less coarsening.^63^ Defects in the crystal lattice can also have a stabilizing effect. An in situ TEM study revealed that coarsening is prevented by planar defects because diffusing atoms are pinned to these defects.^359^ However, also crystal defects are object to change under reaction conditions as pointed out in ref (356). Furthermore, different studies suggested that coarsening is less pronounced when the samples are electrochemically cycled prior to catalysis experiments^359,462^ or when oxide particles or layers are deposited on the ligament surfaces of NPG.^105,381,460,463−466^

In addition to the structure, the distribution of LNEs remaining in the material after dealloying potentially is subject to evolution and, in particular, to segregation to the surface. At first sight, it can be expected that the surface of dealloyed NPG consists of pure Au (section 2). However, a complete removal of the sacrificial element is impossible for thermodynamic reasons (Nernst distribution law) so that *x*_Ag_^res^ of a few at. % is typically detected even after long dealloying times. It is obvious that a movement of atoms on the surface, in particular at step edges, and concomitant coarsening of the structure can eventually expose LNEs to the surface, leading to an increased concentration there during (or after) catalysis. Furthermore, also diffusive segregation of the LNE to the surface is likely under reaction conditions.^23^ Such an effect (in most studies detected for Ag because Au−Ag starting alloys were predominantly used) was actually observed during catalytic CO oxidation^57,359^ and, e.g., proven by XPS measurements.^190^ Also theoretical studies investigated Ag segregation to the surface.^364^ A possible explanation is that surface-adsorbed oxygen drives the diffusion of subsurface Ag to the surface,^467^ a phenomenon which became especially apparent when applying an ozone treatment to the surface. It was found that a drastic change of the surface takes place. While Au and Ag are oxidized, Ag segregation to the surface is favored. Under reducing conditions, Ag eventually recedes back into the bulk.^17^

An ozone treatment is one of many procedures that was tried and carried out to activate NPG for catalysis, but this approach represents no universal measure to reliably activate NPG for the oxidation of different molecules.^433^ Activation of NPG is discussed in more detail in sections 4.2.1 and 4.3.1. These diverging results very much suggest that the specific surface chemistry needed to catalytically drive various partial or total oxidation reactions, respectively, is of different nature. This conclusion is also supported by the wealth of oxygen species and surface oxide phases that may form and (co)exist on Au surfaces.^388^ Overall, the question which activation treatment is most successful for a certain reaction has not finally been answered yet and will require further investigations in the future.

In essence, the mesoscopic and microscopic structure of NPG as well as the chemical distribution of residual LNE in the material are less defined than it may be assumed at first sight and are likely to considerably change in a catalytic environment. Hence, when NPG is characterized in terms of its structure and chemical composition, it should clearly be specified, at which stage the samples were characterized, how they were dealloyed, how they were treated (e.g., activated before catalysis), how long they were stored in which atmosphere at which temperature before use, and whether they were investigated in situ or ex situ. All of these different conditions might explain detected differences between different samples often reported in the literature.

## 5 Liquid Phase Aerobic Oxidation of Methanol

The previously discussed findings prompted us to focus the review on liquid phase catalysis with NPG on the aerobic oxidation of methanol and its correlation to mechanistic studies in the gas phase and electrochemistry (sections 4 and 6). Within this review, the term “liquid phase catalysis” will be used to describe thermally induced catalytic reactions and explicitly excludes electrochemical or electrocatalytical processes. Working in the liquid, i.e., condensed phase is the requirement for a widespread use in chemical synthesis and, hence, an important aspect. In fact, NPG has a much wider scope for organic transformations in the liquid phase which will be addressed in section 7.

### 5.1 Challenges for Working in the Liquid Phase

The description and understanding of chemical processes at the interface between the solid catalyst surface and a condensed phase is inherently more complex and more challenging as compared to the solid–gas interface. Yet reports suggest that a correlation to mechanistic studies at the gas/solid interface is possible,^58^ which is due to the noble character,^468^ i.e., a low propensity to chemisorb species on its surface.

There is a gap in between the degree of precision, with which solid–liquid interfaces can be characterized compared to the solid–gas counterpart. This is partially due to a lack of appropriate methodology for solid–liquid interfaces. As a result, an improved understanding of processes at the solid–liquid interface are a major focus in catalytic research.^469^ The limitations are mainly due to three reasons: (i) the interface is spectroscopically not easily accessible,^470^ (ii) the much higher concentration of reagents and the presence of solvent molecules,^471,472^ and (iii) the dispersion and diffusion of gaseous reagents such as oxygen within the reaction medium.^472^ Further aspects such as the formation of a charged double layer on the catalyst surface can play an important role also in liquid phase catalysis.^473^ When in contact with a liquid such as water, an electric field on the solid surface evolves as a consequence of the rates of all oxidative and all reductive partial reactions. The potential changes the chemical potential and thus the reactivity of species such as H^+^. This effect is well-known and established for electrochemical catalysis and corrosion, yet the effect is not as well documented in heterogeneous catalysis in liquids due to the difficulties in determining and predicting this potential.

In order to spectroscopically access the interface, the probes, typically photons or electrons, have to penetrate the dense liquid phase. This is already an issue when dealing with higher pressures of gases on the order of 0.1 mbar as operative in ambient-pressure XPS, yet, makes detection of electrons emerging from the solid–liquid interface very challenging.^474^ Spectroscopy at the solid–liquid interface is thus restricted to photons of various energies ranging from IR all the way to X-rays. Employing special reactor designs, those techniques can provide information on the catalyst surface *in situ* or *in operando*.^470,475,476^

The second reason why mechanistic insights from gas phase experiments cannot directly be transferred to the solid–liquid interface is the inherently much higher concentration of reagent and solvent molecules. For instance, 2 vol % of methanol in the gas phase at 1 bar total pressure corresponds to to a molar concentration in the range of 10^–3^ mol L^–1^, yet undiluted methanol in liquid phase catalysis results in a concentration of about 25 mol L^–1^, hence, about 4–5 orders of magnitude higher, which leads to a considerable increase in coverage. At first sight, a simple solution to this problem is diluting the reagents such as methanol with a solvent. Yet, a solvent cannot necessarily be considered an innocent spectator. In fact, it was demonstrated that a solvent can significant change the reaction rate and the reaction mechanisms. As will be discussed later, alcohol oxidation in liquid phase containing water provides an example, where the first step of the reaction pathway, the deprotonation of the alcohol, is likely to proceed already in solution^477^ and is very dependent on the pH of water-based solvents. Much effort is devoted to the description of the solid–liquid interface by computational methods,^471^ but its complexity remains to be a challenge.

Another challenge in aerobic oxidation catalysis in the liquid phase is the necessity to dissolve oxygen in the reaction medium. For instance, 1 bar of oxygen pressure at 298 K gives a concentration in the gas phase of about 40 mmol L^–1^. At equilibrium, the oxygen concentration in liquid methanol is on the order of 10 mmol L^–1^.^478^ Very similar concentrations and supposedly activities can be reached in the liquid phase by applying higher pressures of a few bars in an autoclave. However, the transfer rate of oxygen from the gas phase to the liquid phase strongly depends on the setup. For instance, it was demonstrated that bubbling O_2_ through the reaction medium makes a significant difference in the measured activity by up to an order of magnitude as compared to the application of an O_2_ atmosphere using a balloon.^58^ The diffusion coefficient of O_2_ in solution is about 3 orders of magnitude smaller than in the gas phase at the same temperature. Therefore, proper mixing and stirring may be very important to enhance the otherwise slow mass transport by forced convection.

### 5.2 Comparison of Mechanistic Aspects in the Aerobic Oxidation of Methanol in Liquid and Gas Phases

**Figure 24:**
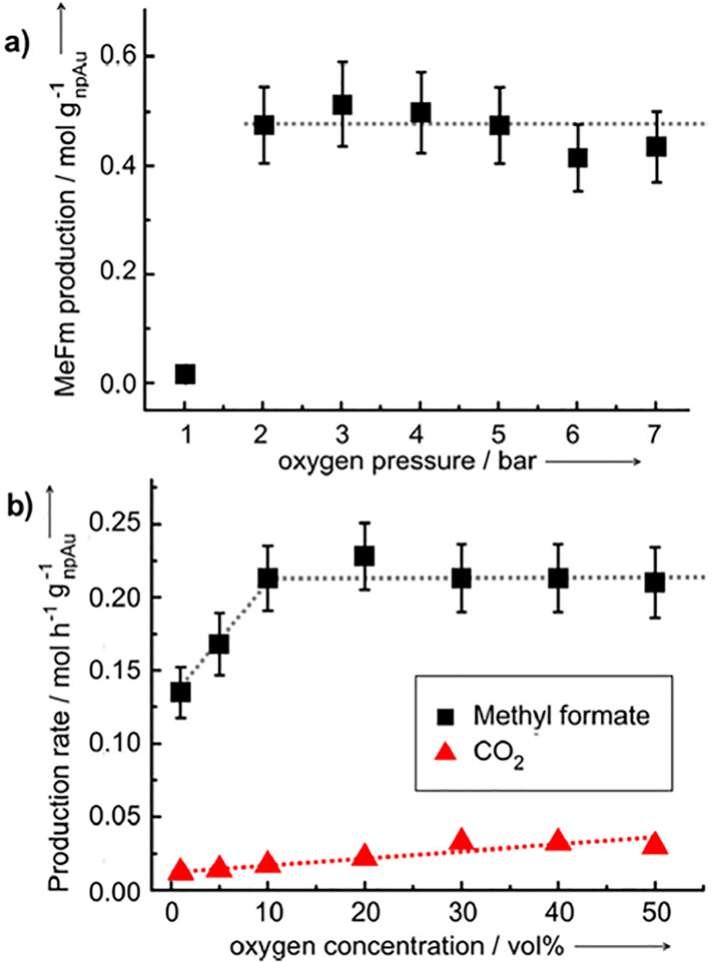
Aerobic oxidation of methanol to the corresponding ester (a) in the liquid phase at O_2_ pressures of more than 1 bar (batch reactor, after 24 h at 60 °C) and (b) in the gas phase employing a tubular flow reactor (at 60 °C). While the reaction is apparently zero-order for O_2_ in both cases, the onset of CO_2_ formation can be observed in the gas phase, indicating total oxidation and higher oxygen concentrations on the catalyst surface in the gas phase reaction. Reproduced with permission from ref (59). Copyright 2017 Elsevier.

Due to its more inert nature, methanol (MeOH) has been employed as solvent by Asao and co-workers,^479^ e.g., in the aerobic oxidation of secondary alcohols. However, MeOH itself can undergo oxidative conversion to methyl formate in the liquid phase at oxygen partial pressures above 1 bar (Figure 24).^59,480^ Here, MeOH is both solvent and reagent. For mechanistic studies, it would be beneficial to avoid an additional solvent, as this minimizes the number of species in the system and thus possible side reactions and may thus allow elucidating similarities between liquid and gas phase catalysis. The liquid phase aerobic oxidation of methanol was studied in a batch reactor.^59^ Similarly, as in gas phase experiments, the aerobic oxidation of methanol leads to the formation of methyl formate after an induction period in case the oxygen pressure was above 1 bar (Figure 24a) and the temperature between 40 to 80 °C. Taking into account that undiluted methanol (60 mL) and about 100 mg of catalyst was used, the observed conversion was in the range of a few percent. Such low conversions were chosen to reduce the influence of consecutive reactions on products such as water which accumulate in batch type reactions.

The formation of the corresponding ester is in line with gas phase experiments and can be understood by the reaction of the dehydrogenation of adsorbed methoxy and a subsequent coupling of the aldehyde with another methoxy species to form the ester by subsequent hydrogen abstraction reactions. But how does it depend on the oxygen concentration, i.e., the oxygen pressure? As can be seen in Figure 24a, the reaction proceeds only slowly at pressures of 1 bar, yet the rate steeply increases and levels of at pressures above 2 bar. The pressure independence indicates a zero-order kinetics with respect to O_2_ as also observed in the gas phase (Figure 24b). Hence, the catalysis is limited by the formation of reactive oxygen on the surface, i.e., the number of reactive surface sites on the surface and not on the proliferation from the gas phase. When plotting the conversion or yield as a function of time, the catalyst activity (rate of the catalyzed reaction) in a batch-type reaction can be determined. Often the rate of the catalyzed reaction is referred to the amount of active sites or more simply to surface sites. The resulting quantity is the TOF. The calculation of the TOF requires knowledge of the surface area of the catalyst and the area density of surface atoms, which can for example be determined by electrochemical methods (section 3.4).^304^ In this way, the formation of the product molecules per surface atoms and time is calculated as 380 h^–1^ at 80 °C in liquid phase. This value is indeed very comparable to the values obtained in gas phase experiments (396 h^–1^ at 80 °C).^59^ The observation of an apparent activation energy of 74 kJ mol^–1^ at temperatures below 330 K in the liquid phase and 61.5 kJ mol^–1^ in the gas phase for the partial oxidation pathway (in the limit of no mass transport limitation) exemplifies the very comparable activity of the NPG catalyst under both conditions.^59^

The most prominent difference under both conditions is the absence of the total oxidation pathway to CO_2_ in the liquid phase catalysis (cf., Figure 24a vs 24b) even at pressures up to 7 bar. This can be rationalized by the much higher concentration of methanol in the liquid phase. The total oxidation pathway requires the presence of a surplus of reactive oxygen on the catalyst surface (cf., Figure 17). It is likely that, due to the high concentrations of methanol close to the solid–liquid interface, any temporary surplus of oxygen reacts immediately with methanol, resulting in adsorbed methoxy as the predominant surface species which suppresses the total oxidation pathway. This situation can be circumvented by diluting simply with water and, hence, decreasing the concentration in methanol, but already concentrations of water on the order of 5 vol % reduced the observed conversion by more than 50%.^58^ A possible explanation is the formation of the geminal diol when scarce surface-bonded aldehyde reacts with water, forming the geminal diol of methanol instead of its reaction with a methoxy.^481^ This exemplifies the difficulty of using solvents to dilute reagents as they can also participate in the reaction pathways.

### 5.3 Influence of Residual Ag

In gas phase oxidation reactions as well as in theoretical calculations, the mole fraction *x*_Ag_^res^ of residual Ag in NPG was determined to be the decisive factor for the activation of molecular oxygen.^364,407,411,482^ The alloying of Au with Ag can change the electronic and geometric properties of the surface, also called ligand and ensemble effects, respectively.^483^ It can be anticipated that the presence of Ag also impacts the activity and selectivity for the aerobic oxidation in the liquid phase. This was studied for the partial oxidation of methanol and ethanol.^59^ In this context, the corrosion of NPG was modified and adapted to generate NPG with comparable ligament sizes and, thus, comparable specific surface area, but with varying concentration of Ag by employing a galvanostatic dealloying (GD) method (cf., section 2).^185^

**Figure 25:**
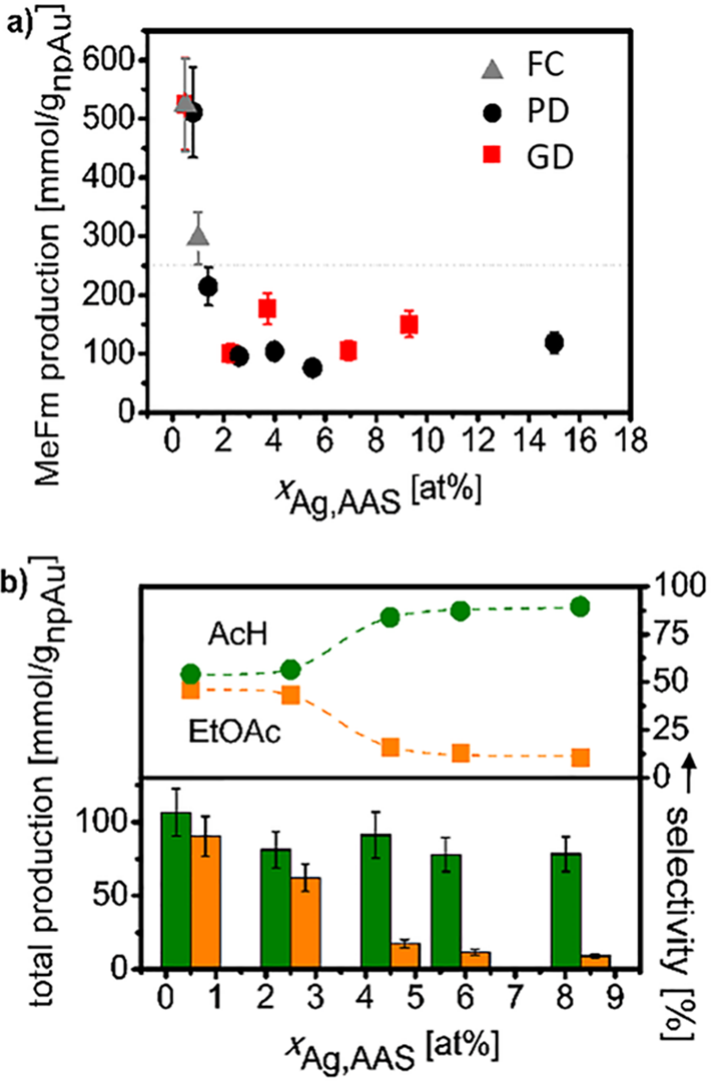
(a) Aerobic oxidation of methanol to methyl formate (MeFm) as a function of the total Ag content in the samples determined by atomic absorption spectroscopy (AAS) obtained by free corrosion, potentiostatic dealloying, and galvanostatic dealloying. (b) Partial aerobic oxidation of ethanol as a function of Ag content in NPG obtained by GD. The corresponding aldehyde and ester are formed in a ratio of 1:1 at low Ag contents of 1 at. %, yet aldehyde formation increases with increasing Ag content. Experiments were performed at 3 bar O_2_ pressure at 60 °C for 24 h. Reproduced with permission from ref (185). Copyright 2017 Royal Society of Chemistry.

In the case of methanol oxidation, the activity drastically dropped when increasing the overall Ag content to above *x*_Ag_^res^ > 1% (Figure 25), indicating that the surface of NPG becomes less active and even poisoned when larger fractions of Ag are exposed. This is quite in contrast to the CO oxidation in the gas phase.^390^ It is, however, difficult to align the total Ag content in the bulk with the concentration of Ag on the surface of the ligaments, which might be increased by a factor of 10 and more.^93^ Local Ag concentrations close to those of the master alloy, e.g., *x*_Ag_^res^ = 70%, are possible if such Ag-rich regions are exposed to the surface during catalysis (cf., section 3.13). It is reasonable that the likelihood for this scenario strongly increases with rising in the total Ag content.^93^ The impact of increasing the Ag content at the surface of ligaments up to *x*_Ag_^res^ = 60% was studied in gas phase experiments under UHV conditions by Friend and co-workers using bimetallic Au/Ag surfaces.^484^ The temperature of formation of methyl formate increases for mixed sites on Au sites being the lowest (200 K). Indeed, on Ag-like sites the temperature for formation of the methyl formate increased to 260 K. The formation of the aldehyde proceeded at even higher temperatures of 350 K on such sites. This is the reason why NPG becomes inactive at low temperatures for increasing content of Ag on the surface. Formation of CO_2_ was observed on all types of sites only at temperatures above 330 K and at higher oxygen coverages (i.e., above 0.05 ML), which are presumably not achieved in liquid phase catalytic conditions.^484^

### 5.4 Roles of Acid and Base, and Activation Period of the Catalyst

**Figure 26:**
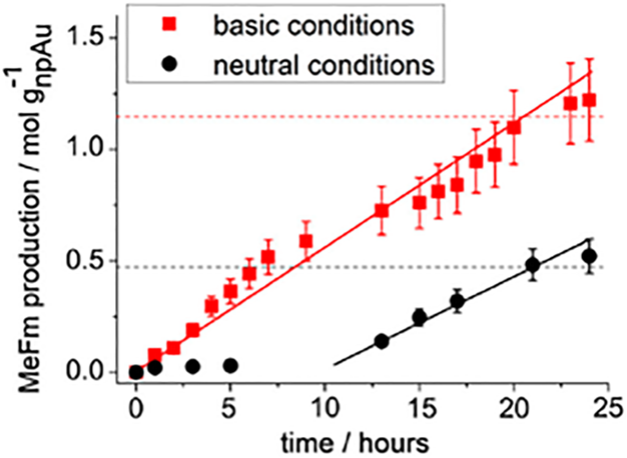
Methanol oxidation at 60 °C and 3 bar O_2_ as a function of reaction time. Temporal evolution of MeFm formation under neutral as well as basic (12 mmol L^–1^ KOH) conditions. Reproduced with permission from ref (480). Copyright 2019 The Authors License CC-BY-4.0.

Several reports on the aerobic liquid phase oxidation of alcohols such as benzyl alcohol^485^ or glucose^60^ indicate that the conversion is considerably increased by adding catalytic amounts of a base. One aspect is that the reaction in solution, e.g., the abstraction of the alcoholic proton by OH^–^ can speed up the oxidation on the catalyst surface. In the case of methanol oxidation with NPG catalysts, an increase of conversion by more than 100% was observed after 24 h.^480^ The product distribution was not changed, indicating a similar reaction pathway in neutral and alkaline solutions.^480^ While the rates and activity of the catalyst (slope of the lines in Figure 26) remained similar, a strong impact of the added base on the activation period of the catalyst was noted, that is, the time after which a conversion of methanol to methyl formate was detected (Figure 26). Such an activation period is usually ascribed to the removal of advantageous species blocking surface sites and the generation of a catalytically active state, as for example surface oxides, and the concomitant migration of Ag to the surface. In the absence of hydroxyl, reactive oxygen must be generated by adsorption of O_2_ on free Ag sites on the surface. By transient adsorption of OH^–^, however, electrons are provided which then can be used to generate an O^2–^ species from O_2_ also on available Au surface sites (*):^480^
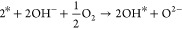
15

The reaction of O^2–^ with, for example, Ag sites leads to the formation of an oxide, which in turn releases OH^–^ into solution, ensuring electroneutrality [eq 16].
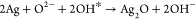
16

This explanation is supported by the observation that the conversion of methanol at NPG commenced without delay associated to an activation period in the presence of catalytic amounts of OH^–^.^480^ Simply, the entire Au- and Ag-like surface can be activated for reaction with O_2_ in the presence of OH^–^. This is a unique feature of liquid phase catalysis, in which ionic species such as OH^–^ can be facilely employed.

## 6 Nanoporous Gold as Electrode Materials

While the preparation of NPG by dealloying is inherently an electrochemical process as discussed in section 2, the applications discussed in sections 4 and 5 are not electrochemical. Section 6 deals with the use of NPG as an electrode material, at which reactions *other* than dealloying are conducted. While NPG is an active electrode during dealloying, i.e., an electrode from which material is dissolved, NPG is mainly used as inert electrode material in electrochemical application very much like conventional planar gold electrodes. The term “inert electrode” in the context of electrochemistry refers to an electrode that supplies/accepts electrons to/from reactants dissolved in an adjacent liquid electrolyte solution but does not change its own mass by electrolytic dissolution/deposition.

The electrode reactions are often complicated multistep reactions involving adsorbed intermediates and coupled homogeneous reaction steps in solution. Reactants that adsorb at the electrode surface compete for adsorptions sites with anions, cations, and sometimes solvent molecules of the electrolyte. The stabilization of reaction intermediates at the electrode surface may accelerate otherwise slow reactions and is often called (heterogeneous) electrocatalysis due to the similarity to heterogeneous catalytic processes covered in section 4. Consequently, the reactions involving adsorbed intermediates tend to show a specific dependence of the precise surface composition and structure of the adsorption site. Electrodes that exhibit a particularly fast rate of a specific reaction are also called “electrocatalytically active”, a terminology that should not be confused with “active dissolution” as an important feature during the preparation of the NPG electrode itself.

Homogenous electrocatalysis as the analogue to homogeneous catalysis exploits a molecularly defined reaction center (transition metal complex, enzyme,...) with which reactants interact. The electrode then supplies electrons to or accepts electrons from such a reactions center rather than directly to/from the adsorbed reactant. Both forms of electrocatalysis can benefit from the tunable properties of NPG. Next to a dedicated chapter in the book on NPG,^36^ the application of NPG electrodes in the sense described above has recently been covered in several specialized reviews.^25,63,69,486,487^

### 6.1 Comparison of Nanoporous Gold to other Gold Electrodes

NPG shares all properties with planar Au electrodes that are directly linked to the properties of metallic gold as the main constituent of NPG. This includes, for instance, the applicable potential ranges in different electrolyte solutions as well as specific adsorption of HSO_4_^–^, halide, and pseudohalide anions (I^–^, Br^–^, Cl^–^, CN^–^, OCN^–^, SCN^–^) and chemisorption of sulfur-containing compounds.^488−490^ F^–^ and ClO_4_^–^ are anions that do not chemisorb on Au and are therefore preferred anions for the characterization of the electrical double layer in the absence of specific adsorption.^488^ Commonly used electrolytes with HSO_4_^–^/SO_4_^2–^ form complicated potential-dependent structures of adsorbed ions on gold.^491,492^ Many cations such as Pb^2+^, Bi^3+^, Ag^+^, and Cu^2+^ interact specifically with NPG and planar Au by a process called underpotential deposition/stripping (UPD).^493,494^ Such ions form a monolayer of (partially) reduced metal ions at a foreign substrate (e.g., Pb^2+^ on Au) at potentials more positive than the thermodynamic potential of the electrolytic deposition of this ion on its own metallic substrate (e.g., Pb^2+^ on Pb). The UPD process is very sensitive for specific sites, and we will consider it in detail in section 6.1.3.

Besides similar material properties, there are important differences between NPG and planar gold electrodes such as the specific surface area, the density of low-coordinated surface atoms as well as the occurrence of low amounts of LNE in NPG that have not been removed completely in the dealloying process (sections 2 and 3). Mass transport is very often a limiting factor in electrochemical reactions because diffusion in liquid electrolytes is orders of magnitude slower than in gas phase. For a porous electrode with a bicontinuous distribution of metal and electrolyte phases, mass transport limitation may arise from the transport of the reagents to the outer parts of the porous electrode, similar to the situation at a planar electrode. This transport mode is called external mass transport. In order to utilize the huge surface area of NPG, it is important that reagents reaching the outer parts of an NPG electrodes are distributed within the pore network to the inner surface area of the electrode by a process termed internal mass transport. This general consideration applies to diffusive mass transport as well as to the transport of ions by migration.

#### 6.1.1 Comparison of Surface Voltammetry of Au Electrodes

Besides the determination of surface area covered in section 3.4, surface voltammetry of noble metal electrodes can reveal details about surface facets, defects, and specific adsorption of ions. The signals in surface voltammograms of gold result from the chemisorption of OH^–^, the formation of a surface oxide layer, whose completion can be clearly identified by a minimum in current–voltage curves, called the Burshtein minimum, which is well identified at around 1.65 V (RHE) in Figure 10a.^306^ At the potential of this minimum, exactly a two atom-thick “Au^II^O” layer is formed.^495,496^ Other processes, such as exchange or reorientation of ions in the double layer, are associated with capacitive currents. They can be of diagnostic value if very clean electrodes and electrolyte solutions are used.^363^ However, quite often the exact nature of a specific signal is not clear because the structure–signal relationship are derived from combinations of voltammetry and structure-sensitive methods at planar single-crystal electrodes^491,492^ that lack the multitude of low-coordinated surface atoms that are characteristic of NPG. For nanoporous gold, the voltammetric signals from different facets with their individually distinct defect types overlap. An assignment to structural features of the surface is typically made by a “fingerprint” method, in which the voltammogram at NPG is considered as a superposition of contributions from single-crystal faces. Thus, characteristic voltammetric features are compared to voltammograms obtained at single-crystal electrodes under the same conditions (especially in the same electrolyte solution). An authoritative source of single-crystal voltammograms in acidic and neutral electrolytes is available from the work of Hamelin.^307,497^ This source also contains an in-depth discussion of experimental problems. For alkaline electrolytes, the data of Feliu and co-workers are recommended for a comparison.^498^ For convenience, recalculation of the potential of common reference electrodes is detailed in SI, SI-2, examples of single-crystal voltammograms from refs (307 and 498) are reproduced in the SI, SI-3, Figures S4 and S5. Further sources of single-crystal voltammetry are listed in SI, SI-3, Table S4.

#### 6.1.2 Tuning of Internal Surface Structure as Followed by Metal Underpotential Deposition/Stripping

**Figure 27:**
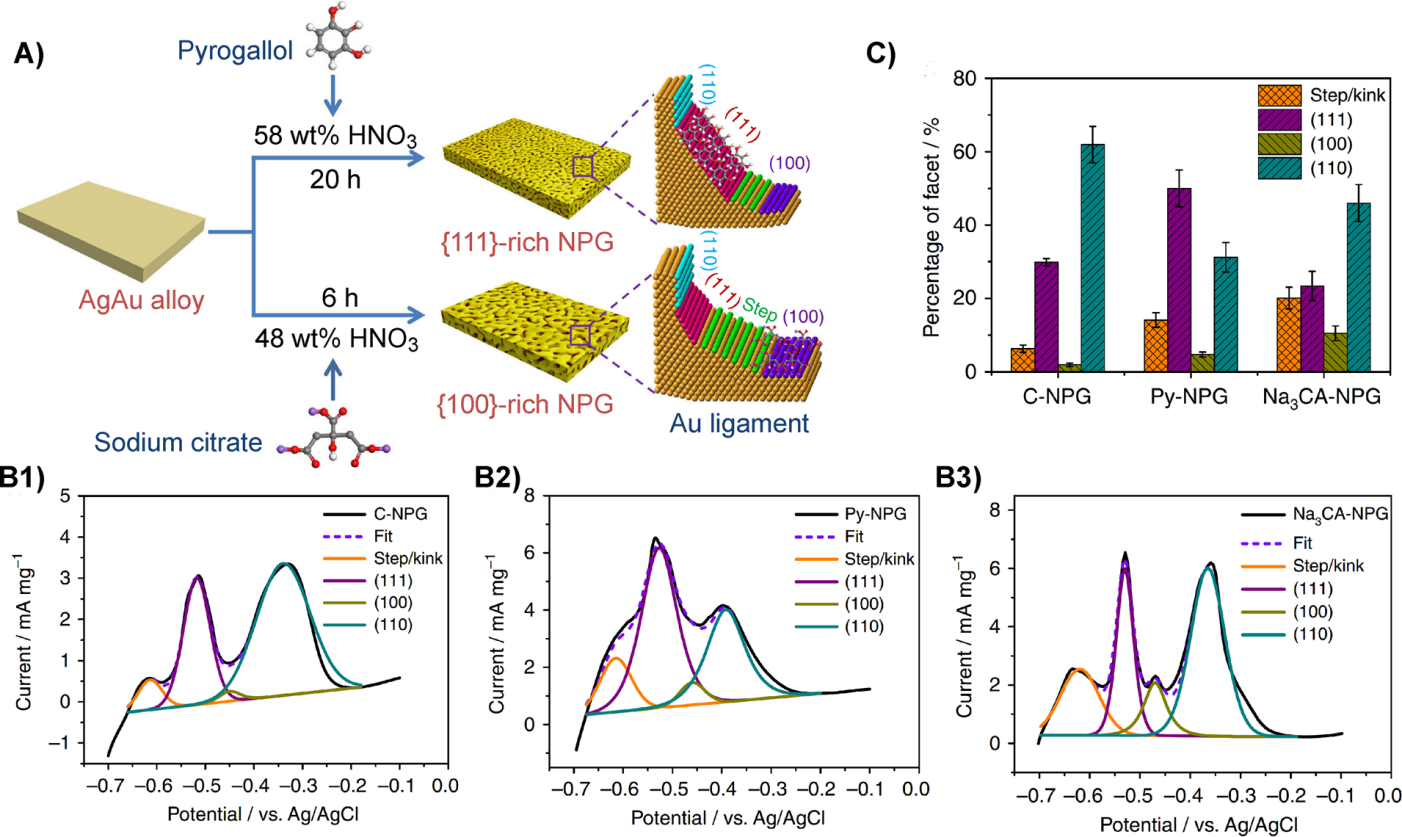
(A) Schematic representation of the internal surface of NPG by free corrosion in the presence of surfactants. (B) voltammetric scans for the stripping of a Pb UPD layer in 10^–3^ mol L Pb(NO_3_)_2_ + 0.1 mol L^–1^ NaOH, *v* = 50 mV s^–1^ with fits of the UPD signals for low-index crystal facets: (B1) from Ag_65_Au_35_ in 58 wt % HNO_3_ at room temperature for 3 h; (B2) from Ag_65_Au_35_ in 58 wt % HNO_3_ + 0.2 mg mL^–1^ pyrogallol at room temperature for 20 h; (B3) from Ag_75_Au_25_ in 48% HNO_3_ + 2.0 mg mL^–1^ sodium citrate at room temperature for 6 h. (C) Comparison of the proportions of the different facets as determined from the area of the Pb UPD peaks in (B1–B3). Reproduced with permission from ref (339). Copyright 2017 Springer Nature.

Besides surface voltammetry in common electrolyte, the observation of UPD and stripping of metal adlayers is very sensitive to specific features on surfaces.^493^ Pb^2+^ yields characteristic UPD signals, both on Ag and Au electrodes and has been particularly often applied as structure-sensitive probe reaction. The scientific basis for using this system rests in the observation of an electrosorption valency close to the charge of Pb^2+^, the absence of surface alloying under electrochemical conditions and the relatively small effect exerted by water and anions on the particular UPD process of Pb^2+^ on Au facets.^499^ The UPD signals occur at different potential for the different single-crystal faces due to electronic interaction between the Au substrate and Pb^2+^ at the specific sites and possible superstructures of the UPD layer on a specific face of the Au substrate. Generally, more than one signal is observed even for well-prepared single-crystal electrodes. The signals correspond to the transitions between different superstructures of the adatom on the substrate.^499^ With this in mind, it is clear that the commonly used fitting of Pb UPD signals on NPG with a low number of peaks clearly ascribed to one low-index facet (as in Figure 27B1, B2, and B3) is a very coarse approximation. For convenience and to illustrate this point, voltammograms of Pb UPD on single-crystal Au are compiled in SI, SI-4, Figures S6–S12. The charge corresponding to the different peaks is proportional to the surface area of the specific facet within the NPG structure.

This method can be applied both in acidic as well as in alkaline solution.^499,500^ Pb^2+^ ions added, e.g., as Pb(NO_3_)_2_, in precisely weighted amount to the alkaline electrolyte solution are immediately converted to plumbites [Pb^II^(OH)_*m*_]^(2–*m*)–^ (*m* > 2), which dissolve in alkaline solution up to millimolar concentrations. Pb^2+^ stripped off the surface by the oxidation of a Pb UPD layer dissolves in acidic electrolytes. The stripping of an Pb UPD layer in alkaline solution forms [Pb^II^(OH)_*m*_]^(2–*m*)–^ that remain at the surface as specifically adsorbed anion. This fact can be used for selectively blocking surface structures for electrocatalysis.^107^

Using Pb UPD signals to characterize the internal surface structure, it became evident that tuning of this internal surface structure is possible by adding surfactants while forming NPG by free corrosion in HNO_3_ solution (Figure 27A).^339^ Pyrogallol formed an inner surface enriched in {111} facets, and addition of citrate promoted the formation of {100} facets (Figure 27B1, B2, B3, and C). The different ratios of facets in NPG materials strongly correlated with different activities for methanol oxidation reaction (MOR) in 0.5 mol L^–1^ KOH with the highest activity found for the {111}-rich internal surface formed in the presence of pyrogallol.^339^

The internal surface structure of NPG can also be tuned by a post-dealloying method. Cycling NPG in 0.1 mol L^–1^ H_2_SO_4_ in the potential range of surface oxide formation and reduction lead to a restructuring of an initially amorphous surface to a {111}-rich internal surface, when the cycling was conducted at a low potential scan rate of *v* = 5 mV s^–1^, whereas a {100}-rich internal surface was obtained by cycling at 50 mV s^–1^ in the same electrolyte.^462^

**Figure 28:**
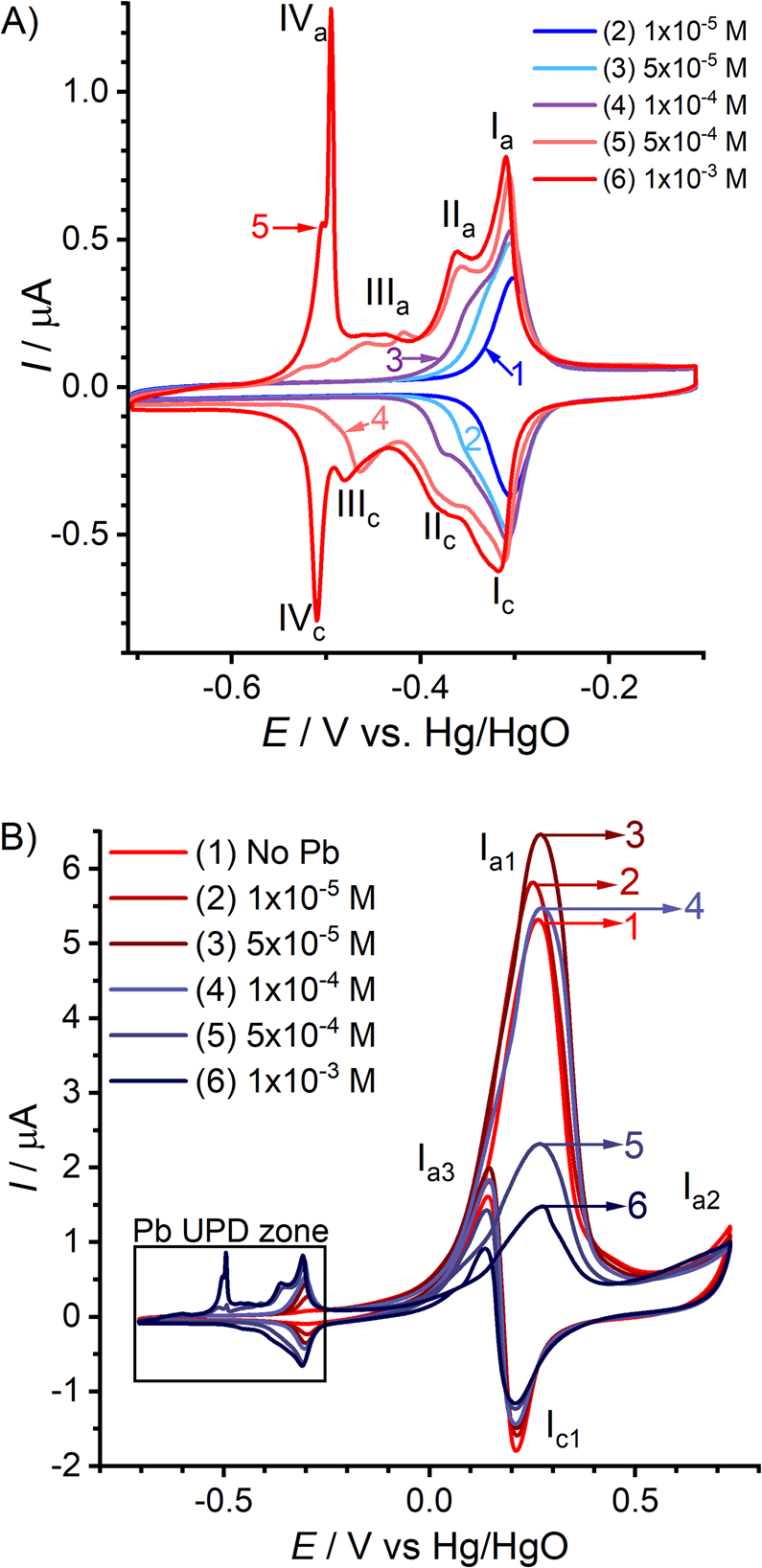
(A) Cyclic voltammograms of NPG *partially* covered by a Pb UPD layer (5th cycle, *v* = 10 mV s^–1^) in 0.1 mol L^–1^ KNO_3_ + 0.1 mol L^–1^ KOH with addition of different concentrations of Pb(NO_3_)_2_ (converted to [Pb^(II)^(OH)_*m*_]^(2–*m*)–^). (B) Cyclic voltammograms of methanol electrooxidation at NPG partially covered by Pb UPD or adsorbed [Pb^(II)^(OH)_*m*_]^(2–*m*)–^ (*v* = 10 mV s^–1^) in 1 mol L^–1^ MeOH + 0.1 mol L^–1^ KNO_3_ + 0.1 mol L^–1^ KOH with different concentration of [Pb^(II)^(OH)_*m*_]^(2–*m*)–^). The surface is covered by specifically adsorbed [Pb^(II)^(OH)_*m*_]^(2–*m*)–^ anions in the potential range of MOR. The bulk concentrations of [Pb^(II)^(OH)_*m*_]^(2–*m*)–^ in mol L^–1^ are (1) 0, (2) 1 × 10^–5^, (3) 5 × 10^–5^, (4) 1 × 10^–4^, (5) 5 × 10^–4^, (6) 1 × 10^–3^ in (A) and (B). Reproduced and adapted with permission from ref (107). Copyright 2021 The Authors under license CC-BY.

It is key that the cycling is conducted in acidic solution because residual Ag, that tends to enrich at the surface during storage in air or during cycling in alkaline solution, may impede the restructuring because this process does not occur in 0.1 mol L^–1^ KOH solution.^191^ However, conducting one cycle in 0.1 mol L^–1^ H_2_SO_4_ removes the surface-enriched Ag (and Ag oxides) and allows restructuring. The restructuring can be confirmed by the observation of the signals from low-index crystal facets by surface oxidation^191^ and Pb UPD.^107^ After stripping of UPD-Pb, [Pb^II^(OH)_*m*_]^(2–*m*)–^ (*m* > 2) remains at the surface as specifically adsorbed anions or as PbO_2_-like species at potentials more positive than 0.25 V (Hg|HgO).^107^ Using a small amount of NPG in a cavity microelectrode (Figure 6) and adjusting the bulk concentration of plumbites in 0.1 mol L^–1^ KOH facilitated a very controlled partial coverage of the internal facets in NPG. By increasing the plumbite concentration in the bulk solution by adding more Pb(NO_3_)_2_ to the alkaline electrolyte, the facets were sequentially filled up in the sequence of their energetics for the Pb UPD (Figure 28A).^107^ Also in this case, the {111} facets were crucial for MOR: When the {111} facets were covered by Pb species, the current maximum at 0.25 V (Hg|HgO) dropped significantly (Figure 28B), whereas the onset of MOR shifted to a more positive potential already with the coverage of defects and {100} and {110} facets (Figure 28B, inset).^107^

Pb UPD also showed that NPG obtained by roughening pure Au electrodes by cycling in Cl^–^-containing electrolytes exhibits a morphology very similar to that obtained by dealloying (Figure 5C). In both cases, {111} facets dominate the internal structure of the material.^20^ Such intentional roughening procedures for Au electrodes are commonly applied and result in specific electrocatalytic reactivities.^501−504^

**Figure 29:**
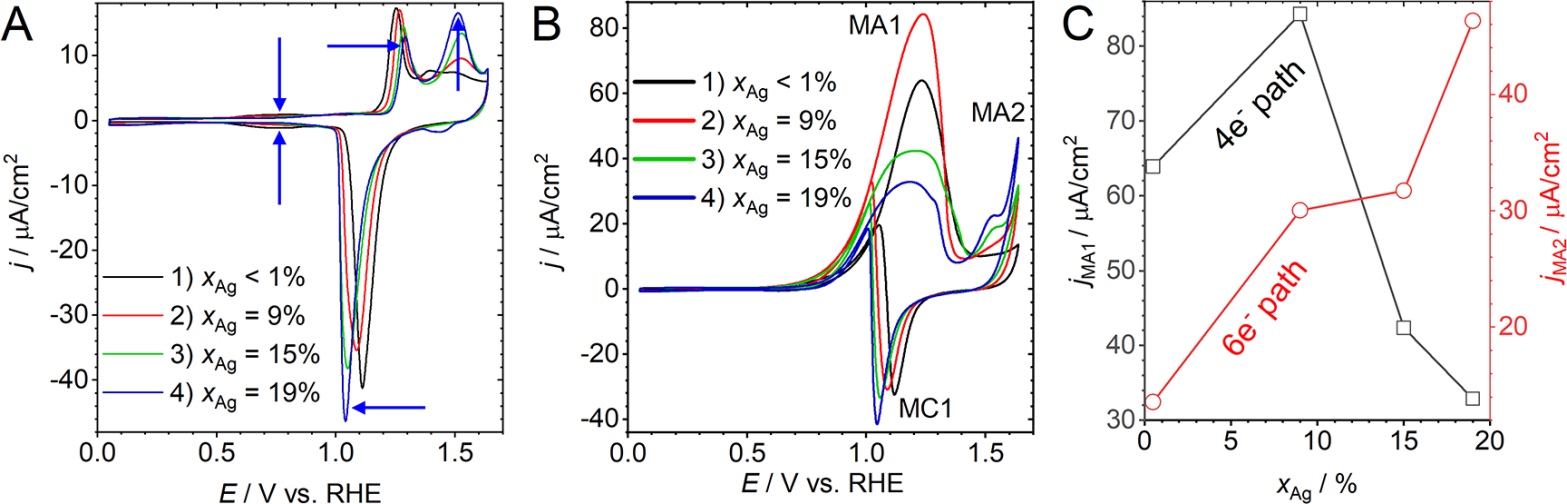
Effect of UPD-Ag at the surface of NPG. (A) Cyclic voltammograms in 0.1 mol L^–1^ NaOH at *v* = 10 mV s^–1^ with variable amounts of UPD-Ag. The legend indicates *x*_Ag_^res^ as determined by XPS. (B) Cyclic voltammograms of the same electrodes in 1 mol L^–1^ MeOH + 0.1 mol L^–1^ NaOH at *v* = 10 mV s^–1^. (C) Maximum current densities of signals MA1 and MA2 in (B) as a function of *x*_Ag_^res^ (as determined by XPS). Reproduced with permission from ref (505). Copyright 2022 American Chemical Society.

Ag (and Cu) also show structure-sensitive UPD processes onto NPG.^313^ However, the UPD process of Ag (and Cu) on Au is complicated by the tendency of Ag to alloy with Au.^313^ Such surface alloys form at room temperature in diluted acids on a time scale of 5–45 min.^313^ This has been used to modify the amount of Ag on the surface after dealloying *without* affecting the bulk Ag content in the ligaments (Figure 29A).^505^ Ag UPD layers on NPG have only a modest effect on the MOR peak current, corresponding to the 4e^–^ oxidation of methanol to formate (Figure 29B). The onset of the peak is shifted to less positive potentials with higher Ag surface concentration.^505^ However, the peak for the direct 6e^–^ oxidation of methanol to CO_2_ at more positive potentials of *E* > 1.5 V (RHE) significantly grows with a higher Ag surface concentration (Figure 29C). In this potential range, Ag is present as AgO-like species as detected by XPS.^505^

#### 6.1.3 Model Reactions that Show Differences to Planar Au Electrodes

Differences in the reactivity of clean planar Au electrodes and NPG have been described for a number of electrocatalytic reactions. Some could be related to specific surface structures as described in section 6.1.3 for the MOR. Another structure-sensitive reaction is the CO_2_ reduction reaction (CO_2_RR).^503^ This particular reaction does not only depend of the available facets that influence the energetics and geometries of adsorption sites for reaction intermediates but also on the mesostructure of the material.^250^ For instance, the selectivity of CO_2_RR can be favored vs the hydrogen evolution reaction (HER) by exploiting limitations of the internal mass transport (Figure 6A) in thicker NPG layers where the increased alkalinity helps to suppress the HER.^250^

The oxidation of ascorbic acid to dehydroascobic acid proceeds at solid electrodes by an EC_1_ mechanism, i.e., an electron transfer step (E) is followed by a homogeneous first-order decomposition reaction (C_1_) of the initially formed product to dehydroascorbic acid. The relative rates of the E and C_1_ steps depend on the internal mass transport in the pore system, which could be modified by changing parameters in the preparation of the porous Au electrodes using the H_2_ bubble templating method.^506^

#### 6.1.4 Structure Evolution of NPG during Use as Electrode Material

Besides the intended modification of the internal surface structure (section 6.1.3), the surface structure may also change unintentionally during application of the electrode. As the NPG structure is a metastable state, a trend toward formation of larger ligaments and pores is expected. Most often, this is noted by a decrease of the electrochemical surface area *A*_ECSA_ over time. However, the available reports arrive a quite different conclusion about the morphological stability of NPG during potential cycling or during electrocatalytic reactions. First reports showed a decrease of *A*_ECSA_ during continuous cycling for MOR in alkaline solution.^61^ Others reported stability of electrodes for MOR in alkaline electrolyte for NPG obtained by alloying/dealloying in an ZnCl_2_-benzylacohol electrolyte.^62^ As has been described in sections 2.1 and 4.4, the coarsening can be prevented or significantly retarded by the use of ternary alloys that contain a small fraction of an element with lower surface mobility, e.g., Pt,^99,112−114^ or by decorating NPG with Pt.^61^ A layer of Pt on NPG can also be obtained after dealloying by first forming a Cu UPD layer and then using a galvanic replacement reaction of Cu for Pt. The resulting material was structurally stable and had a high oxygen reduction reaction activity due to the Pt layer.^507^ Galvanic replacement of Pt for Cu has also been used to decorate Cu-containing porous materials obtained by bubble-templated electrodeposition.^18^ Here again, enriching the surface in Pt significantly increases the stability compared to pure porous Au.^18^ A similar process has also been demonstrated by first forming a Pb UPD layer and then using galvanic replacement of Pb for Pt to form a thin catalyst layer.^208^

There have also been reports that NPG can be obtained by pulsed electrochemical dealloying by which the ligament size can be tuned. Materials with an average ligament size of 8 nm showed the highest activity for glucose oxidation in neutral buffer but also the highest stability.^216^ During storage, the surface composition was changing. For NPG produced from AuAg alloys, Ag is enriched at the surface (determined by X-ray photoelectron spectroscopy (XPS)) relative to the bulk ligaments (determined by EDX).^190^ The enrichment factor *x*_Ag, XPS_^res^/*x*_Ag, EDX_^res^, is decreasing with the overall content of residual Ag.^190^ The stability of those materials during electrochemical cycling depends very much on the details of the cycling condition.^191^ If the material is cycled in alkaline solution only after an intermediate storage in air, the material is stable and shows the characteristic anodic peak A2 at *E* = 1.5 V (RHE) in Figure 29A. However, a short cycling in H_2_SO_4_ removes surface Ag as shown by XPS.^191^ Afterward, coarsening sets in, as observed by a decrease in *A*_ECSA_. A transfer back to an alkaline solution after short cycling in H_2_SO_4_ shows that the surface oxidation process A2 in Figure 29A is lost and does not recover during short cycling experiments.^191^

Material obtained by dealloying an amorphous starting material Au_40_Cu_28_Ag_7_Pd_5_Si_20_ becomes not only nanoporous but also nanocrystalline.^508^ In other words, it develops a polycrystalline microstructure with a grain size of nanometers, comparable to the ligament size. The intercrystalline regions are amorphous. The material is very active for MOR but loses the activity rapidly.^508^ NPG obtained by dealloying from the same metallic glass in short time (10 min to 1 h) also showed a rapid decay of catalytic activity for MOR in alkaline solution.^509^ NPG, which was obtained by first assembling a layer of Au nanoparticles in a Langmuir trough and subsequently interconnecting them by electroless deposition of gold, yielded a freestanding membrane.^21^ However, the voltammetric signals for MOR in alkaline solution rapidly decayed, in agreement with the absence of less mobile atoms that stabilize the high energy structures.^21^

### 6.2 Electrocatalysis of Model Reactions

NPG has been tested as an electrocatalyst for a variety of reactions that include the reaction for which platinum group metals are typically used in practical application. Besides costs, potential advantages of NPG are the ease by which a porous structure with tuned mass transport conditions can be formed without the need of a support material, such as carbon, that is susceptible to corrosion itself. Gold is chemically stable in a wide range of aqueous solutions. However, mass-related and area-related activity remain below that of Pt and Pt alloys. Furthermore, the stability of the NPG morphology as a metastable structure must be sustained over long periods. This has turned out to be challenging and, therefore, the use of NPG as electrode material for sensors has received more attention. The requirement for operation stability over very long times is relaxed for sensors, and aspects of system integration are of higher importance than mass-related or area-related activity.

#### 6.2.1 Hydrogen Evolution Reaction

Hydrogen evolution reaction (HER) is one of the simplest electrocatalytic reactions that is often used to compare different electrode materials. Reaction rates, represented by exchange current densities or by heterogeneous standard reaction rate constants of hydrogen evolution on different metals vary by several orders of magnitude and are often represented as volcano plots.^510,511^ Besides the scientific interest in HER, it is an important reaction in water electrolysis that currently receives revived attention. Due to the position of Au in the volcano plots for HER, early approaches to utilize NPG for HER resorted to modification of the surface of NPG by a monolayer of Pt using galvanic exchange reactions.^512^ Interestingly, some recent works used porous Au electrodes without Pt-group elements for HER: Islands of porous Au obtained by bubble-templated electrodeposition and thus without remaining LNE showed stable operation and impressive area-related HER currents.^254^ Similar performance was obtained by electrochemically roughening of a planar gold electrode.^513^ The procedure involves oxidation of the surface at 2.0 V (RHE) in 0.5 mol L^–1^ H_2_SO_4_ followed by electroreduction of the optically visible gold oxide film.^513^ The control and efficacy of NPG preparation by anodization of massive Au electrodes can be refined by exploiting the effects of specifically adsorbed anions in the anodization solution that increased the ratio of {111} vs {100} facets in the resulting material.^20^ The increased fraction of {111} facets was given as the main reason controlling the high activity (3 mA cm^–2^ at −0.049 V (RHE) in 0.5 mol L^–1^ H_2_SO_4_),^20^ because experiments on planar Au single-crystal electrodes had shown a 2.3 times higher activity for the {111} facet compared to the {100} facet.^514^ Efficient catalysts have also been prepared by forming an aerogel of interconnected Au nanoparticles on carbonitride membranes that sustained an HER current density of 10 mA cm^–2^ at −0.185 V (RHE).^515^

#### 6.2.2 Oxygen Reduction Reaction and Other Oxygen Redox Chemistry

Oxygen reduction reaction (ORR) at planar gold electrodes proceeds much slower than at Pt and with a considerable formation of hydrogen peroxide H_2_O_2_.^516^ H_2_O_2_ causes subsequent material degradation in electrochemical cells. Although its formation may be intended, it is mostly an undesired byproduct in fuel cell or energy applications or when ORR is used as anodic reaction for a synthetic reduction reaction. The best catalysts for oxygen evolution reaction (OER) are IrO_2_ and RuO_2_.^517^ As the availability of Ir and Ru is even more critical than that of Pt, there is an intensive search for alternative electrode materials for OER.

Plating Pt on NPG is an obvious application scenario for NPG in the context of ORR electrocatalysis and has been demonstrated very early.^518^ In this work, Pt was deposited at different loading between coverages of a submonolayer, a wetting monolayer, and as nanoparticles on a monolayer.^518^ Pt-containing NPG has also been obtained by dealloying ternary alloys such as Pt_1_Au_0.5_Cu_98.5_^224^ (see section 2.2) or by dealloying a AuAg alloy and modifying the resulting NPG by Cu UPD followed by a galvanic exchange reaction of Pt for Cu.^507^ By repeating Cu UPD on the first Pt layer followed by another galvanic exchange reaction, the amount of deposited Pt could be increased in a defined way above a monolayer coverage.^507^ Those electrodes showed very good performance and stability even when compared to commercial Pt/C catalysts. A key feature is the low amount of formed H_2_O_2_ and the better adhesion of Pt nanostructures to Au compared to the adhesion of Pt nanoparticles to conventional carbon supports.^507^ Oxygen evolution has been demonstrated with Pt-modified and Ir-modified anodized porous Au.^519^

While the results on Pt-modified NPG may not be entirely unexpected, it was reported rather early that Pt-free NPG also shows a remarkable ORR activity and that H_2_O_2_ formation known from planar Au electrodes is effectively suppressed.^338^ The increased catalytic activity is due to the higher fraction of low-coordinated surface sites in NPG relative to planar Au. The reaction at NPG as well as at Au occurs by two sequential 2e^–^ steps (as opposed to the 4e^–^ route on Pt). At NPG, the second step, namely H_2_O_2_ reduction, is very efficient, probably further supported by the confinement of H_2_O_2_ in the pore space of NPG.^338^ NPG obtained from metal-induced crystallization and thus without LNE showed stable ORR activity in alkaline solution, which was higher than the activity of NPG the authors obtained from dealloying.^520^ Other preparation methods that are more suitable for sensors like anodization at 2.0 V (Ag|AgCl) and subsequent electroreduction of gold oxides yielded linear calibration curves for ORR currents over a wide range of O_2_ content in neutral solution.^225^ Aerogels of interconnected Au nanoparticles on carbonitride membranes showed high onset potentials for ORR in alkaline and acidic electrolytes.^515^ The performance was dramatically increased by forming aerogels from AuCu alloy nanoparticles directly during the synthesis.^521^ Those aerogels not only outperformed Au aerogels in ORR (0.906 mA cm^–2^ at 0.85 V (RHE) for the optimum composition Au_52_Cu_48_) but also Pt/C catalysts (0.203 mA cm^–2^).^521^ Another advantage is the high methanol tolerance of the AuCu aerogels compared to Pt/C catalysts.^521^

The use of NPG for ORR in *nonaqueous* metal–air batteries is covered in section 6.5.2.

#### 6.2.3 Alcohol Oxidation Reaction

As discussed in section 6.1.3, alcohol oxidation, especially methanol oxidation, is very sensitive to surface structures. Its therefore often used as a model reaction to gain insights in structure–reactivity relations at electrocatalytically active electrodes.^522^ The oxidation of methanol can be conducted in acidic and alkaline solutions. Koper and co-workers^523^ attributed the higher activity of the alcohol oxidation reaction in alkaline media to a homogeneous deprotonation of the alcohol preceding its adsorption although some interactions with the (planar) gold surface is necessary for the reaction to occur.
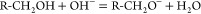
17The oxidation proceeds at the bare metallic surface in the potential range 0.9–1.35 V (RHE) (Figure 29B, signal MA1). At more anodic potentials, the current drops when the surface oxide layer is formed at the electrode surface. At potentials more positive than the Burshtein minimum, the oxidation commences again and may overlap with oxygen evolution (Figure 29B, signal MA2). In principle, several oxidation reactions are possible, each composed of several elementary reactions.^522^
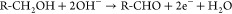
18
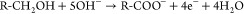
19
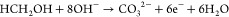
20The 2e^–^-oxidation leads to the aldehyde RCHO. In the case of MeOH as reagent, the product formaldehyde enters the Cannizzaro reaction 21 in alkaline solution, in which the primary oxidation product disproportionates to MeOH and formate.
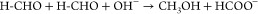
21The Cannizzaro reaction is preferred only if there is no H-atom in α-position to the carbonylic C-atom; thus, it does not occur for ethanol. However, the aldehydes of higher alcohols are also unstable in alkaline solution, especially in the presence of dissolved oxygen.^523^ Thus, product analysis may underestimate the amount of aldehyde formed in the electrode reaction.

The reactivity in the different potential regions is influenced by the number of low coordinated sites, the crystal facets exposed to solution and the content of LNE that remains from the dealloying process or is purposefully deposited on the surface of the ligaments. However, the influence of those factors is very different in the different potential regions. The onset of the methanol oxidation current is shifted to less positive potentials in agreement with the role of Ag (Figure 29B, signal MA1).^107^ However, the peak current at 1.2 V (RHE) decreases when the Ag content increases above an optimum value. At potentials more positive of the Burshtein minimum (1.35–1.45 V (RHE) in Figure 29B), no pronounced peak for methanol oxidation is observed for NPG with *x*_Ag_^res^ < 1%, whereas there is a clear signal MA2 if Ag is present at the surface of the ligaments at *x*_Ag_^res^ > 4%, either as remaining from the dealloying process^107^ or intentionally added by UPD to the surface.^505^ There is also a very clear dependence on the crystal facets that dominate in the material. By selective poisoning the surface, it was found that especially the {110} facet is most active close to the onset potential,^107^ in agreement with findings for ethanol oxidation on planar Au single-crystal electrodes.^524^

Disentangling the effects of surface sites and elemental composition is not trivial because morphological and compositional parameters are correlated to each other and both may influence electrocatalysis. As has been pointed out in section 2, the content of residual Ag in NPG obtained by dealloying Ag–Au correlates systematically with the ligament size. Larger size implies a lower overall Ag content due to longer and thus more complete dealloying. In a comparison of the catalytic activities in NPG with different ligament sizes, it was found that the fine ligaments showed the lowest current density related to the internal surface area.^34^ However, the samples with the smallest ligaments also had the highest Ag content, and thus it is difficult to relate the effect to either of the two factors.^34^ Interestingly, the selectivity for formate production vs formaldehyde changed with ligament size and Ag content when electrolyzing methanol at a constant potential (selected as the peak potential of respective cyclic voltammograms).^34^ At this potential, the surface is slowly covered by an oxide layer, which may change the activity and selectivity. As shown in Figure 29A, the metallic character of the surface is preserved to higher potentials when more Ag is present at the surface and promoted the oxidation formate. In another study, formaldehyde was not detected as a product by ^1^H NMR after prolonged electrolysis in alkaline solution, probably due to fast further oxidation to formate and consumption by the homogeneous reaction 21 in alkaline solution.^505^

Ag was found to play a crucial role for the activity of porous nanoparticles obtained from dealloying an Au–Ag–Cu ternary alloy.^240^ Ag enhanced the dissolution rate of Cu and stabilized the porous structure and substantially enhanced the catalytic activity related to the mass of Au in the electrode material compared to porous nanoparticles prepared from Au–Cu alloys.^240^ This effect was found for methanol, ethanol, 2-propanol, and glycol.

The presence of Ag also changes the product distribution.^505^ By investigation the oxidation of methanol and formate in alkaline solution at NPG obtained by dealloying to *x*_Ag_^res^ < 1% and then reintroducing Ag by UPD, it could be shown that increasing Ag surface concentration leads to an optimum of *x*_Ag_^res^ for the 4e^–^ oxidation at 1.2 V (RHE). A further oxidation of methanol and formate to CO_3_^2–^ occurs only at an oxide-covered surface at 1.55 V (RHE) when Ag oxide is present at the surface. The current density of this process increases with *x*_Ag_^res^ (Figure 29B,C).^505^

High current densities at lower potentials (starting at −0.4 V Hg|HgO or 0.53 V (RHE)) were obtained with NPG obtained from dealloying Al–Au–Pt and Al–Au–Pt–Pd alloys in NaOH compared to materials obtained from a Al–Au alloy.^241^ The material was processed to a powder, from which a catalyst ink was formed with Nafion as binder. The current densities were reported as normalized to the catalyst mass and geometric area of the electrode rather than to *A*_ECSA_ (Table 1). The much higher activity of the Pt- and Pd-containing alloys is very likely an effect of the intrinsic catalytic activity of Pt and Pd contained in the alloy. However, their effect may not only originate from their intrinsic catalytic activity alone but also from their lower surface mobility. Slowing down the rates of metal atom surface diffusions allows obtaining material with smaller feature sizes and thus a higher mass-related surface area.^241^

**Table 1:** Performance Data of Selected NPG Electrodes for Methanol Oxidation Reaction

electrode, preparation route	electrolyte	*v* [mV s^–1^]	*E*_p_ [V vs RHE]	*j*_p_ [mA cm^–2^]	ref
potentiostatic dealloying Ag_77_Au_22_Pt_1_ in 0.77 mol L^–1^ HClO_4_ passing a charge of 5 C cm^–2^, subsequent annealing in air at 425 °C for 2 h	1 mol L^–1^ MeOH + 5 mol L^–1^ KOH	10	0.69	0.92	(525)
free corrosion of Au_16_Cu_84_ nanoparticles in 3 mol L^–1^ HNO_3_, final composition *x*_Cu_^res^ = 2% (EDX)	1 mol L^–1^ MeOH + 0.5 mol L^–1^ KOH	5	1.30	0.045	(240)
potentiostatic dealloying from Ag_75_Au_25_, final *x*_Ag_^res^ < 1% (EDX), UPD deposition of Ag to surface content of *x*_Ag_^res^ = 9% (XPS)	1 mol L^–1^ MeOH +0.1 mol L^–1^ NaOH	10	1.25	0.084	(505)
potentiostatic dealloying from Ag_75_Au_25_, final *x*_Ag_^res^ < 1% (EDX)	1 mol L^–1^ MeOH + 0.1 mol L^–1^ NaOH	10	1.20	0.042	(505)
porous gold nano bowls, *x*_Ag_^res^ = 4%, from reduction of HAuCl_4_ by hydroquinone in presence of poly(vinylpyrrolidone) and AgCl nanocubes, removal of AgCl by NH_4_OH	2 mol L^–1^ MeOH + 0.5 mol L^–1^ KOH	20	1.18	0.133	(527)
planar polycrystalline Au	1.5 mol L^–1^ MeOH + 0.01 mol L^–1^ KOH	10	1.25	0.076	(528)

The effectiveness of such catalysts with respect to the use of Pt as the most valuable component can be optimized by dealloying Au–Ag–Pt alloys with only 1–3% Pt.^525^ In this case, the alloy was prepared as ribbon, which was subsequently dealloyed partially by passing a fixed charge under potentiostatic conditions. This causes the dealloying front to advance 7–10 μm into the 200 μm thick ribbon. There are significant differences to the dealloying of binary Au–Ag alloys: as has been noted above, the addition of Pt yields smaller ligament size (see section 2.2). Due to the correlation between ligament size and residual Ag, the residual silver content in nanoporous Au–Pt can be quite high, e.g., 45–55%,^114^ depending on the exact alloy and the depth in the dealloyed layer. Dealloying protocols that bring down *x*_Ag_^res^ to 10% even in bulk samples of nanoporous Au–Pt have been reported.^119^

**Figure 30:**
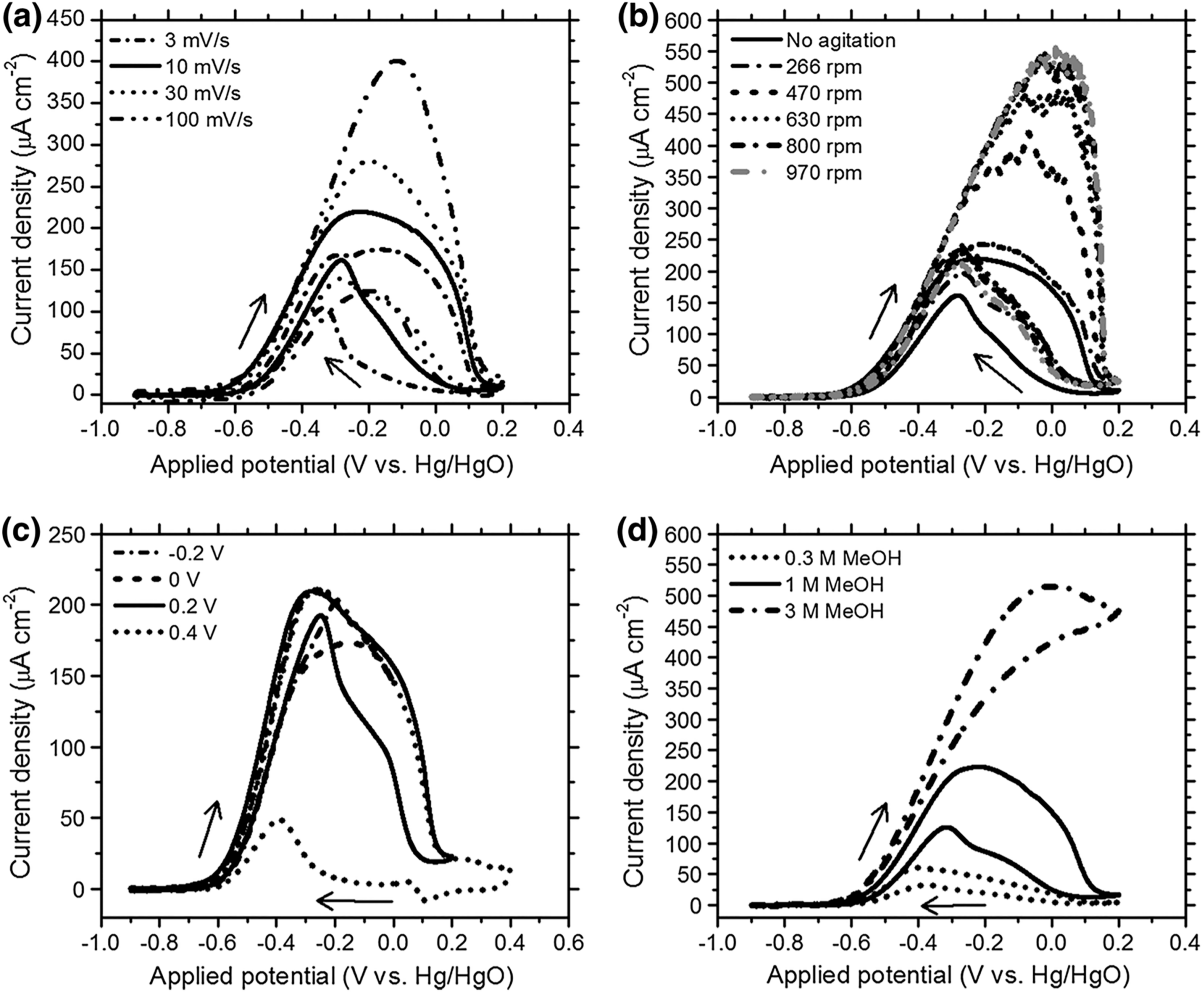
MOR studied at NPG obtained from Ag_77_Au_22_Pt_1_ at 298 K, current densities are calculated with respect to the true surface area. (a) Variation of scan rate; 1 mol L^–1^ MeOH + 5 mol L^–1^ KOH in quiescent solution. (b) Variation of solution agitation by an external stirrer, *v* = 10 mV s^–1^, 1 mol L^–1^ MeOH + 5 mol L^–1^ KOH. (c) Variation of the positive vertex potential, *v* = 10 mV s^–1^, 1 mol L^–1^ MeOH + 5 mol L^–1^ KOH, quiescent solution. (d) Influence of MeOH concentration, *v* = 10 mV s^–1^, MeOH + 5 mol L^–1^ KOH, quiescent solution. Reproduced with permission from ref (525). Copyright 2016 Springer.

Those electrodes achieved higher current densities of 210 μA cm^–2^ under comparable conditions than those found by dealloying Ag–Au alloys (Table 1).^525^ The peak current density is observed at considerable less positive potentials than for NPG obtained from Ag–Au alloys. The study also provided a careful evaluation of other limiting factors.^525^ Mass transport limitations are evident from the dependence on the scan rate (Figure 30a) and the possibility to increase the observed current density by enhancing the external mass transport by stirring (Figure 30b).^525^ In this study, potential excursions were generally restricted to avoid the formation of surface oxides because this may impact the MOR in the negative-going half-cycle. Finally, the role of the MeOH concentration was demonstrated (Figure 30d).^525^

With the addition of Pt, CO_3_^2–^ was found as one oxidation product besides formate, whereas in Pt-free samples, the oxidation proceeded to formate only.^525^ The amount of CO_3_^2–^ formed increased with the Pt content.^525^

**Figure 31:**
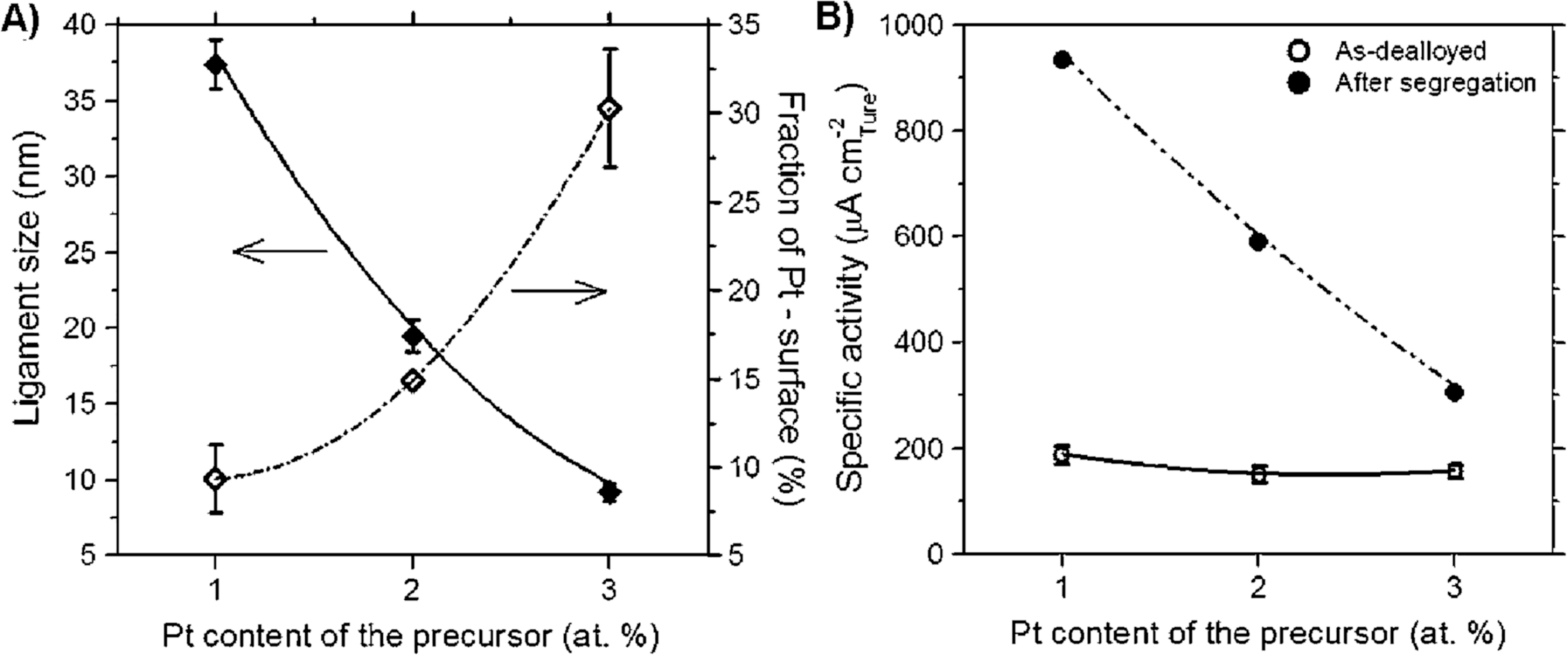
MOR studied at NPG obtained from Ag_77_Au_23–*x*_Pt_*x*_ (*x* = {1, 2, 3}) at 298 K with and without surface segregation of Pt during treatment at 425 °C for 2 h. (A) Variation of ligament size and surface Pt content. (B) Comparison of peak current densities before and after Pt surface segregation. Peak current densities are calculated with respect to the true surface area. *v* = 10 mV s^1^, 1 mol L^1^ MeOH + 5 mol L^1^ KOH, quiescent solution. Reproduced in part with permission from ref (525). Copyright 2016 Springer.

Interestingly, the alloy with the lowest Pt content resulted in the highest activity. However, this activity could be substantially increased to 920 μA cm^–2^ by a heat treatment of the NPG sample in O_2_-atmosphere. This causes the oxophilic Pt to segregate to the surface (Figure 31A). Although the current density increased dramatically with surface segregation of Pt, the sample with the overall smallest amount of Pt at the surface (Figure 31A, as determined by hydrogen UPD) gave the largest current density (Figure 31B, related to the true surface area). This can be seen as an indication that it is not the Pt content alone, but the synergy of Pt well dispersed in an Au matrix, that gives rise to the high MOR activity.^525^

The role of Pt in those NPG samples is also evident from the MOR electroxidation in 0.5 mol L^–1^ HClO_4_. In this electrolyte, NPG obtained by dealloying Au–Ag shows almost no activity before the onset of surface oxidation, whereas, NPG obtained from Au–Ag–Pt alloys provided a current density of about 0.1 mA cm^–2^ at 0.23 V (Hg|Hg_2_SO_4_|K_2_SO_4_) or 0.91 V (RHE). In contrast to alkaline solution, the sample with the highest surface concentration of Pt provided the highest current density. Surface segregation shifted the onset of the MOR peak in negative direction but slightly decreased the peak current density. However, the effect was much lower than observed in alkaline solution. This can be seen as a hint toward the passive role of Au in MOR in acidic solution, whereas it exhibits some electrocatalytic activity in alkaline media in the sense of stabilizing adsorbed intermediates,

Special NPG architectures have been synthesized.^526^ They allowed measurement of the binding strength of CO to Pt embedded in an NPG structure vs Pt metal. The results highlighted that Pt-modified NPG is not only advantageous because of the high degree of dispersion of the more valuable Pt component, but also because of the electronic interaction of Pt and Au that favorably modulates the binding strength of intermediates of MOR.^526^ Overall, NPG as a matrix or support for a Pt-based or Pd-based electrocatalyst seems to come closest to technical requirements compared to other NPG materials that are free of Pt-group metals (Table 1). A more extensive list with more details is provided in the SI, Table S5.

Next to Pt, Pd has received attention as the third, catalytically more active metal. For instance, Pd-containing electrocatalysts are broadly explored for direct ethanol fuel cells (Table 1 of ref (529)). Impressive performance was obtained by dealloying Au–Pd–Ni alloys, in which the resulting ratio between Au and Pd could be easily tuned by the ratio of the two noble elements in the precursor alloy.^530^ The catalytic activity for ethanol oxidation to acetate increased with the final Pd content and reached up to 6 mA cm^–2^ for 75% Pd in the nanoporous metal. The paper does not specify the method of area determination and most likely the current was normalized to the geometric surface area. In this system, the morphology is not very much affected by the Pd–Au ratio and thus facilitates studies of the intrinsic catalytic properties of the metals within an alloy without superimposed effects of morphology.^530^ Nanoporous Au–Pd materials have also been obtained by sputtering an Au–Pd–Mg film and dissolving Mg.^529^ Currents of up to 250 mA cm^–2^ (geometric area) were reported.^529^

Au–Pd alloys have also been produced on anodically roughened stainless steel by first depositing Cu and then performing a galvanic replacement reaction with Au, or Pd, or Au and Pd. The electrodes were used for the oxidation of glycerol.^531^ The best performance with current densities of up to 140 mA cm^–2^ were reported for the electrodes containing Au and Pd compared to the electrodes that contained only one of the two metals.^531^

#### 6.2.4 Oxidation of Aldehydes and Carbonic Acids

Formic acid is another C1 building block which can be of interest for oxidation in a direct formic acid fuel cells or for a complete oxidation of methanol to CO_2_/CO_3_^2–^. At Au, this reaction is slow and requires other elements to proceed with appreciable rate. Ag in NPG can enable this reaction at relatively high potentials in alkaline solution.^505^ However, technically interesting current densities are only approached with Pd and Pt as minor element.^532^ Different procedures have been used to arrive at nanoporous Au–Pt or Au–Pd materials. Nanoporous alloy nanoparticles were formed starting from Ag nanoparticles and galvanic replacement with Au and Pt followed by a dealloying step to remove more Ag.^255,532,533^ Two reaction pathways were observed dominating at different potentials in 0.5 mol L^–1^ H_2_SO_4_.
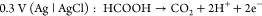
22
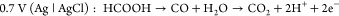
23

Similarly, active materials have been obtained by bubble-assisted galvanic codeposition of Au and Pt.^255^ Pt thin layers have also been formed by decorating NPG obtained from dealloying by Pt and a subsequent UPD of Bi.^533^ Formic acid oxidation in 0.5 mol L^–1^ H_2_SO_4_ at this material reached 100 A mg^–1^_Pt_ at +0.6 V (RHE).^533^

Au–Pd systems have been obtained by modifying NPG from dealloying by reducing Pd in the inner surface of NPG using hydrazine as chemical reductant.^534^ Current densities of 11 mA cm^–2^ at −0.3 V (with respect to geometric area) were reported.^534^

#### 6.2.5 CO_2_ Reduction Reaction

Electrochemical carbon dioxide reduction reaction (CO_2_RR) at planar Au electrodes yields CO but occurs concurrently with the hydrogen evolution reaction (HER). This is mainly a result of the low adsorption energy of CO on Au.^535^ The preference for CO_2_RR can in principle be increased by a design of the surface structure of the electrocatalyst or by manipulating the accessibility of the electrode for the reactants CO_2_ and H^+^. For relatively small nanoparticles and nanowires (2–8 nm diameter), DFT calculation predicted different CO_2_RR and HER activities that indeed correlated with experimental data for those nanostructures and were related to different ratios of low coordinated Au surface atoms.^536^ It was also shown that grain boundaries increase the specific activity for CO_2_RR on planar Au electrodes^537^ and in massive nanoparticles on carbon nanotubes.^538^ Residual copper from dealloying Au_20_Cu_80_ resulted in a nanoporous Au_3_Cu alloy in which electron density is transferred from Cu to Au atoms at the surface that promotes the release of CO.^535^

The effect of morphology control, i.e., the control over feature sizes on much larger length scale, on the CO_2_RR has been successfully demonstrated by electrodeposited NPG in the shape of inverse opal structure.^250^ The formation of a a pH gradient in the pore space as a typical feature of porous electrodes and the role of grain boundaries have also been confirmed for NPG obtained from deposited AuAg layers of 1 μm that were subjected to chemical dealloying.^539^ A partial current density of CO_2_RR of 6 mA cm^–2^ with an Faradaic efficiency of 99% was reported in CO_2_-saturated 50 mmol L^–1^ K_2_CO_3_.

Interestingly, the efficiency of NPG obtained by dealloying can be tuned by the dealloying procedure itself.^39^ Potentiostatic dealloying at high positive potentials leads to an oxide film that reduces surface mobility of Au and thus causes smaller ligaments as compared to NPG obtained from the same alloy by free corrosion. If NPG obtained by these two routes is used for CO_2_RR, the material derived from the NPG with a thick oxide layer yielded a much higher Faradaic efficiency at low overpotentials (90% at −0.3 V (RHE)). As both materials were obtained from the same alloy with low density of grain boundaries, this is unlikely the reason for higher activity. The smaller ligaments and pores may enhance the depletion of the reactants for the competing HER. The thick oxide layer may leave some subsurface oxide when reduced only partially. This in turn may cause dislocations at the surface that may be a further reason for the observed enhanced CO_2_RR reduction activities.^39^ The authors also noted an analogy to the role of intermediate thick oxide layers in the gas phase oxidation of alcohols.^17^ With the alkalization of the solution close to the interface, not only Cu but also low amounts of other reduction products like formate and methanol were detected by recording them with a microelectrode positioned above the NPG electrode and by NMR.^503^ The product distribution could be varied by the roughness of the NPG electrodes that influenced the pH within the porous material.^503^

For technical application, the mass transport of the reactants must be improved beyond the limits that are possible with bulk NPG. Hierarchically structured electrodes obtained by templated electrodeposition of an AuAg alloy into a photoresist template followed by removal of the template and dealloying yielded a material with high surface area due to the nanometer-sized pores in the dealloyed material and fast mass transport into the depth of the electrode via the micrometer-sized channels templated by the photoresist.^37^ It can also be potentially attractive to produce CO with a less than maximum Faradaic efficiency. The resulting CO-H_2_ mixture can be used directly as syngas, provided that the exact ratio CO:H_2_ can be adjusted. This has been demonstrated with NPG obtained by pulsed laser deposition.^540^ Ionic liquids and ionic liquid–water mixtures have also been used to enrich CO_2_ and suppress HER.^43^ Due to the high viscosity of ionic liquids, all mass transport processes are generally slowed down strongly. A combination of engineering pore sizes and variation of the ionic liquid–water ratio facilitated current densities of about 5 mA cm^–2^ at −0.5 V (RHE) with Faradaic efficiencies of approximately 90% for CO.^43^ The NPG material with strictly defined pore sizes was obtained by replicating twice an anodic aluminum oxide template, first by poly(methyl methacrylate) (PMMA) and dissolution of aluminum oxide, followed Au electrodeposition into the PMMA template. The final electrode was obtained after dissolution of PMMA.^43^

### 6.3 Surface Modification for Improvement of Electrode Properties

Section 6.2 has already highlighted the importance of dealloying ternary alloys to introduce a third element for enhancing catalytic activity or to stabilize the morphology. Pt can also be electrodeposited using the limited amount of precursor contained in the solution-filled pores after transfer of the NPG electrode to a precursor-free solution.^541^ It has been applied to fabricate Pt nanoparticles on NPG as support.^518^ Pd has been electrodeposited by electroless deposition.^534^ Probably, more defined surface architectures are obtained by UPD of Cu to prepare the surface for a subsequent galvanic replacement reaction (e.g., Cu UPD, exchange for Pt^507^ or Pd^531,542^). Another variation has been termed atomic layer electrodeposition. Pt is electrodeposited on NPG, and the concomitant UPD process of hydrogen on Pt was said to prevent the deposition of more than one Pt layer.^543^ Removing the capping hydrogen layer by application of potentials positive of the hydrogen UPD zone opens the surface for deposition of another Pt layer during the subsequent deposition pulse. The resulting Pt layers were used for pH sensing.^543^

Some modification with other material classes shall be reviewed below that open doors for hybrid materials as catalysts. The combination of NPG with thin layers of transition metal dichalcogenides as hydrogen evolution catalysts is attractive to combine the advantages of both materials. Dichalcogenides such as MoS_2_ can be electrodeposited on NPG by potential cycling from solution containing precursors (such as MoS_4_^2–^ sometimes formed upon mixing the deposition solution).^544^ This procedure allows the variation of the thickness of the material on the surface of the ligaments.^544^ In a recent study, the plasmonic excitation of NPG was used to initiate and guide the growth of a thin film of MoS_*x*_ on the ligament surface.^545^ The resulting materials showed a less negative onset potential for HER and a smaller Tafel slope than MoS_2_ alone. The advantages of the hybrid material were attributed to a large number of sulfur sites and the better conductivity within the catalyst due to the 3D NPG structure.^545^ Electrodeposition on NPG has also been used to coat the ligaments of NPG by a composite of WS_2_ embedded in poly(3,4-ethylenedioxythiophene) (PEDOT) by electropolymerization of 3,4-ethylenedioxythiophene in an aged solution of (NH_4_)_2_WS_4_, polyethylene glycol, and LiClO_4_ in water.^546^ The material was tested for HER and showed a low onset potential and a low Tafel slope. Here, NPG is used as a 3D support material of high conductivity. The details of the preparation of NPG are not crucial, and NPG from different preparation routes has been used. Even the replacement by a less costly 3D metal is conceivable for this application.^546^ However, the relative ease of the wet chemical procedures and the high control over a bulk 3D morphology for NPG is certainly the main advantage in these developments.

NPG is also an interesting support material for coordination network compounds such as metal–organic frameworks and metal hexacyanometallates. Many compounds of these material classes show limited ionic or electronic conductivity that severely hamper the exploitation of their interesting functional properties for electrocatalysis, electrochromism, or photoelectrochemistry.^547,548^ The potential advantages of a combination of the metal−organic framework compound ZIF-8 (zeolitic imidazolate framework of ordinal number 8) has recently been highlighted.^549^ Nanoporous gold nanoparticles (ca. 340 nm diameter with ligaments of ca. 37 nm diameter) were encapsulated by a 200 nm layer of ZIF-8. An electrode prepared from this hybrid material showed good activity and Faradaic efficiency of 44% for nitrogen reduction to ammonia in neutral aqueous solution at −0.6 V (RHE).^549^ The superior performance compared to other materials was attributed to the intrinsic catalytic activity of NPG (with about 3% of Ag). Usually, this electrocatalytic ability of NPG cannot be harvested because of the competing HER that leads to very low current efficiencies for NH_3_ formation. In the hybrid material HER was suppressed by the ZIF-8 encapsulation.^549^

Iron hexacyanoferrate (Prussian Blue), a known catalyst for selective H_2_O_2_ reduction in glucose biosensors, has been electrodeposited on NPG obtained by anodizing and reduction of pure gold. The large surface area increased the linear working range of the resulting sensor.^550^ In this application, NPG has the role of a highly conductive and inert support material. The high conductivity of the NPG support was also used to construct an OER catalyst working at high positive potential in alkaline solution. It consists of CoMoN_*x*_ nanosheets electrodeposited on NPG wires.^551^

### 6.4 Electrochemical Sensors based on Nanoporous Gold

Electrocatalytic sensors are a main field of application for NPG and NPG-derived electrode materials. While electrocatalytic activity is certainly required, catalytic activities or current densities do not need to be extremely high because the substance under study (the analyte) is usually available in μmol L^–1^ to mmol L^–1^ concentration only, whereas 1 mol L^–1^ is often used in MOR for energy conversion. More important for chemical sensors is the selectivity, that is, the ability to distinguish a signal coming from the analyte vs signals caused by other compounds contained in the sample (the matrix). An important aspect for sensors is the possibility to integrate the NPG electrode in sensors of miniaturized cells. Here, as pointed out in section 2.6, NPG is distinguished by the fact that its preparation by thin film deposition and chemical or electrochemical corrosion affords precise lateral structuring in lithographic processes.^218,219^ This promises a seamless integration into established microdevice production lines. 3D printing approaches provide further opportunities for structuring NPG.^152^ This suggests opportunities for particularly purposeful structuring in technological applications. Yet, porous gold obtained by other methods (section 2.9) than dealloying has so far found more attention for these applications. Another important aspect is biocompatibility, especially if the sensor surface shall be used in direct contact with biological tissue. This situation occurs not only for sensors but also for electrodes in biomedical devices. Electrocatalytic sensors and biomedical devices made from NPG have recently been reviewed in detail.^27,65,66,552^

#### 6.4.1 Inorganic Ions and Compounds

NPG film electrodes have been applied for the coulometric reduction of U(VI) as a possible replacement of the traditionally used Hg electrodes.^229^ The procedure takes advantage of the high surface area and the relatively large overpotential for the competing HER.

Hydrogen peroxide H_2_O_2_ is an interesting analyte, as it is released as part of inflammatory response in biological organisms. It is one of the reactive oxygen species that are of concern in biology and in energy conversion devices, and thus, there is a need for analyzing its concentration. It is also a redox-active product of the enzymatic glucose oxidation according to eq 24.
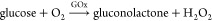
24Detection of H_2_O_2_ is exploited in some glucose sensors, although most current commercial devices exploit mediated glucose oxidation.^553^

H_2_O_2_ can be detected in neutral solution such as phosphate buffer saline (PBS) by *reduction* at −0.4 V (vs SCE).^554^ This potential is less negative than the potential required for H_2_O_2_ reduction at planar Au electrodes. The use of a reduction (instead of oxidation) avoids interferences with the electrooxidation of alcohols, glucose, ascorbic acid,^554^ dopamine, and uric acid^226^ that are common interfering compound in amperometric biosensors. The samples have to be deaerated to avoid interference with the reduction of dissolved O_2_. The performance has been achieved with dealloyed material^554^ but also with porous Au obtained by electrochemical roughening that does not contain other elements that modulate catalytic activity.^226^

Besides detection of H_2_O_2_ at the bare nanoporous electrode, surface modifications are used in order to enhance the selectivity or to increase the sensitivity. For instance, iron hexacyanometallate was deposited on NPG as an catalyst that enabled H_2_O_2_ reduction at much less negative potentials (−0.05 V (Ag|AgCl)) than at bare NPG.^550^ The inner surface of NPG electrodes has also been used for modification with a layer of hemoglobin, a redox protein able of direct electron transfer with roughened gold surface and the ability to catalyze H_2_O_2_ reduction at −0.3 V (Ag|AgCl).^264^ Of course, the surface of NPG can also be modified with a monolayer of Pt that is more active for H_2_O_2_ reduction than Au and provided detection limits below 1 μmol L^–1^.^555^ Such an electrode was used to detect H_2_O_2_ released by stimulated PC12 cells in a cell culture medium.^555^

H_2_S is a toxic gas; however, at trace levels in the parts-per-billion (ppb) concentration range, it is a biomarker formed in the walls of arteries and is informative about peripheral artery disease and small vessel disease. The detection of trace H_2_S levels emitted through the skin has been achieved with a gas-diffusion electrode at which NPG is in direct contact with skin and linked by a Nafion polymer electrolyte to the reference and counter electrode. The efficient capture of almost all molecules transdermally released enabled the very low limits of detection required for this application.^556^

Electrochemical detection on nitrite NO_2_^–^ was accomplished by anodic oxidation at nanoporous gold that was obtained by bubble-templated electrodeposition. The morphology could be tuned by the deposition time and the potential applied for the reduction of the gold precursor and the concomitant formation of H_2_ bubbles.^19^

An interesting application is the use of NPG in all-liquid fuel cells with hydrazine N_2_H_4_^557^ or NaBH_4_^558^ as fuels and H_2_O_2_ as oxidant. In case of NaBH_4_ as fuel, Pt-coated NPG showed a higher open circuit voltage in agreement with the higher catalytic activity for the BH_4_^–^ oxidation at Pt.^558^ However, the cell operation under load was much better with unmodified NPG. The authors related this to the tendency of Pt to catalyze the disproportionation of H_2_O_2_ that does not inject electrons into the external circuit but may lead to gas bubble formation that hinders mass and charge transport in the electrolyte. The lower activity of NPG compared to Pt-coated NPG for the disproportionation was thus an advantage for this application.^558^

#### 6.4.2 Nonenzymatic Glucose Sensing

Besides detection of blood glucose as part of managing diabetes, for which enzymatic glucose sensors are the dominating approach,^553^ there is a need for the detection of glucose and other carbohydrates in other applications, for example, in electrochemical detectors after a separation by anion-exchange high performance liquid chromatography^559^ or in urine.^252^ Nonenzymatic detection of glucose in neutral solution is possible at planar gold electrodes when using pulsed amperometric detection to avoid blocking of the electrode by intermediates or matrix components.^560^ Similar to the oxidation of alcohols, the high number of undercoordinated surface atoms and the high surface area can improve the performance. Apart from sensing, there is also a current interest in the conversion of carbohydrate and other natural compounds as sustainable feedstock for the synthesis of versatile building blocks for a wide array of chemicals and materials.^561−563^

In this context, glucose oxidation has frequently been studied at NPG as a model reaction.^216,231,253,265^ A key challenge for the use of NPG electrodes as sensors is the presence of Cl^–^ in most sample matrices.^564−567^ Cl^–^ specifically adsorbs to Au surfaces and may inhibit electrocatalytic reactions and thus change the sensitivity for glucose and interfering compounds such as ascorbic acid. The porous Au electrodes can also be prepared by electrochemical treatment other than dealloying (section 2.9) and work without the presence of other elements.^252,253,566^ The effect of residual Ag in NPG obtained by pulsed dealloying has been studied in acidic,^216^ neutral, and alkaline solutions.^568^ Coating with a cation exchange membrane such as Nafion, that is impermeable for anions but permeable for neutral compounds and cations, was found to be an effective mean for enabling glucose detection in this matrices.^567,569^ Nanoporous copper obtained from dealloying Cu_30_Mn_70_ was coated with a thin gold film and provided similar performance as NPG with a much lower Au content.^265^

NPG electrodes have been further modified in order to enhance the performance. For instance, films of cobalt oxides were deposited under hydrothermal conditions in the presence of surfactants to coat the ligament surface of NPG with a film of cobalt oxide.^570^ In this case, the catalytic reaction proceeds at the metal oxides and NPG provides a conductive support with high surface area to the metal oxides, which have a low intrinsic conductivity and thus cannot be used as porous electrode material by themselves. In turn, the metal oxide coating inhibits the coarsening of the NPG ligaments. Coating with Pt showed a rather minor effect^541^ compared to the effect Pt coating has on MOR (section 6.2.3). An optimum coverage was identified.^541^ Pt-containing material was also obtained by dealloying Pt_4_Au_16_Cu_80_.^569^ The obtained material with a Nafion coating was active for glucose oxidation and H_2_O_2_*oxidation* in PBS.^569^

Occasionally, the use of NPG for glucose biofuel cells has been envisioned.^260^ Despite high current densities that have been obtained, the high potential of 1.2 V (vs RHE) leaves little room for the development of a usable voltage output when combined with an ORR as the cathodic reaction, which yields usable current densities at around 0.9 V (vs RHE).

#### 6.4.3 Detection of Other Organic Analytes

The detection of dopamine, a neurotransmitter, and ascorbic acid have been studied on porous Au electrodes obtained by dynamic hydrogen bubble templating, i.e., a material that does not contain a LNE.^35^ The two analytes responded differently to structural features in neutral solution. Dopamine adsorbs to Au and benefits from the larger surface area which allows preconcentration, whereas the irreversible ascorbic acid oxidation requires surface defects for enhanced oxidation currents.^35^ The detection of ascorbic acid has been investigated at NPG and could be enhanced by coating the ligaments with small amounts of Pd.^542^ Reactions of biomolecules typically involve heterogeneous electron transfer steps coupled to homogeneous proton transfer. The nanopore environment enhances the probability that reactants encounter each other during the homogeneous reaction steps, independent of the chemical nature of the electrode material.^506^ This confinement mechanism may contribute to the enhanced oxidation current and the observed lower overpotentials in dopamine oxidation and ascorbic acid oxidation.^35^ Exploiting the reduction of the oxidized dopamine by an a excess of ascorbic acid could also be used to boost the sensitivity for dopamine without too large signals from ascorbic acid itself.^571^ Adaption of the electrochemical detection for the detemination of ascorbic acid in acidic environments^572^ and in the presence of a strongly absorbing matrix with uric acid as an interferent^573^ were demonstrated as well.

The confinement effect was explicitly studied for 1,4-benzoquinone electrochemistry at nanoporous Pt and Au electrodes, in which the nanoporous materials showed an enhanced reversibility of the electrode reactions.^574^ A much faster reduction kinetics was also reported for the pharmaceutical drug metronidazole, 1-(2-hydroxyethyl)-2-methyl-5-nitroimidazole, on NPG electrodes produced by pulsing the electrode potential applied to a planar Au electrode.^575^*p*-Nitrophenol, a compound that is of concern when released to the environment, has been detected at porous Au electrodes obtained by an anodization process in oxalic acid.^227^

Epinephrine, a redox-active neurotransmitter, was determined at porous Au with good detection limits.^576^ The material was obtained by electrodeposition of Au inside the pores of anodic aluminum oxide templates.^576^ Similar results were also obtained by dealloyed NPG by the same authors,^577^ indicating that, in this case, it is indeed the confinement effect in the pores that accelerates the overall reaction rather than the surface chemistry of the ligaments.

#### 6.4.4 Nanoporous Gold as Support for Biomolecules

Au surfaces in general lend themselves to surface functionalization by thiol chemistry under mild conditions by which a large variety of structural motifs for further coupling chemistry can be anchored to the metal surface.^578^ The transfer of those concepts has been demonstrated early for template-deposited mesoporous Au, in which a monolayer of the redox mediator (4-carboxy-2,5,7-trinitro-9-fluorenylidene)malononitrile (TNF) was linked to a layer of the cofactor nicotinamide adenine dinucleotide (NAD^+^) and the enzyme glucose dehydrogenase to realize an artificial electron transfer chain.^251^ The enzyme horse radish peroxidase (HRP) was immobilized in NPG and used to realize a direct electron transfer, which was claimed to be improved by a higher packing of HRP in the porous material.^579^ Direct electron transfer was also reported for the enzyme fructose dehydrogenase (FDH).^580^ In a detailed study, the effect of different thiol linker molecules was studied, and a comparison between planar Au(111) electrodes and NPG was conducted, which showed similar trends with respect to catalytic efficiency of FDH and the linker chemistry. Enzyme electrodes based on NPG showed higher currents and prolonged stability, which are attractive features for application in biosensors and biofuel cells.^580^

Immobilization of glucose oxidase (GOx), which catalyzes reaction 24, and H_2_O_2_ detection at the NPG electrode were used for measuring of the glucose concentrations.^581^ Apart from demonstrating the surface functionalization scheme, this model sensor cannot compete with commercial glucose biosensing devices especially with respect to sustainability and price.^581^

Other biosensing approaches use a support electrode to immobilize a biorecognition element, for instance, an antibody or a single-strand oligonucleotide, to capture the analyte, which in those cases is the antigen or a specific nucleotide sequence, respectively. Along this line, NPG was used for a DNA assay with detection by electrogenerated chemiluminescence (ECL).^582^ The analyte oligonucleotide was first captured by an oligonucleotide strand immobilized on NPG. The CdTe quantum dot as ECL emitter was also modified by single-strand oligonucleotides and could recognize the other end of the analyte DNA sequence. The use of NPG enhanced the available surface area for the determination.^582^ The advantage of a larger surface area to hold more probe molecules is offset for thicker layers or narrow pores by transport hindrance of the analyte oligonucleotides in the pore space. Consequently, an optimum for the thickness and pore morphology was found for assays with a redox-active label that undergoes electron transfer with NPG.^583^ Aptamers are artificially selected antibodies consisting of self-hybridized single DNA strands that are able to recognize non-DNA analytes. Such aptamers against bisphenol A have been immobilized on the ligament surface of NPG.^584^ Bisphenol A is a monomer used in the production of polymers that are used in food packaging. Its release in trace quantities raises health concerns due to its hormone-like properties, and thus, it must be monitored. The immobilized aptamers enriched bisphenol from the sample during an exposure time of 30 min. Afterward, bisphenol A was electrooxidized at NPG, which showed a slightly lower overpotential for this electrooxidation than planar gold or glassy carbon.^584^ The sensor benefited from the high surface area to accommodate more aptamers and the electrocatalytic activity of the ligament surfaces in NPG.

#### 6.4.5 Electrodes in Biomedical Devices

The noble character of gold, the high surface area, and thus high capacity and the absence of particulate matter that may have harmful biological effects are advantageous properties for the use of NPG as contact electrodes in biomedical advices. Macro-/mesoporous gold has been used as electrodes for extracellular recording in which the biological cells couple capacitively to the recording electrode.^67^ Another example is the use of two flexible NPG thin film electrodes on the inside of a contact lens for an integrated enzymatic biofuel cell.^585^ One electrode, acting as anode, was modified by lactate oxidase to utilize lactate from the tear liquid as fuel. The cathode was modified with bilirubine oxidase that is able to reduce O_2_ smoothly to H_2_O. In this application the inertness and high surface area of NPG was beneficial.

### 6.5 Nanoporous Gold in Electrochemical Energy Conversion Devices

The use of NPG in electrochemical energy conversion devices has received a lot of attention due to the urgency to develop drastically improved devices for the decarbonization of the economy and their widespread deployment. Recently, the area has been reviewed.^25,69,486,487^ Electrochemical energy conversion plays a central role in almost all sustainable energy supply scenarios. However, the range of conductive materials that withstand high positive or high negative potentials in acidic solution is small. Nanoporous gold for energy applications and the required widespread use of such devices will meet supply issues, especially for the most active metals such as Ir, Ru, and Pt. Although Au is a noble metal, the supply is much larger than that of the aforementioned metals, and it has been investigated as an alternative because it meets the chemical stability requirements.

#### 6.5.1 Nanoporous Gold in Fuel Cells

The oxygen electrode is the more critical electrode in a fuel cell and water electrolyzer. Unfortunately, the obtained catalytic activity for NPG falls short of technical requirements in those devices unless the surface is modified with other precious metals (section 6.2). As cathode for the ORR, Au has the disadvantage that it favors the 2e^–^ reduction to H_2_O_2_.^516^ Although the second 2e^–^ reduction to H_2_O is promoted in NPG (compared to planar Au) due to the confinement effect, it does not compete with Pt that promotes almost exclusively the 4e^–^ reduction. However, Pt nanoparticles supported on carbon tend to oxidize the carbon support at high positive potentials during start–stop cycles. Here, NPG as support offers improved stability^507^ compared to carbon supports although being associated with substantially higher costs. The use for fuel cell anodes is most advanced for the oxidation of small organic molecules at NPG decorated with a low amount of Pt brought to the material as a component of the original alloy^114,224,525^ or by postdealloying modification.^18,507,533^

#### 6.5.2 Nanoporous Gold in Batteries

NPG has found application in research batteries that have not yet entered the commercial market. The use of new negative electrode materials with very high Li storage capability (e.g., lithiated Li_3.75_Si 3500 mA h g^–1^, Li_4.4_Ge 1600 mA h g^–1^, Li_4.4_Sn 990 mA h g^–1^ vs LiC_6_ 373 mA h g^–1^) is hampered by their poor stability upon cycling, which is routed in their large volume expansion upon lithiation (over 200%) that causes enormous stress and ultimately pulverzation of the materials when applied in macroscopic shape.^586−588^ However, successful applications of those materials have been demonstrated by building three-dimensional nanostructured electrodes, allowing for accommodation of the volume change.^586^ The accommodation of the volume changes of Sn upon lithiation/delithation were demonstrated with a NPG electrode as a three-dimensional current collector onto which Sn clusters were deposited as active material by currentless deposition.^588^ Further advantages were demonstrated for Ge as active electrode material coated onto NPG by a gas phase process as a continuous conformal film.^587^ This electrode showed an excellent rate capability (47.5% capacity at 60 C compared to 1 C) and capacity retention of 90% after 100 cycles. These achievements were due to the use of an amorphous Ge layer, the exploitation of the interconnecting networks of a phase with high electronic conductivity (Au), and another phase with high ionic conductivity in the electrolyte-filled pores, short ion-transport distances within the thin layer of the active material on the surface of the ligaments, and the accommodation of the volume change of the lithiated Sn in the pore space.^587^ In a similar direction, NPG has been used as a support and current collector for transition metal oxides (e.g., TiO_2_) that can intercalate Li^+^ at negative potentials but have low electronic conductivities. Conventionally, they are used with a conductive carbon agent processed in complicated formulation steps that must balance a high content of the active material, good conductivity, and mechanical stability of the composite electrode. In the case of NPG, films of TiO_2_ have been coated around the ligaments of NPG so that access of electrons to all parts of the active material is easily ensured without binder and composite formation.^589^ Electrodeposition of MnO_2_ with low electronic conductivity on NPG enabled the use of this material as intercalation electrode without a binder.^590^

Most notable is the use as positive electrodes in rechargeable Li–O_2_^235,591,592^ and Na–O_2_ cells^593^ in organic electrolyte solution. In this application, O_2_ is reduced to Li_2_O_2_, thus the promotion of the 4e^–^ reduction of O_2_ is not desired. Instead, high positive potentials during charging must be tolerated. NPG offers excellent ways of designing separately a high surface area for the ORR and at the same time transport pathways for O_2_ by using a bimodal pore size distribution^207^ that improved the performance over NPG with a monomodal pore size distribution.^235,591,592^

#### 6.5.3 Nanoporous Gold in Supercapacitors

Supercapacitors store energy in the electric double layer and/or in a thin redox-active film around the actual electrode material. The key feature of this storage device is the fast uptake and release of the charge (high power) and the very high stability over several 10 000 cycles. Usually, the capacity is inferior to batteries. The energy stored in the electric double-layer scales with the surface area and the potential that can be applied to it without electrolyzing the electrode material or the electrolyte. Thus, it is very important to avoid electrocatalytically active electrode materials that would be able to decompose the solvent or other electrolyte components. In this respect, nonaqueous electrolytes can be of high interest.^594^ However, often their ionic transport properties are inferior compared to concentrated aqueous electrolyte solutions or gels. The energy stored in a thin redox-active film depends on the density of the redox centers and the total amount of the redox-active material. Making redox-active layers thicker is often limited by the low electronic conductivity of the redox-active material itself. A low conductivity would prevent a fast charging and discharging under full utilization of the active material and thus give away one of the central features of a supercapacitor. Here the high electronic conductivity of NPG in combination with a tunable pore size for ionic transport offers the possibility to realize supercapacitors using very thin films of redox-active material such as MnO_2_^595^ and to exceed the performance characteristics that can be achieved with the more conventional porous carbons as conductive support.^596^ This is especially true when the metrics are made with respect to volume instead of mass.

The high surface area of NPG together with the inertness of this noble metal is an attractive feature for supercapacitors. Accordingly, the modification of the ligament surface with redox-active layers has been explored in order to enhance the performance beyond a pure double-layer capacitance (similar to their common use in combination with porous carbons).^597^ Successful modification with redox-active transition metal oxides/hydroxides include MnO_2_,^594,595^ RuO_2_,^598^ CuO,^599^ and Ni(OH)_2_.^600^ Intrinsically conducting polymer films, such as poly(pyrrole)^109,601^ and poly(aniline),^602,603^ have also been tested. The combination of different redox-active materials has been demonstrated with MnO_2_ and poly(pyrrole).^604^ This even applies to combination of NPG with further electrodeposited nanomaterials such as reduced graphene oxide that was assembled with poly(pyrrole) to a planar device with outstanding characteristics.^605^ As exemplified in ref (605), electrochemical procedures are often used in various ways for the preparation of the redox-active films offering high control over the amount of deposited material. This is needed to adjust the capacity and the transport characteristics under operating conditions. A recent review provides a detailed comparison of the different approaches.^69^

Overall, the use of NPG in energy conversion devices has seldom advanced to experiments on the device level despite the promising results that have been collected in half-cell experiments and miniaturized laboratory cells. Beside the high cost of Au, this is also caused by procedural problems to integrate NPG in larger devices of layered architecture. Therefore, the prospective for real-world application is seen in niche applications such as electroorganic synthesis of high-value chemicals, high-cost medical devices, and sensors that can exploit the high surface area and use planar integration.^599,604,605^ Those areas may be more receptible to the material cost of NPG than commodity application such as conventional batteries, fuel cells, or blood glucose sensing.

## 7 Catalysis by Nanoporous Gold in Organic Synthesis

Many studies have been conducted in the gas phase^38,71,606^ because of faster mass transport, lower variety of products, and avoidance of homogeneous background reaction.^607,469^ However, working in the liquid, i.e., condensed phase is a crucial requirement for a widespread use of NPG in organic chemical synthesis.^608^ Considering the maturity of the field in the gas phase, there are relatively few reports on reactions in solution. One can only speculate as to the reasons for this, but there seems to be very little predictability of reactivity and selectivity and relatively little mechanistic understanding. On the work that has been done, several reviews have appeared that describe the synthetic value of NPG in the liquid phase for substrates beyond MeOH that was treated in this paper in section 5. In 2013^609^ and 2014,^38,610^ the first reviews that dealt with NPG in organic synthesis listed already the most important transformations, to which few fundamental additions have been reported subsequently.

Perhaps this illustrates the difficulties of this area. The review paper by Jin et al.^611^ from 2019 provides a summary of synthetically useful oxidation and reduction reactions with the perspective on general catalysis by nanoporous metals, which builds on the foundations of an earlier review on the same topic.^612^ A further review deals with transformations that form carbon–heteroatom linkages that are either catalyzed by Au nanoparticles or NPG, in which the different reaction outcomes illustrate the influence of the support on the reaction.^613^ The latest review in the field focuses on new insights into structure–property relationships in the context of synthetically useful reactions catalyzed by NPG.^40^

The following section will attempt to describe the reasons for current limitations of NPG in organic synthesis and highlight more recent work that has not been covered in previous reviews. We will exclude reports in which the NPG was used as a high-surface area support for the immobilization of active materials such as photocatalysts^614,615^ or enzymes.^68^

The reactions with NPG are all either oxidation reactions or reduction reactions, although some may be classed as comproportionation reactions. Oftentimes, the reaction does not halt at the direct oxidation product, but further products such as condensation products may be observed as exemplified in detail in section 5 for MeOH oxidation in liquid phase.

### 7.1 Oxidation Reactions of Organic Molecules by Nanoporous Gold

**Scheme 2:**
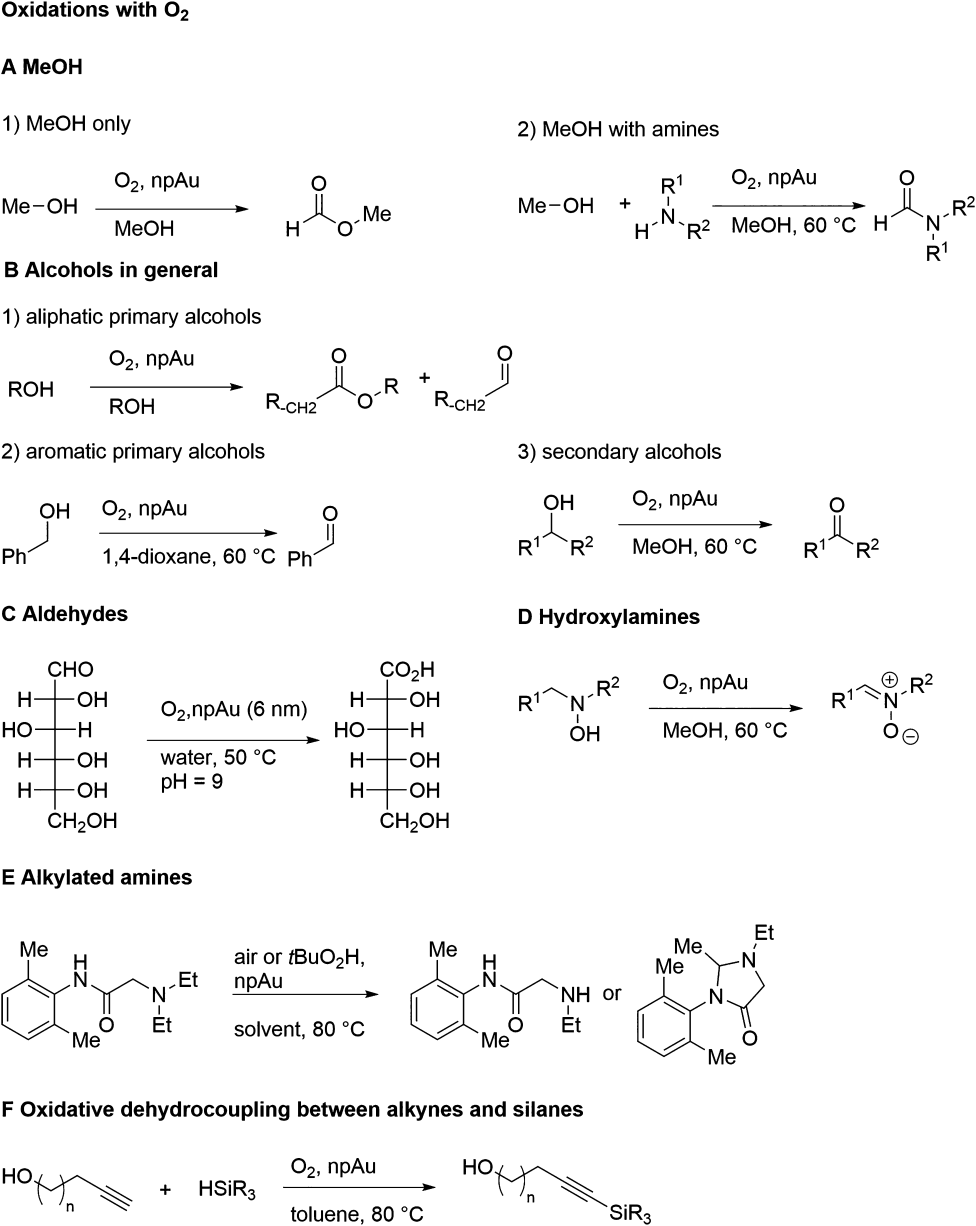
Overview of the Type of Oxidation Reactions that Have Been Performed to Date

Oxidation reactions with oxygen are environmentally benign, cheap, and do not lead to residual reagent. As of the time of this review, there are only a few classes of starting materials that have been used for this transformation (Scheme 2).

The best researched liquid phase oxidation with NPG is the oxidation of methanol to give methyl formate (Scheme 2A). Other primary alcohols react in a similar manner, giving either the ester or the corresponding aldehyde in mixtures.^59^ This reaction is therefore not competitive in preparative organic chemistry, as many, much more selective, and cheaper options exist, many of them based on supported Au nanoparticles.^616^ Secondary alcohols react without such complications to the corresponding ketones with oxygen as the oxidant (Scheme 2B).^479^ The solvent in these reactions was MeOH, which appeared not to influence the yield of the desired alcohol. However, when the primary alcohol benzylalcohol was used under these conditions, methylbenzoate was formed with MeOH. Only when MeOH was excluded and 1,4-dioxane was used, benzaldehyde was the exclusive product (Scheme 2B).^479^

Considering the complex postulated mechanism for the oxidation of primary alcohols (section 5), it was perhaps a logical extension to present additional nucleophiles other than MeOH. Indeed, when an oxidation of MeOH with oxygen is performed with primary or secondary amines, which are much superior nucleophiles, the corresponding amides result in excellent yields (Scheme 2A).^617^

Aldehydes can also be oxidized to the carbonic acids: One of the earlier examples in which oxidations have been examined for larger, synthetically relevant molecules was the oxidation of d-glucose to d-gluconic acid in water with oxygen as an oxidant (Scheme 2C).^60^ The focus of this work was on the mechanism rather than on the scope: The reaction mechanism was postulated to proceed in three steps: the adsorption of glucose on NPG, the surface oxidation reaction, and the desorption of the product. A detailed molecular reaction mechanism was not provided, but because the reaction proceeded best in slightly alkaline reaction conditions, the desorption step was thought to be rate-determining. Smaller ligament sizes (as low a 6 nm diameter) and slightly elevated temperatures of 50 °C provided shorter reaction times.

The oxidation of nitrogen-containing organic molecules is often difficult to perform in a selective fashion because a variety of products and intermediates may be involved starting from the amine to the highest oxidation state: hydroxylamines, nitrones, oximes, nitroso compounds, and nitro compounds as well as N–N coupled products such as hydrazines, diazocompounds, and azoxycompounds. Therefore, the selective oxidation of hydroxylamines to the nitrones can be regarded as a breakthrough in this regard (Scheme 2D).^618^

A more unusual oxidation reaction that is one of the very few applied reactions is the oxidative dealkylation or cyclization of lidocaine, which was performed to synthesize a drug metabolite (Scheme 2E).^619^ This reaction can yield two products, depending entirely on the reaction conditions, but in a selective fashion. It needs pointing out that this is one of the very few reactions, in which a peralcohol, *tert*-butylperoxide, can also be used as the oxidant, which may be more easily administered than O_2_ in a synthetic organic chemistry laboratory.

NPG has also been used in oxidative dehydrocoupling reactions between terminal alkynes and silanes (Scheme 2F).^620^ This is of synthetic relevance because terminal alkynes are C–H acidic, which may interfere with other reactions, and thus, silylgroups serve as protecting groups. The reaction may also become of synthetic relevance as it tolerates hydroxy groups, which in other silylation protocols (with silyl chlorides and bases) typically react first and thus require additional protection steps, although other catalytic alternatives exist.^621^

The oxidation of olefins is also of potential interest, but selectivity is often a problem. Oxidation products may be epoxides, diols, diketones, and further oxidation products that may also involve C–C bond breaking. Thus, it is perhaps unsurprising that this avenue has not yielded much success in oxidative processes with NPG. However, there is one report on the oxidation of cyclohexene, in which different surface treatments were carried out prior to the reaction.^622^ Selectivity is low and products are 2-cyclohexen-1-one 2-cyclohexen-1-ol cyclohexene oxide cyclohexenyl hydroperoxide.

### 7.2 Comproprotionation Reactions

**Scheme 3:**
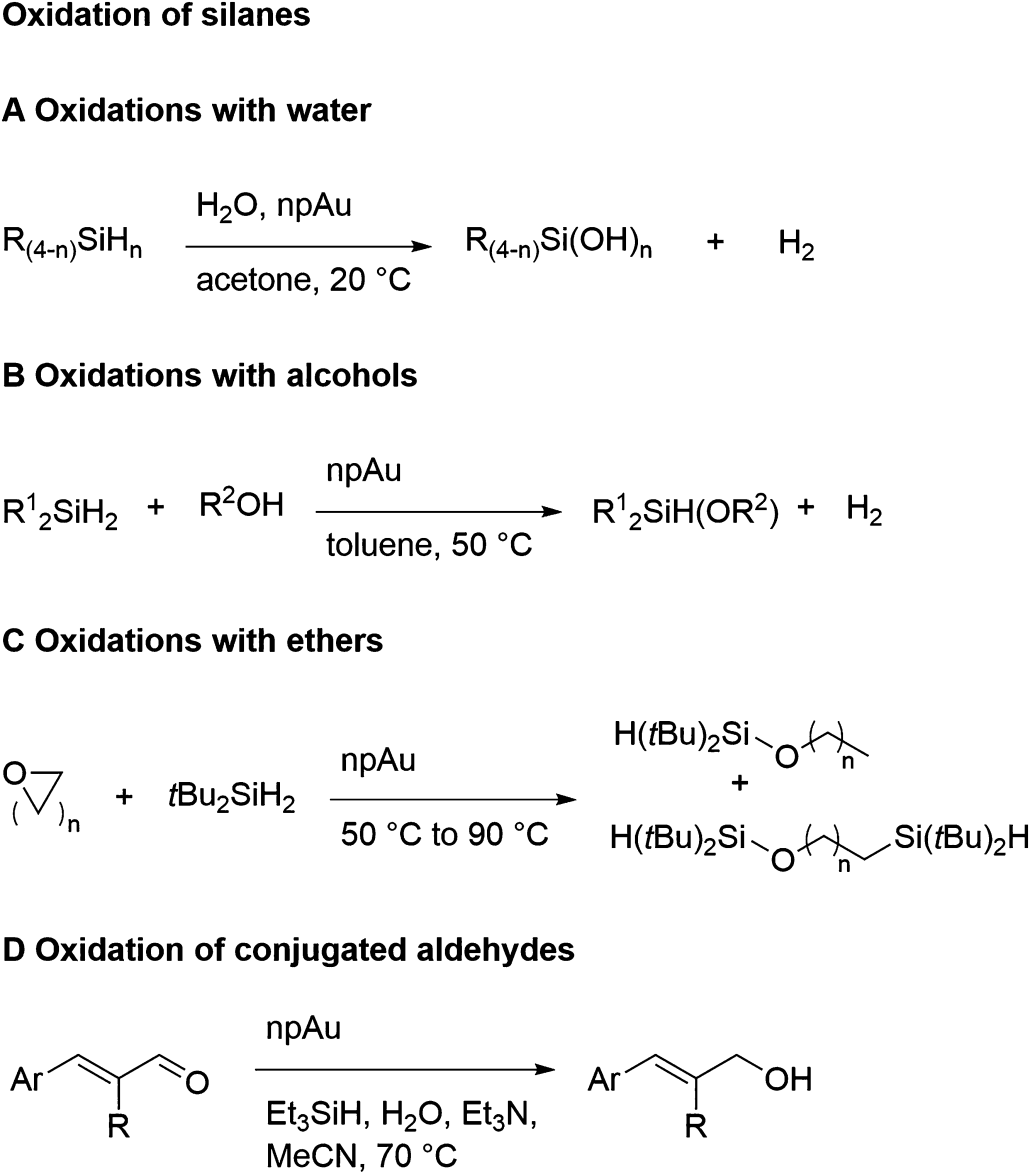
Oxidations with Water, Alcohols, or Ethers

In silanes, the hydrogen atom attached to Si has a slightly hydridic character. It can therefore react relatively easily with a proton, thus forming dihydrogen. Although this reaction may be viewed as an acid–base reaction, if one regards the formal oxidation states of the hydrogen atoms before and after the reaction, it can also be seen as a comproportionation. Because protons are typically easy to provide, silanes often oxidize easily to the corresponding silanols. With NPG as a catalyst, this reaction can be facilitated with water as the formal oxidant, while dihydrogen is being released (Scheme 3A).^623^ In a seemingly similar vein, silanes are able to react with alcohols, forming the corresponding silanol ethers (Scheme 3B).^624^ Even reactions with cyclic ethers are possible, in which the ring is opened; however, with a reduced selectivity (Scheme 3C).^624^ If one compares this reaction with the oxidative dehydrocoupling described above (Scheme 2F), one might reasonably wonder why in that case,^620^ oxygen was required whereas here, it is not. The literature does not offer any mechanistic explanation but merely observations: First, the reaction between the alkynes and silanes does not proceed if there is no oxygen present. Instead, a hydrosilylation reaction takes place, which is reductive (cf., section 7.3). Second, if the catalyst was prepared from a dealloyed AuAl alloy, this reaction was also the dominant one.^624^ As residual Ag is well-documented to be necessary for oxygen activation, the mechanism for the silylation of alkynes does not appear to follow a simple acid–base reaction mechanism.^620^ Indeed, the reactions of silanes with alcohols are only formally acid–base reactions.^624^ Electron spin resonance spectroscopy (EPR) revealed that the silane reacts with the NPG by forming radicals. It was suggested that the abstracted hydrogen atoms recombine on the Au surface to form H_2_, which was indeed observed.^624^ Ring opening of cyclic ethers can relatively easily be envisioned following a radical pathway, but the silyl ether formation with the alcohols remains mechanistically unclear. All reactions described in this section are very sensitive to solvents, temperature, and even residual less-noble atoms in NPG^624^ that mechanistic generalizations should not be made at this stage. Last, silanes can also be employed to perform selective 1,2-reductions of conjugated aldehydes (Scheme 3D).^625^

### 7.3 Reduction Reactions by Nanoporous Gold

**Scheme 4:**
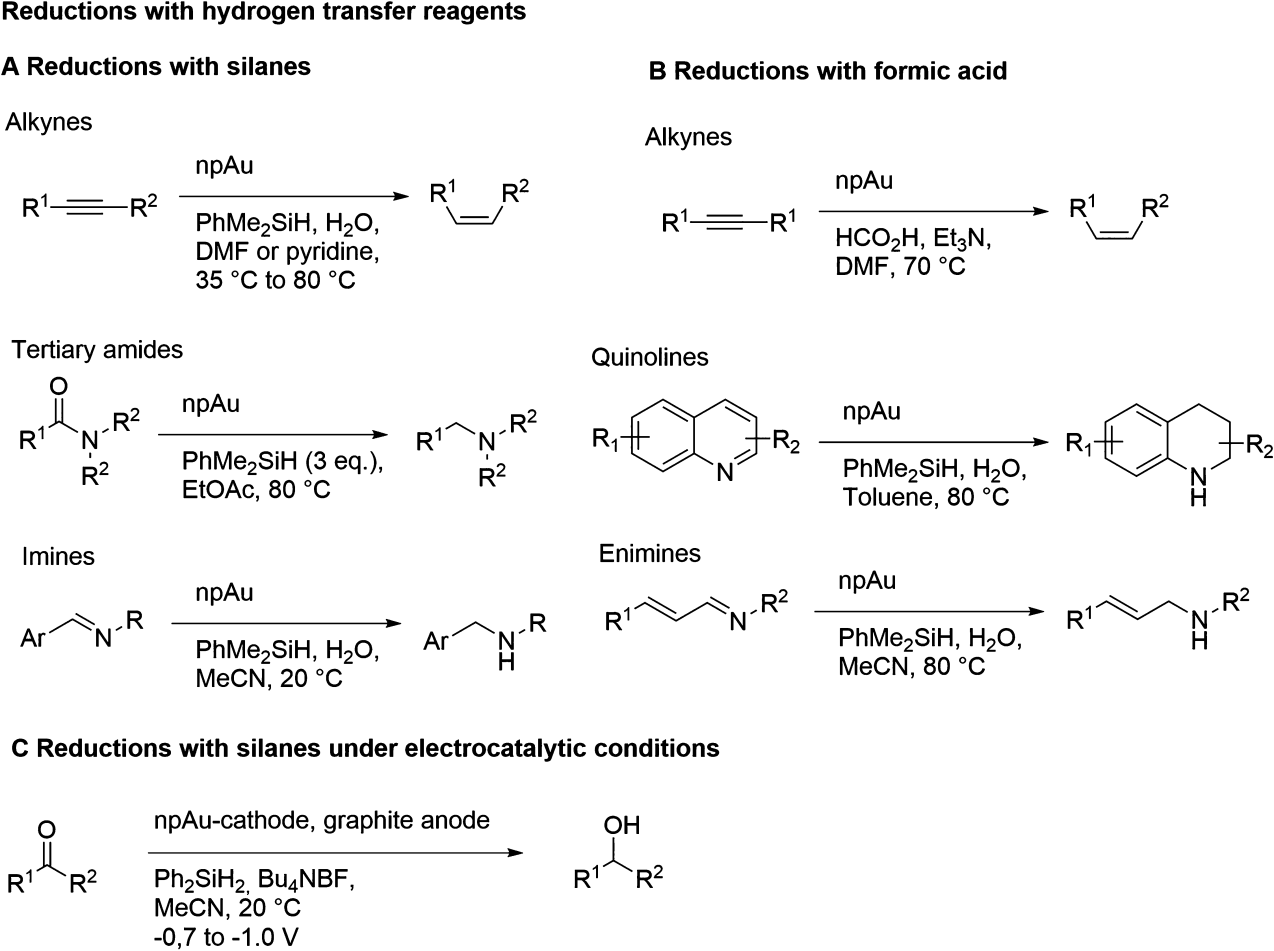
Reduction Reactions with Nanoporous Gold

Reduction reactions (Scheme 4) would be ideal if they could be performed with hydrogen, H_2_. However, H_2_ does not dissociate as easily on Au surfaces as it does on other metals, such as Pt or Pd, which renders it ineffective.^468^ Therefore, transfer hydrogenation reagents are often employed, which are typically hydride donors in the combination with water.

One type of such hydride transfer reagent are silanes, which are relatively easily oxidized. For example, alkynes can be reduced exclusively to the *Z*-alkenes with PhMe_2_SiH and water in the presence of amines (Scheme 4A).^626^ As avoiding overoxidation and *Z*-selectivity are not easy to achieve, this may become a useful reaction. A Z-selective reduction of alkynes has also been performed with formic acid and an amine, which may be considered “greener” chemistry than the use of silanes (Scheme 4B).^627^

PhMe_2_SiH and water were also used as hydrogen transfer reagents for the reduction of quinolines to 1,2,3,4-tetrahydroquinolines with NPG, which did not proceed at all with hydrogen.^628^ The proposed reaction mechanism is an indirect hydrogen transfer, in which a hydride is abstracted from the silane, which is simultaneously attacked by water. Deuteration experiments with D_2_O showed a preferential incorporation in the 3-position. Based on the data presented, it is possible to propose a subsequent hydration/protonation reaction mechanism, in which hydrogen bonding may play a role in arranging the reagents on the gold surface. Even amides, which are relatively difficult to reduce and are often treated with the aggressive LiAlH_4_ can be reduced with silanes, using a NPG catalyst.^629^ The reduction of imines and enimines under similar conditions is perhaps to be expected; however, the high selectivity that allowed aldehydes to remain intact without protection is notable.^630^

Silanes can also be activated electrocatalytically and ketone and aldehyde reduction can take place with high selectivity, leaving the normally extremely sensitive nitro group untouched (Scheme 4C).^631^ The high yields and selectivity of this reaction projects a future application in organic synthesis.

**Scheme 5:**
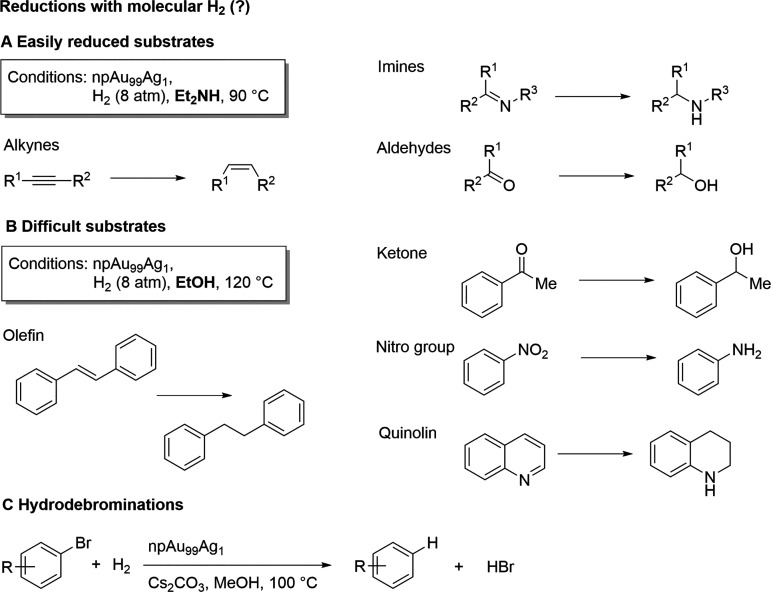
Reductions under a Hydrogen Atmosphere

As previously mentioned, H_2_ used to be considered a poor reductant. However, Yamamoto and Bao and co-workers^632^ reported in 2016 that H_2_ could be used at a relatively low pressure of 8 atm for the reduction of aldehydes, imines, and alkynes (Scheme 5A), with triethylamine as a solvent, provided that the residual Ag content in NPG was particularly low (ca. 1 mol %). Less reactive substrates like ketones, alkenes, quinolines, and aromatic nitro groups required ethanol as a solvent (Scheme 5B). A bit later, the same groups reported the hydrodebromination of aromatic bromides by NPG with H_2_ (Scheme 5C).^633^ This reaction is useful in that there are occasions in which the Br substituent is used to block a certain otherwise reactive site. If it can be removed easily, it becomes a valuable protection group. As impressive as these results are, the activation of hydrogen remains relatively unexplained. XPS data did not convincingly demonstrate hydrogen activation, and the authors speculated that in fact, the altered structure of nanoporous Au_99_Al_1_ compared to nanoporous Au_90_Al_10_ caused the difference.^632^ However, all of these reactions are most likely more complex than merely involving a H_2_ dissociation. The hydrodebromination, for instance, was performed in MeOH, and a substantial, quite similar turnover was detected even in the absence of H_2_.^633^ It is also notable that there was no reaction when no base was added,^633^ making it very likely that MeOH is being oxidized to formaldehyde, thus MeOH is the reductant. Moreover, the reaction is not very tolerant of other functional groups: aldehydes, carbamates, and nitro groups get reduced and esters hydrolyze.^633^ In the other cases, it is not clear whether the solvent diethylamine was entirely innocent or, indeed, why triethylamine was used in the optimization reactions, whereas diethylamine was used in reactions in which the scope was explored.^633^ Like MeOH, amines can be oxidized, although this reaction has been shown only for Au nanoparticles^634^ but not for NPG.

**Scheme 6:**
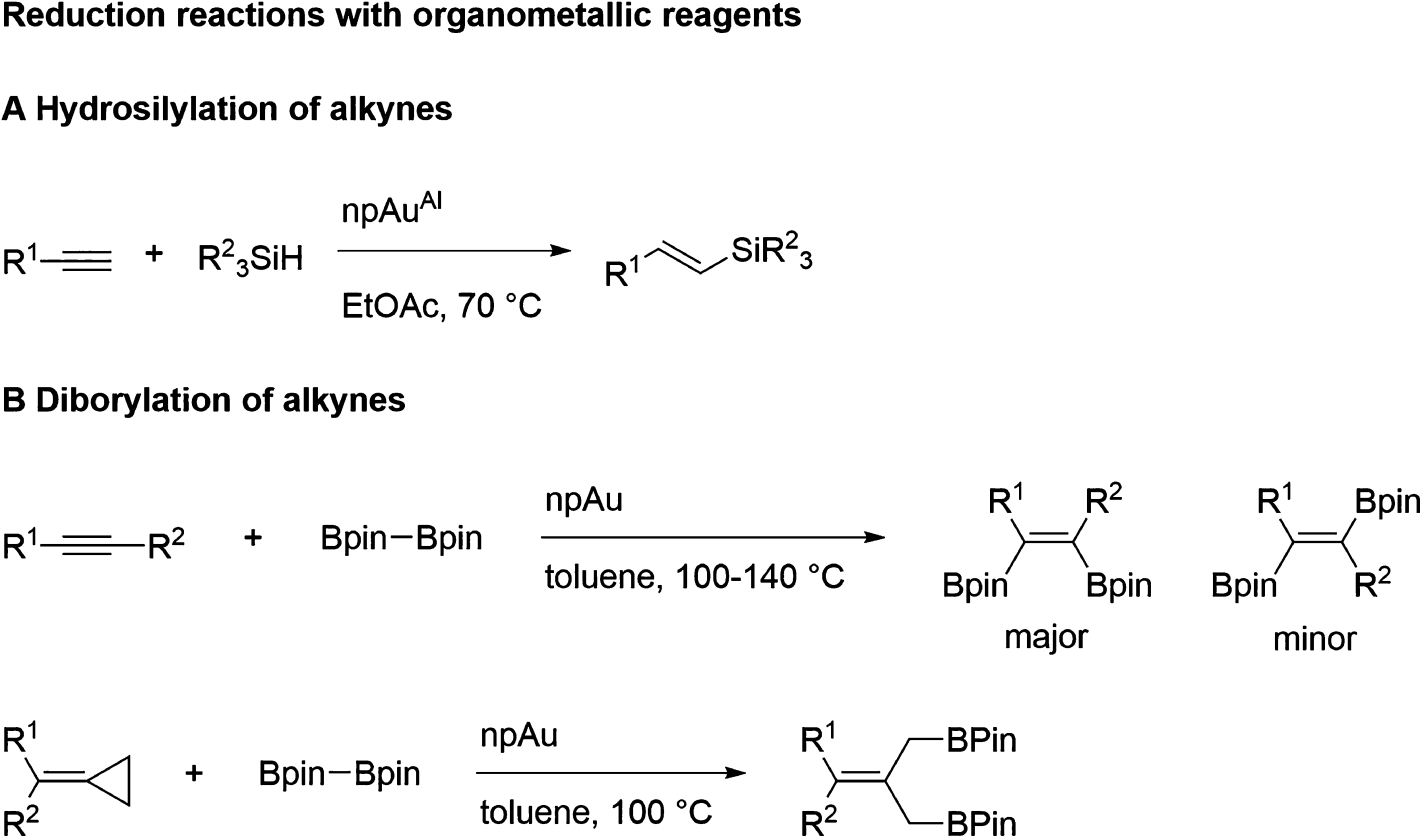
Reduction Reactions Yielding Organometallic Reagents

There are other formal reduction reactions, in which not (only) hydrogen is transferred, but in which an organometallic reagent is generated that is commonly used as reactive intermediates in syntheses (Scheme 6). Notable reactions are, for example, hydrosilylation reactions of alkynes, which proceed much more smoothly with NPG prepared from an AuAl than from AuAg alloys.^635^ The diborylation of alkynes (Scheme 6A) serves as another example.^636^ Although this reaction can be performed slightly more efficiently with nanoporous Pt (dealloyed from PtCu), the use of NPG avoids leaching of ions into the reaction mixture, which can be difficult to remove. Instead of alkynes, strained rings such as methylene cyclopropanes can also serve as substrates (Scheme 6B).^637^

The use of NPG as a catalyst in oxidation or reduction reactions has not yet become commonplace in organic chemistry. Thus far, literature reports are mainly concerned with testing the scope of a reaction and starting to elucidate mechanisms, but no study has been performed in which a particular synthetic goal was reached with such a reaction as the key synthetic step. This is likely due to both the scarcity of transformations possible at this present time and the availability of easily reproducible catalyst formation protocols that can be performed in an organic laboratory. Moreover, the scalability of the procedures is yet to be demonstrated.

## 8 Conclusion

The bicontinuous pore structure featuring a comparatively narrow size distribution of ligaments and pores in the range of a few 10 nm is a defining and outstanding feature of nanoporous gold (NPG). Its formation by a corrosive chemical or electrochemical dealloying process can be mechanistically understood in terms of a kinetically controlled self-organization process, in which the nanoscale pore structure results from the competition of two concomitantly occurring surface processes. While the less noble element (LNE) of a binary Au alloy used as a starting material is continuously dissolved under oxidizing wet-chemical conditions, surface diffusion of the remaining Au atoms provides for the generation of Au layers, eventually passivating the surface. Further curvature-driven surface diffusion minimizes the net interfacial excess energy and leads to coarsening of the structure accompanied by the formation of voids and finally pores. These processes and thus the morphology of the nanoporous Au material obtained in the end can be tuned by a multitude of adjustable parameters, such as the composition of the master alloy, the temperature, the electrochemical settings, potential thermal treatments subsequently applied, and the storage conditions before use (section 2). The possibility to tailor important structural parameters, such as the mean ligament and pore sizes and the porosity or solid fraction, respectively, distinguishes NPG from other mesoporous materials and qualifies it as a model system for studies aiming at exploring systematically structure–property relationships in areas for which Au nanostructures hold promising application perspectives as being the case in catalysis and electrocatalysis, for instance.

To fully exploit this potential, it is necessary to strive for ensuring the highest possible level of reproducibility when fabricating NPG. When furthermore aiming at tuning its structural features, stringent and reliable preparation protocols are mandatory and much effort was invested in the past to develop suitable procedures, ranging from the manufacturing of the starting alloy to various elaborated dealloying techniques. Section 2.4 describes such a protocol that has been proven to reproducibly yield NPG materials in different laboratories, exhibiting comparable structural and functional properties.

When trying to correlate catalytic properties with structural features to reveal, e.g., microkinetic rate constants or turnover frequencies, it does, however, not suffice to control ligament or pore sizes or the porosity alone. Equally important are quantities, such as the specific surface area and the tortuosity of the pore system, determining the catalytic capacity (per mass), on the one hand, and the diffusive mass transport properties of the porous material, on the other hand. How to derive these characteristics in case of NPG, is discussed in sections 3.3, 3.4, and 3.10. As became obvious there, such numbers can differ up to a factor of 2 when applying different conceptional approaches, even for one and the same sample.

One must then ask which of the conceptional approaches is relevant for catalysis. As far as the mass transport properties and their influence on the catalytic turnover are concerned, emerging techniques to directly measure the diffusive mass transport in porous matter, such as PFG NMR, can provide a remedy here (section 4.3). The diffusion-related tortuosity of the pore system in NPG is found to be well correlated with structural tortuosity values extracted from TEM tomography. This demonstrates how the comprehensive knowledge about NPG’s pore system meanwhile allows disentangling the observable macrokinetics from the microkinetics of the surface reaction.

NPG always contains an unavoidable residual content of the LNE that, in the case of catalytic applications, turned out to be a crucial ingredient. For instance, molecular oxygen can only be activated in the presence of such a LNE but not on pure gold surfaces. Even though the overall amount of the remaining LNE can be adjusted by dealloying procedures optimized in this respect, its distribution within the material is less easily controllable and can depend on various parameters. As exemplified for NPG obtained from AuAg master alloys in particular, it can change during storage and use of the material. Specifically, it was observed that Ag segregates to the surface in oxygen-containing atmospheres (section 4.1) or during electrochemical cycling in alkaline solutions (section 6.1). Moreover, it can be removed from the surface by electrochemical surface oxidation under acidic conditions. Surprisingly and unexpectedly in the first instance, it was shown by advanced TEM methods (section 3.13) that NPG may even contain nanometer-sized volumes within the ligaments that exhibit the composition of the master alloy and are surrounded by regions with Ag concentrations corresponding to the nominal overall bulk residual content. As revealed by high resolution micrographs, these Ag clusters had been protected by a continuous gold film during the dealloying step, preventing the oxidative dissolution of the LNE. Due to the surface mobility of gold atoms, however, it cannot be excluded that such regions might later be exposed to species adsorbed on the surface under catalytic turnover conditions. Given the importance of the LNE, in particular for the activation of molecular oxygen, such structural irregularities may thus have a considerable impact on the catalytic properties, such as activities and selectivities. Coarsening outside the corrosive environment has been demonstrated as a strategy for homogenization. It is desirable to routinely characterize the degree of compositional heterogeneity in catalysis studies and to explore in more detail how this aspect of the NPG microstructure affects its performance in various scenarios of catalysis.

In spite of such uncertainties, it is undisputable though that the research on NPG not only established a reliable knowledge base to control catalytically important structural and chemical characteristics, but also allowed obtaining a deeper mechanistic insight into its complex surface chemistry. The partial oxidation of alcohols is a good example in this respect. The generic reaction mechanism initially proposed for methanol oxidation to methyl formate emanated from the Brønsted basicity of oxygen atoms adsorbed on the Au surface as the main underlying driving force (section 4.1). As known from single-crystal studies carried out under UHV conditions, such species are indeed able to abstract hydrogen atoms from methanol and various other reaction intermediates playing a role in the catalytic cycle. While this mechanistic picture can readily explain the observed selectivity, including the formation of oxidative cross coupling between different alcohols as well as the formation of amides if reacting alcohols with amines, growing evidence revealed in the last years that additional aspects are important to reconcile all experimental observations with mechanistic explanations and theoretical predictions. One of the key issues in this respect concerns the question which of the various pathways, theoretically conceivable for the activation of molecular oxygen, is actually effective and how these pathways differ for total and partial oxidation reactions (sections 4.1 and 4.3). The generic mechanistic picture mentioned above leaves, for instance, open how oxygen species created at Ag sites are transferred and become available at Au sites where then the Au-specific oxidation chemistry takes place (section 4.1). Moreover, theory has identified reaction pathways other than a direct dissociative adsorption of molecular oxygen which may exhibit evenly low barriers (section 4.1). The situation is further complicated by the fact that activated oxygen species tend to aggregate on Au surfaces into islands. This does not only influence the activity of the species, but does also modulate the relative importance of their Brønsted and Lewis basicity and thus the propensity for partial or total oxidation reactions. Such differences are also reflected by quite different activation protocols necessary to prepare catalytically active samples for CO and methanol oxidation (section 4.3).

A further degree of complexity arises in the presence of water, which, for example, is a byproduct of partial oxidation reactions and was experimentally found to modify the surface reactivity significantly (section 4.2). While CO oxidation benefits from co-dosed water, the selectivity toward methyl formate suffers from its presence. From a mechanistic point of view, it was shown that adsorbed water is able to enhance the mobility of oxygen species bound to oxygen islands. In particular, theory proved that water may react with activated oxygen and other coadsorbed species to form reactive intermediates, which further on open new reaction channels for the partial oxidation so that a different product distribution is observed.

The catalytic versatility of NPG is documented by the fact that it not only can be utilized for gas phase reactions but also for applications in the liquid phase. As far as methanol oxidation is concerned, methyl formate is preferentially formed also here with a similar activation energy and comparable turnover numbers (section 5). Despite these analogies and the possibility to mechanistically explain effects specific to the liquid phase (such as the beneficial influence of an added base), the level of understanding regarding NPG’s catalytic properties is less advanced here and needs further research. Even though NPG has meanwhile been applied in a number of synthetically interesting organic reactions going beyond what has been tried in the gas phase, the experimental results obtained so far do not provide a consistent picture and oftentimes suffer from low yields and/or low selectivities (section 7). It should be kept in mind, though, that NPG was rarely characterized to an extent in the corresponding studies, as described in section 3. This leaves a margin of uncertainty with respect to the chemical properties of the material used in such work, rendering a more detailed discussion of possible reasons impossible at present.

In contrast, the electrochemical and electrocatalytic properties of NPG have been investigated and characterized in much more detail. The application of the material in this area is particularly attractive in view of the large surface area available for adsorbates and reactants or functional layers (section 6). The electrocatalytic properties depend on the surface composition, as in case of gas and liquid phase catalysis, as well as on the electrolyte used. This was exemplified for the methanol oxidation reaction in alkaline solution, revealing that Ag as the LNE plays a different role for the various reaction steps involved (section 6.1.2). While the 4-electron oxidation of methanol to formate is somewhat enhanced by small amounts of Ag at the surface, the 6-electron oxidation to CO_2_/CO_3_^2–^ occurring at higher positive potentials is significantly promoted by the presence of Ag. In contrast to that, the latter reaction channel is suppressed in the case of LNE-free NPG within the stability range of water. The effect of Ag can be associated with the presence of AgO, being the stable species at the potential where the 6-electron oxidation occurs. In acidic solution, on the contrary, the effect of Ag is small because of its dissolution from the surface in these electrolytes. This process is accompanied by a restructuring of the surface, which provides a means to tune the distribution of various surface sites (section 6.1) and thus the reactivity of the surface. However, the current densities observed, and potentials used for methanol oxidation reaction on NPG in alkaline solution are incompatible with a technical application of this material. Sufficiently high current densities at low potentials require the presence of Pt or Pd on the surface of NPG surface (section 6.2.3).

With respect to CO_2_ reduction, the activity of NPG benefits from an increased alkalinity in the pore space during electrolysis. This effect, which suppresses the competing hydrogen evolution reaction, originates from the porosity and mass transport conditions in the system (section 6.2.5).

## 9 Outlook

Despite the significant progress that has been made in recent years resulting in an increased understanding and command of the structural and compositional features of NPG as well as in atomistic insights into the mechanism of a variety of oxidation reactions, harnessing those achievements in terms of active and stable catalysts is still challenging. In the following, a number of areas are discussed, where ongoing research is expected to overcome existing hurdles for future applications.

### 9.1 Need for Establishing “Good Practice” Procedures for Catalytic Testing

The previous section revealed that NPG represents a catalytic material with well-defined structural and chemical characteristics, which can either be synthetically adjusted (such as pore and ligament sizes or the porosity) or are predetermined by the corrosive dealloying process (such as the tortuosity of the pore system for instance). In contrast to the various applications of the material being studied in materials science for a long time already and depending on the bulk properties, for catalytic applications the surface and its chemistry is essential. Previous research revealed that reaching control over the surface structure and composition is much more complicated. To accomplish this task and to establish standardized testing protocols which eventually allow identifying robust structure–property relationships for various reactions and fields of application, further research effort is necessary from two directions:

(a) In the interaction of theory and experiment further *mechanistic* insight must be gained, e.g., with respect to the surface sites and species which are crucial for an understanding of the reaction network determining the catalytic process. In case of oxidation reactions, it is clear so far that undercoordinated Au sites (i.e., step and kink sites) are important and that the LNE is likely to play an important role for the activation of molecular oxygen. However, it is unclear in which chemical form—as isolated metal sites, corresponding islands, or rather in the form of oxidic species—the LNE participates in the catalysis and to which degree nondissociative (with respect to molecular oxygen) reaction pathways contribute to the overall mechanistic scenario. Here, experiments with suitable model systems, e.g., studied under UHV conditions, as well as further theory can help. Moreover, investigations under catalytic working conditions carried out with modern operando techniques are necessary, taking into account that structure and composition at the surface, as is known, dynamically change as a function of the surrounding gas atmosphere.

(b) From a *practical* point of view, a better and more advanced understanding of the correlation between bulk properties which can be well-controlled already, and the surface properties must be established. To this end, more systematic studies are needed, elucidating to which extent the LNE segregates to the surface as a function of the bulk content and the composition of the gas atmosphere. Further research is necessary to clarify whether and in which way the presence of LNE-rich, buried clusters in NPG can be avoided or purposefully taken advantage of. It may be speculated that the clusters may otherwise cause unreproducible differences between nominally similar samples. Apart from that, the development of reliable activation protocols needs more attention, in particular for liquid phase applications. So far, corresponding recipes have been reported in the literature only for methanol oxidation and CO oxidation in the gas phase.

Given progress in these directions, NPG can emerge as catalytic material serving, for instance, as a reference catalyst in Au catalysis and as an example of good practice in testing heterogeneous catalysts with well-defined properties. As far as the preparation and a broadening of the material’s property space is concerned, further research regarding the following topics will support this goal:

### 9.2 Widening the Compositional Space of NPG

The structural and compositional properties of NPG ultimately determine the range of catalytic applications. Already in the past, a lot of effort has been undertaken to widen the compositional space of NPG, using, for instance, different LNEs or functionalizing the inner surfaces of the material with oxide deposits or organic molecules. This not only enlarged the scope of applications, but also allowed to gain further mechanistic insights, e.g., in the activation of oxygen or the use of water as an oxygen delivering agent (e.g., for the water gas shift reaction). Further research in this direction thus appears as an important field for future developments.

### 9.3 Need for Understanding the Impact of Dealloying on the Defect Structure

It is important to note that the dealloying process results in materials with a complex microstructure, details of which may critically depend on small variations in the preparation and processing protocols. While a detailed quantitative understanding of central aspects determining the dealloying process has been reached for AuAg master alloys, a similarly systematic understanding remains to be established for binary master alloys containing other LNEs and for ternary master alloys. In this regard, it would be important to better understand three separate issues: First, what is the correlation between, on the one hand, the crystal structure of the master alloy, and, on the other hand, the final structure of the material on the meso- and microscopic scale? Second, how is the lattice parameter misfit accommodated at the corrosion front? Third, how is the defect structure, grain size and the density of crystallographic defects such as dislocations, twin boundaries, and stacking faults, affected by the choice of master alloy and by the corrosion conditions?

### 9.4 LNE-Free NPG as a Reference Material

While we emphasize the essential role of the LNE for catalytic and electrocatalytic applications, it would also be desirable to have nanoporous gold materials with a comparable and similarly well-defined morphology available that are entirely free of LNEs. Such systems would be highly welcome as reference materials, to which the catalytic behavior of NPG containing LNEs can be compared.

### 9.5 Need for Scalable Production Protocols

From a practical point of view, approaches toward a scalable production of NPG are desirable. Although electrochemically controlled dealloying procedures currently offer better control and thus may yield materials of choice for sensors and microreactor technology, a scalable production of NPG is easier to achieve by using free corrosion protocols. Free corrosion processes can be varied by the choice of the corrosion medium and temperature in order to improve the controllability of structural (ligament and pore sizes) as well as chemical characteristics (residual LNE content). It is desirable to explore this approach with the aim of eventually achieving similar tuning options as given in the case of electrochemical dealloying.

### 9.6 Need for Appreciating Postdealloying Processing and Conditioning

Between the dealloying process and the catalytical application, NPG may undergo changes. These are either intended, such as coarsening to optimize mass transport conditions, or they occur spontaneously under catalytic working conditions. A fundamentally improved understanding of changes to the morphology as well as to the chemical and geometric structure upon interaction of NPG with adsorbates and neighboring phases (such as metal oxides) would open another turn key for controlling the reactivity and the stability of NPG-based catalysts.

### 9.7 NPG with Engineered Macroscale Morphologies

Other shapes of NPG than monoliths, foils, and thin films should find more attention in the future. Such engineered bodies from NPG may be flexibly fitted into integrated reactors. Examples are NPG powders processed to gas-diffusion electrodes combining macroscopically large area, flexible shapes, and direct connection to, e.g., membranes with optimized transport conditions for gaseous reactants and products. 3D printed and then dealloyed scaffolds have been demonstrated and might be explored further. Issues of recycling materials in different states of the life cycle (LNE in dealloying, usage of the very same NPG lot in multiple organic synthesis batches) may become more important as the application of NPG becomes more widespread.

Finally, it should be mentioned that there are a few applications, in which NPG serves as a mere support or back contact of other materials (metal oxides for catalysis and energy storage, biomolecules) or as a plasmonic absorber with excellent chemical stability and an optimum pore structure for efficient transport. For most of these applications, (electro)catalytic reactions are not required and are even detrimental to the stability of the technical device. Those fields may greatly benefit from the material as it is, and, consequently, these areas may be closest to technical applications at present.

## Supporting Information Available

Relations between different measures for the ligament size; conversion of electrode potentials between different reference electrodes; reference data for surface voltammetry of single crystal of Au electrodes; reference data for Pb UPD on Au single-crystal electrodes; performance data of porous gold electrodes for methanol oxidation reaction(PDF)

## Abbreviations

AAS: atomic absorption spectroscopy
BET: Brunauer–Emmett–Teller
CO_2_RR: electrochemical carbon dioxide reduction reaction
DFT: density functional theory
DOSC: degree of selectivity control
ECL: electrogenerated chemiluminescence
EDX: energy-dispersive X-ray analysis
FDH: fructose dehydrogenase
FC: free corrosion
GOx: glucose oxidase
GD: galvanostatic dealloying
GC-MS: gas chromatography–mass spectrometry
HAADF-STEM: high-angle annular dark-field scanning transmission electron microscopy
HER: hydrogen evolution reaction
KMC: kinetic Monte Carlo (simulations)
LEED: low-energy electron diffraction
LNE: less noble element
MARI: most abundant reaction intermediate
MNE: more noble element
MSE: mercury–mercury sulfate electrode Hg|Hg_2_SO_4_|KHSO_4_
MeOH: methanol, CH_3_OH
MeFm: methyl formate HCOOCH_3_
ML: monolayers
MOR: methanol oxidation reaction
NAD^+^: nicotinamide adenine dinucleotide
NMR: nuclear magnetic resonance
NPG: nanoporous gold
OER: oxygen evolution reaction
ORR: oxygen reduction reaction
PBS: phosphate buffer saline
PD: potentiostatic dealloying
PEDOT: poly(3,4-ethylenedioxythiophene)
PFG-NMR: pulsed field gradient nuclear magnetic resonance
PMMA: poly(methyl methacrylate)
PROX: preferential CO oxidation
RHE: reversible hydrogen electrode
RVE: representative volume element
SHE: standard hydrogen electrode
SCE: saturated calomel electrode Hg|Hg_2_Cl_2_|KCl_sa_
STEM: scanning transmission electron microscopy
TAP: temporal analysis of product
TEM: transmission electron microscopy
TOF: turnover frequency
TPR(S): temperature-programmed reaction (spectroscopy)
UHV: ultrahigh vacuum
UPD: underpotential deposition/stripping
XPS: X-ray photoelectron spectroscopy.
