# Supplementary material for: Nanoporous Gold: From Structure Evolution to Functional Properties in Catalysis and Electrochemistry
Source: Chem Rev. 2023 May 3;123(10):6716–92. doi: 10.1021/acs.chemrev.2c00751 (PMC10214458; doi:10.1021/acs.chemrev.2c00751)
Supplement: Supplementary file 1 — cr2c00751_si_001.pdf [file cr2c00751_si_001.pdf]

Nanoporous Gold: From Structure Evolution to Functional Properties in Catalysis and Electrochemistry

-- Supporting Information --

Gunther Wittstock,[1] Marcus Bäumer,[2,3] Wilke Dononelli,[3,4] Thorsten Klüner,[1] Lukas Lührs,[5] Christoph Mahr,[3,6] Lyudmila V. Moskaleva,[7] Mehtap Oezaslan,[8] Thomas Risse,[9] Andreas Rosenauer,[3,6] Anne Staubitz,[10] Jörg Weissmüller,[5,11] Arne Wittstock[10]*

** Corresponding author email: wittstock@uol.de*

[1] Carl von Ossietzky University of Oldenburg, School of Mathematics and Science, Institute of Chemistry, D-26111 Oldenburg, Germany

[2] University of Bremen, Institute for Applied and Physical Chemistry, 28359 Bremen, Germany

[3] University of Bremen, MAPEX Center for Materials and Processes, 28359 Bremen, Germany

[4] University of Bremen, Bremen Center for Computational Materials Science, Hybrid Materials Interfaces Group, Am Fallturm 1, Bremen 28359, Germany

[5] Hamburg University of Technology, Institute of Materials Physics and Technology, 21703 Hamburg, Germany

[6] University of Bremen, Institute of Solid State Physics, Otto Hahn Allee 1, 28359 Bremen, Germany

[7] University of the Free State, Department of Chemistry, PO Box 339, Bloemfontein 9300, South Africa

[8] Technical University of Braunschweig, Institute of Technical Chemistry, Technical Electrocatalysis Laboratory, Franz-Liszt-Str. 35a, 38106 Braunschweig, Germany

[9] Freie Universität Berlin, Institute of Chemistry and Biochemistry, Arnimallee 22, 14195 Berlin, Germany

[10] University of Bremen, Institute for Organic and Analytical Chemistry, Leobener Str. 7, D-28359 Bremen, Germany

[11] Helmholtz-Zentrum Hereon, Institute of Materials Mechanics, 21502 Geesthacht, Germany

SI-1 Relations between Different Measures for the Ligament Size of Nanoporous Gold

Section 3 in the main text of this review introduces the following measures for the ligament size of nanoporous gold (NPG):

- L_{SEM}, an average of the diameter of ligaments, measured along their waist, typically obtained by evaluating scanning electron micrographs
- L_{G}, an average of the diameter of ligaments, measured by granulometry
- $\tilde{L}$, the characteristic spacing between the centers of neighboring ligaments, as embodied in the first maximum of the microstructure autocorrelation function
- L_{α}, the diameter of an equivalent cylindrical ligament with the same volume-specific surface area as the sample

The considerations below explore how those various measures are interrelated in NPG.

To start out, L_{α} will be linked to $\tilde{L}$, exploiting the finding that the microstructure of that material is well represented by the leveled-wave model, as presented in section 3.1. The model is generated as a superposition of plane waves with identical wavelength, λ, but different directions of the wave vector, of magnitude

$$q_0 = 2\pi/\lambda, \tag{S1}$$

and with random phase shifts. The resulting random field is then binarized ("leveled"), using the threshold value ξ for discriminating between solid and pore phase. The solid fraction, φ, and the threshold value are interlinked by[1]

$$\xi = \sqrt{2}\,\mathrm{erf}^{-1}(2\varphi - 1). \tag{S2}$$

For the leveled wave model, one finds that[1]

$$\tilde{L} = 1.23\,\frac{2\pi}{q_0} = 1.23\,\lambda, \tag{S3}$$

and for the volume-specific (per volume of the solid phase) surface area, α_{V},

$$\alpha_{\mathrm{V}} = \frac{1}{\varphi}\,\frac{2q_0}{\pi\sqrt{3}}\,e^{-\frac{\xi^2}{2}}. \tag{S4}$$

The specific surface area is linked to L_{α} by

$$\alpha_{\mathrm{V}} = \frac{4}{L_{\alpha}}. \tag{S5}$$

The numerical constant 1.23 in Eq. (S3) is related to the conversion between reciprocal space (wave number q_0, wavelength λ) and autocorrelated real space (mean distance $\tilde{L}$ between ligaments). According to the Debye scattering equation,[2,3] and except for a constant pre-factor and a constant background scattering, the structure factor for a set of randomly oriented objects with a characteristic near-neighbor spacing r_{NN} and with otherwise random positioning (for instance, a

gas of diatomic molecules) is $\frac{\sin r_{\mathrm{NN}} q}{r_{\mathrm{NN}} q}$. Numerically, its first maximum is found at the wavenumber $q_0 \approx 7.72525\, r_{\mathrm{NN}} \approx 1.23\ 2\pi\, r_{\mathrm{NN}}$.

Combinations of equations (S3) – (S5) lead to expressions for L_α and $\tilde{L}$ as the function of λ and φ:

$$\tilde{L} = 1.23\,\lambda, \tag{S6}$$

$$L_\alpha = \sqrt{3}\,\lambda\,\varphi\,\exp[\mathrm{erf}^{-1}(2\,\varphi - 1)^2], \tag{S7}$$

Contrary to $\tilde{L}$ and L_α, no analytic solution is known for L_{G} of the leveled-wave model. Analyzing the results of numerical 3D image analysis of leveled-wave model renderings, one finds that, approximately and for $0.2 < \varphi < 0.5$, L_{G} satisfies[1]

$$L_{\mathrm{G}} = \lambda\,(0.53\,\varphi + 0.41). \tag{S8}$$

We are interested in the relative magnitude of those three measures for size, and in conversion factors. With this in mind, we chose one of the three measures as the reference one. Here, somewhat arbitrarily, L_{G} is chosen for that role. We then obtain

$$\frac{\tilde{L}}{L_{\mathrm{G}}} = \frac{1.23}{(0.53\,\varphi + 0.41)} \tag{S9}$$

and

$$\frac{L_\alpha}{L_{\mathrm{G}}} = \frac{\sqrt{3}\,\varphi}{0.53\,\varphi + 0.41}\,\exp[\,\mathrm{erf}^{-1}(2\,\varphi - 1)^2]. \tag{S10}$$

Those results are displayed in Figure 11 of the main text.

Next, it will be explored how the ligament size parameter L_{SEM} relates to the aforementioned parameters. Recall that L_{SEM} is a measure for the mean diameter of ligaments, measured at their smallest cross-section. Out of the set of parameters under inspection here, one can therefore expect to L_{SEM} to have the smallest numerical value for any given sample of NPG. Comparing definitions, one finds that L_{SEM} is conceptually more closely related to L_{G} than to the remaining parameters: both, L_{SEM} and L_{G}, are based on measuring diameters. They are distinguished inasmuch as L_{G} also considers the thicker regions near the nodes, were ligaments meet, and that larger regions carry more weight in the averaging for L_{G}.[4,5] The commonalities suggest that L_{G} provides the most obvious reference to which values of L_{SEM} are to be compared. One expects L_{SEM} to be not too dissimilar from L_{G}, but systematically smaller, see the reasoning above.

As the basis for the above-mentioned comparison, one requires – for one and the same sample or set of samples – experimental data for L_{SEM} and for any one of the three remaining parameters discussed above. The literature does not provide an ultimately satisfactory database in this respect. We here analyze a single data set, from the PhD thesis work of Nadiia Mameka at Hamburg University of Technology.[6] Samples in that work were prepared and conditioned as outlined in section 2.3 of the main document of this review. Table 4.1 in ref 6 shows all required

microstructural parameters, along with L_{SEM} from scanning electron microscopy and with α_{m} determined by electrochemical capacitance measurements in 1 mol L^{-1} $HClO_4$. The relevant data from that table is reproduced here as the first 4 columns in Table S1. One can readily use the composition data to evaluate the mass density and, accounting for the solid fraction, the volume-specific surface area α_{V}. Equation (S5) then leads to L_{α}, and Eq. (S10) provides L_{G}. That data is shown as columns 5-7 in Table S1.

Table S1. Experimental microstructure data for 4 independent samples of nanoporous gold. The bottommost 4 rows show the 4 samples, and the leftmost 4 columns (residual silver fraction $x_{\mathrm{Ag}}^{\mathrm{res}}$, solid fraction φ, mass-specific surface area α_{m}, scanning-electron-microscope based ligament size L_{SEM}) are from Table 4.1 in ref 6. The 3 remaining columns (volume-specific surface area α_{V}, area-based ligament size L_{α}, granulometry-based ligament size L_{G}) are estimated as explained in the text.

	$x_{\mathrm{Ag}}^{\mathrm{res}}$ no units	φ no units	α_{m} [m²/g]	L_{SEM} [nm]	α_{V} [µm⁻¹]	L_{α} [nm]	L_{G} [nm]
sample 1	15.7 ± 0.2	0.28 ± 0.02	7.2 ± 0.4	15 ± 5	129 ± 6	31 ± 2	30 ± 2
sample 2	3.1 ± 2.5	0.27 ± 0.01	6.1 ± 1.8	20 ± 5	115 ± 34	35 ± 10	34 ± 10
sample 3	0.5 ± 0.2	0.29 ± 0.02	3.8 ± 0.4	40 ± 5	74 ± 8	54 ± 6	54 ± 6
sample 4	7.3 ± 2.1	0.32 ± 0.03	1.0 ± 0.6	150 ± 5	19 ± 11	210 ± 120	208 ± 120

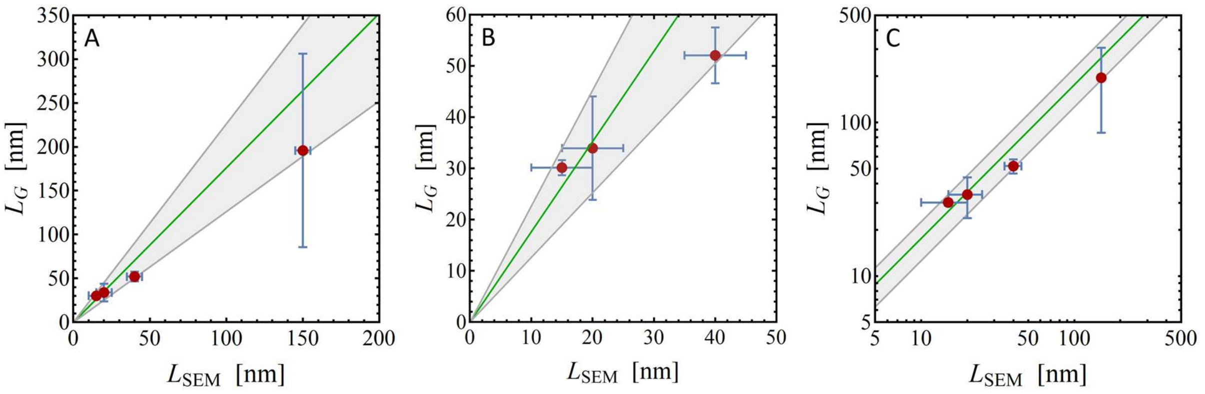

Figure S1. Implication of the data in reference 18 for the comparison between scanning-electron-microscopy-based ligament size, L_{SEM} , and the granulometry-based ligament size, L_{G}, in a small set of samples with solid fraction φ around 0.3. A) and B) plots with linear axes in different scaling; C) plot with double logarithmic axis. Circles and error bars refer to experimental data. Green line is best fit of straight line through the origin. Gray lines and shaded regions indicate a symmetric confidence band containing all data points.

Figure S1 explores the correlation between the data for expects L_{SEM} and for L_{G} as displayed in Table S1. In view of the extremely slim database, only the simplest model can be tested in a meaningful way. That is here a linear scaling, independent of the solid fraction, in other words $L_{\mathrm{G}}(L_{\mathrm{SEM}}, \varphi) = const\ L_{\mathrm{SEM}}$. The green lines in the figure panels represent the straight line of best

fit through the origin on a linear scale. The gray lines represent a symmetric confidence band including all data points. That data can be represented numerically by

$$L_{\mathrm{G}} = (1.8 \pm 0.5)\, L_{\mathrm{SEM}} \tag{S11}$$

The various representations in Figure S1 communicate clearly that the data provides only a weak confirmation of the linear scaling. Therefore, the scaling factor and its confidence interval, as derived from fitting the data set, must be viewed as a tentative result. Additional experiments are needed as a basis for a reliable conclusion on the scaling between L_{SEM} and other measures for the characteristic microstructural length scale of NPG. The result, as it stands, however confirms the expectation, based on the definitions of the various size parameters, that L_{SEM} provides a particularly small numerical value compared to the remaining measures, likely about twice smaller than the granulometry ligament size.

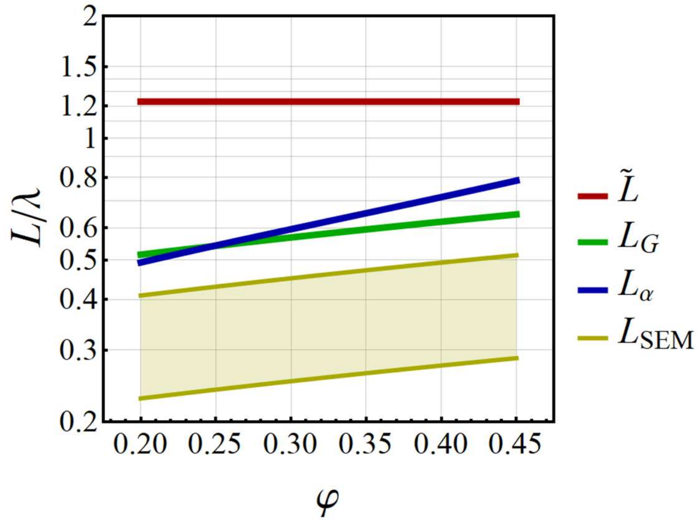

Figure S2. The various size measures (as identified in the legend and explained in the text) for characteristic length scales of the leveled-wave model as a representation of nanoporous gold. Each measure has been scaled with the characteristic wavelength, λ, used in constructing the model microstructure. Data for L_{SEM} must be viewed as tentative, see discussion in the main text.

Figure S2 summarizes the findings for the various size measures in a graph where each measure has been scaled with the characteristic wavelength λ. Furthermore, Figure 11 in section 3.5 of the main document shows the same data but with the sizes scaled with L_G. The compilations again advertise the large differences in the numerical values of the differently defined size measures. It is emphasized that the differences, in themselves, do not represent "error" or other inconsistency between the measures. It is simply a consequence of their different definitions. There is, however, a large uncertainty in how to convert between the SEM ligament size and the remaining sizes. That relation calls for a dedicated experimental study, which is not covered by literature so far.

SI-2 Conversion of the Potential Scale Between Commonly Used Reference Electrodes

The papers reviewed in this article use a large variety of reference electrodes that have a thermodynamically defined electrode potential with respect to the standard hydrogen electrode (SHE). Except for statements where a direct comparison is required, we avoided recalculated electrode potentials to a single reference electrode. Instead, we provide below the potentials of commonly used reference electrodes in the field of NPG (Table S2). The SHE has by definition an electrode potential of 0.0000000 V at all temperatures. It is formed by a Pt metal in contact with gaseous hydrogen at the pressure of 100 kPa and an aqueous solution with a hydronium ion *activity* $a(\mathrm{H_3O^+}) = 1\ \mathrm{mol\ L^{1}}$. This solution has the pH 0. A solution of HCl fulfilling this condition has approximately a *concentration* of 1.2 mol $\mathrm{L^{1}}$. If instead, the half-cell of the hydrogen electrode is prepared with an HCl solution of the concentration 1 mol $\mathrm{L^{1}}$, this electrode is called normal hydrogen electrode (NHE). Its use as reference point is discouraged by IUPAC. Another hydrogen electrode is called reversible hydrogen electrode (RHE). This is a Pt metal in contact with gaseous hydrogen of pressure 100 kPa and the working solution of the cell. Equation (S12) can be used to convert an electrode potential measured against SHE, E_{SHE}, to a potential measured against RHE, E_{RHE}, or vice versa.

$$\begin{aligned} E_{\mathrm{RHE}} &= E_{\mathrm{SHE}} + 2.3\frac{RT}{F}\lg\frac{a(\mathrm{H_3O^+})}{a^\circ} \\ &\approx E_{\mathrm{SHE}} - 0.059\ \mathrm{V}\cdot\mathrm{pH}\ \text{for}\ T = 298.15\ \mathrm{K} \end{aligned} \tag{S12}$$

The experimental advantage of using a RHE rests in the avoidance of any diffusion potentials between the inner filling solution of a reference electrode in contact to a working solution that differs in composition from the filling of the reference electrode. Conceptually, the RHE has the advantage that potentials of electrode reactions involving the transfer of protons remain constant vs RHE, but would show a pH-dependence when stated against SHE. This applies to such important reactions as hydrogen evolution reaction (HER), oxygen evolution reaction (OER), oxygen reduction reaction (ORR), methanol oxidation reaction (MOR) etc.

Table S2. Potentials of Commonly Used Reference Electrodes with Respect to the Standard Hydrogen Electrode (SHE) at 298 K

**Half cell**	***E* / V vs SHE**
Hg | Hg_2SO_4 | H_2SO_4 (*aq*, *c* = 0.5 mol L^{-1}) (	+0.679
Hg | Hg_2SO_4 | K_2SO_4 (*aq*, sa)	+0.640
Hg | Hg_2Cl_2 | KCl(*aq*, *c* = 0.1 mol L^{-1})	+0.334
Hg | Hg_2Cl_2 | KCl(*aq*, *c* = 1 mol L^{-1}) (NCE, normal calomel electrode)	+0.280
Hg | Hg_2Cl_2 | KCl(*aq*, *sa*) (SCE, saturated calomel electrode)	+0.241
Hg | Hg_2Cl_2 | NaCl (*aq*, sa) (SSCE sodium chloride-saturated calomel electrode)	+0.236
Ag | AgCl | KCl(*aq*, *c* = 0.1 mol L^{-1})	+0.290
Ag | AgCl | KCl(*aq*, *c* = 1 mol L^{-1})	+0.236
Ag | AgCl | KCl(*aq*, *c* = 3 mol L^{-1})	+0.210
Ag | AgCl | NaCl(*aq*, *c* = 3 mol L^{-1})	+0.209
Ag | AgCl | KCl(*aq*, *sa*)	+0.197
Hg | HgO | NaOH (*aq*, *c* = 0.1 mol L^{-1})	+0.165
Hg | HgO | KOH (*aq*, *c* = 1 mol L^{-1})	+0.107
Pt | H_2 (100 kPa), H^+(*aq*) (*a*(H^+) = 1 mol L^{-1}) (SHE)	0.00000000
Pt | H_2 (100 kPa), H^+(*aq*) (*c*(H^+) = 1 mol L^{-1}) (NHE)	-0.006
Pt | H_2 (100 kPa), H^+(*aq*) (*a*(H^+) = 0.1 mol L^{-1}) (RHE, pH 1)	-0.059
Pt | H_2 (100 kPa), H^+(*aq*) (*a*(H^+) = 0.01 mol L^{-1}) (RHE, pH 2)	-0.118
Pt | H_2 (100 kPa), H^+(*aq*) (*a*(H^+) = 10^{-3} mol L^{-1}) (RHE, pH 3)	-0.177
Pt | H_2 (100 kPa), H^+(*aq*) (*a*(H^+) = 10^{-4} mol L^{-1}) (RHE, pH 4)	-0.236
Pt | H_2 (100 kPa), H^+(*aq*) (*a*(H^+) = 10^{-5} mol L^{-1}) (RHE, pH 5)	-0.295
Pt | H_2 (100 kPa), H^+(*aq*) (*a*(H^+) = 10^{-6} mol L^{-1}) (RHE, pH 6)	-0.354
Pt | H_2 (100 kPa), H^+(*aq*) (*a*(H^+) = 10^{-7} mol L^{-1}) (RHE, pH 7)	-0.413
Pt | H_2 (100 kPa), H^+(*aq*) (*a*(H^+) = 10^{-8} mol L^{-1}) (RHE, pH 8)	-0.472
Pt | H_2 (100 kPa), H^+(*aq*) (*a*(H^+) = 10^{-9} mol L^{-1}) (RHE, pH 9)	-0.531
Pt | H_2 (100 kPa), H^+(*aq*) (*a*(H^+) = 10^{-10} mol L^{-1}) (RHE, pH 10)	-0.590
Pt | H_2 (100 kPa), H^+(*aq*) (*a*(H^+) = 10^{-11} mol L^{-1}) (RHE, pH 11)	-0.649
Pt | H_2 (100 kPa), H^+(*aq*) (*a*(H^+) = 10^{-12} mol L^{-1}) (RHE, pH 12)	-0.708
Pt | H_2 (100 kPa), H^+(*aq*) (*a*(H^+) = 10^{-13} mol L^{-1}) (RHE, pH 13)	-0.767
Pt | H_2 (100 kPa), H^+(*aq*) (*a*(H^+) = 10^{-14} mol L^{-1}) (RHE, pH 14)	-0.826

Frequently, the solution composition is given as concentration. For acidic solution, the pH can be conveniently be measured. For alkaline solution with pH > 12, this is more difficult due to the alkali error of common pH glass electrodes. Thus, activity coefficients have to be considered when estimating the potential of the RHE in a solution of a particular concentration of a base.

$$E_{\text{RHE}} = E_{\text{SHE}} + 2.3\frac{RT}{F}\lg\frac{a(\text{H}_3\text{O}^+)}{a^\circ}$$
$$= E_{\text{SHE}} + 2.3\frac{RT}{F}\lg\frac{c(\text{H}_3\text{O}^+)}{c^\circ} + 2.3\frac{RT}{F}\lg f_c(\text{H}_3\text{O}^+) \quad \text{(S13)}$$

For a base this gives

$$E_{\text{RHE}} = E_{\text{SHE}} + 2.3\frac{RT}{F}\lg\left[\frac{10^{-14}\text{mol}^2\text{L}^{-2}}{a^\circ a(\text{OH}^-)}\right]$$
$$= E_{\text{SHE}} - 2.3\frac{RT}{F}\cdot 14 - 2.3\frac{RT}{F}\lg\frac{c(\text{OH}^-)}{c^\circ} - 2.3\frac{RT}{F}\lg f_c(\text{OH}^-) \quad , \quad \text{(S14)}$$
$$\approx E_{\text{SHE}} - 0.059\,\text{V}\cdot\left[14 + \lg\frac{c(\text{OH}^-)}{c^\circ}\right] - 0.059\,\text{V}\cdot\lg f_c(\text{OH}^-) \quad \text{for } T = 298\,\text{K}$$

with c° = 1 mol L^{-1} and a° = 1 mol L^{-1} being the standard concentrations and standard activities. A selection of activity coefficients from Ref 7 is compiled in Table S3. Please note, that the content of the solution is stated a molality b_i, i.e.,

$$b_i = \frac{\text{Amount of compound } i}{\text{Mass of the solvent}} = \frac{N_i}{m_{\text{solv}}} \quad \text{(S15)}$$

Since water as the solvent has a density close to 1 kg L^{-1} and additions of electrolytes changes this only by a small amount, estimations are often sufficiently accurate by assuming

$$\frac{b_i}{\text{mol}\,\text{kg}^{-1}} \approx \frac{c_i}{\text{mol}\,\text{L}^{-1}} \quad \text{(S16)}$$

Of course, precision data especially for higher concentrations require a transformation of the quantities taking in account the density of the solution and using interpolation techniques. However, the correction introduced by considering an activity coefficient f_c of 0.9 is +2.7 mV and for f_c = 0.6 it is +13.1 mV. Thus, the correction may be comparable to other uncertainties in the electrode potential especially if a reference electrode with a diaphragm (e.g., SCE, Ag|AgCl, $Hg|Hg_2SO_4|K_2SO_{4(sa)}$) is used that is prone to the formation of liquid junction potentials.

Table S3. Activity Coefficients of Electrolytes Commonly Used in the Work with NPG

b **[$\text{mol}_{\text{electrolyte}}$ $\text{kg}_{\text{sovent}}$-1]**	**f_b(NaOH)**	**f_b(KOH)**	**f_b($HClO_4$)**	**f_b(H_2SO_4)**
0.1	0.766	0.798	0.803	0.265
0.5	0.690	0.732	0.769	0.154
1.0	0.678	0.756	0.823	0.130
5	1.181	1.72	3.11	0.212

The different reference electrode potentials exhibit different dependencies on temperature, e.g., via the temperature dependence of solubility products. Therefore, the values in Table S2 are valid only for 298 K. For other temperatures, the temperature functions of the reference electrodes must be considered. They are typically available from the manufacturers. A detailed consideration of this issues goes beyond the scope of this review.

Occasionally, one needs the conversion of electrodes potential [V] measured against a reference electrode to the vacuum energy scale [eV]. According to IUPAC,[8] equation (S17) is recommended.

$$E_{\mathrm{vac}} = -e \cdot E_{\mathrm{SHE}} - (4.44 \pm 0.02)\ \mathrm{eV} \qquad \text{(S17)}$$

Apart from an offset and a different unit, the potential axes have opposing directions as illustrated in Figure S3.

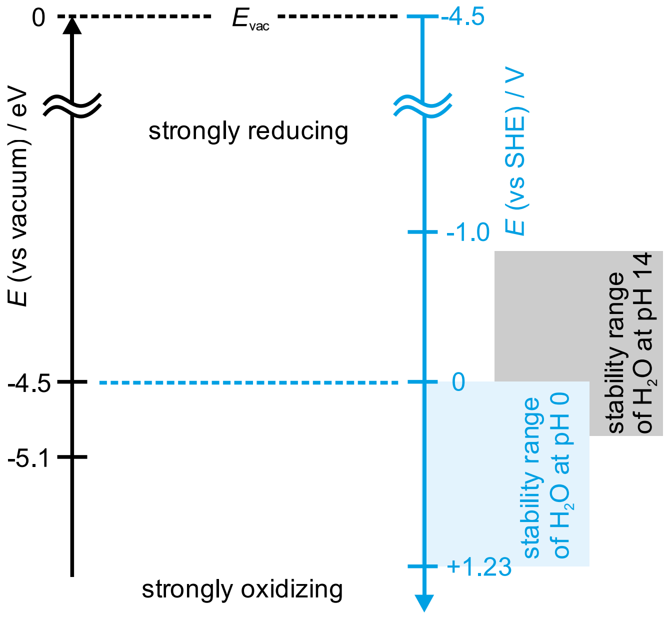

Figure S3. Schematic illustration of the vacuum energy scale and the electrode potential scale.

SI-3 Voltammetry of Single Crystal Gold Electrodes

The surface voltammetry is often considered as a superposition of the contribution of the most stable, low-index crystal faces of planar Au electrodes. While this approach naturally neglects the high fraction of low-coordinated surface atoms that are typical for NPG, it provides a rough indication about the dominating facets and can capture changes of the surface structure during use of NPG electrodes.

Figure S4 exemplifies such a data set in acidic perchlorate solution. The review paper [9] is recommended as an initial source showing the complex dependency of the appearance of surface voltammetry on the kind and concentration of the used electrolyte, especially the presence of anion and pH, the temperature, scan rate and the quality of the used single crystal electrodes. A more extensive collection of reference data for various electrolyte composition and single crystal faces is compiled in Table S4. Recently application in alkaline solution have gained in importance and Figure S5 provides reference voltammograms for such conditions.

Table S4. References to Flat Au Single Crystal Voltammetry in Different Electrolytes

Electrolyte	Facet	Ref.
0.002, 0.005 and 0.1 mol L^{-1} $HClO_4$	(1 0 0)	9
0.01 mol L^{-1} $HClO_4$	(1 1 1), (1 1 0), (1 0 0)	9
0.01 mol L^{-1} $HClO_4$	(9 1 1), (7 5 5), (7 7 1), (5 5 4), (5 5 1), (5 3 3), (5 1 1), (4 1 0), (4 1 1), (3 3 2), (3 3 1), (3 2 0), (3 1 0), (3 1 1), (2 2 1), (2 1 0), (2 1 1),	10
7 mol L^{-1} $HClO_4$	(1 1 1)	9
0.01 mol L^{-1} $KClO_4$	(1 1 1)	9
0.05 mol L^{-1} H_2SO_4	(1 1 1), (1 0 0)	11
0.1 mol L^{-1} H_2SO_4	(1 1 1), (1 1 0), (1 0 0)	12
0.32 mol L^{-1} H_2SO_4	(1 1 1)	9
0.5 mol L^{-1} H_2SO_4	(1 1 1)	9
0.09 mol L^{-1} $NaClO_4$ + 0.01 mol L^{-1} $HClO_4$	(1 1 1), (1 1 0), (1 0 0)	9
0.01 mol L^{-1} H_2SO_4	(1 1 1), (1 1 0), (1 0 0)	9
0.01 mol L^{-1} H_2SO_4	(3 1 1)	10
0.011 mol L^{-1} KPF_6	(1 1 1), (1 1 0), (1 0 0)	9
0.1 mol L^{-1} NaOH	(1 1 1), (1 1 0), (1 0 0)	13

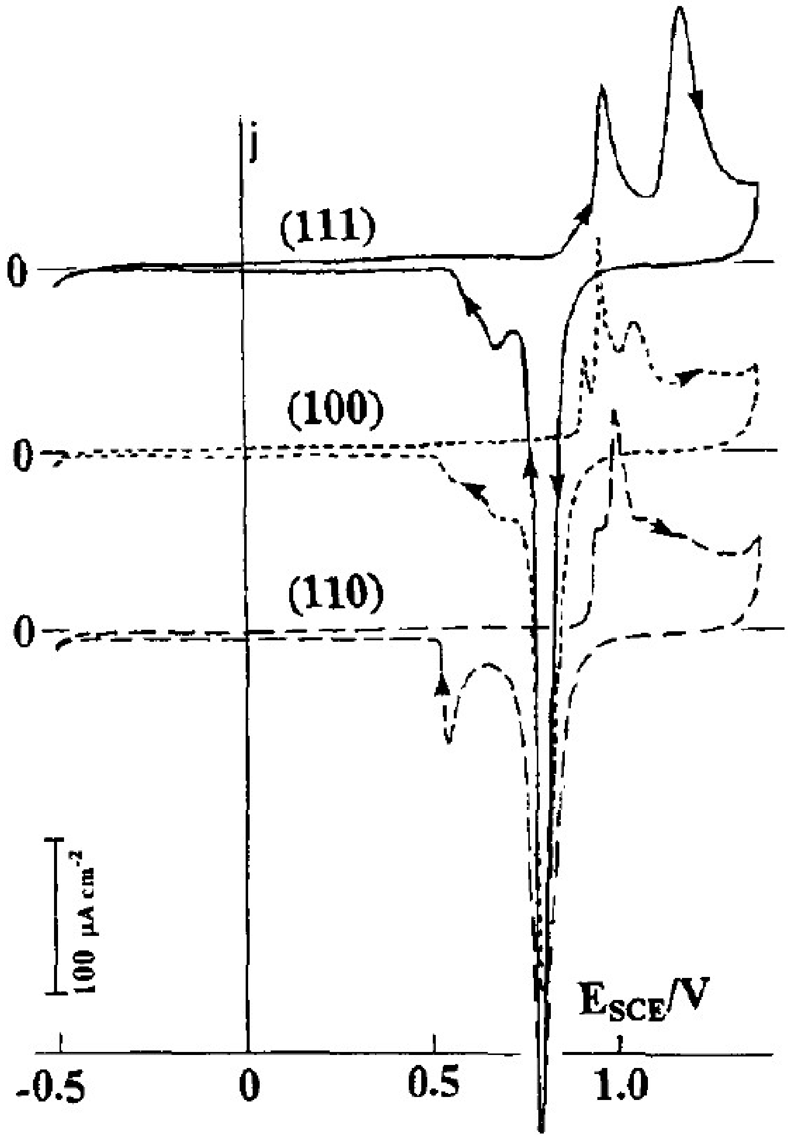

Figure S4. Cyclic voltammograms of flat single crystal Au(1 1 1), Au(1 0 0) und Au(1 1 0) electrodes in 0.09 mol L^{-1} $NaClO_4$ + 0.01 mol L^{-1} $HClO_4$ at T = (298 ± 2) K at v = 50 mV s^{-1}.[9] Reprinted from J. Electroanal. Chem., 407, Hamelin, A., Cyclic Voltammetry of Gold Single-Crystal Electrodes. Part 1. Behavior of Low-Index Faces. pp. 1-11, Copyright (1996) with permission from Elsevier. Original data from ref 14.

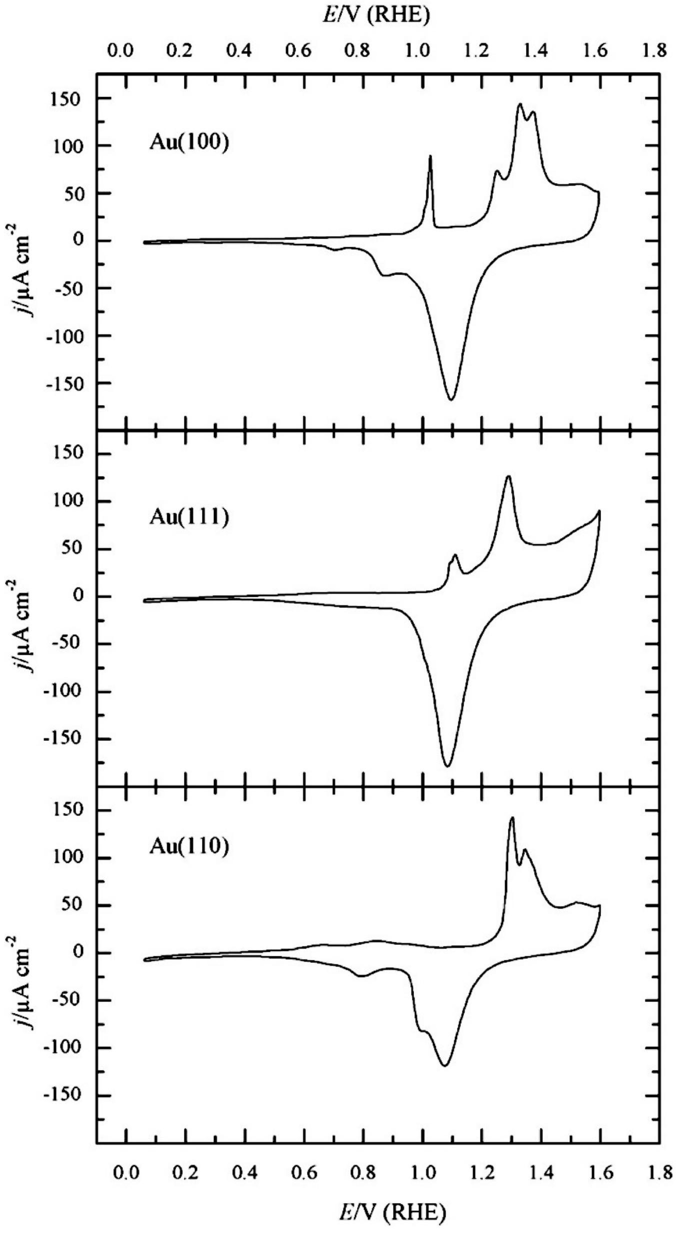

Figure S5. Cyclic voltammograms of Au(1 0 0), Au(1 1 0) and Au(1 1 1) electrodes in 0.1 M NaOH at v = 50 mV s^{-1}.[13] Reprinted from Electrochim. Acta, 52, Hernández, J.; Solla-Gullón, J.; Herrero, E.; Aldaz, A.; Feliu, J. M. Methanol Oxidation on Gold Nanoparticles in Alkaline Media: Unusual Electrocatalytic Activity, pp 1662–1669, Copyright (2006) with permission from Elsevier.

SI-4 Reference Data for Underpotential Deposition/Stripping of Pb on Au Single Crystal Electrodes

Extensive experimental material for Pb UPD on Au single crystal electrodes is available from the initial period of singly crystal electrochemistry.[15,16] The experiments were conducted in 10^{-3} mol L^{-1} PbF_2 + 10^{-2} mol L^{-1} $HClO_4$, a system in which no specific anion adsorption is expected to interfere with the UPD process. Later, it turned out the Pb UPD on Au electrodes is quite insensitive to the presence of anions (in contrast to Cu UPD on Au). The data even agree quite well with vacuum deposition as long as the amount of vacuum deposited Pb does not exceed a monolayer. Hence water adsorption does not significantly interfere with the UPD process. The absence of surface alloying that would cause Pb to diffuse into the Au electrode and give time-dependent data for the dissolution of the UPD layer is a further prerequisite for the use of Pb UPD on Au as a structure sensitive probe. Finally, the electrosorption valency is close to 2 and thus equal to the charge of the Pb^{2+} ion. It does not vary significantly with the adsorption site. This is important for relating charges of the UPD process to the number of adsorbed Pb adatoms and thus to the available site of a certain structure.[16]

Figure S6 to S8 show the voltammetric curves for the dissolution of a complete Pb UPD layer from the three low index single crystal Au electrodes. For Au(1 1 1) and Au(1 1 0) the deposition curves are symmmetric to the stripping curve and are not shown. Note the asymmetry for the first cycle for Au(1 0 0) in Figure S8. The superstructures of Pb on the Au substrates given in Figures S6 to S8 were determine by low-energy electron diffraction (LEED) in vacuum.

Stepped surface representing the transition between the low-index faces shown in Figure S9 to S11. It is very clear that the assignment of on a signal from a NPG surface to only a very limited number of structural elements from low-index faces is a very strong simplification of the complexity of the UPD signals even for a well-behaved system like Pb UPD on Au.

Data in alkaline solution show similarity to those obtained in acidic solution (Figure S12).[17] The application of such single crystal data for the analysis of polycrystalline Au electrodes and Au electrodes with small terrace size (nanoparticles and nanorods) has been exemplified[18] and is typically applied in analogous way to NPG.

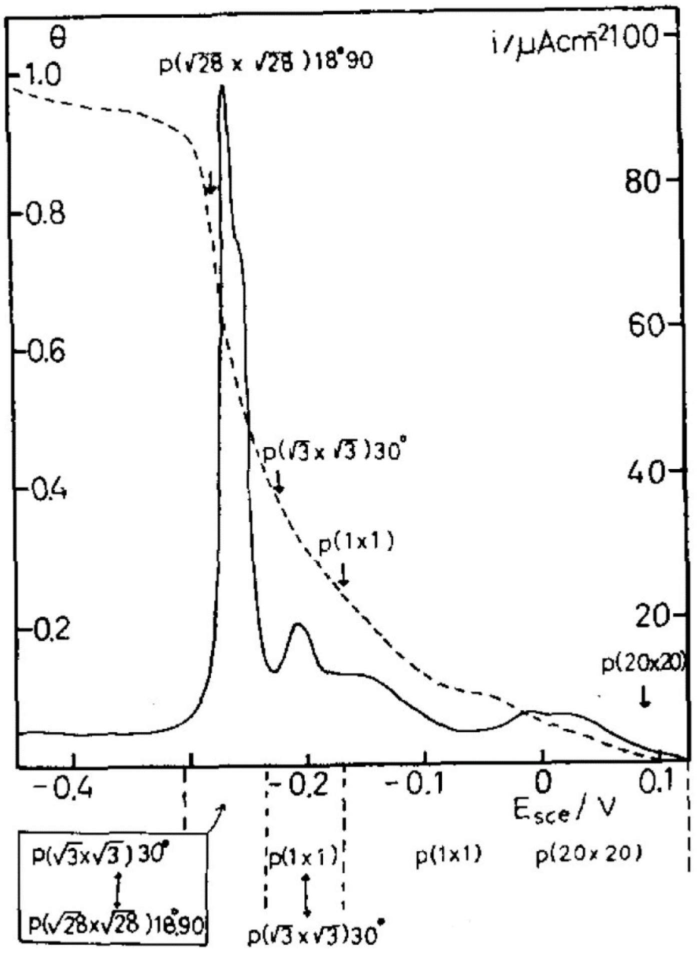

Figure S6. Positive going scan (stripping) of a cyclic voltammogram (—, right ordinate) of Pb UPD at flat Au(1 1 1) and the resulting Pb coverage (---, left ordinate) the labels indicate the superstructures found by LEED; 10^{-3} mol L^{-1} PbF_2 + 10^{-2} mol L^{-1} $HClO_4$, v = 20 mV s^{-1}.[16] Reprinted from J. Electroanal. Chem., 171, Hamelin, A.; Lipkowski, J., Underpotential Deposition of Lead on Single Crystal Faces: Part II. General Discussion, pp 317-330, Copyright (1984) with permission from Elsevier.

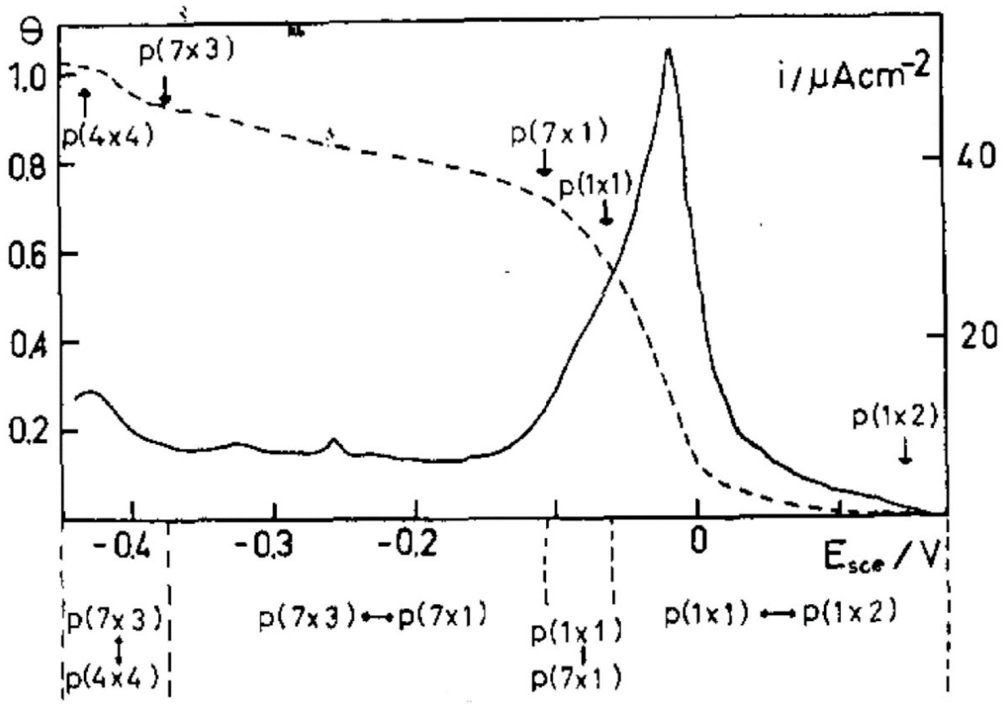

Figure S7. Positive going scan (stripping) of a cyclic voltammogram (—, right ordinate) of Pb UPD at flat Au(1 1 0) and the resulting Pb coverage (---, left ordinate) the labels indicate the superstructures found by LEED; 10^{-3} mol L^{-1} PbF_2 + 10^{-2} mol L^{-1} $HClO_4$, v = 20 mV s^{-1}.[16] Reprinted from J. Electroanal. Chem., 171, Hamelin, A.; Lipkowski, J., Underpotential Deposition of Lead on Single Crystal Faces: Part II. General Discussion, pp 317-330, Copyright (1984) with permission from Elsevier.

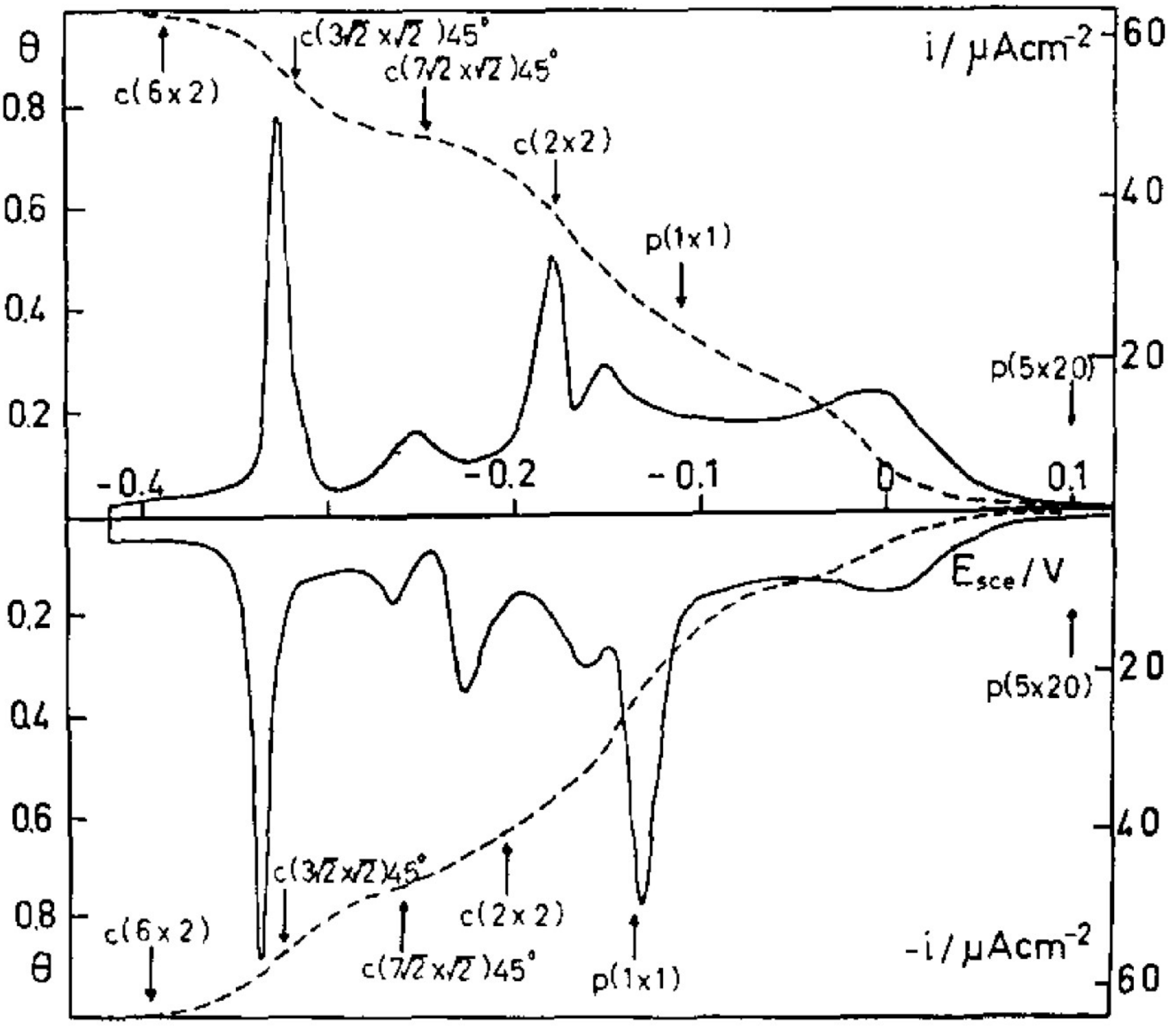

Figure S8. Cyclic voltammogram (—, right ordinate, first cycle) of Pb UPD at flat Au(1 0 0) and the resulting Pb coverage (---, left ordinate) the labels indicate the superstructures found by LEED; 10^{-3} mol L^{-1} PbF_2 + 10^{-2} mol L^{-1} $HClO_4$, v = 20 mV s^{-1}.[16] Reprinted from J. Electroanal. Chem., 171, Hamelin, A.; Lipkowski, J., Underpotential Deposition of Lead on Single Crystal Faces: Part II. General Discussion, pp 317-330, Copyright (1984) with permission from Elsevier.

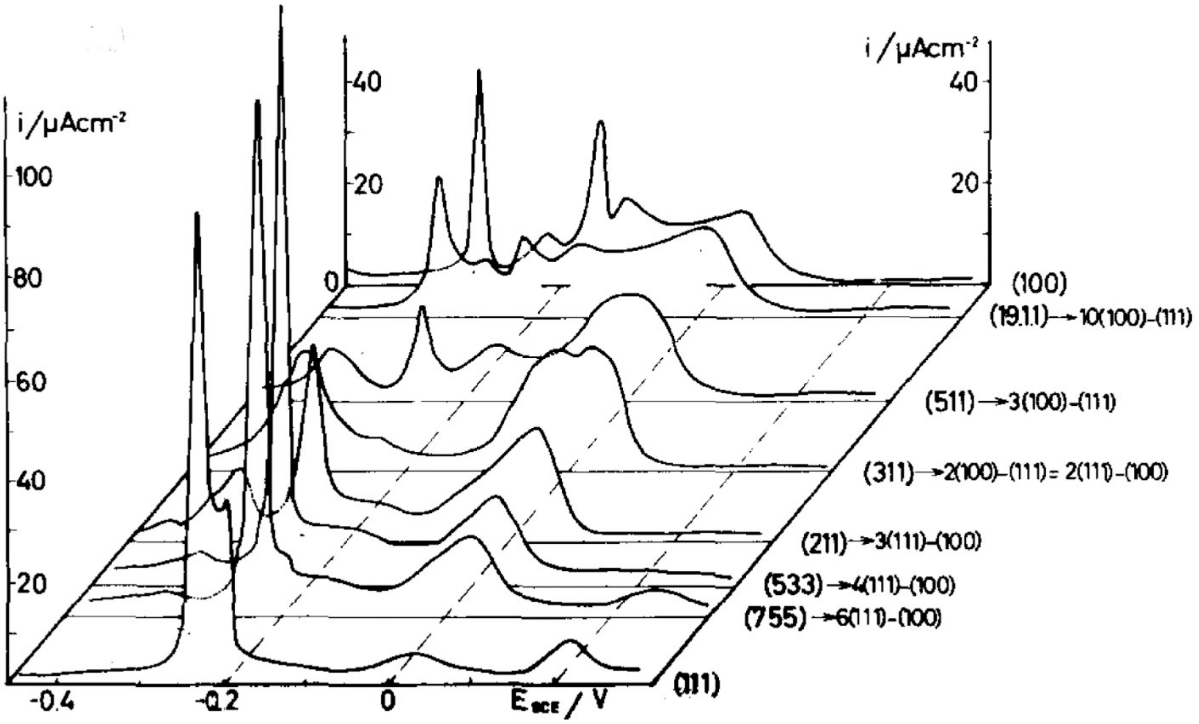

Figure S9. Collections of positive-going half scans (stripping) of cyclic voltammograms of Pb UPD for stepped surface in different angles between flat Au(1 1 1) and Au(1 0 0); 10^{-3} mol L^{-1} PbF_2 + 10^{-2} mol L^{-1} $HClO_4$, v = 20 mV s^{-1}.[16] Reprinted from J. Electroanal. Chem., 171, Hamelin, A.; Lipkowski, J., Underpotential Deposition of Lead on Single Crystal Faces: Part II. General Discussion, pp 317-330, Copyright (1984) with permission from Elsevier.

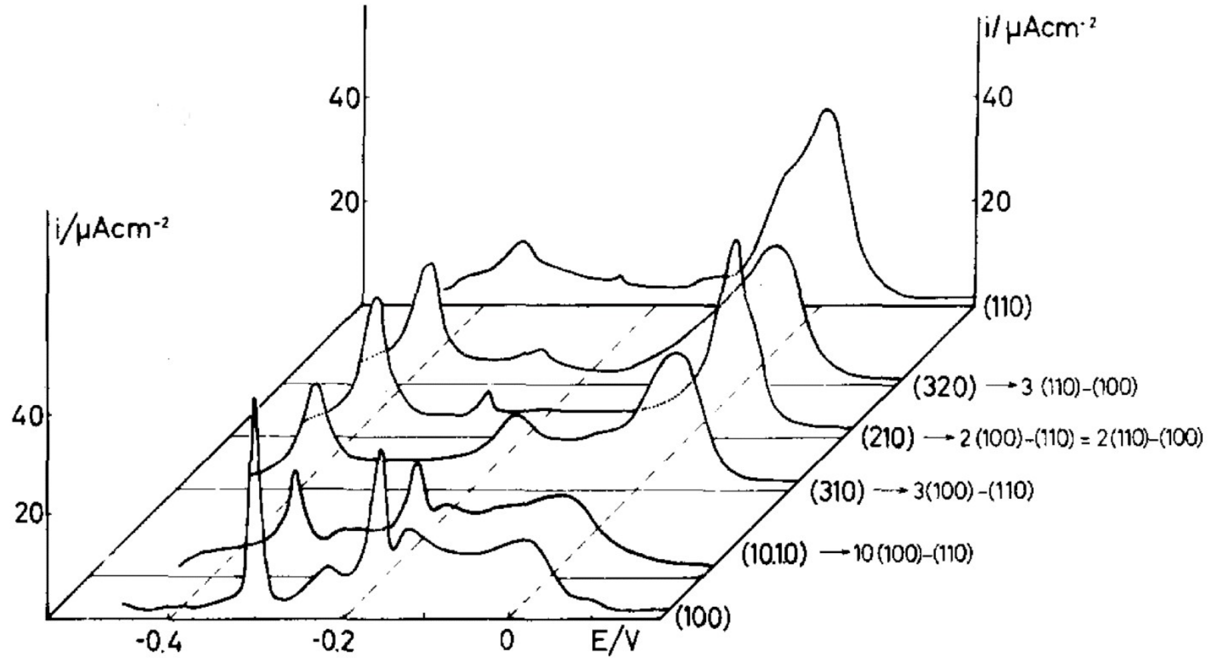

Figure S10. Collections of positive-going half scans (stripping) of cyclic voltammograms of Pb UPD for stepped surface in different angles between flat Au(1 0 0) and Au(1 1 0); 10^{-3} mol L^{-1} PbF_2 + 10^{-2} mol L^{-1} $HClO_4$, v = 20 mV s^{-1}.[16] Reprinted from J. Electroanal. Chem., 171, Hamelin, A.; Lipkowski, J., Underpotential Deposition of Lead on Single Crystal Faces: Part II. General Discussion, pp 317-330, Copyright (1984) with permission from Elsevier.

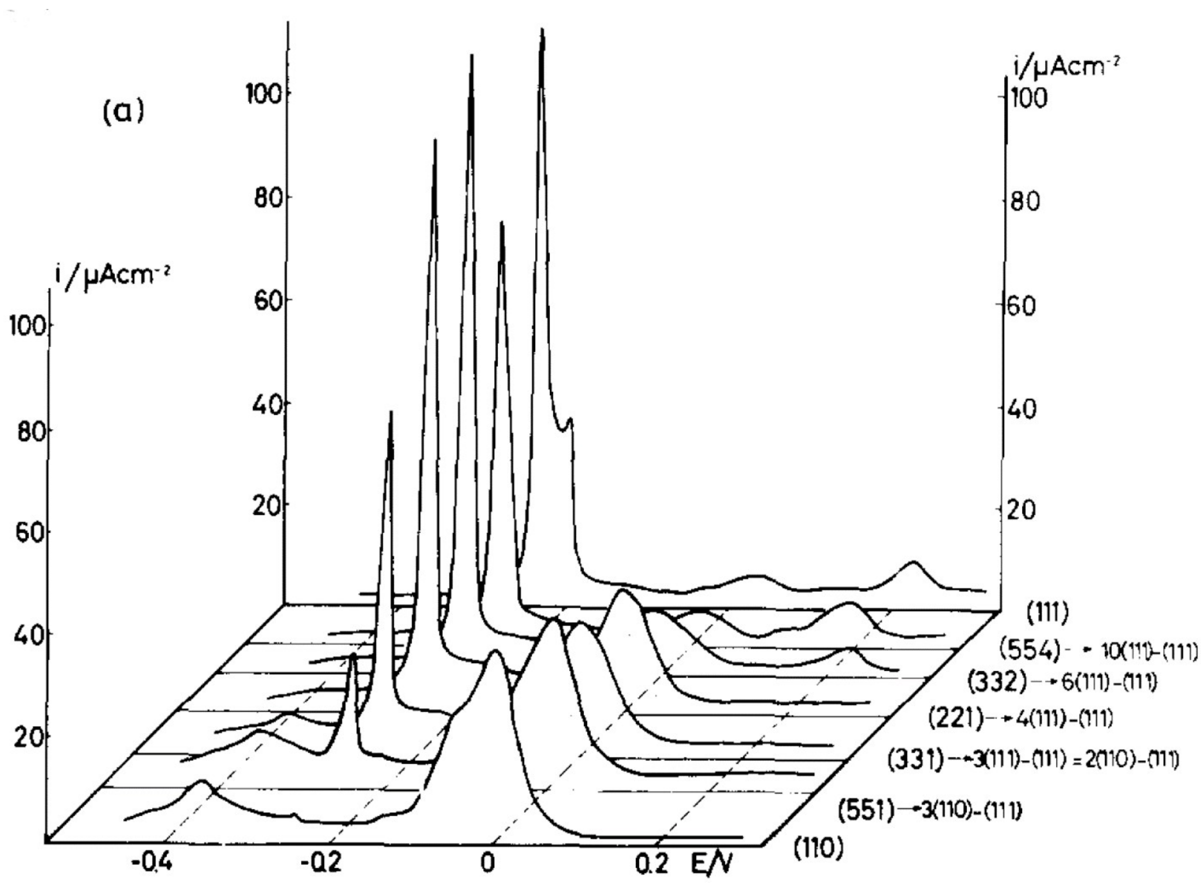

Figure S11. Collections of positive-going half scans (stripping) of cyclic voltammograms of Pb UPD for stepped surface in different angles between flat Au(1 1 0) and Au(1 1 1); 10^{-3} mol L^{-1} PbF_2 + 10^{-2} mol L^{-1} $HClO_4$, v = 20 mV s^{-1}.[16] Reprinted from J. Electroanal. Chem., 171, Hamelin, A.; Lipkowski, J., Underpotential Deposition of Lead on Single Crystal Faces: Part II. General Discussion, pp 317-330, Copyright (1984) with permission from Elsevier.

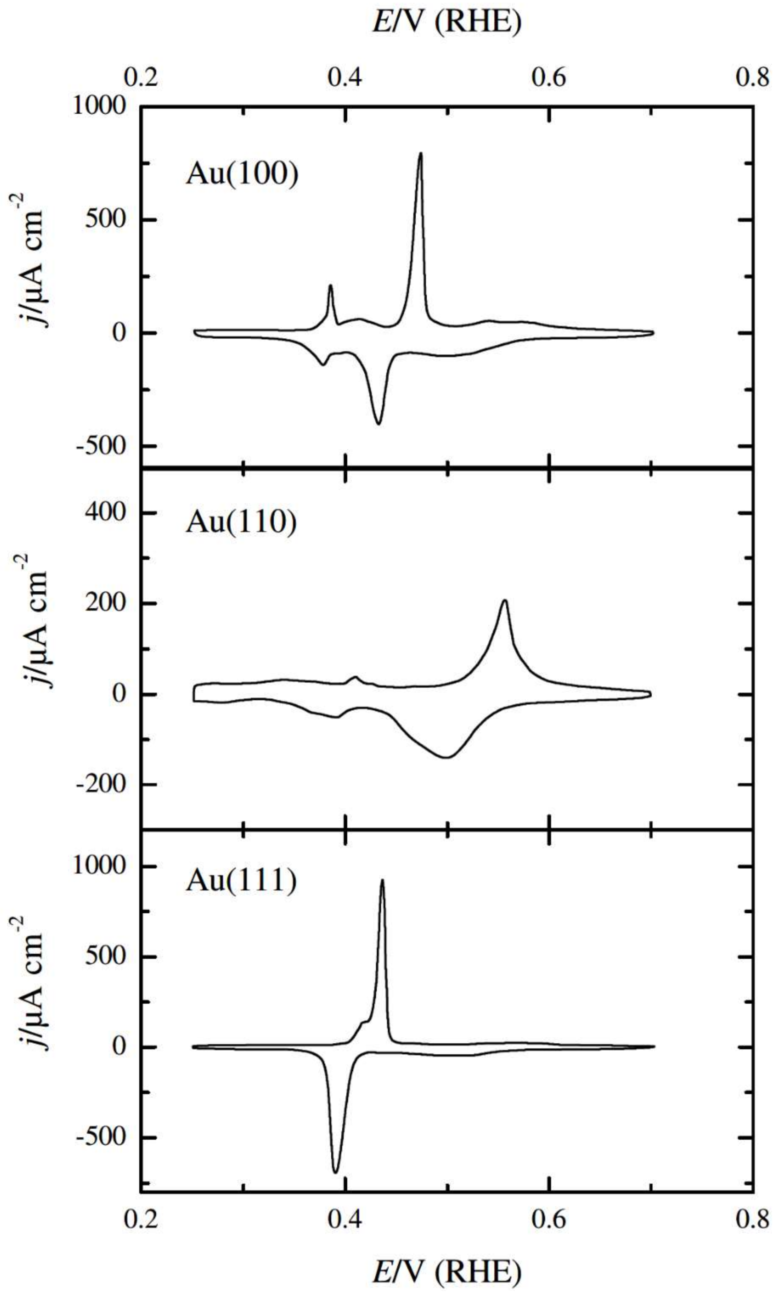

Figure S12. Cyclic voltammograms of Pb UPD at flat Au(1 0 0), Au(1 1 0) and au(1 1 1) in 10^{-3} M $Pb(NO_3)_2$ + 0.1 mol L^{-1} NaOH, v = 50 mV s^{-1}.[17] Reprinted from J. Electroanal. Chem., 574, Hernández, J.; Solla-Gullón, J.; Herrero, E.; Electrochemical Characterization and Effect of the Surface Structure on the Oxygen Reduction Reaction, pp 185-196, Copyright (2004) with permission from Elsevier.

SI-5 Performance Data for Porous Gold Electrode for Methanol Oxidation Reaction

Table S5. Performance Data of Selected NPG Electrodes for Methanol Oxidation Reaction in Cyclic Voltammograms; the Lines Shaded in Blue Were Used for Table 1 in the Main Text

Electrode, preparation route	Electrode Thickness [µm]	Surface Area Determination	Electrolyte	v [mV s^{-1}]	E_p [V vs RHE][a]	j_p [mA cm^{-2}][b]	Ref.	Comment
Potentiostatic dealloying $Ag_{77}Au_{22}Pt_1$ in 0.77 mol L^{-1} $HClO_4$ passing a charge of 5 C cm^{-2}	7 µm of a 200 µm-thick sheet	Capacitance measurement 0.44 V and 0.69 V in 1 mol L^{-1} $HClO_4$, calculated with 28 µF cm^{-2}	1 mol L^{-1} KOH + 5 mol L^{-1} KOH	10	0.69<br>-0.3 (Hg|HgO)	0.21	[19]	Samples were incompletely dealloyed, Pt inhibited surface diffusion, large excess of KOH vs MeOH
Potentiostatic dealloying $Ag_{77}Au_{22}Pt_1$ in 0.77 mol L^{-1} $HClO_4$ passing a charge of 5 C cm^{-2}, subsequent annealing in air at 425 °C for 2 h	7 µm of a 200 µm-thick sheet	Capacitance measurement at 0.40 V and 0.64 V in 1 mol L^{-1} $HClO_4$, calculated with 28 µF cm^{-2}	1 mol L^{-1} MeOH + 5 mol L^{-1} KOH	10	0.69<br>-0.3 (Hg|HgO)	0.92	[19]	Samples were incompletely dealloyed, Pt inhibited surface diffusion, large excess of KOH vs MeOH
Dealloying $Al_{66}Au_{27.2}Pt_{3.4}Pd_{3.4}$ in 20 % (*m*/*m*) NaOH, final composition x_{Pt} = 10 %, x_{Pd} = 10%	25 µg NPG auf GCE Ø4 mm	Surface area was not reported	1 mol L^{-1} MeOH + 1 mol L^{-1} KOH	50	0.83<br>-0.1 V (Hg|HgO)	870 mA cm$^{-2}_{GCE}$ mg$_{metal}^{-1}$	[20]	processed to catalyst inkwith Nafion , A_{ECSA} not reported
Dealloying $Al_{66}Au_{27.2}Pt_{6.8}$ in 20 % (*m*/*m*) NaOH, final composition x_{Pt} = 10%	25 µg NPG auf GCE Ø4 mm	Surface area was not reported	1 mol L^{-1} MeOH + 1 mol L^{-1} KOH	50	0.83 V<br>-0.1 V (Hg|HgO)	694 mA cm$^{-2}_{GCE}$ mg$_{metal}^{-1}$	[20]	processed to catalyst ink with Nafion, A_{ECSA} not reported
Free corrosion of $Au_{14}Ag_{14}Cu_{72}$ nanoparticles in 3 mol L^{-1} HNO_3, final composition x_{Ag} = 2%, x_{Cu} = 2% (EDX)	4 µg nanoparticles on GCE of Ø3 mm	Surface oxide reduction in 0.5 mol L^{-1} H_2SO_4, calculation with 450 µC cm^{-2}	1 mol L^{-1} MeOH + 0.5 mol L^{-1} KOH	5	1.30<br>0.25 V (SCE)	0.18	[21]	use of Nafion binder
Free corrosion of $Au_{16}Cu_{84}$ nanoparticles in 3 mol L^{-1} HNO_3, final composition x_{Cu} = 2% (EDX)	4 µg nanoparticles on GCE of Ø3 mm	Surface oxide reduction in 0.5 mol L^{-1} H_2SO_4, calculation with 450 µC cm^{-2}	1 mol L^{-1} MeOH + 0.5 mol L^{-1} KOH	5	1.30<br>0.25 V (SCE)	0.045	[21]	use of Nafion binder
Potentiostatic dealloying from $Ag_{72}Au_{28}$, final x_{Ag} < 1% (EDX), x_{Ag} = 16 % (XPS), protocol B	150 µm-thick disk	Capacitance measurement in 0.1 mol L^{-1} $HClO_4$ around 1.26 V (RHE), calculation with 40 µF cm^{-2}	1 mol L^{-1} MeOH + 1 mol L^{-1} KOH	10	1.47 V<br>0.532 V (Hg|HgO)	0.10	[22]	
Potentiostatic dealloying from $Ag_{75}Au_{25}$, final x_{Ag} < 1% (EDX), UPD deposition of Ag to surface content of x_{Ag} = 9% (XPS)	20 µm (CME)	Reduction of surface oxide formed up to 1.65 V (RHE), calculation with 386 µC cm^{-2}	1 mol L^{-1} MeOH + 0.1 mol L^{-1} NaOH	10	1.25	0.084	[23]	

Potentiostatic dealloying from $Ag_{75}Au_{25}$, final x_{Ag} < 1% (EDX)	20 µm (CME)	Reduction of surface oxide formed up to 1.65 V (RHE), calculation with 386 µC cm^{-2}	1 mol L^{-1} MeOH + 0.1 mol L^{-1} NaOH	10	1.20	0.042	23	
Porous gold nano bowls, x_{Ag} = 4%, from reduction of $HAuCl_4$ by hydroquinone in presence of poly(vinyl pyrrolidone) and AgCl nanocubes, removal of AgCl by NH_4OH	2 µg on GCE	Surface oxide reduction in 0.5 mol L^{-1} H_2SO_4, calculation with 450 µC cm^{-2}	2 mol L^{-1} MeOH + 0.5 mol L^{-1} KOH	20	1.18<br>0.17 V (Ag|AgCl)	0.133<br>27.8 µA µg^{-1}	24	Only mass activity reported, current density from mass-related current and A_{ECSA} (20.9 m^2 g^{-1});
$AuAg_{12}$ nanoparticles, non-aqueous synthesis with $AgNO_3$, cetyltrimethyl-ammonium bromide, octadecylamine, CuCl and addition of $HAuCl_4$	About 0.5 mg cm^{-2} on GCE	CO stripping in 0.1 M $HClO_4$, calculation with 450 µC cm^{-2}	2 mol L^{-1} MeOH + 0.5 mol L^{-1} KOH	20	1.21	0.42<br>20.9 mA mg$_{Au}^{-1}$	25	processed to ink with Ketjen Black and Nafion
Au_2Ag nanoparticles, non-aqueous synthesis using $AgNO_3$, cetyltrimethyl-ammonium bromide, octadecylamine, CuCl and addition of $HAuCl_4$	About 0.5 mg cm^{-2} on GCE	CO stripping in 0.1 M $HClO_4$, calculation with 450 µC cm^{-}	2 mol L^{-1} MeOH + 0.5 mol L^{-1} KOH	20	1.15	0.73<br>141 mA mg$_{Au}^{-1}$	25	processed to ink with Ketjen Black and Nafion
Au_3Ag nanoframes, non-aqueous synthesis using $AgNO_3$, cetyltrimethyl-ammonium bromide, octadecylamine, CuCl and addition of $HAuCl_4$	About 0.5 mg cm^{-2} on GCE	CO stripping in 0.1 M $HClO_4$, calculation with 450 µC cm^{-}	2 mol L^{-1} MeOH + 0.5 mol L^{-1} KOH	20	0.92	3.38<br>950 mA mg$_{Au}^{-1}$	25	processed to ink with Ketjen Black and Nafion
Ag-Au nanoparticles (NP_{AgAu}), Au seed-mediated (Ag) growth using certyl-trimethylammonium chloride (CTAC) as capping agent, x_{Ag} = 89 %	Nanoparticle ink on glassy carbon	Capacitance measurement in 0.1 M KOH	2 mol L^{-1} MeOH + 0.1 mol L^{-1} KOH		2.04<br>1.0 V (Ag|AgCl)	11.7 mA cm^{-2} mg^{-1}	26	Scan rate not reported, A_{ESCA} not reported, current at positive potential limit was evaluated,
Ag core – Au shell nanoparticles ($NP_{core-shell}$), NP_{AgAu} seed-mediated (Au) growth technique using certytrimethylammonium bromide (CTAB) as capping agent. x_{Ag} = 44 %	Nanoparticle ink on glassy carbon	Capacitance measurement in 0.1 M KOH	2 mol L^{-1} MeOH + 0.1 mol L^{-1} KOH		2.04<br>1.0 V (Ag|AgCl)	28.2 mA cm^{-2} mg^{-1}	26	Scan rate not reported, A_{ESCA} not reported, current at positive potential limit was evaluated.
Porous alloyed nanoparticles (NP_{alloy}), $NP_{core-shell}$ and galvanic replacement technique by addition of Au^{3+} into the NP suspension, x_{Ag} = 32 %	Nanoparticle ink on glassy carbon	Capacitance measurement in 0.1 M KOH	2 mol L^{-1} MeOH + 0.1 mol L^{-1} KOH		2.04<br>1.0 V (Ag|AgCl)	88.8 mA cm^{-2} mg^{-1}	26	Scan rate not reported, A_{ESCA} not reported, current at positive potential limit was evaluated.
Planar polycrystalline Au	planar	Surface oxide formation to Burshtein minimum	1.5 mol L^{-1} MeOH in 0.01 mol L^{-1} KOH	10	1.25<br>0.3 V (SCE)	0.076	27	

[a] Peak potential as reported in the paper is given in gray below the value recalculated for RHE

[b] Related to the surface area measurement if not stated otherwise. If applicable the quantity as reported in the paper is given in gray below the peak current density

REFERENCES

(1) Soyarslan, C.; Bargmann, S.; Pradas, M.; Weissmüller, J. 3D Stochastic Bicontinuous Microstructures: Generation, Topology and Elasticity. *Acta Mater.* **2018**, *149*, 326–340. https://doi.org/10.1016/j.actamat.2018.01.005.

(2) Debye, P.; Anderson, H. R.; Brumberger, H. Scattering by an Inhomogeneous Solid. II. The Correlation Function and Its Application. *J. Appl. Phys.* **1957**, *28*, 679–683. https://doi.org/10.1063/1.1722830.

(3) Warren, B. E. *X-Ray Diffraction*; Dover Publications, New York, 1990.

(4) Richert, C.; Huber, N. A Review of Experimentally Informed Micromechanical Modeling of Nanoporous Metals: From Structural Descriptors to Predictive Structure-Property Relationships. *Materials* **2020**, *13*, 3307. https://doi.org/10.3390/ma13153307.

(5) Richert, C.; Odermatt, A.; Huber, N. Computation of Thickness and Mechanical Properties of Interconnected Structures: Accuracy, Deviations, and Approaches for Correction. *Front. Mater.* **2019**, *6*. https://doi.org/10.3389/fmats.2019.00327.

(6) Mameka, N. Surface-Controlled Mechanical Properties of Bulk Nanoporous Gold. Dissertation, Dissertation, Hamburg University of Technology, Hamburg, 2016. https://doi.org/10.15480/882.1328.

(7) Conway, B. E. *Electrochem Data*; Greenwood Press Publishers.

(8) Trasatti, S. The Absolute Electrode Potential: An Explanatory Note. *Pure Appl. Chem.* **1986**, *57*, 955–966. https://doi.org/10.1351/pac198658070955.

(9) Hamelin, A. Cyclic Voltammetry at Gold Single-Crystal Surfaces. Part 1. Behaviour at Low-Index Faces. *J. Electroanal. Chem.* **1996**, *407*, 1–11. https://doi.org/10.1016/0022-0728(95)04499-X.

(10) Hamelin, A.; Martins, A. M. Cyclic Voltammetry at Gold Single-Crystal Surfaces. Part 2. Behaviour of High-Index Faces. *J. Electroanal. Chem.* **1996**, *407*, 13–21. https://doi.org/10.1016/0022-0728(95)04500-7.

(11) Kondo, T.; Morita, J.; Hanaoka, K.; Takakusagi, S.; Tamura, K.; Takahasi, M.; Mizuki, J.'i.; Uosaki, K. Structure of Au(111) and Au(100) Single-Crystal Electrode Surfaces at Various Potentials in Sulfuric Acid Solution Determined by In Situ Surface X-ray Scattering. *J. Phys. Chem. C* **2007**, *111*, 13197–13204. https://doi.org/10.1021/jp072601j.

(12) Dakkouri, A. S.; Kolb, D. M. Reconstruction of Gold Surface. In *Interfacial Electrochemistry: Theory, Experiment and Application*; Wieckowski, A., Ed.; M. Dekker: New York, 1999; pp 151–173.

(13) Hernández, J.; Solla-Gullón, J.; Herrero, E.; Aldaz, A.; Feliu, J. M. Methanol Oxidation on Gold Nanoparticles in Alkaline Media: Unusual Electrocatalytic Activity. *Electrochim. Acta* **2006**, *52*, 1662–1669. https://doi.org/10.1016/j.electacta.2006.03.091.

(14) Hamelin, A.; Weaver, M. J. Metal Substrate Effects upon the Kinetics of Simple Electrochemcial Reactions: The Reduction of Cobalt(III) Ammines at Single-Crystal Gold Faces. *J. Electroanal. Chem.* **1986**, *209*, 109–124. https://doi.org/10.1016/0022-0728(86)80190-5.

(15) Hamelin, A. Underpotential Deposition of Lead on Single Crystal Faces of Gold: Part I. The Influence of Crystallographic Orientation of the Substrate. *J. Electroanal. Chem.* **1984**, 167–180. https://doi.org/10.1016/S0022-0728(84)80095-9.

(16) Hamelin, A.; Lipkowski, J. Underpotential Deposition of Lead on Gold Single Crystal Faces: Part II: General Discussion. *J. Electroanal. Chem.* **1984**, *171*, 317–330. https://doi.org/10.1016/0022-0728(84)80123-0.

(17) Hernández, J.; Solla-Gullón, J.; Herrero, E. Gold Nanoparticles Synthesized in a Water-in-Oil Microemulsion: Electrochemical Characterization and Effect of the Surface Structure on the Oxygen Reduction Reaction. *J. Electroanal. Chem.* **2004**, *574*, 185–196. https://doi.org/10.1016/j.jelechem.2003.10.039.

(18) Hernández, J.; Solla-Gullón, J.; Herrero, E.; Feliu, J. M.; Aldaz, A. In Situ Surface Characterization and Oxygen Reduction Reaction on Shape-Controlled Gold Nanoparticles. *J. Nanosci. Nanotechnol.* **2009**, *9*, 2256–2273. https://doi.org/10.1166/jnn.2009.SE38.

(19) Vega, A. A.; Newman, R. C. Methanol Electro-Oxidation on Nanoporous Metals Formed by Dealloying of Ag–Au–Pt Alloys. *J. Appl. Electrochem.* **2016**, *46*, 995–1010. https://doi.org/10.1007/s10800-016-0978-5.

(20) Wang, X.; Frenzel, J.; Wang, W.; Ji, H.; Qi, Z.; Zhang, Z.; Eggeler, G. Length-Scale Modulated and Electrocatalytic Activity Enhanced Nanoporous Gold by Doping. *J. Phys. Chem. C* **2011**, *115*, 4456–4465. https://doi.org/10.1021/jp110011w.

(21) Li, G. G.; Lin, Y.; Wang, H. Residual Silver Remarkably Enhances Electrocatalytic Activity and Durability of Dealloyed Gold Nanosponge Particles. *Nano Lett.* **2016**, *16*, 7248–7253. https://doi.org/10.1021/acs.nanolett.6b03685.

(22) Graf, M.; Haensch, M.; Carstens, J.; Wittstock, G.; Weissmüller, J. Electrocatalytic Methanol Oxidation with Nanoporous Gold: Microstructure and Selectivity. *Nanoscale* **2017**, *9*, 17839. https://doi.org/10.1039/c7nr05124g.

(23) Silva Olaya, A. R.; Kühling, F.; Mahr, C.; Zandersons, B.; Rosenauer, A.; Weissmüller, J.; Wittstock, G. Promoting Effect of the Residual Silver on the Electrocatalytic Oxidation of Methanol and Its Intermediates on Nanoporous Gold. *ACS Catal.* **2022**, *8*, 4415–4429. https://doi.org/10.1021/acscatal.1c05160.

(24) Pedireddy, S.; Lee, H. K.; Koh, C. S. L.; Tan, J. M. R.; Tjiu, W. W.; Ling, X. Y. Nanoporous Gold Bowls: A Kinetic Approach to Control Open Shell Structures and Size-Tunable Lattice Strain for Electrocatalytic Applications. *Small* **2016**, *12*, 4531–4540. https://doi.org/10.1002/smll.201601371.

(25) Xiong, L.; Sun, Z.; Zhang, X.; Zhao, L.; Huang, P.; Chen, X.; Jin, H.; Sun, H.; Lian, Y.; Deng, Z.; Rümmerli, M. H.; Yin, W.; Zhang, D.; Wang, S.; Peng, Y. Octahedral Gold-Silver Nanoframes with Rich Crystalline Defects for Efficient Methanol Oxidation Manifesting a CO-Promoting Effect. *Nat. Commun.* **2019**, *10*, 3782. https://doi.org/10.1038/s41467-019-11766-w.

(26) De, S. K.; Nandy, A.; Mondal, S.; Roy, A.; Mondal, S.; Senapati, D. Void-Enriched and Highly Strained Porous Au–Ag Nanoalloy as a Bifunctional Electro-Catalyst in Alkaline Direct Alcohol Fuel Cell. *ACS Appl. Energy Mater.* **2021**, *4*, 5367–5374. https://doi.org/10.1021/acsaem.1c00475.

(27) Borkowska, Z.; Tymosiak-Zielinska, A.; Shul, G. Electrooxidation of Methanol on Polycrystalline and Single Crystal Gold Electrodes. *Electrochim. Acta* **2004**, *49*, 1209–1220. https://doi.org/10.1016/j.electacta.2003.09.046.
